# Enzymatic glycosylation and amidation reshapes polyene bioactivity

**DOI:** 10.1038/s41586-026-10834-8

**Published:** 2026-07-29

**Authors:** Saadia N. Mirza, Alberto Carella, Joseph W. Thompson, Deepanjan Panda, Anna R. I. McDonald, Matthew D. Crossley, Wei Li Thong, Katherine J. Robins, Sarah. A. Shepherd, Matthew J. Cliff, Clara Valero, Andrew Thom, Michael Bromley, Jason Micklefield

**Affiliations:** 1 Department of Chemistry, Molecular Sciences Research Hub, Imperial College, London, UK; 2 Manchester Institute of Biotechnology, University of Manchester, Manchester, UK; 3 Manchester Fungal Infection Group, University of Manchester, Manchester, UK

**Keywords:** Biosynthesis, Biocatalysis, Natural products

## Abstract

Fungal diseases are an escalating threat to human health, driven by rising antimicrobial resistance and a lack of new antifungal treatments^1,2^. Polyenes, a class of complex natural products, have been widely used as antifungal agents due to their broad-spectrum activity^3^. However, their clinical use is limited by severe toxicity and poor solubility, making safer and more effective alternatives urgently needed^4^. Previous efforts to generate improved polyenes have relied on costly, step-inefficient and atom-inefficient chemical syntheses^5,6^. Here we describe the discovery and characterization of pathways to previously undescribed polyenes, including unusual glycosyltransferase enzymes that introduce sugars onto polyene scaffolds. We also show how the reaction scope of an amidotransferase enzyme can be expanded to transform the detrimental carboxylate substituent of polyenes to alternative functionality. Introduction of a second sugar combined with carboxylate modifications leads to more potent polyene antifungals, with reduced toxicity, that are accessible by clean and efficient fermentation or enzymatic routes.

## Abstract (web summary)

Pathways to previously undescribed polyenes, including unusual enzymes that introduce more sugars onto polyene scaffolds, are discovered, and their accessibility through clean and efficient fermentation and potential for safe use as antifungal treatments is described.

## Main

**Fig. 1:**
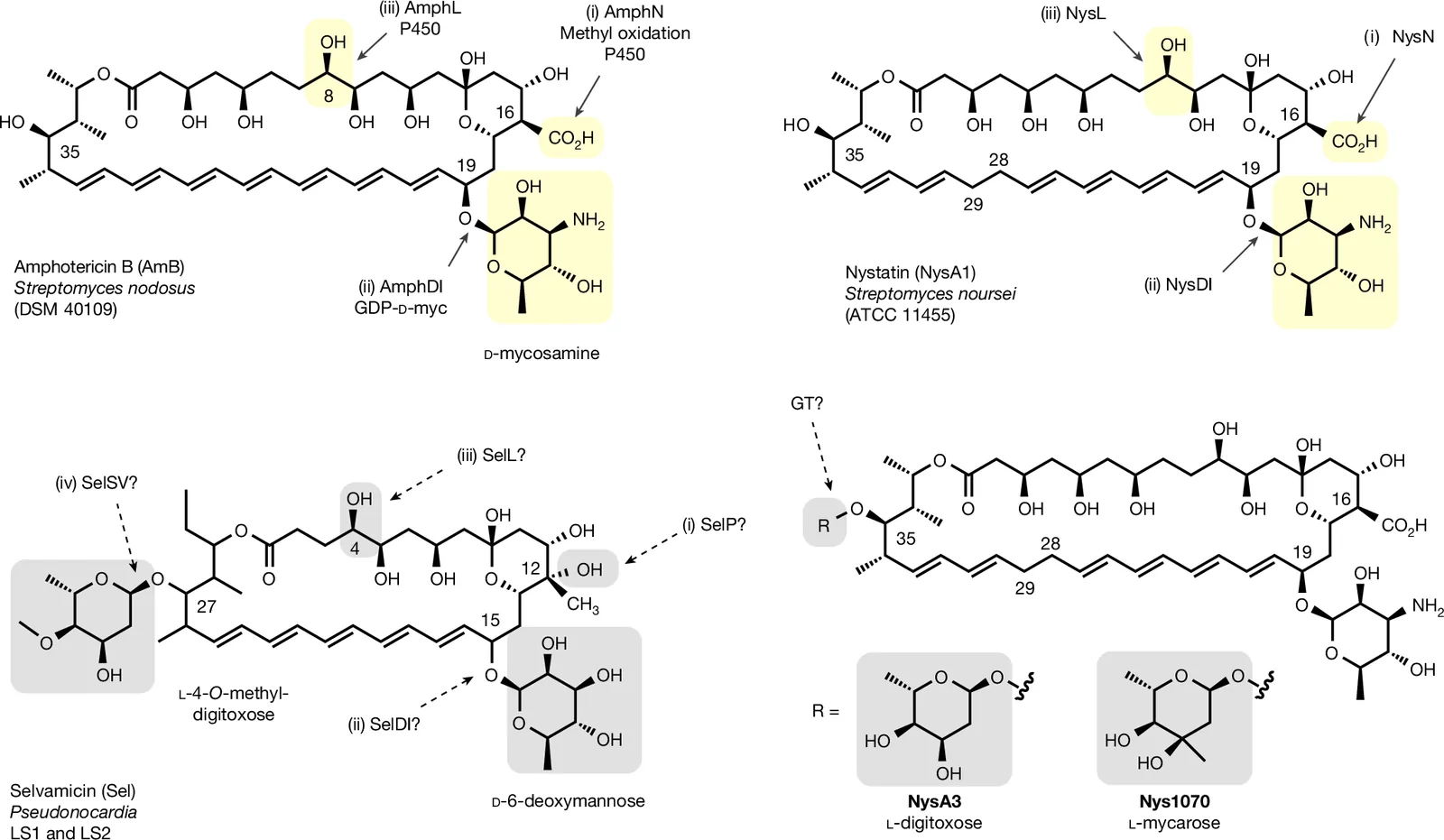
Polyenes and post-PKS tailoring enzymes. Confirmed tailoring steps in yellow for AmB and NysA1 include oxidation of a C16-methyl substituent by cytochrome P450 (AmphN, NysN), to a carboxylic acid group. This is followed by C19 glycosylation (AmphDI or NysDI) and then C8 or C10 hydroxylation by a second P450 (AmphL or NysL). The GT enzyme(s) required for addition of the second C35 sugar of NysA3 and Nys1070 have not been identified. Proposed tailoring steps in selvamicin biosynthesis are similar, in which C15 glycosylation and C4 hydroxylation are carried out by SelDI and SelL, respectively, except an Fe(II)/2-oxoglutarate dependent enzyme (SelP) is predicted to hydroxylate C12, and SelSV is postulated to install 4-*O*-methyl-l-digitoxose^23^.

The rising prevalence of life-threatening fungal infections, coupled with emerging resistance against most clinically approved antifungal agents, represents a major global health problem^1,2,7–12^. With few new classes of antifungal drugs in development^9–11^, amphotericin B (AmB), nystatin A1 (NysA1) and related polyenes remain frontline treatments for fungal infections. Polyenes are complex polyketide natural products with broad-spectrum antifungal activity^3,13^ (Fig. 1). In addition, AmB is the main treatment for leishmania, a parasitic disease common in the global south^14,15^. Although polyenes have largely evaded resistance even after extensive clinical use^16^, they suffer from high toxicity and low solubility^17,18^. The amphiphilic polyenes are suggested to act as a ‘sterol sponge’, extracting ergosterol from the microbial cell membrane giving rise to their antimicrobial activity^4,19^. Similar interactions with cholesterol in human cell membranes are thought to be responsible for polyene toxicity^20^. Less toxic liposomal formulations of AmB have been developed^21^, but these are expensive to produce, limiting availability in the less affluent regions of the world. The provision of more effective, safer and affordable antifungal drugs remains a critical unmet need.

Glycosylation plays a key role in modulating both the activity and solubility of polyenes. Most polyenes have a mycosamine sugar, present at C19 in AmB and NysA1 (Fig. 1), that is essential for bioactivity^22^. A few polyenes have been identified with multiple glycosyl units, including selvamicin (Fig. 1) and most recently mandimycin^23,24^. Although their biosynthetic gene clusters (BGC) have been sequenced, none of the enzymes from these pathways have been characterized. Nystatin congeners NysA3 and Nys1070 are also proposed to possess a second C35 sugar but they were isolated in very low quantities, preventing further development^25,26^. Furthermore, semi-synthetic polyene derivatives have been produced that include extra sugar moieties or other groups that improve solubility and reduce toxicity^27^. Furthermore, the C16-carboxylate group in AmB, NysA1 and most other polyenes also contributes to polyene toxicity^27–29^, and synthetic derivatives with alternative substituents, including amides, at C16 have been shown to have reduced toxicity^5,6,27,30^. Because polyenes are complex molecules, their synthetic modification typically requires many steps, extensive use of protecting groups and deleterious reagents. For example, a very promising AmB derivative that includes a modified sugar and a C16-amide moiety was recently introduced^6^, with considerably reduced toxicity. However, this ‘renal-sparing’ AmB derivative required 12 chemical steps to synthesize in 0.7% overall yield from AmB^6^, which is difficult to produce itself. The development of more atom and step efficient methods for producing polyene derivatives that are not reliant on toxic and/or expensive reagents, would be highly desirable in future endeavours to deliver improved and affordable polyene drugs.

Here we report genome mining studies leading to the discovery of new polyene natural products and pathways, including previously undescribed glycosyltransferase (GT) enzymes. We demonstrate that these newly discovered GTs can be used to produce polyene derivatives, in vitro and in vivo, with several different glycosyl groups. We also expand the substrate and reaction scope of an amidotransferase to functionalize the detrimental carboxylic acid substituent common to most polyenes. The addition of a second sugar, combined with carboxylate modification, leads to new polyene derivatives with increased antifungal activity, lower toxicity and higher solubility than the parent polyenes used at present in the clinic. Our enzymatic approach is also more efficient and cleaner than the synthetic methods used so far to produce improved polyenes.

## Discovery of polyene GTs

**Fig. 2:**
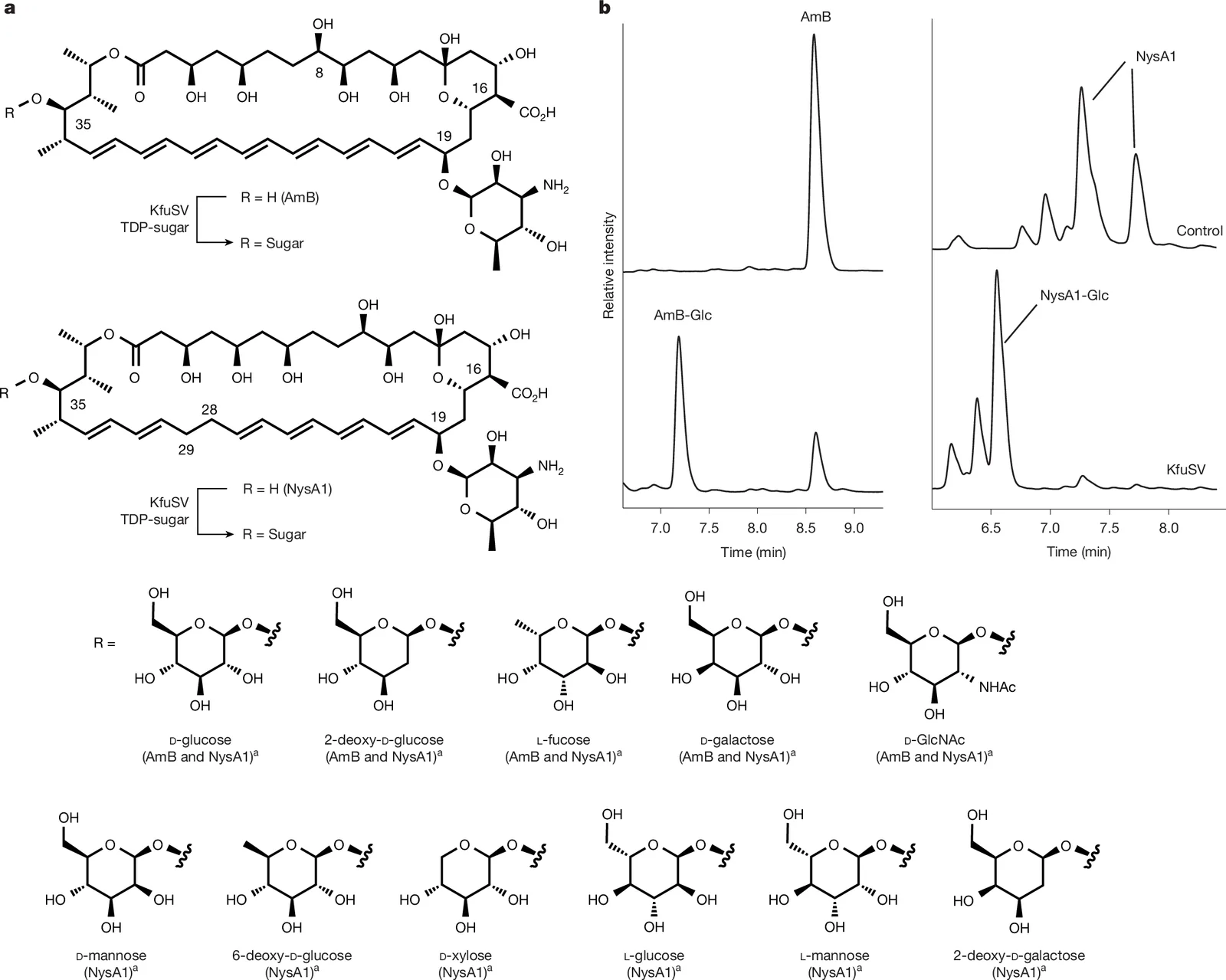
Glycosylation of AmB and NysA1 by SelSV, NysSV, MycS3 or KfuSV. **a**, Glycosylated variants of AmB and NysA1 generated using KfuSV and TDP sugars. A set of 19 TDP sugars were tested, of which 11 were shown to be substrates for KfuSV (also see Extended Data Fig. 1). The regioselectivity of the GTs was confirmed in reverse glycosylation assays with native products (Fig. 3). ^a^Sugar added to AmB and/or NysA1. **b**, RP-HPLC chromatogram showing conversion of AmB and NysA1 into glucosylated derivatives by KfuSV. Control traces refer to substrates incubated in the absence of KfuSV.

**Extended Data Fig. 1:**
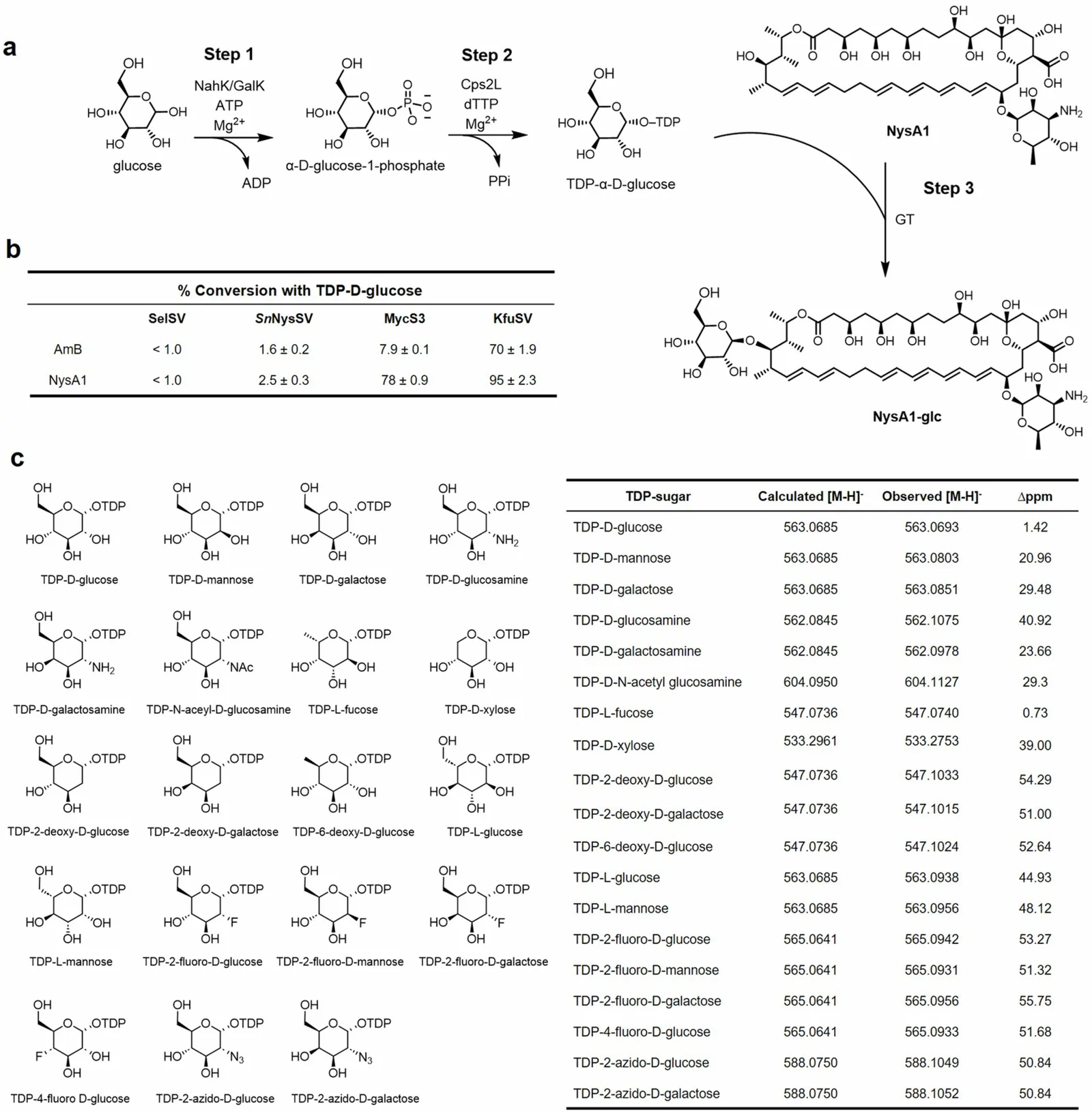
(**a**) Three step reaction used for glycodiversification of polyenes, with glucosylation of NysA1 shown in this example. (**b**) Comparison of % conversion of AmB and NysA1 with TDP-d-glucose and the four GTs. Glucosylated products from SelSV were detected in trace amounts via LC-HRMS. For other enzymes, the data is in triplicate and means with standard deviations are included. (**c**) HRMS data for TDP-sugars produced by NahK/GalK and Cps2L cascade reactions. The production of 19 TDP-sugars was confirmed by HRMS. These TDP-sugars were then used to test the substrate scope of KfuSV with AmB and NysA1 in the cascade reactions. Source data

Initially, we sought to characterize the GT enzyme that installs the second sugar (4-*O*-methyl-l-digitoxose) of selvamicin (Fig. 1). Although selvamicin has low antifungal activity^23^, we reasoned that this GT may be useful for derivatizing the more therapeutically relevant polyenes AmB and NysA1. The selvamicin BGC has a TDP-l-digitoxose (thymidyl diphosphate-l-digitoxose) subcluster with a putative GT enzyme, SelSV, that was predicted to add the second sugar^23^. As SelSV’s function has not been determined experimentally, we overproduced the enzyme in *Escherichia coli* and, in the absence of the putative native aglycone and TDP-sugar substrates, we tested its in vitro activity using TDP-d-glucose together with either AmB or NysA1 as substrates (Extended Data Fig. 1). Liquid chromatography with high-resolution mass spectrometry (LC–HRMS) analysis revealed products consistent with the addition of glucose to AmB and NysA1 (Supplementary Fig. 1). Only very low levels (less than 1%) of the glucose-polyenes were produced, probably due to the substantial differences between the native SelSV substrates and those used in the assays (Extended Data Fig. 1b). The SelSV sequence was therefore used to identify homologues that might accommodate larger polyenes. First, we explored *Streptomyces noursei* (American Type Culture Collection (ATCC) 11455), which in addition to NysA1 is reported to produce low levels of the NysA3 and Nys1070 congeners, each with a second C35 sugar^25,26^ (Fig. 1 and Supplementary Table 1). Although the nystatin BGC encodes a single GT (NysDI) that introduces d-mycosamine, there are no genes present in the BGC, or elsewhere on the *S. noursei* (ATCC 11455) genome, that have been shown to encode enzymes responsible for the incorporation of second sugars in NysA3 and Nys1070. Analysis of the *S. noursei* genome sequence revealed a candidate GT, which we named *Sn*NysSV, that is present in a small cluster of genes resembling the SelSV subcluster that we predicted may introduce l-digitoxose onto C35 of NysA1 (Supplementary Fig. 2 and Supplementary Table 2). Wider bioinformatics analysis revealed that several strains possess similar putative GTs, along with polyene-like BGCs, and three of these that we named *Sa*NysSV, MycS3 and KfuSV, were selected for investigation (Supplementary Fig. 3 and Supplementary Table 3). *Sa*NysSV from *Streptomyces albulus* (DSM40492) shows very high identity to *Sn*NysSV (98% id). Genes *mycS3* from *Streptomyces netropsis* (DSM40846) and *kfuSV* from *Streptomyces kasugaensis* (DSM40819) were both located in previously unreported polyene-like BGCs. The putative GTs were overproduced in *E. coli* and assayed in vitro with TDP-d-glucose and AmB or NysA1. All showed higher activity than SelSV, with KfuSV showing the highest activity with both AmB and NysA1 (Fig. 2 and Extended Data Fig. 1b). KfuSV was also shown to glycosylate three more polyene scaffolds with TDP-d-glucose (Supplementary Fig. 4). The sugar substrate scope of KfuSV was further explored with AmB or NysA1, using tandem enzyme assays^31^ to generate 19 TDP sugars from available sugar precursors (Extended Data Fig. 1). Glycosylated polyene derivatives could be detected by LC–HRMS with 11 of the 19 TDP sugars tested (Fig. 2 and Supplementary Figs. 5 and 6), which indicates KfuSV is a particularly flexible enzyme for polyene glycodiversification.

## NysSV is responsible for NysA3 production

**Fig. 3:**
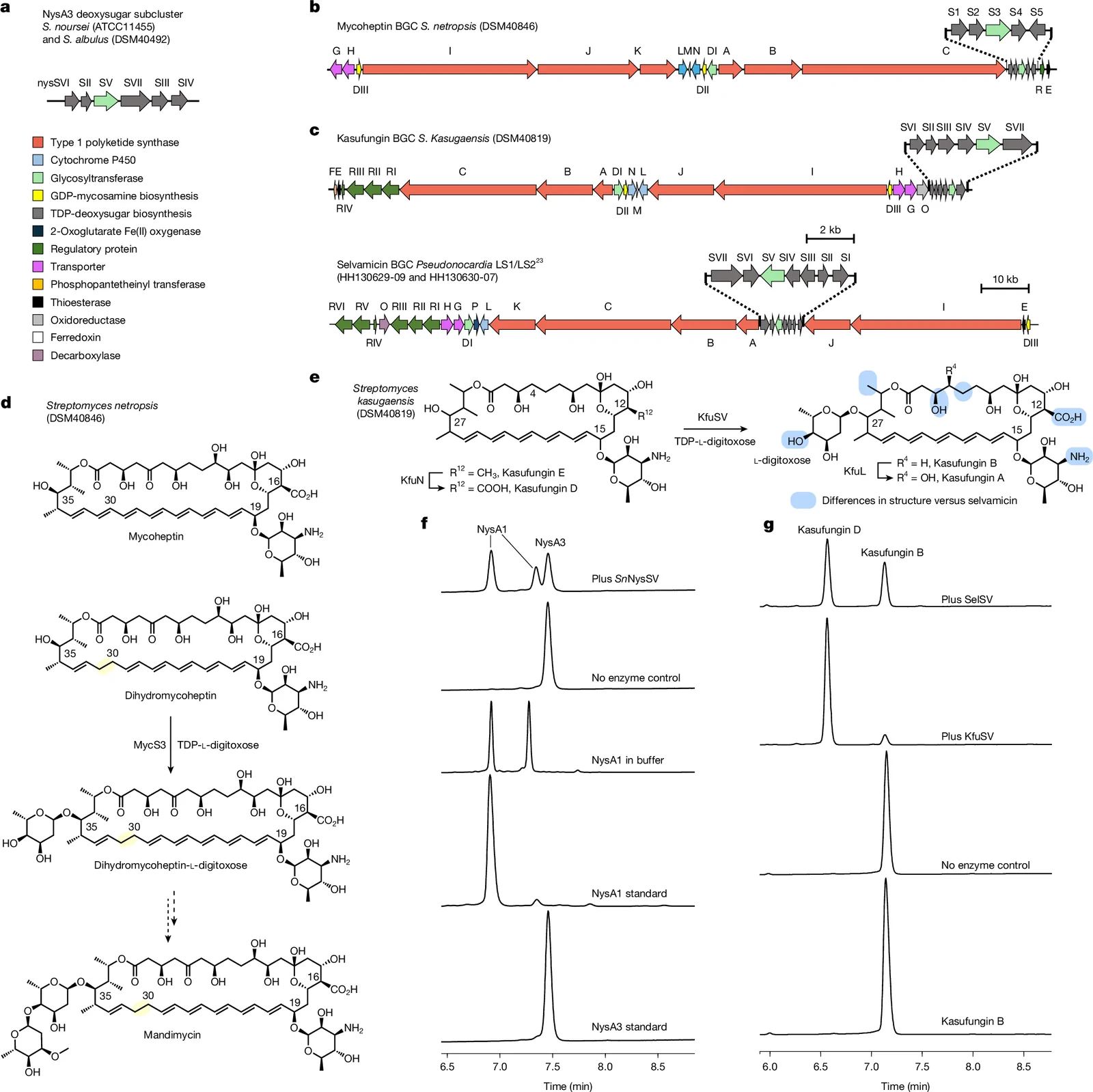
Organization of BGCs and reverse glycosylation reactions confirming the function of NysSV, MycS3 and KfuSV in polyene biosynthesis. **a**, The NysSV-containing deoxysugar cluster in *S. noursei* and *S. albulus*. **b**, The mycoheptin and mandimycin BGC. **c**, Comparison of the kasufungin and selvamicin BGC. **d**, Glycosylation reaction of MycS3 in mandimycin biosynthesis. **e**, Final steps in the biosynthesis of kasufungin A. The differences in structure between kasufungin A and selvamicin are highlighted in blue. **f**, HPLC analysis of the reverse glycosylation reaction of NysA3 catalysed by *Sn*NysSV. **g**, HPLC analysis of reverse glycosylation of kasufungin B catalysed by KfuSV.

*Sa*NysSV is found on the *S. albulus* genome within a small cluster of six genes that we predict produce TDP-l-digitoxose, and which have high identity to the *Sn*NysSV cluster in *S. noursei* (more than 95%) (Fig. 3a). Neither NysSV clusters are associated with any other identifiable BGCs. Further analysis of the *S. albulus* genome sequence revealed a separate putative nystatin-producing BGC elsewhere on the chromosome. *S. albulus* has also been reported to produce low quantities of NysA1 (ref. ^32^), but there are no reports of this strain producing diglycosylated nystatin variants. LC–HRMS analysis of *S. albulus* fermentation supernatant showed a minor amount of NysA1, with the main polyene product possessing a mass consistent with NysA3 (Supplementary Fig. 7). Nuclear magnetic resonance (NMR) characterization of NysA3 has not been reported and its structure was based mainly on degradation studies^33,34^. We therefore purified the main product from *S. albulus* and carried out detailed NMR analysis, confirming the presence of C35-l-digitoxose consistent with the previously proposed structure for NysA3 (refs. ^33,34^) (Supplementary Fig. 8).

**Extended Data Fig. 2:**
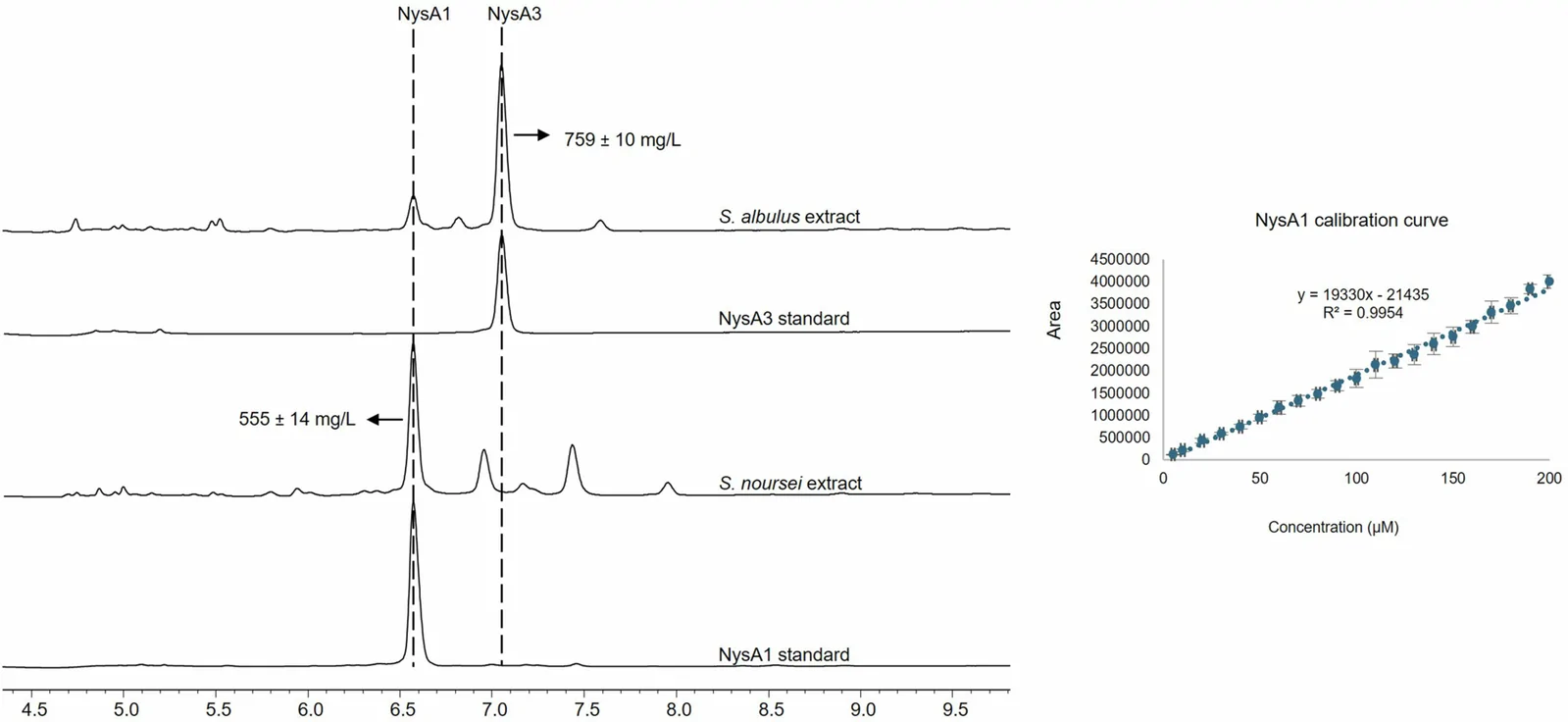
Comparison of NysA1 and NysA3 production from *S. noursei* and *S. albulus.* HPLC traces show that *S. albulus* produces NysA3 as a major fermentation product. *S. noursei* produces NysA1 as a major product under the same fermentation conditions. The relative titres of NysA3 and NysA1 were calculated by calibration curve using NysA1 commercial standard as shown. The experiments were performed in (n = 3) biological replicates as three independent fermentations for each organism and the error represented as ± SD. Source data

The fact that both *S. albulus* and *S. noursei* produce NysA3 and possess similar *nysSV* clustered with TDP-l-digitoxose biosynthesis genes, albeit remote from the main Nys BGCs, suggests that NysSV is responsible for the production of NysA3. To test this, we incubated NysA3 and TDP with both NysSV enzymes, which resulted in the production of NysA1 (Fig. 3f and Supplementary Fig. 9). Given that GT-catalysed reactions are reversible^35^, the fact that both NysSV enzymes remove l-digitoxose from the C35 position with high efficiency fully supports their role in the biosynthesis of NysA3. Furthermore, the entire *Sa*NysSV subcluster was expressed in a heterologous host *Streptomyces albus* (J1074). NysA1 was then incubated with lysate from the resulting *S. albus::nysSV-subcluster* strain that resulted in production of NysA3, providing further evidence that the NysSV subcluster produces l-digitoxose and transfers this sugar to C35 of NysA1 (Supplementary Fig. 10). The observation that the NysSV subclusters and nystatin BGC are at separate chromosomal loci is notable given genes for most reported bacterial natural product pathways are clustered together^36,37^. We also showed that *S. albulus* produces very high levels of NysA3 (759 ± 10 mg l^−1^) with only a minor quantity of NysA1 (Extended Data Fig. 2). By contrast, *S. noursei* produces NysA1 as the main product (555 ± 14 mg l^−1^ in our hands). Targeted gene expression analysis (quantitative polymerase chain reaction with reverse transcription, RT–qPCR) in the two nystatin-producing strains showed that the NysSV subcluster is expressed to a higher level in *S. albulus* than in *S. noursei*, which could account for the higher level of NysA3 production we observe in *S. albulus* (Supplementary Fig. 11). The fact that *S. albulus* produces higher levels of a rare polyene with a second sugar that improves solubility, and may affect other properties, makes this a useful production host.

## MycS3 glycosylates dihydromycoheptin

**Extended Data Fig. 3:**
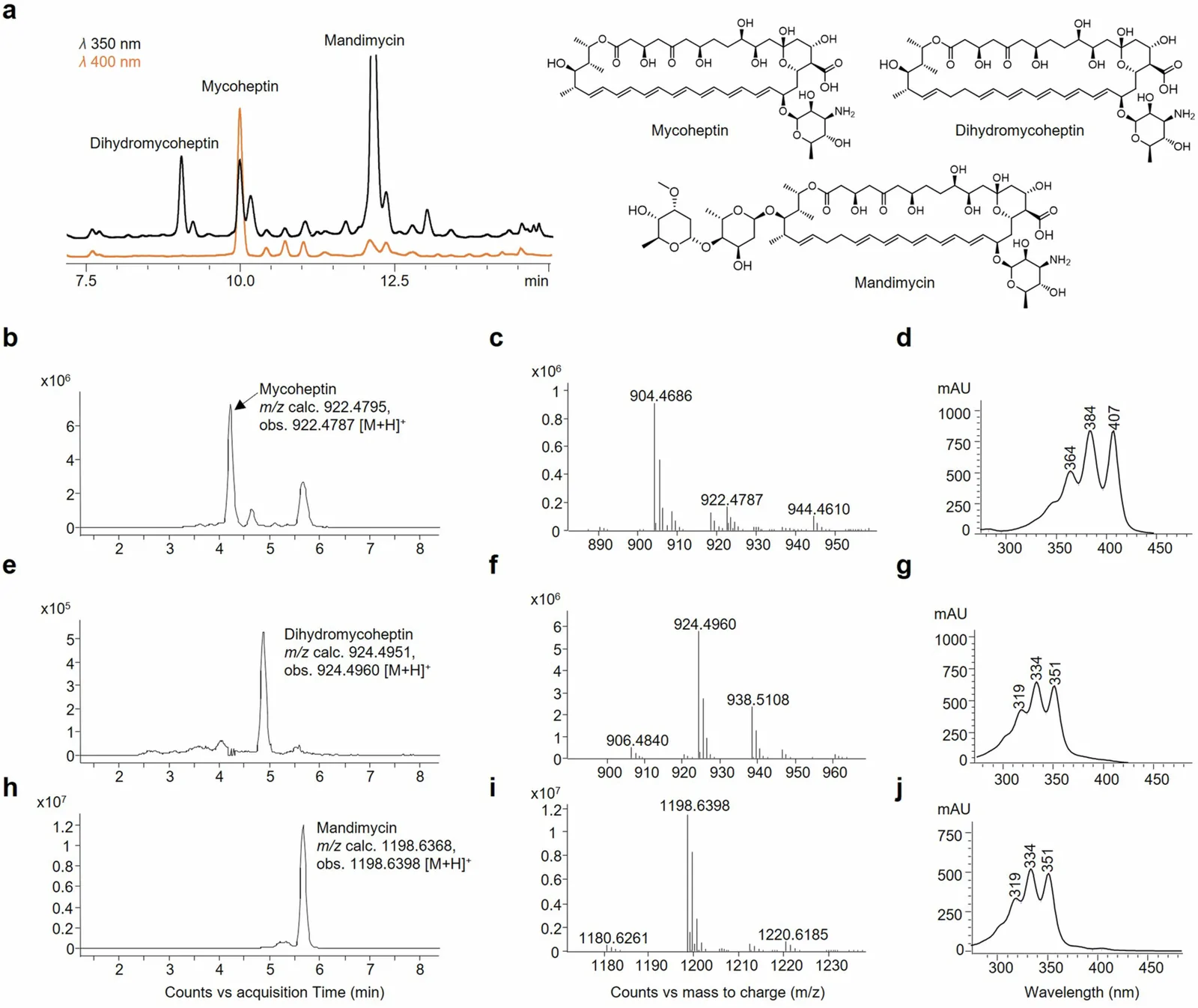
LC-HRMS analysis of polyenes produced by *S. netropsis* (DSM 40846). (**a**) RP-HPLC with diode array detection at 350 nm for pentaenes and 405 nm for heptaenes. The extracted ion chromatogram, observed parent ions (m/z), and UV absorbance spectrum of mycoheptin (**b**-**d**), dihydromycoheptin (**e**-**g**) and mandimycin (**h**-**j**).

MycS3 was found in *S. netropsis* (DSM40846), which is reported to produce mycoheptin, structurally related to AmB^38–40^ (Fig. 3b,d). Mycoheptin possesses the common C19-d-mycosamine but does not have a second sugar. We used PacBio to re-sequence and assemble the *S. netropsis* (DSM40846) genome de novo, which revealed a large 115 kb BGC that we named *myc*. This BGC includes 6 polyketide synthase (PKS) genes composed of 19 modules and several tailoring enzymes predicted to produce mycoheptin. Also present within the BGC is a putative TDP-deoxysugar subcluster that includes *mycS3* (Fig. 3b and Supplementary Fig. 12). Analysis of *S. netropsis* culture supernatant revealed a main product, which was isolated and confirmed by NMR to correspond with the previously determined structure of mycoheptin^40^ (Fig. 3d and Supplementary Fig. 13). LC–HRMS, UV–visible light (UV–Vis) and NMR analysis also revealed the presence of low quantities of a dihydromycoheptin congener, which has undergone reduction of the C30=C31 double bond (Extended Data Fig. 3 and Supplementary Fig. 14). Another pentaene congener was also isolated and shown by NMR experiments to possess two l-digitoxose residues at C35, sharing the same structure as mandimycin that was recently isolated from a different strain^24^ (Supplementary Fig. 15). To explore its function in vitro, MycS3 was incubated with dihydromycoheptin and TDP-l-digitoxose, which resulted in addition of a single l-digitoxose (Supplementary Fig. 16). The product of this assay is also evident in very low quantities from LC–HRMS analysis of *S. netropsis* (DSM40846) extracts, suggesting it is an intermediate on the pathway to mandimycin (Fig. 3d).

## KfuSV is involved in kasufungin biosynthesis

**Extended Data Fig. 4:**
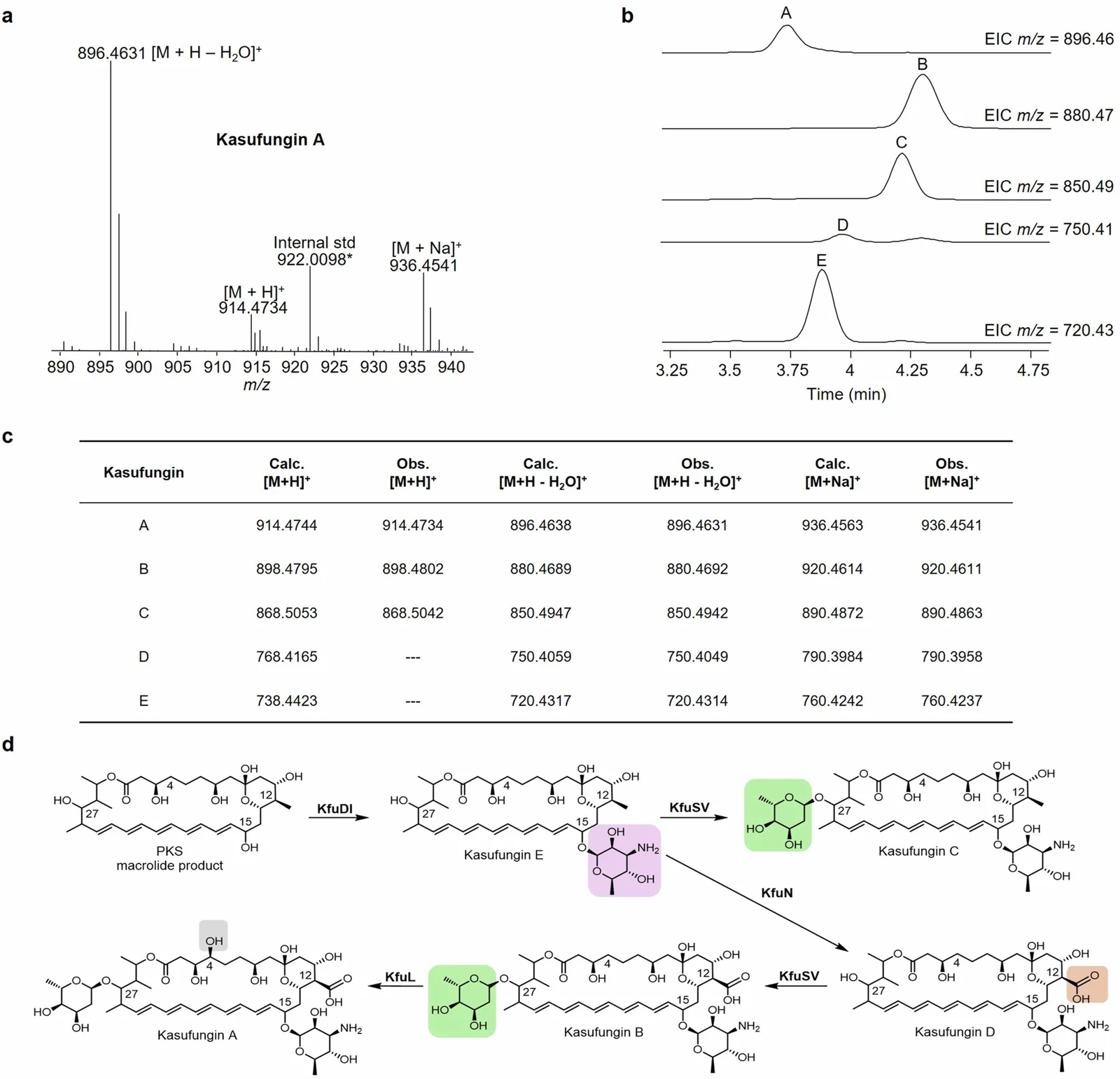
LC-HRMS analysis of kasufungins. (**a**) HRMS of kasufungin A. (**b**) LC-HRMS analysis of kasufungin A-E showing extracted ion chromatograms (ESI positive mode) for [M + H-H_2_O]^+^ (**c**) Table of observed and calculated masses for kasufungin A-E. (**d**) Proposed order of tailoring steps for kasufungin biosynthesis. The structures of kasufungin A-E are based on bioinformatics, LC-HRMS and subsequent NMR analysis (Supplementary Fig. 18). The proposed C15 glycosylation by KfuDI is shown in purple; C27 glycosylation by KfuSV highlighted in green; proposed oxidation of the C12-CH3 by KfuN indicated in peach and proposed site of hydroxylation by KfuL is shown in grey.

*S. kasugaensis* is known to produce aminoglycosides^41^, but has not been reported to produce polyenes. Analysis of the *S. kasugaensis* genome sequence did, however, reveal a single polyene-like BGC that includes *kfuSV* (Fig. 3c and Supplementary Fig. 17a). This BGC (that we named *kfu*) is similar in organization to the selvamicin BGC^23^, with five genes encoding a 15 module PKS, a subcluster containing *kfuSV* and genes predicted to be involved in the biosynthesis of TDP-l-digitoxose. Further detailed bioinformatics analysis enabled a structure of the *kfu* PKS macrolactone core to be proposed (Supplementary Fig. 17b–d). Reverse-phase-high-performance liquid chromatography (RP-HPLC) and LC–HRMS analysis of *S. kasugaensis* fermentation extracts revealed the production of five polyenes with UV–Vis absorbance typical of pentaenes (Extended Data Fig. 4). On the basis of the predicted structures of the five polyenes, we named these kasufungin A–E and a pathway was proposed (Fig. 3e and Extended Data Fig. 4). Although kasufungin A is the most functionalized (likely to be the end product), kasufungin B is most abundant and was therefore isolated and subjected to detailed NMR analysis, which shows it possesses a unique structure (Supplementary Fig. 18). In addition to differences in the macrolide structure (at C3, C5, C12 and C29), kasufungin B has different sugars (d-mycosamine and l-digitoxose) compared with selvamicin.

**Extended Data Fig. 5:**
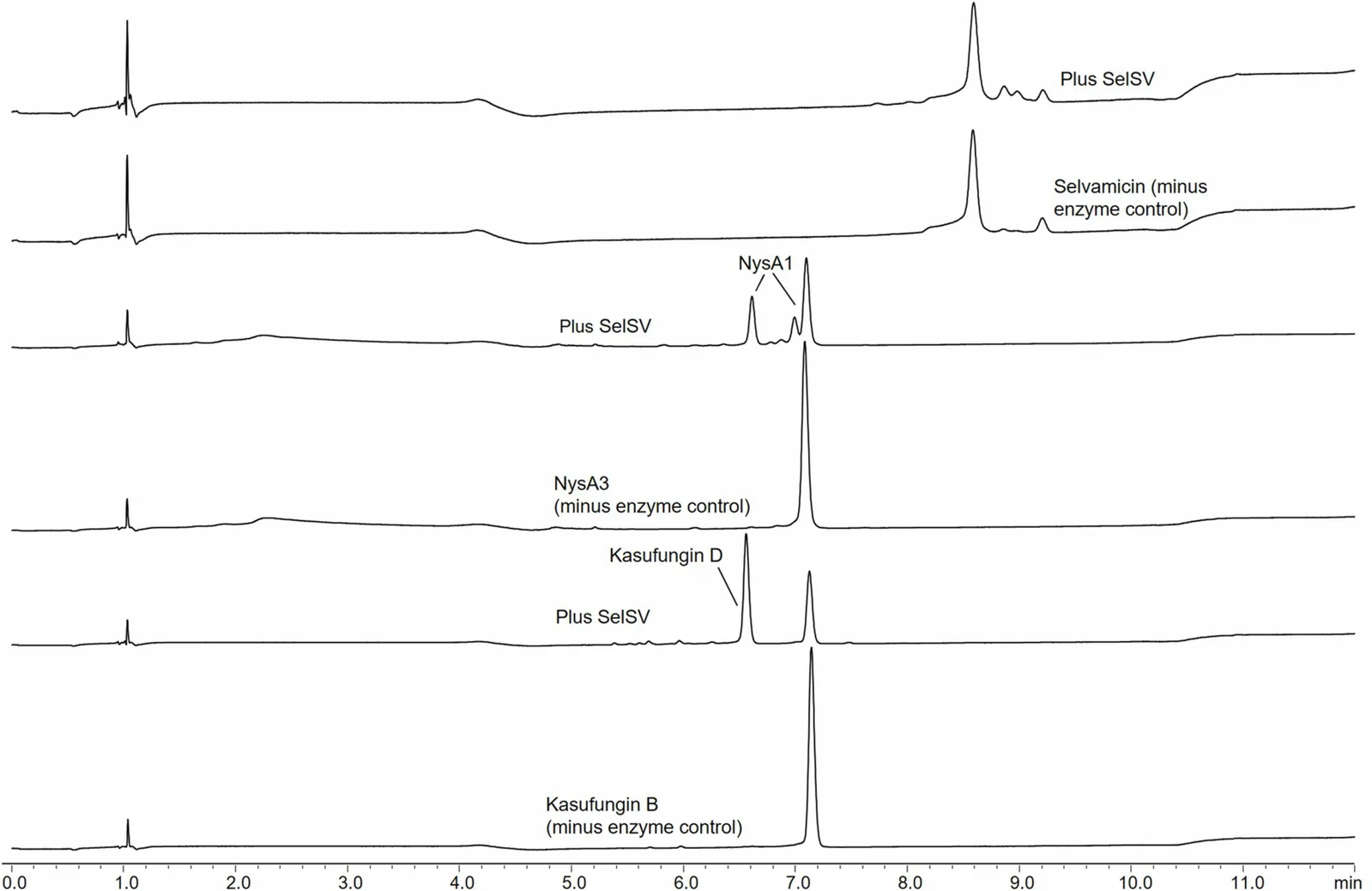
SelSV catalysed reverse glycosylation. HPLC traces show SelSV fails to perform reverse glycosylation on selvamicin (native substrate). However, SelSV does catalyse the reverse glycosylation reaction with kasufungin B and NysA3, generating kasufungin D and NysA1, respectively.

Kasufungin B was then incubated with KfuSV and TDP, which resulted in the formation of kasufungin D, confirming its role in installing the l-digitoxose moiety (Fig. 3g and Supplementary Fig. 19). Furthermore, we found that SelSV can also catalyse reverse glycosylation with kasufungin B, but not with selvamicin (Fig. 3g and Extended Data Fig. 5). This suggests SelSV does not recognize 4-*O*-methyl-l-digitoxose and that selvamicin is glycosylated with l-digitoxose by SelSV, which is subsequently 4-*O*-methylated by the putative methyltransferase SelSI^23^. Initial attempts to crystalize KfuSV, and the related GTs, were unsuccessful. Instead, an AlphaFold model of KfuSV was generated showing a typical GT-B fold similar to that of related bacterial GTs such as YjiC^42^ (Supplementary Fig. 20). The structural model was used to guide active site mutagenesis, which revealed His13 is likely to hydrogen bond to the C35-OH, and may function as a general base. More active site residues (H294, T299 and D318) may also contribute to catalysis and/or substrate binding (Supplementary Fig. 20). KfuSV T297A also showed improved activity with non-native polyene substrates, suggesting that the substrate scope of KfuSV may be further expanded through enzyme engineering.

**Fig. 4:**
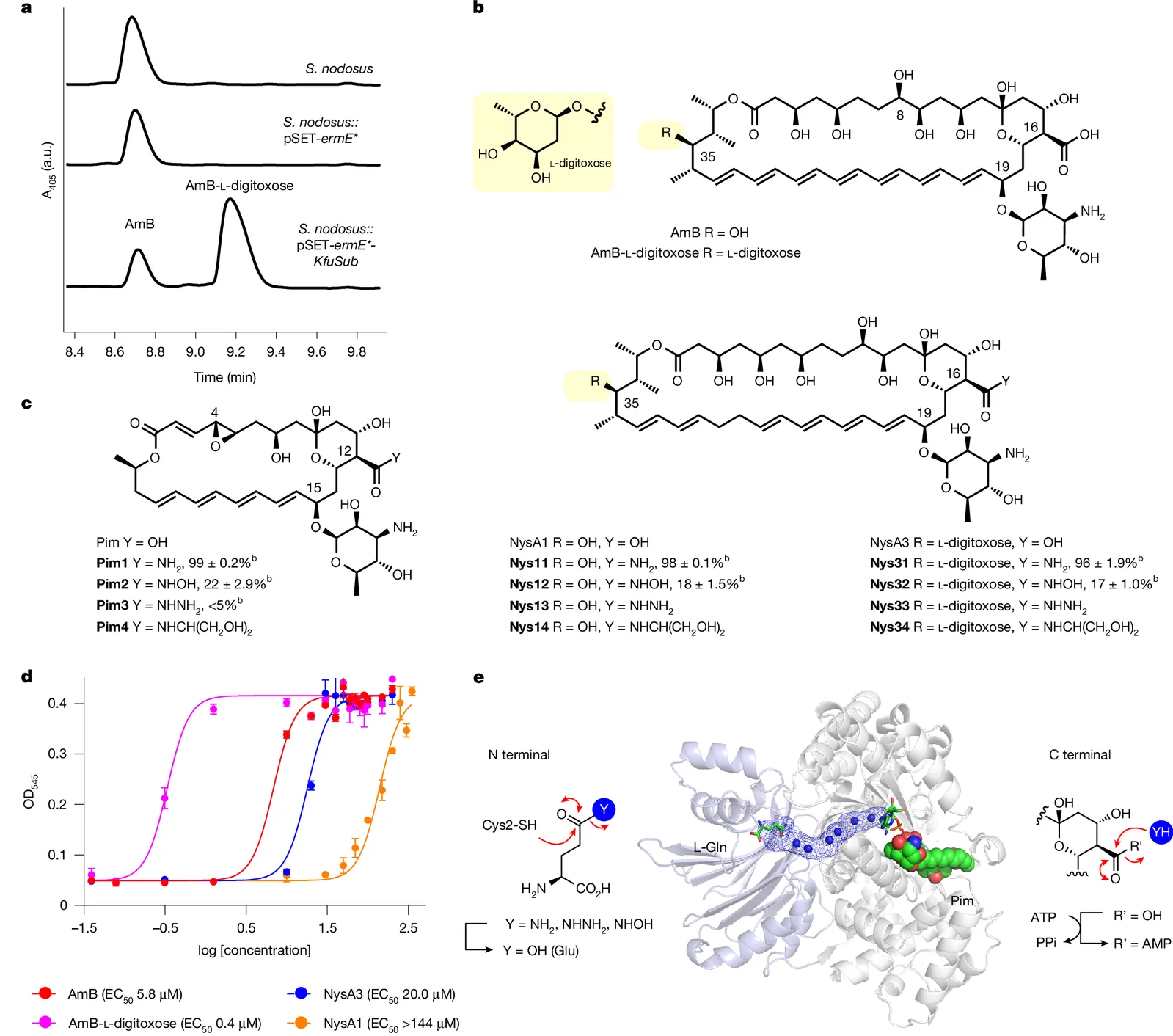
Amphotericin-deoxysugar derivative, bioactivity data and carboxylic acid derivatization. **a**, RP-HPLC analysis of a new glycosylated AmB derivative produced by expression of the *KfuSub* plasmid, possessing the KfuSV subcluster, in *S. nodosus* (compared with no production in *S. nodosus* alone or *S. nodosus* with an empty plasmid). HPLC absorption was measured at 405 nm, which is specific for the AmB chromophore. The AmB-l-digitoxose product was fully characterized by NMR (Supplementary Fig. 22). **b**, Structures of AmB and AmB-l-digitoxose. **c**, Structures of pimaricin with C12 modifications and nystatins with extra C16-carboxylate modifications. ^b^Percentage conversions for the PcsA-catalysed carboxylate-modification reactions. **d**, Horse blood haemolysis EC_50_ (µM) data for sugar modified (AmB-l-digitoxose and NysA3) and the parent polyenes (AmB and NysA1) were obtained from (*n* = 3) biological replicates and error bars represent ±standard deviation (s.d.). **e**, Schematic of the PcsA-catalysed carboxylic acid functionalization reaction, along with a structural model, generated by AlphaFold, highlighting (blue mesh) the tunnel between N and C termini (predicted using CAVER). a.u., arbitrary units. Source data

To further explore whether the flexible KfuSV could be used to derivatize other polyenes in vivo, the entire KfuSV subcluster was introduced into *S. nodosus* (AmB producer). Heterologous expression of this l-digitoxose subcluster led to the production of new AmB and AmA (the tetraene analogue of AmB) derivatives with masses consistent with the addition of a dideoxysugar (Fig. 4a and Supplementary Fig. 21). We isolated the most abundant AmB derivative from the fermentation of the engineered *S. nodosus* strain and subjected this to detailed NMR analysis (Supplementary Fig. 22). Our NMR data, when compared to the NMR data reported for AmB^43^, are fully consistent with the addition of l-digitoxose onto C35 (AmB-l-digitoxose, Fig. 4b). Heterologous expression of the corresponding *Sa*NysSV subcluster in *S. nodusus* also resulted in the same AmB-l-digitoxose derivative (Supplementary Fig. 23), demonstrating that the new GT enzymes can be deployed in vivo, as well as in vitro, to derivatize medically relevant polyenes. Such fermentation methods can ultimately lead to more cost-effective production of improved polyene antifungal agents.

## Enzymatic modifications improve bioactivity

**Extended Data Fig. 6:**
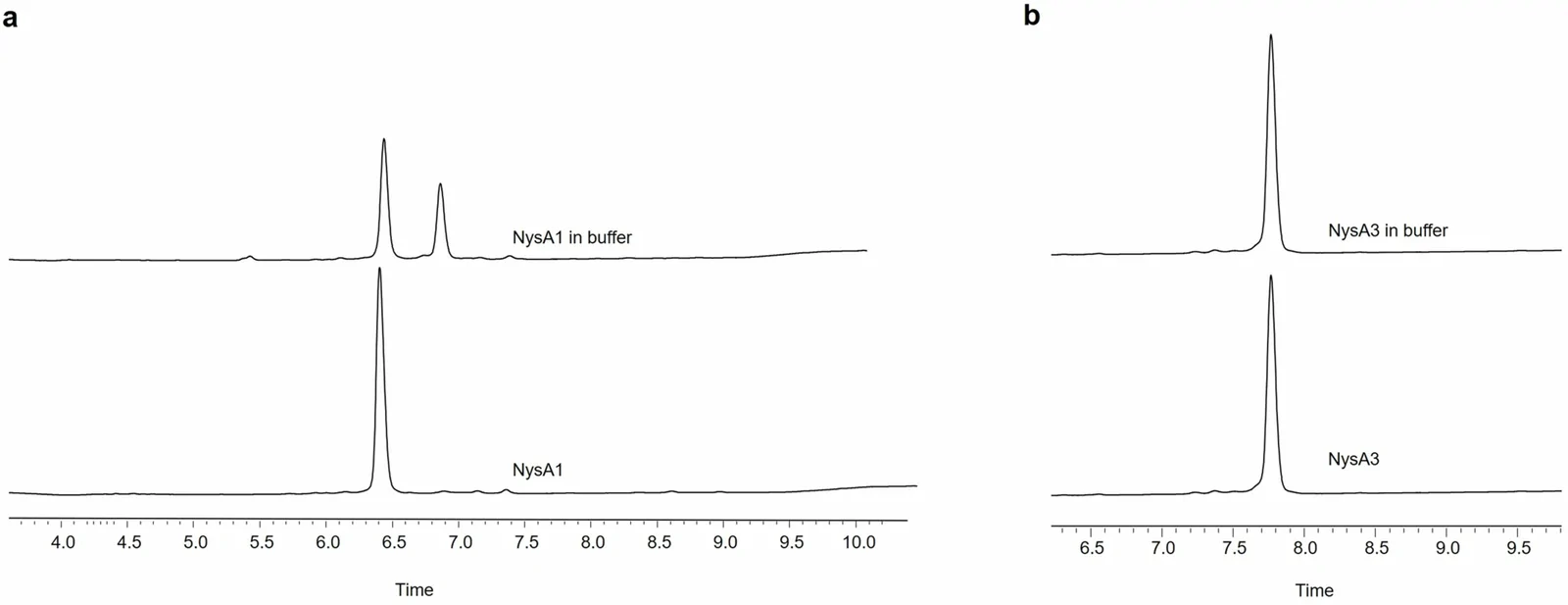
HPLC analysis of NysA1 and NysA3. (**a**) Shows isomerisation of NysA1 following incubation overnight at 30 °C in 50 mM Tris-HCl buffer (pH 7.0). NysA1 is suggested to exist in equilibrium between two conformational states due to the rotation of the C28-C29 single bond^46^. Alternative studies also suggest that the two isomers of NysA1 may arise due to trans lactonisation^47^. (**b**) Shows NysA3 incubated overnight at 30 °C in 50 mM Tris-HCl buffer (pH 7.0) is stable and does not isomerise.

Half-maximal inhibitory concentration (IC_50_) values were determined for AmB-l-digitoxose and NysA3 (Fig. 4b) with ten clinically relevant fungal pathogens defined as critical or high risk by the World Health Organization^44^, including drug-resistant species, such as the *Aspergillus fumigatus cyp51A*^TR34/L98H^ isolate^45^ (Table 1). The IC_50_ values indicate that the addition of l-digitoxose to AmB increases antifungal activity against eight of the ten pathogens tested, including *Candida albicans* ATCC 90028 (2.7-fold) and *Fusarium oxysporum* (2-fold). NysA3 is also more potent than the parent NysA1 with six of the pathogens tested, most notably with *Fusarium solani* (10.2-fold). AmB-l-digitoxose and NysA3 do, however, show increased haemolysis of red blood cells (half-maximum effective concentration (EC_50_)), which is used as an indicator of polyene toxicity^28^ (Fig. 4d). Although the origins of enhanced bioactivity of AmB-l-digitoxose and NysA3 are unclear, in the case of nystatin the C35 sugar seems to prevent isomerization. The parent NysA1 exists as a mixture (roughly 55:45) of bioactive and inactive isomers in buffer^46,47^, which is evident as two peaks on HPLC analysis (Fig. 3f). With NysA3, however, only a single peak is present in HPLC, suggesting the second C35 sugar shifts the equilibrium in favour of the bioactive isomer (Extended Data Fig. 6), which may contribute to the improved antifungal activity we observe.

**Table 1:** IC_50_ values for AmB, NysA1 and their derivatives against fungal pathogens

	IC_50_ (μg ml^−1^)							
**Strain**	**AmB**	**AmB-l-digitoxose**	**NysA1**	**NysA3**	**Nys31**	**Nys32**	**Nys33**	**Nys34**
*Aspergillus fumigatus* A1160	0.45	1.51	1.49	2.74	*0.49*	*0.71*	*0.57*	*0.70*
*Aspergillus fumigatus cyp51A*^TR34/L98H^	0.53	1.11	1.01	1.90	***0***.***39***	*0.57*	***0***.***21***	***0***.***50***
*Rhizopus delemar* 99-880	0.07	0.05	0.83	0.43	*0.79*	*0.58*	*0.78*	1.05
*Mucor circinelloides* 1006Phl	0.57	0.53	0.67	1.19	2.93	1.68	3.09	3.24
*Candida albicans* ATCC 90028	0.16	0.06	1.04	0.69	*0.74*	*0.74*	*0.65*	*0.92*
*Candida albicans* ATCC 200955	0.39	ND	15.74	2.64	*6.32*	*2.82*	*3.45*	*1.78*
*Candida glabrata* NCPF3309	0.54	0.30	0.59	0.50	*0.58*	***0***.***41***	*0.58*	0.71
*Candida auris* H.17.157	0.33	0.19	0.91	1.07	*0.44*	*0.46*	*0.69*	0.95
*Cryptococcus neoformans* F10025	0.05	0.001	0.90	0.78	*0.05*	*0.07*	*0.05*	*0.17*
*Fusarium oxysporum* 9935	1.18	0.56	4.35	2.56	***1***.***14***	***1***.***07***	*1.50*	*1.90*
*Fusarium solani* 9596	1.23	0.71	2.75	0.27	***0***.***88***	***0***.***88***	***1***.***01***	*1.75*

IC_50_ data with ten fungal pathogens shows NysA3 derivatives with better activity than NysA1 (shown in italics), and better than AmB (shown in bold).

Given that the C35 sugar improves both antifungal activity and solubility of clinically relevant polyenes, we sought to explore whether C16-carboxylate derivatization might mitigate the toxicity of NysA3 (for example, enhancing selectivity for the fungal versus mammalian cell membrane). Rather than using the costly synthetic procedures that were used previously to produce less toxic AmB variants^5,6,27^, we sought to develop more efficient and cleaner enzymatic methods to introduce C16 modifications. For example, an amidotransferase, PcsA, was shown to install a C12-primary amide moiety found in the smaller polyene rimocidin^48,49^. PcsA is predicted to possess an N-terminal glutamine hydrolase domain, releasing ammonia that is channelled through a molecular tunnel to a C-terminal domain, where it intercepts a polyene acyl-AMP intermediate (Fig. 4e). PcsA was shown to transform the C12–CO_2_H of rimocidin and related pimaricin (natamycin) to the amide (C12–CONH_2_) in vivo or using crude cell free extracts^49^. However, PcsA was reported not to accept larger polyenes, including nystatin, and attempts to purify the enzyme failed as the addition of affinity tags onto either terminus led to loss of function^49^. Notwithstanding this, we identified a loop region at which a His_6_-tag could be inserted, enabling the purification of PcsA. Contrary to the earlier studies^49^, we showed that PcsA can accept larger polyenes. Both NysA1 and NysA3 C16-amide derivatives (**Nys11** and **Nys31**) were produced with 98% and 96% conversion, respectively, compared with pimaricin (natamycin) that is very similar to the native substrate and gives near-complete conversion (**Pim1**) under similar assay conditions (Fig. 4c and Supplementary Figs. 24–26). PcsA also catalysed amidation of a range of other polyenes, including candicidin (used to treat yeast infections), CE-108, dihydromycoheptin and mandimycin (Supplementary Figs. 27–31). In addition to broad polyene scope, we also showed glutamine derivatives with modified side chains can transfer alternative nucleophiles (NH_2_OH and NH_2_NH_2_) through the tunnel to form various polyene hydroxamic acid derivatives (**Pim2**, **Nys12**, **Nys32**, rimocidin, CE-108, dihydromycoheptin, mandimycin and candicidin hydroxamates) and, in the case of pimaricin (natamycin), an acyl-hydrazine derivative was also observed in small quantities (**Pim3**) (Fig. 4c and Supplementary Figs. 24–31). NMR confirmed the structures of the polyene C16 derivatives (Supplementary Figs. 32–44).

**Fig. 5:**
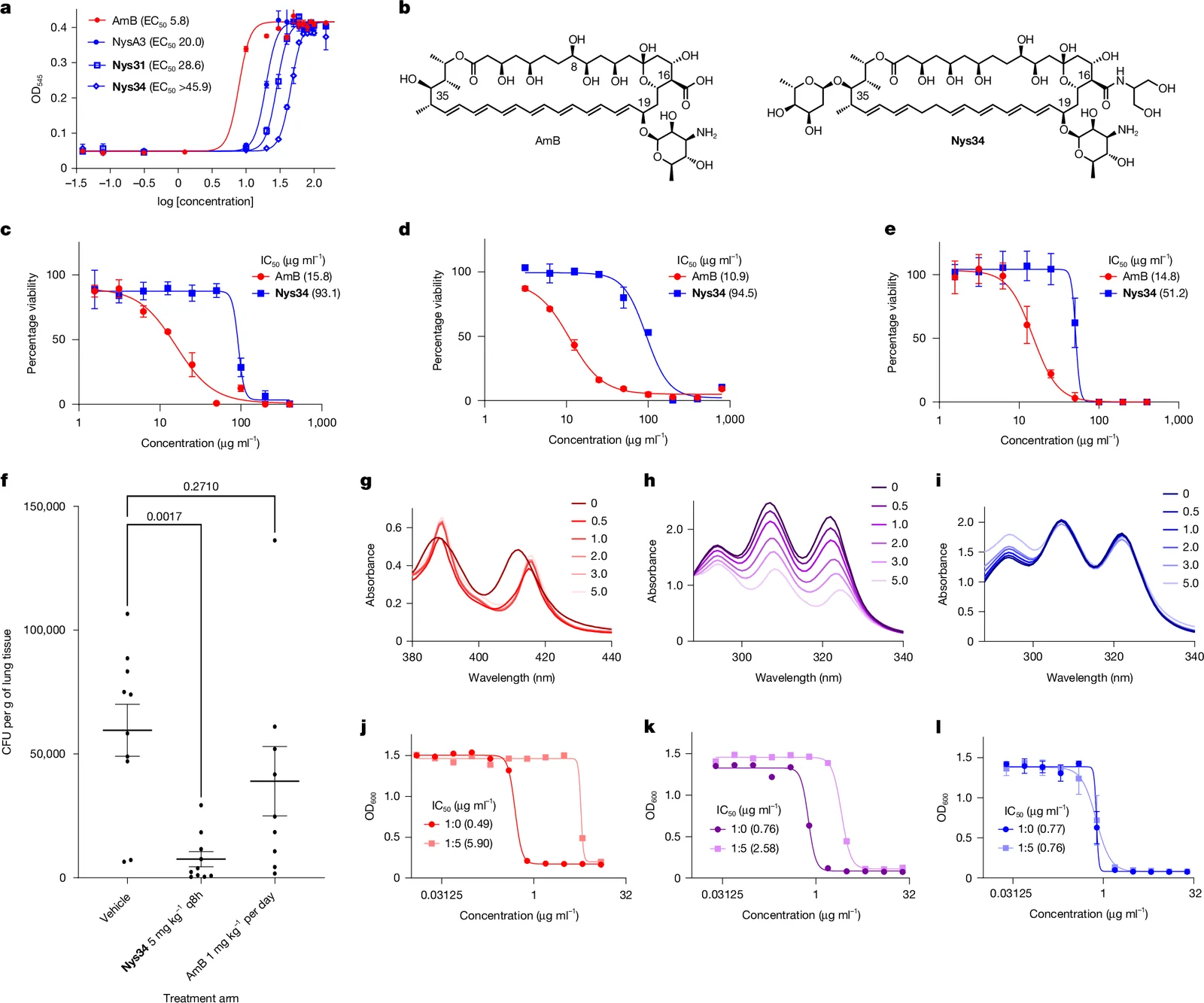
Comparing the bioactivity and toxicity of sugar and C16 modified polyenes with AmB and NysA1. **a**, Horse blood haemolysis EC_50_ (µM), used as an indicator of toxicity, comparing NysA3 and its C16 modified derivatives (**Nys31** and **Nys34**) to AmB. The experiments were performed in (*n* = 3) biological replicates and error bars represent ±s.d. **b**, The structures of AmB and **Nys34**. **c**–**e**, Dose–response curves of HEK293 (**c**), A549 (**d**) and HEPG2 (**e**) cells incubated with varying concentrations of AmB (red) and **Nys34** (blue). All cell lines were tested in (*n* = 3) biological replicates and error bars represent ±s.d. **f**, Treatment of *A. fumigatus* infection of immunosuppressed CD1 mice with **Nys34** at 5 mg kg^−1^ q8h ×4 and AmB at 1 mg kg^−1^ once a day ×2. Mice were immunosuppressed with triamcinolone before intranasal inoculation with 5 × 10^5^ CEA10 spores (5 male and 4 female in the AmB group; 5 male and 5 female in other groups). Treatment was initiated 8 h postinoculum and the mice were culled 32 h after the first treatment. Lungs were homogenized and plated on SDA. Following incubation for 48 h at 37 °C, CFUs were counted and normalized per gram of lung tissue. Each point represents data from an individual mouse. The **Nys34** treatment arm was significantly different from the vehicle (non-treatment) arm *P* = 0.0017 one-way analysis of variance with Dunnett’s post hoc test. Statistical tests were two-sided, with *α* = 0.05. Error bars show the mean with standard error of the mean. **g**–**i**, UV–Vis ergosterol titration binding studies of AmB (**g**), NysA1 (**h**) and **Nys34** (**i**) with increasing molar ratios of ergosterol (0–5 eq.). **j**–**l**, Dose–response curves of AmB (**j**), NysA1 (**k**) and **Nys34** (**l**) (dark shaded lines) with *S. cerevisiae*, compared with AmB, NysA1 and **Nys34** precomplexed with 5 molar equivalents of ergosterol (light shaded lines). For **j** and **k** measurements were taken in biological duplicates whereas **l** was taken in biological triplicates with error bars shown as ±s.d. Source data

**Extended Data Fig. 7:**
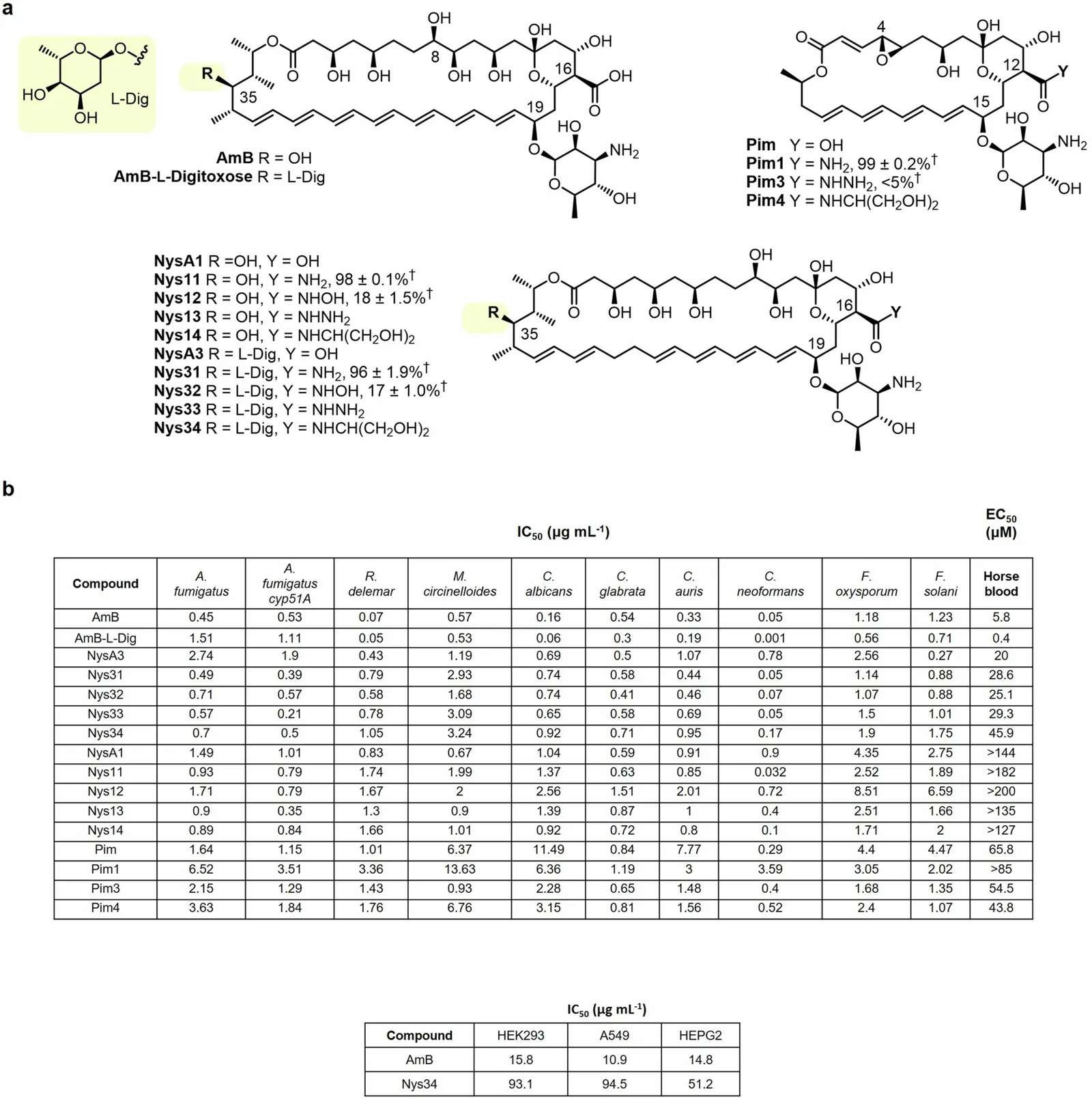
Biological activity data for polyene derivatives. (**a**) Structures of polyene derivatives tested, followed by table of IC_50_ values against *Aspergillus fumigatus A1160, Aspergillus fumigatus cyp51A*^*TR34/L98H*^, *Rhizopus delemar 99-880, Mucor circinelloides 1006Phl, Candida albicans ATCC 90028, Candida glabrata NCPF3309, Candida auris H.17.157, Cryptococcus neoformans* F10025*, Fusarium oxysporum 9935*, and *Fusarium solani 9596*. Also shown is horse blood haemolysis EC_50_ values. (**b**) Table of IC_50_ values for AmB and Nys34 against HEK293, A549, and HEPG2 cell lines.

**Extended Data Fig. 8:**
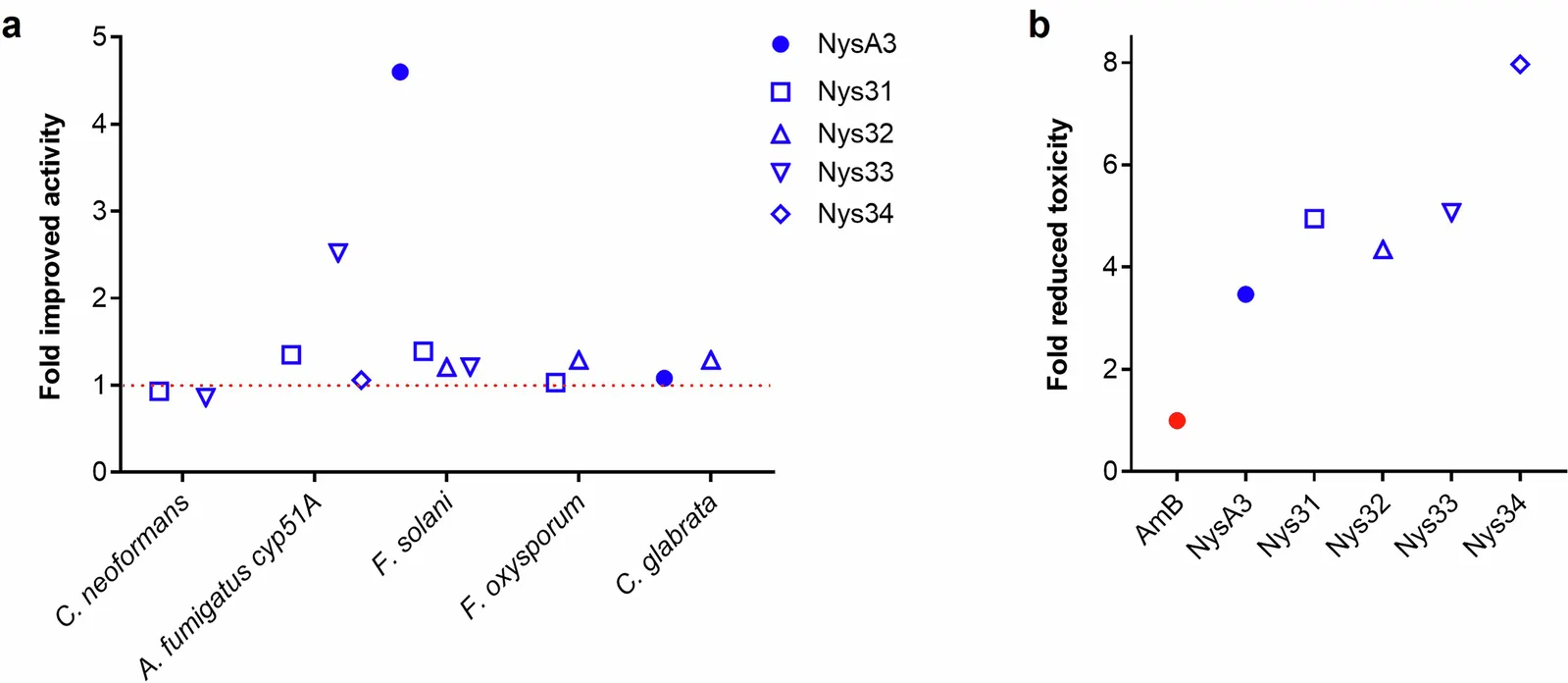
Comparing the bioactivity and toxicity of sugar and C16 modified polyenes with AmB. (**a**) The fold improvement in antifungal activity (IC_50_) of NysA3 and its derivatives (blue) compared to AmB (red line). (**b**) The fold reduced toxicity (horse blood haemolysis EC_50_) of NysA3 and its derivatives (blue) compared to AmB (red).

Finally, we evaluated the antifungal activity and toxicity of the new polyene variants (Table 1, Extended Data Figs. 7 and 8 and Supplementary Figs. 45–47). In addition to enzymatically generated polyene C16 derivatives, the NysA3 carboxylic acid was also chemically modified with serinol to generate a serinol amide derivative (**Nys34**). Although inaccessible with PcsA, the serinol amide modification was previously shown to be most effective for improving the characteristics of AmB^6^. The acyl-hydrazine derivative of NysA3 was also prepared for comparison of bioactivity data. Overall, we observe that nystatin variants combining C35 sugar and C16 modifications (**Nys31**, **Nys32**, **Nys33** and **Nys34**) show higher antifungal activity against most fungal pathogens tested than either parent molecule NysA1 or NysA3, and in several cases are more potent than AmB (the most effective clinically relevant polyene antifungal agent). Notably, Nys33 is 2.5-fold and 5-fold more potent than clinically relevant AmB and NysA1, respectively, when tested against azole-resistant *A. fumigatus cyp51A*^TR34/L98H^. The NysA3 derivatives also show twofold to ninefold better activity than NysA1 with the *Fusarium* species tested and the AmB-resistant *Candida albicans* ATCC 200955. Moreover, all the dual functionalized nystatin variants show reduced haemolysis (Fig. 5a) compared with both NysA3 and AmB, indicating these derivatives also have lower toxicity to mammalian cells.

## Nys34 reduces fungal burden in mice

**Extended Data Fig. 9:**
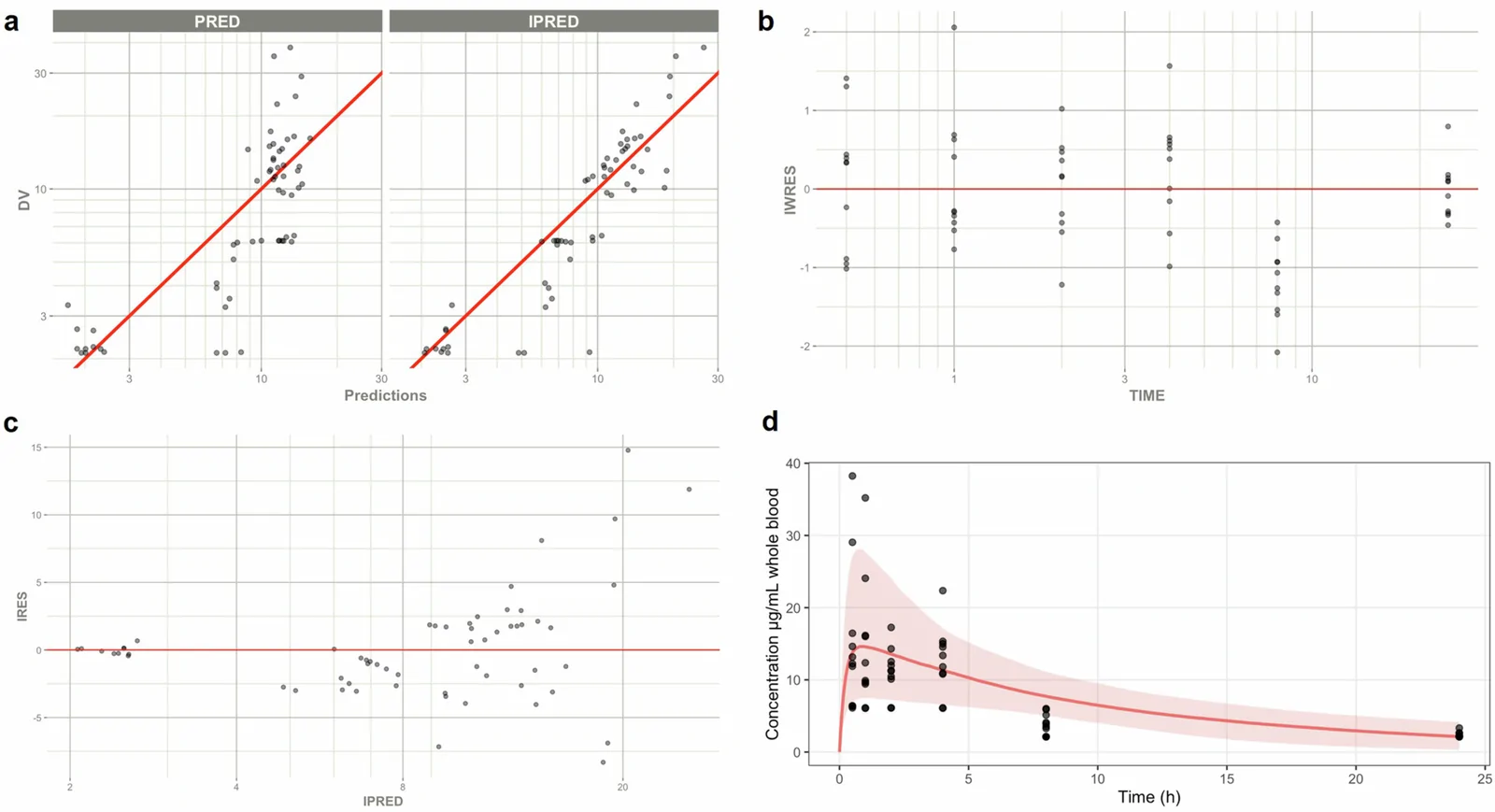
Goodness of fit diagnostics for the population pharmacokinetic (PK) model of Nys34 in mice. Observed concentrations were derived from 10 mice per timepoint at 0.5, 1, 2, 4, 8 and 24 hours dosed at 10 mg/kg IP. (**a**) Observed concentration of Nys34 (µg/mL) (DV) in whole blood plotted against the population predicted concentration (PRED) and individual predicted values (iPRED) derived from fitting a simple one-compartment population PK model to the PK dataset using nlmixr2. (**b**) Individual weighted residuals (IWRES) versus time for the population PK model. Each point represents the weighted residual for an observed concentration at the corresponding sampling time, and the horizontal line indicates zero residual. Residuals were centered around zero (Median IWRES -0.2, Mean -0.12) with no apparent time-dependent trend, supporting an adequate model fit. (**c**) Individual residuals (IRES) versus individual predictions (IPRED). Each point represents one observation, plotted as the difference between the observed concentration and the individual prediction (IRES = DV − IPRED). Again, data is centered around zero. (**d**) Visual predictive check for the population PK model. The observed concentration–time data are shown as points (n = 10), while the solid line and shaded region represent the median and 90% prediction interval (5th–95th percentiles) of simulated concentrations (n = 1000 simulations). The observed data were generally contained within the simulated prediction intervals, supporting model adequacy. Source data

One of the more promising derivatives, **Nys34** (Fig. 5b), was also shown to have between threefold and eightfold lower toxicity than AmB in human embryonic kidney 293 (HEK293) cells, A549 lung cells and human hepatoma (HEPG2) liver cells (Fig. 5c–e). In contrast to the parent polyene NysA1, which shows acute toxicity at low doses (4.4 mg kg^−1^) in mice^18^, **Nys34** was well tolerated following intraperitoneal (i.p.) injection of 3 repeat doses (10 mg kg^−1^, every 8 h (q8h)), however, mice showed signs of toxicity following a fourth dose. Population pharmacokinetics studies were carried out at a single dose of 10 mg kg^−1^ i.p., revealing concentration–time profiles consistent with a one-compartment model with first-order absorption and elimination (Extended Data Fig. 9). The estimated absorption rate constant (*k*_a_) and elimination rate constant (*k*_el_) were *k*_a_ = 4.82 h^−1^ and *k*_el_ = 0.082 h^−1^, corresponding to half-lives of roughly 9 minutes and 8.5 hours, respectively. Distribution of **Nys34** to all main organs tested, including the lungs, was observed. Using a population pharmacokinetics model, we defined a toxic exposure level on the basis of our observed toxic regimen (10 mg kg^−1^, q8h ×4). Candidate regimens were evaluated by Monte Carlo simulation with 5 mg kg^−1^ q8h ×4 resulting in <0.5% probability of exceeding toxic *C*_max_ or area under the curve thresholds. Using this dosing regimen in a mouse invasive aspergillosis model, **Nys34** reduced *A. fumigatus* counts (colony forming units, CFUs) in lungs by 87%, achieving statistical significance from the vehicle (*P* = 0.0017, Fig. 5f). Furthermore, no overt signs of toxicity were observed. Treatment with AmB at 1 mg kg^−1^ per day, which was previously shown to be effective at improving survival in a murine model, reduced *A. fumigatus* tissue burden by 35% after 2 days, but this change did not reach statistical significance^50^ (Fig. 5f).

The promising in vitro and in vivo activity of **Nys34** prompted an investigation into its mechanism of action. Unlike AmB and NysA1 (refs. ^6,19^), UV–Vis titration experiments show no evidence of **Nys34** binding to ergosterol (Fig. 5g–i). Furthermore, precomplexation^19^ of **Nys34** and ergosterol (1:5) had no effect on antifungal activity, whereas NysA1 and AmB showed considerable 3.4-fold and 11.8-fold reductions in IC_50_ with yeast *Saccharomyces cerevisiae* (Fig. 5j–l), and a similar trend was observed for *A. fumigatus* IC_50_ values with precomplexation (Supplementary Fig. 48). This indicates that **Nys34** has a different mechanism of action from the extramembranous sponge formation that is proposed to account for the activity of the parent polyenes^4,6,19^. Taken together, the results presented here indicate that the nystatin variants, especially **Nys34**, show improved properties over the parent polyenes used in the clinic, and have substantial potential for further clinical development.

## Conclusion

A few polyenes, such as selvamicin, mandimycin and NysA3, have previously been isolated with an extra sugar moiety, but the GTs responsible for these modifications had not been characterized. Through genome mining, we identified and characterized a family of GTs capable of efficiently installing a variety of second sugars onto clinically relevant polyenes. Several of these GTs were located within previously undescribed polyene biosynthetic pathways, which guided the isolation and characterization of a new family of kasufungin natural products. We also expanded the substrate and reaction scope of the amidotransferase PcsA, demonstrating that this enzyme can accept larger polyenes and glutamine derivatives. This enables the introduction of amide, hydroxamate and acyl-hydrazine functionality in place of the detrimental carboxylic acid group present in manypolyenes.

Most previous efforts to improve polyene therapeutics have relied on chemical synthesis^5,6,20,27^. However, the structural complexity of polyenes makes regioselective derivatization challenging, often necessitating extensive protecting-group strategies and multistep synthetic routes. Such approaches are costly and can restrict access to improved analogues and treatments. Earlier attempts to enzymatically diversify polyene scaffolds were met with limited success. For example, the GTs responsible for installation of the mycosamine sugar present in most polyenes are highly substrate selective and therefore poorly suited to glycodiversification^51^. By contrast, the GTs described here, particularly KfuSV, showed high substrate promiscuity, accepting a broad range of sugar donors and polyene acceptors.

The efficient and selective enzymatic installation of a second sugar, together with carboxylate modification, improves antifungal activity, reduces toxicity and increases the solubility of polyenes relative to widely used parent natural products, including several World Health Organization essential medicines. In a mouse infection model, **Nys34** emerged as a promising lead compound, showing in vivo efficacy through reduction of fungal burden in invasive aspergillosis without substantive signs of toxicity. Notably, the two structural modifications present in **Nys34** substantially altered its mechanism of action, eliminating ergosterol binding. Although further studies will be required to define this alternative mechanism, these findings demonstrate that targeted regioselective derivatization can fundamentally reshape the biological properties of clinically important polyene scaffolds. In addition to **Nys34**, several of the other polyene derivatives generated enzymatically in this study (for example, **Nys31**) show equally promising bioactivity and may also prove valuable for combating other fungal infections.

Taken together, the previously undescribed polyenes, enzymes, biosynthetic pathways and methodologies reported here provide a powerful platform for optimizing bioactivity and modulating the mechanism of action of polyene therapeutics. The enzymes and pathways described here are also amenable to engineering, which could enable further structural diversification and fine-tuning of polyene bioactivity. Directed evolution and strain engineering could also assist the development of scalable bioprocesses, making improved polyene therapeutics more widely available.

## Methods

### In vitro GT glycosylation assays

For GT assays with TDP glucose; GT enzyme (5–20 µM), TDP-glucose (2 mM), MgCl_2_ (1 mM) and polyene substrates, including amphotericin B (AmB), AmB-aglycone, nystatin A1 (NysA1) and mycoheptin and dihydromycoheptin, at 100 µM concentration were incubated in a total reaction volume of 50 µl in Tris-HCl buffer (50 mM, pH 7.4) at 30 °C for 24 h. For TDP sugars other than TDP glucose with available sugar-1-phosphates, an extra step was required in which the TDP sugar was produced from the corresponding sugar-1-phosphate. In this first step, deoxythymidine triphosphate (2 mM), sugar-1-phosphate (2 mM), MgCl_2_ (2.2 mM) and Cps2L (50 µM) in a total volume of 50 µl in Tris-HCl buffer (50 mM Tris-HCl, pH 7.4) were incubated for 24 h at 37 °C in a shaking thermomixer. After 24 h, the temperature was adjusted to 30 °C and GT (5–20 µM) and polyene (100 µM) were added. Assays were then incubated for a further 24 h at 30 °C. For TDP sugars with no available sugar-1-phosphate, TDP sugars were produced from the corresponding sugar. In this modified first step, deoxythymidine triphosphate (2 mM), ATP (2 mM), sugar (2 mM), MgCl_2_ (2.2 mM), NahK_ATCC15697 or GalkSpe4 (50 µM) and Cps2L (50 µM) in a total volume of 50 µl in Tris-HCl buffer (50 mM Tris-HCl, pH 7.4) was incubated for 24 h at 37 °C in a shaking thermomixer. After 24 h, the temperature was adjusted to 30 °C and GT (5–20 µM) and polyene (100 µM) were added. Assays were then incubated for a further 24 h at 30 °C.

For all assays (1, 2 or 3 steps), reactions were quenched by heating to 95 °C for 5 min, followed by the addition of 1 reaction volume of methanol. The protein was then pelleted by centrifugation, and the supernatant was analysed by analytical RP-HPLC and LC–HRMS. Assays were analysed on a Shimadzu Analytical ultrahigh-performance liquid chromatography (UHPLC) apparatus with a Kinetex 5 μm XB-C18 100 × 4.6 mm (Phenomenex) column, with a flow rate of 1 ml min^−1^, using a solvent system of water and methanol with 0.1% formic acid and a gradient of 60–78% methanol over 12 min. Heptaenes were monitored at 405 nm and pentaenes at 350 nm. LC–HRMS was performed on an Agilent 1290 Infinity II coupled to a 6560 Ion Mobility quadrupole time of flight (Q-TOF) LC–MS apparatus and 1290 Infinity II HPLC coupled to a 6546 LC–Q-TOF (Agilent Instruments), using a Luna Omega 5 μm 100 × 2.1 mm (Phenomenex) column, with a flow rate of 0.5 ml min^−1^, using a solvent system of water and methanol and a gradient of 60–95% methanol over 15 min. On LC chromatograms, tetraenes were monitored at 304 nm, pentaenes at 350 nm and heptaenes at 405 nm.

### GT-catalysed reverse glycosylation of polyenes

Each of the polyenes (25–100 μM, kasufungin B, NysA3, semipurified selvamicin) were incubated with TDP (2 mM), MgCl_2_ (1 mM), GT (KfuSV, NysSV, SelSV, 25 μM) in 50 mM Tris-HCl buffer pH 7.5–8, 30 °C overnight, in a 50 μl reaction. The reactions were quenched with methanol (50 μl) and centrifuged (10,000 rpm, 5 min). The supernatant was analysed by HPLC and masses were confirmed by LC–MS using the same conditions as mentioned above in the in vitro GT glycosylation assays section.

### MycS3 assay with dihydromycoheptin and TDP-l-digitoxose

Reverse glycosylation of NysA3 was performed to generate TDP-l-digitoxose. A 200–300 μM solution of NysA3 was incubated with TDP (2 mM), MgCl_2_ (1 mM) and KfuSV (50 μM) in 50 mM Tris-HCl buffer pH 7.5–8, at 30 °C overnight, in a 2-ml reaction. The reactions were quenched with an equal volume of methanol to inactivate enzyme and centrifuged (10,000 rpm, 5 min). The supernatant was concentrated under vacuum to reduce the volume to one-quarter, applied to silica filled in a small syringe (silica C18 100 Å, 30 μm, silica bed 4 ml) and washed twice with an equal volume of H_2_O to elute TDP-l-digitoxose. The filtrate volume was reduced to one-fifth under vacuum and used for MycS3 assays.

For assays with MycS3, a solution of dihydromycoheptin (50 µM) in MycS3 reaction buffer (1 mM MgCl_2_, 50 mM Tris-HCl, pH 7.5) was incubated with the TDP-l-digitoxose containing concentrated filtrate (25 μl) and MycS3 (25 μM) at 30 °C overnight in 50-μl reactions. The reactions were quenched with methanol (50 μl) and centrifuged (10,000 rpm, 5 min) before the supernatant was analysed by HPLC and LC–MS. In separate experiments, dried *S. netropsis* (DSM40846) methanolic extract containing crude dihydromycoheptin, was resuspended in MycS3 reaction buffer (1 mM MgCl_*2*_, 50 mM Tris-HCl, pH 7.5) was incubated with the TDP-l-digitoxose containing concentrated filtrate (25 μl) and MycS3 (25 μM) at 30 °C overnight in 50-μl reactions. The reactions were quenched with methanol (50 μl) and centrifuged (10,000 rpm, 5 min) before the supernatant was analysed by LC–HRMS. HPLC analysis was performed on a Shimadzu Analytical UHPLC with a Kinetex 5 μm XB-C18 100 × 4.6 mm (Phenomenex) column, with a flow rate of 1 ml min^−1^, using a solvent system of water and methanol (+0.1% formic acid) and a gradient of 60–78% methanol over 12 min, with UV monitored at 350 nm. LC–HRMS was performed on an Agilent 1290 Infinity II coupled to a 6560 Ion Mobility Q-TOF LC–MS and 1290 Infinity II HPLC coupled to a 6546 LC/Q-TOF (Agilent Instruments), using a Luna Omega 5 μm 100–2.1 mm (Phenomenex) column, with a flow rate of 0.5 ml min^−1^, using a solvent system of water and methanol and a gradient of 60–95% methanol over 15 min.

### In vitro PcsA reaction conditions

Enzyme activity assays were carried out in a reaction containing 100 µM polyene (pimaricin, rimocidin, CE-108, NysA1, NysA3, mycoheptin, dihydromycoheptin, kasufungin B or candicidin) and l-glutamine, l-glutamic acid γ-hydroxamate or l-glutamic acid γ-hydrazide (2 mM), ATP (4 mM), MgCl_2_ (10 mM) and 25 µl of eluted protein in Tris buffer (125 mM Tris and 25 mM NaCl, pH 7) in a total volume of 50 µl. In addition, l-glutamine analogues (l-glutamic acid γ-methylamide, l-glutamic acid γ-ethylamide (l-theanine), l-glutamic acid γ-methyl ester and l-glutamic acid γ-ethyl ester) were also tested but no activity was observed. The reactions were quenched with methanol (50 µl) after overnight incubation at 30 °C, and centrifuged (10,000 rpm for 5 min). Assays were analysed on a Shimadzu Analytical UHPLC with a Kinetex 5 μm XB-C18 100 × 4.6 mm (Phenomenex) column, using 1 ml min^−1^ flow rate with a solvent system of water and methanol each containing 0.1% formic acid, using a gradient of 60–78% methanol over 12 min. LC–HRMS was performed on an Agilent 1290 Infinity II coupled to a 6560 Ion Mobility Q-TOF LC–MS instrument, using a Luna Omega 5 μm 100 × 2.1 mm (Phenomenex) column, using a 0.5 ml min^−1^ flow rate with a solvent system of water and methanol each containing 0.1% formic acid, and a gradient of 60–80% methanol over 15 min.

### Expression of *S. albulus nysSV-subcluster* in *S. albus*

The sugar subcluster from *S. albulus* (DSM40492), *Sa-nysSV-sub*, was amplified from the genomic DNA as two fragments; fragment 1 amplifying *nysSVI*, *nysSII*, *nysSV*, partial *nysSVII* and fragment 2 amplifying partial *nysSVII*,* nysSIII* and* nysSIV* using primer pairs (Supplementary Table 3). The plasmid pSET152-ermE* was linearized with *NdeI* and *EcoRI*, and the two fragments were assembled using HiFi assembly to form the construct pSET-ermE*-*Sa-nysSV-sub*. The construct was introduced into *S. albus* (J1074, a gift from M. Bibb at the John Innes Centre) by *E. coli* (ET12567) mediated conjugal transfer using standard conjugation protocols^52^, resulting in the *S. albus::Sa-nysSV-sub* strain. The empty plasmid pSET-ermE* was also introduced by conjugation resulting in S. albus::pSET-ermE* strain to be used as the negative control.

For NysA1 assays with the lysate, both *S. albus::Sa-nysSV-sub* and S. albus::pSET-ermE* strains were grown in tryptone soy broth (TSB) for 3 days. The resulting seed cultures (2% v/v) were used to inoculate 10 ml of SG2 growth medium (glucose 20 g l^−1^, yeast extract 5 g l^−1^ and soytone 10 g l^−1^, pH 7.2). After 48 h, 1 ml of each culture was centrifuged at 12,000 rpm for 10 min. The cell pellet was resuspended in lysis buffer at four times the pellet weight (50 mM Tris Cl, 150 mM NaCl, 10% glycerol, 2 mM MgCl_2_, pH 7.5), followed by addition of 4–6 mg ml^−1^ of lysozyme. The resulting cell suspension was incubated at 30 °C for 30 min, vortexed and centrifuged at 12,000 rpm for 20 min. A 100-µl portion of the supernatant was incubated with 100 µM of NysA1 at 30 °C overnight. The reactions were quenched with equal volumes of methanol and centrifuged (10,000 rpm for 10 min). Assays were analysed on a Shimadzu Analytical UHPLC with a Kinetex 5-μm XB-C18 100 × 4.6 mm (Phenomenex) column, using 1 ml min^−1^ flow rate with a solvent system of water and methanol each containing 0.1% formic acid, using a gradient of 60–78% methanol over 12 min. On LC, the conversion of NysA1 to NysA3 was monitored at 304 nm. LC–HRMS was performed on an Agilent 6546 LC/Q-TOF and 1290 Infinity II HPLC instrument, using a Luna Omega 5 μm 100 × 2.1 mm (Phenomenex) column, using a 0.5 ml min^−1^ flow rate with a solvent system of water and methanol each containing 0.1% formic acid, and a gradient of 60–80% methanol over 15 min.

### Expression of *kfuSV-* and *Sa-nysSV-subclusters* in *S. nodosus*

The sugar subcluster from *S. kasugaensis* (DSM40819), *kfuSV-sub*, was amplified from *S. kasugaensis* genomic DNA as three fragments; fragment 1 amplifying *kfuSVI*, *kfuSII*, fragment 2 amplifying *kfuSIII*, *kfuSIV*, *kfuSV* and fragment 3 amplifying *kfuSVII* using three primer pairs (Supplementary Table 3). The plasmid pSET152-ermE* was linearized with *NdeI* and *EcoRI*, and the three fragments were assembled using HiFi assembly to form the construct pSET-ermE*-*kfuSV-sub*. Similarly, the sugar subcluster from *S. albulus* (DSM40492)*, Sa-nysSV-sub* was amplified from the genomic DNA as two fragments to form the construct pSET-ermE*-*SanysSV-sub* using HiFi, as described above. The two constructs pSET-ermE*-*kfuSV-sub* and pSET-ermE*-*SanysSV-sub* were introduced separately into *S. nodosus* (DSM 40109) by standard conjugation protocols, resulting in the *S. nodosus::kfuSV-sub* and *S. nodosus::Sa-nysSV-sub* strains, respectively^52^. The empty plasmid pSET-ermE* was also introduced into *S. nodosus* by conjugation resulting in the S. nodosus::pSET-ermE* strain to be used as the negative control. For polyene production, both strains were grown in TSB for 3 days. 2% v/v of the seed culture was inoculated into 50 ml of FSM (fructose 20 g l^−1^, dextrin 60 g l^−1^, soya flour 30 g l^−1^, CaCO_3_ 10 g l^−1^) growth medium. Fermentation cultures were supplemented with amberlite XAD16N (50 g l^−1^) resin and were incubated for 7 days with shaking at 30 °C. Mycelia and amberlite XAD16N resin were pelleted by centrifugation and polyenes were extracted with methanol (1× culture volume). A second round of methanol extraction could be performed to improve polyene recovery.

### *S. albulus* and *S. noursei* transcriptome analysis (RT–qPCR)

The expression levels of *nysSII*, *nysSIII* and *nysSVII* were examined using a two-step RT–qPCR system. Starter cultures of *S. noursei* (ATCC 11455) and *S. albulus* (DSM40492) were grown at 28 °C for 48 h in TSB media before 2% (v/v) of starter culture was used to inoculate SPG fermentation media. After 48 h of incubation at 28 °C, 0.5 ml of culture was harvested, washed with phosphate-buffered saline (PBS) and total RNA was extracted using the Monarch Spin RNA Isolation Kit (NEB) as per the manufacturers’ instruction, with an on-column DNase I treatment to remove any contaminating DNA. The quantity of RNA was determined using Nanodrop (ThermoScientific) and the integrity of the purified samples was determined using a 4150 TapeStationAnalyser (Agilent). Then 1 μg of purified RNA was immediately used as a template for complementary DNA (cDNA) synthesis using the SuperScript IV VILO Master Mix (ThermoFisher) following the manufacturers’ instructions. cDNA was flash frozen and stored at −80 °C until use. Primers were designed using the PrimerQuest Tool (IDT) to amplify 80–150 base-pair fragments of the genes of interest and are listed in Supplementary Table 3. qPCR was performed using PowerTrack SYBR Green Master Mix (ThermoFisher) and a StratageneMx3000P system. Amplification conditions used were 95 °C for 2 min and then 40 cycles of 95 °C for 15 s, 60 °C for 60 s, followed by a single default dissociation cycle. Next, 16S ribosomal RNA was used as an internal control. The absence of contamination was verified with no-template and no-reverse-transcription controls. Three biological replicates were performed for each gene. For statistical analysis, multiple (three) unpaired *t*-tests were performed, specifying an *α* value of 0.05.

### Production of polyenes from *S. albulus* and *S. noursei*

*S. albulus* (DSM40492) or *S. noursei* (ATCC 11455) spores were used to inoculate GYM media and the seed cultures were cultivated for 3 days at 30 °C. For fermentation, the seed cultures were used to inoculate FSM media (2% v/v inoculum, 3 × 50 ml, triplicates). Fermentation cultures were supplemented with amberlite XAD16N resin (50 g l^−1^) and were incubated for 7 days with shaking at 30 °C. Mycelia and amberlite XAD16N resin were pelleted by centrifugation. Polyenes were extracted with methanol (1 culture volume) overnight at 4 °C, protected from light. The resulting polyene extracts were diluted fivefold and 10 µl was injected for analysis by analytical HPLC. The relative titres of NysA1 and NysA3 were calculated by comparison with NysA1 standard calibration curve.

### Production of polyenes from *S. netropsis*

*S. netropsis* (DSM40846) spores were used to inoculate TSB media and the seed cultures were cultivated for two days at 30 °C. For fermentation, the seed cultures were used to inoculate 200 ml of FSM (fructose 20 g l^−1^, dextrin 60 g l^−1^, soya flour 30 g l^−1^, CaCO_3_ 10 g l^−1^) fermentation cultures in 1 l flasks (2% v/v inoculum). Fermentation cultures were supplemented with amberlite XAD16N (50 g l^−1^) resin to aid natural product recovery and were incubated for 7 days with shaking at 30 °C. Mycelia and amberlite XAD16N resin were pelleted by centrifugation. Polyenes were extracted with methanol (1× 1 culture volume). LC–HRMS was performed as described above.

### Production of polyenes from *S. kasugaensis*

*S. kasugaensis* (DSM40819) spores were used to inoculate TSB and the seed cultures were cultivated for 2 days at 30 °C. For fermentation, the seed cultures were used to inoculate 200 ml of FSM (fructose 20 g l^−1^, dextrin 60 g l^−1^, soya flour 30 g l^−1^, CaCO_3_ 10 g l^−1^) fermentation cultures in 1-l flasks (2% v/v inoculum). Fermentation cultures were supplemented with amberlite XAD16N (50 g l^−1^) resin and were incubated for 7 days with shaking at 30 °C. Mycelia and amberlite XAD16N resin were pelleted by centrifugation. Polyenes were extracted with methanol (2× 1 pellet volume).

### Purification of polyenes and NMR structural characterization

Extracts from *S. nodosus::kfuSV-sub*,* S. nodosus::Sa-nysSV-sub*,* S. albulus*, *S. netropsis* (DSM40846) and *S. kasugaensis* were dried in vacuo to complete or near-complete dryness. Water (50 ml) was added to the dry material, the resulting suspension was incubated at 4 °C for 3 h and then centrifuged to produce pellets containing polyenes. All polyene pellets were then dissolved in dimethylsulfoxide (DMSO) for purification by semi-preparative RP-HPLC (Shimadzu Prominence HPLC with a Phenomenex Gemini C18 column 250 × 10 mm, 5-μm particle size). Before HPLC kasufungin B was partially purified using a Mega BE-C18, 10 g, 60-ml bond elute column (Agilent Technologies). The bond elute was washed with up to 70% methanol to remove most impurities, followed by 100% methanol to elute the pentaene compounds. The eluant was concentrated in vacuo and further purified with semi-preparative HPLC. Mycoheptin, dihydromycoheptin and mandimycin were purified from *S. netropsis* (DSM40846) extract using a 5 ml min^−1^ flow rate with a solvent system of water and methanol at an isocratic flow of 65% methanol (MeOH) for 25 min, followed by 95% MeOH for 12.5 min. Kasufungin B from *S. kasugaensis* was purified using 5 ml min^−1^ flow rate with a solvent system of water and methanol using a 65% isocratic flow of methanol over 11 min followed by a gradient of 66–84% methanol over 12.5 min. AmB-l-digitoxose from *S. nodosus::kfuSV-sub* was purified using 5 ml min^−1^ flow rate with a solvent system of water and methanol with a gradient of 70–88% methanol over 30 min. NysA3 from *S. albulus* was purified using methanol gradients (40–65% for the first 5 min and 65–85% over the next 18 min). Fractions containing the appropriate polyenes were dried in vacuo and dissolved in DMSO-D_6_ (NysA3 and AmB-l-digitoxose) or CD_3_OD (kasufungin B) for NMR analysis. One-dimensional (^1^H, ^13^C) and two-dimensional NMR (correlation spectroscopy, heteronuclear single quantum coherence, heteronuclear multiple-bond correlation spectroscopy and rotating-frame nuclear Overhauser effect spectroscopy) experiments were performed at 500-MHz frequency to confirm the structures of mycoheptin, dihydromycoheptin and mandimycin, and 800 MHz for NysA3, kasufungin B and AmB-l-digitoxose.

### Antifungal activity tests (IC_50_ determination)

Antifungal susceptibility testing was performed according to the European Committee for Antimicrobial Susceptibility Testing reference microdilution method (version 9.3.2)^53^ and (v.7.4)^54^. Yeasts (*C. albican*s ATCC 90028, *C. albican*s ATCC 200955, *C. glabrata* NCPF3309, *C. auris* H.17.157 and *Cryptococcus neoformans* F10025) were cultured on Sabouraud’s dextrose agar (SDA) plates at 30 °C for 3 days. Inocula were prepared by suspending five distinct colonies in PBS-0.1% Tween and adjusted to 5 × 10^5^ cells per ml in sterile distilled water. Filamentous fungi (*Aspergillus fumigatus* A1160, *Aspergillus fumigatus cyp51A*^TR34/L98H^, *Rhizopus delemar* 99-880, *Mucor circinelloides* 1006Phl, *Fusarium oxysporum* 9935 and *Fusarium solani* 9596) were cultured in SDA T25 vented flasks at 37 °C for 3 days, except *Fusarium* species that were cultured at 28 °C for 5 days. Conidia were collected with PBS-0.1% Tween and filtered through miracloth. Inocula were adjusted to 5 × 10^5^ cells per ml in sterile distilled water. Tests were performed in flat-bottom 96-well plates (CytoOne, StarLab) containing 100 μl of 2× Roswell Park Memorial Institute-1640 medium of a twofold dilution series of antifungal agents and a drug-free control well. Compound stock solutions were prepared in DMSO and concentrations tested were 12.5–0.012 μg ml^−1^ for the AmB series and 50–0.048 μg ml^−1^ for the Pim, NysA1 and NysA3 series. Each well was inoculated with 100 μl of the respective strain at a final concentration of 5 × 10^4^ conidia. In addition, Milli-Q water (100 μl) was added to a row of wells in each plate as a sterility control. All plates were incubated at 37 °C for 48 h and optical density at 600 nm (OD_600_) was measured using a BioTek Synergy 2 SL microplate reader. From the optical density measurement, the IC_50_ of each drug was determined by fitting inhibitory dose–response curves using variable slope model on GraphPad Prism, version 10.2.3 (347).

### Horse blood haemolysis

Measurement of polyene haemolytic activity on horse blood was performed with slight modifications to the method reported in ref. ^28^. Polyene dilutions ranging from 0.04 µM to 350 µM were prepared from 4 mM stock in DMSO. From each of the dilutions, 6 µl was added to 114 µl of PBS buffer containing 2.5% horse blood (ThermoFisher) and incubated at 37 °C for 1 h in 96-well plates, centrifuged (4,000 rpm for 15 min) and OD_545_ of the supernatant (100 µl) was measured using a plate reader. Compounds were added to randomly assigned rows in a 96-well plate. A total of 16 different final concentrations (0.04 µM, 0.08 µM, 0.3 µM, 1.25 µM, 10 µM, 20 µM, 30 µM, 40 µM, 50 µM, 60 µM, 70 µM, 80 µM, 90 µM, 100 µM, 150 µM, 200 µM) were tested for AmB, AmB-l-digitoxose, NysA3, **Nys31**, **Nys32**, **Nys33** and **Nys34**, 15 final concentrations (0.6 µM, 1.3 µM, 2.5 µM, 10 µM, 30 µM, 50 µM, 70 µM, 100 µM, 120 µM, 140 µM, 160 µM, 200 µM, 250 µM, 300 µM, 350 µM) for NysA1, **Nys11**, **Nys12**, **Nys13** and **Nys14**, 13 final concentrations (10 µM, 30 µM, 50 µM, 70 µM and 90 µM, and between 110 µM and 350 µM) for Pim, **Pim1**, **Pim2**, **Pim3** and **Pim4**. Positive and negative controls were determined by adding 6 µl of DMSO into 114 µl of 2.5% horse blood in distilled water and PBS buffer, respectively, incubated at 37 °C for 1 h and OD_545_ was determined as described above. All modified polyenes and standards were tested in parallel with controls in triplicates. The values for 50% horse blood haemolysis (EC_50_) were determined by a four-parameter logistic curve using GraphPad Prism, version 10.2.3 (347).

### Human cell toxicity tests

A549 (human alveolar basal epithelial), HEK293 and HEPG2 cell lines were maintained using standard culture conditions in Gibco DMEM high glucose, pyruvate medium. Cells were seeded in T75 flasks and incubated at 37 °C under a 5% CO_2_ atmosphere. After trypsinization, A549 (10,000 cells per well), HEK293 (20,000 cells per well) and HEPG2 (50,000 cells per well) cells were seeded into tissue-culture 96-well plates and incubated at 37 °C for 24 h, with 100-μl volume in each well. Polyene (AmB and **Nys34**) stock solutions were prepared in DMSO and the final concentrations for HEK293 were set at 400 μg ml^−1^, 200 μg ml^−1^, 100 μg ml^−1^, 50 μg ml^−1^, 25 μg ml^−1^, 12.5 μg ml^−1^, 6.25 μg ml^−1^ and 3.125 μg ml^−1^, the final concentrations for A549 were set at 800 μg ml^−1^, 400 μg ml^−1^, 200 μg ml^−1^, 100 μg ml^−1^, 50 μg ml^−1^, 25 μg ml^−1^, 12.5 μg ml^−1^ and 6.25 μg ml^−1^ and the final concentrations for HEPG2 were set at 400 μg ml^−1^, 200 μg ml^−1^, 100 μg ml^−1^, 50 μg ml^−1^, 25 μg ml^−1^, 12.5 μg ml^−1^, 6.25 μg ml^−1^ and 3.125 μg ml^−1^. The compounds were added to randomly assigned rows on tissue-culture 96-well plates keeping final DMSO concentration at 2%. The wells containing cells and media at 2% DMSO were used as viability controls whereas wells containing media at 2% DMSO and no cells were used as negative controls. The compound-treated plate was incubated at 37 °C under a 5% CO_2_ for 18 h. A colorimetric MTS reduction assay was performed by the addition of MTS reagent (CellTiter 96 AQ_ueous_ One Solution Reagent (Promega) to quantify viable cells) to the polyene-treated 96-well plates. Absorbance at 490 nm was measured using a microtitre plate reader before and 4 h after incubation with MTS. To measure cell viability, absorbance at 490 nm before MTS treatment was subtracted from the reading taken after addition of MTS. The 2% DMSO-treated cells were considered as 100% viability. Cell viability is shown relative to DMSO-only control (100% viability). All assays were done in triplicates. The dose–response curves were plotted using Prism and IC_50_ values were determined by a four-parameter logistic curve using GraphPad Prism. All cell lines were tested negative for mycoplasma and were used as received from Sigma without further authentication.

### Drug tolerability and pharmacokinetic studies in mice

CD1 mice were purchased from the Jackson laboratory and transferred to the University of Manchester animal unit for use under licence no. PP0175051. The mice were quarantined and acclimatized to the facility for a minimum of 1 week before any regulated procedures took place. Mouse husbandry was performed in individually ventilated cages on a 12-hour light and dark cycle with temperature regulated to 21 °C (±2 °C) and 40–50% humidity. Mice were kept in cages containing nesting material and wood chips. Access to water and food was provided ad libitum for the entirety of the study. While on protocol, mice were checked at the start and end of the working day and a third check was introduced when mice started showing signs of infection or 10% body weight loss.

Maximum tolerated dose finding was performed in rising concentrations starting at 1 mg kg^−1^, 2 mg kg^−1^, 5 mg kg^−1^ and 10 mg kg^−1^ delivered by i.p. injection with 1 mouse receiving each dose. Mice were observed continuously over the course of 1 h to monitor for immediate adverse effects and then hourly for a total of 4 h. Mice were anaesthetized with isoflurane (Isofane) before terminal cardiac puncture blood collection followed by cervical dislocation.

Pharmacokinetic (PK) analysis of the **Nys34** compounds was performed with 20 CD1 male and female mice (10 males and 10 females of roughly 20 g). Blood microsamples were taken twice per mouse from the tail vein in a heparinized capillary tube and a third terminal cardiac puncture sample was taken from each mouse to limit the number of mice used for this experiment in line with the 3R’s principles. Microsamples from mice were taken at 0.5 h, 1 h, 2 h, 4 h and 8 h postdosing with the cardiac punctures performed at the 8 h and 24 h postdosing time points following the scheme outlined in ref. ^55^.

The organ homogenates were analysed for **Nys34** concentration by means of the addition of 200% v/w MeOH, followed by sonication at room temperature for 30 min and vigorous vortexing. Extracts were then clarified by means of centrifugation, and the extraction repeated for a total of three times. Combined liver extracts were directly injected for analysis on LC–MS. Combined extracts of lung, spleen and kidney samples were dried under a stream of nitrogen and resuspended in a given amount of MeOH for analysis by means of LC–MS. Lung and spleen samples were resuspended in 50 µl of MeOH, and kidney samples in 80 µl. Whole blood samples were analysed from 5-µl aliquots, which were frozen and lyophilized until dry. Dry samples were resuspended in 23 µl of MeOH, sonicated at room temperature for 30 min and vortexed vigorously. Samples were then clarified by centrifugation and injected for analysis on LC–MS. LC–HRMS for biological sample analysis was performed on a 1290 Infinity II HPLC coupled to a 6546 LC/Q-TOF Agilent instrument, using a Luna Omega 5 μm 100 × 2.1 mm (Phenomenex) column, with a flow rate of 0.5 ml min^−1^, using a solvent system of water and methanol and a gradient of 60–95% methanol with 0.1% formic acid over 8 min.

Population pharmacokinetics modelling was performed in R using nlmixr2. A one-compartment model with first-order absorption and first-order elimination was fitted to the whole blood concentration–time data. The model was parameterized using the absorption rate constant (*k*_a_), clearance (CL) and volume of distribution (*V*). Inter-individual variability was included on CL and *V* using exponential random effects. Residual unexplained variability was modelled using a proportional error structure. Model parameters were estimated using the stochastic approximation expectation–maximization algorithm implemented in nlmixr2. Model adequacy was assessed using standard goodness-of-fit plots and residual diagnostics.

To support regimen selection, toxicity risk was evaluated using peak exposure (*C*_max_) and total exposure (area under the curve). A regimen corresponding to 10 mg kg^−1^ administered every 8 h for 4 doses (q8h ×4) was defined as a toxic reference regimen. Using the population pharmacokinetics model derived from nlmixr2, Monte Carlo simulations were performed for this regimen to generate a distribution of 0–32 h peak concentrations. The median simulated *C*_max_ for the toxic reference regimen was used as the primary toxicity threshold for subsequent screening analyses.

### Infection experiments in mice

For efficacy experiments requiring infections with *Aspergillus fumigatus* conidia; mice between 4 and 5 weeks of age were immunocompromised 24 h before infection by subcutaneous injection of Kenalog (Triamcinolone Acetonide, Bristol Myers Squibb) at 40 mg kg^−1^, on the introduction of immunosuppression the water was supplemented with 2 g l^−1^ antibiotic (Neomycin, Sigma) and changed daily. Mice were infected intranasally with freshly prepared 5 × 10^5^ spores of CEA10 in 40 µl of sterile saline. Ten CD1 (five male and five female) mice were randomly allocated to each treatment arm of the efficacy experiments. Mice were dosed i.p. with 5 mg kg^−1^ **Nys34** or the drug carrier vehicle 10% DMSO in saline q8h ×4 i.p. and, AmB at 1 mg kg^−1^ every 24 h 12 h after infection. Mice were weighed and health checked at each timepoint and culled 32 h after the first dosing.

To determine *A. fumigatus* lung fungal burden, mice lungs were harvested immediately after euthanasia, weighed and transferred to a sterile 2-ml microcentrifuge tube containing 1 ml of sterile PBS supplemented with 0.1% Tween-20. Lungs were then homogenized using a sterile tissue homogenizer until no visible fragments remained. Tenfold serial dilutions of each lung homogenate were prepared in sterile PBS containing 0.1% Tween-20 and 100 µl from each dilution were plated onto SDA plates supplemented with chloramphenicol (50 µg ml^−1^). All samples were plated in technical duplicates and plates were incubated at 30 °C for 72 h. After incubation, *A. fumigatus* CFUs were enumerated and the mean colony count from replicate plates was used to calculate total fungal burden. To allow comparison across mice with different lung sizes, fungal burden was normalized to tissue mass (CFU per gram of lung tissue). Researchers were blinded to sample identity for CFU counts.

### UV–Vis studies to determine polyene-sterol binding

Stock solutions of ergosterol (100 mM) were prepared in chloroform and diluted in DMSO to a final concentration of 1 mM. Polyene stocks (AmB, NysA1, **Nys34**) were prepared in DMSO as 1 mM solutions. Polyene-sterol complexes were prepared in triplicate in a 96-well microplate by combining polyene and sterol stock solutions at defined molar ratios (1:0 to 1:5) as detailed in Supplementary Table 6. Sterol complexes were incubated at room temperature for 30 min before UV–visible absorption spectra were recorded using a microplate spectrophotometer. NysA1 derivatives were scanned from 280 nm to 340 nm and AmB derivatives from 380 nm to 440 nm, with a wavelength interval of 1 nm^6^.

### Effect of sterol precomplexation on polyene antifungal activity

Ergosterol was recrystallized from ethanol and prepared as a stock solution in CHCl_3_. Relevant amounts of ergosterol were dispensed before the solvent was removed under nitrogen gas and fully dried under vacuum overnight. A DMSO solution of polyene was added to solid Erg at a 5:1 molar ratio of Erg:polyene. The resulting suspension was gently vortexed and heated at 80 °C for 1 hour before leaving to cool to room temperature to allow complex formation.

*S. cerevisiae* was grown on SDA solid media for 3 days at 30 °C. To prepare the inoculum, five distinct colonies (greater than 1 mm) were picked and resuspended in PBS with 0.1% Tween-20 and diluted to an OD_600_ of 0.5. This was further diluted tenfold to give a final inoculum of 5 × 10^5^ cells per ml. For tests with *A. fumigatus* A1160, it was grown on SDA solid medium at 37 °C, spores were harvested in PBS + Tween-20 (0.1%) and inocula were adjusted to 5 × 10^5^ spores per ml in sterile distilled water. Compounds were prepared as 2.7 mM (AmB) or 5.4 mM (NysA1 and **Nys34**) stock solutions in DMSO with or without precomplexation with ergosterol. Stocks were serially diluted twofold with DMSO before diluting 100-fold in 2× YPD media (*S. cerevisae*) or Roswell Park Memorial Institute media (*A. fumigatus*). Then 100 µl of each media–polyene solution was added to a CytoOne 96-well plate containing 100 µl of yeast or fungal inoculum in triplicate. Plates were incubated at 30 °C for 24 h before measuring OD_600_. Control wells with 0.5% DMSO were tested to confirm viability and a water only inoculum was included as a sterility control^54^.

### Reporting summary

Further information on research design is available in the Nature Portfolio Reporting Summary linked to this article.

## Online content

Any methods, additional references, Nature Portfolio reporting summaries, source data, extended data, supplementary information, acknowledgements, peer review information; details of author contributions and competing interests; and statements of data and code availability are available at https://doi.org/10.1038/s41586-026-10834-8.

## Supplementary information

Supplementary Information Supplementary Materials and Methods, Tables 1–6, Figs. 1–48 and Refs. 57–69.

Reporting Summary

Supplementary Figures Raw gel scans in Supplementary Fig. 11a–d.

Supplementary Data Gene and protein sequences.

Supplementary Data Source data for Supplementary Figs.

Peer Review File

## Source data

Source Data Figs. 4 and 5 and Extended Data Figs. 1, 2 and 9.

## Data Availability

The whole genome sequences of *Streptomyces kasugaensis* DSM40819 and *Streptomyces netropsis* DSM40846 (*Streptomyces syringium* DSM40846) were deposited at DDBJ, ENA and GenBank and were released under the accession numbers JBNAKK000000000 and JBNCJY000000000, respectively. The taxonomic nomenclature of the organism *Streptomyces netropsis* DSM40846 was changed by GenBank to *Streptomyces syringium* DSM40846. Proteins characterized in this study can be accessed from UniProt using the accession codes presented in Supplementary Fig. 2 and amino acid sequences of the enzymes used in this study are presented in the Excel sheet in the Supplementary Information. All the remaining data are available in the main text or the Supplementary Information. Correspondence and requests for materials should be addressed to J.M. Source data are provided with this paper.
