# Supplementary material for: Enzymatic glycosylation and amidation reshapes polyene bioactivity
Source: Nature. 2026 Jul 29;657(8130):295–303. doi: 10.1038/s41586-026-10834-8 (PMC13538060; doi:10.1038/s41586-026-10834-8)
Supplement: Supplementary file 1 [file 41586_2026_10834_MOESM1_ESM.pdf]

nature portfolio

https://doi.org/10.1038/s41586-026-10834-8

Supplementary information

Enzymatic glycosylation and amidation reshapes polyene bioactivity

In the format provided by the authors and unedited

Materials and Methods

General methods

All chemicals were purchased from Sigma Aldrich UK, Fisher Scientific UK, Bachem, Alfa Aesar, or US Pharmacopeia (USP) unless stated otherwise. Primer oligonucleotides were purchased from Integrated DNA Technologies (IDT). Codon optimised synthetic genes were purchased from Twist Bioscience. General molecular biology enzymes such as restriction enzymes, DNA polymerases, and DNA ligases, including those present in kits such as HiFi assembly mastermix, were purchased from New England Biolabs (NEB). Unless stated otherwise, strains were purchased from the Leibniz Institute DSMZ strain collection and were received as lyophilized stocks **(Supplementary Table 1)**. *In vacuo* solvent evaporation was carried out using a Buchi rotavapor system. Analytical RP-HPLC was performed using a Shimadzu Analytical UHPLC with Diode Array Detector. Columns used are referenced when necessary. Semi-preparative HPLC was performed on a Shimadzu Preparative HPLC system. LC-HRMS was performed on an Agilent 1290 Infinity II, coupled to a 6560 Ion Mobility Q-TOF + 1300 UPLC instrument and 1290 Infinity II HPLC coupled to 6546 LC/Q-TOF (Agilent Technologies). NMR was performed using Bruker 500 and 800 MHz spectrometers and processed with TopSpin v3.6.3 & IconNMR v5.0.11. DNA sequencing was performed using services from Eurofins Genomics. *De novo* whole genome sequencing was performed using a Sequel I PacBio instrument. Samples were centrifuged using Eppendorf 5810R. Bacterial cultures were grown using New Brunswick Innova 44 shaker incubator. Synergy HT Microplate Reader was used for cell viability assays and for monitoring bacterial growth. ThermoFisher Nanodrop Ultra was used to quantify DNA and protein. PCR was performed on Eppendorf Mastercycler Nexus GX2.

Bioinformatics to explore glycosyltransferases and novel polyenes

A genome-wide glycosyltransferase search on *S. noursei* ATCC 11455 was done using the dbCAN3 and HMMER search servers **(Supplementary Table 2)**. The glycosyltransferase *Sn*NysSV was identified using homology searches of SelSV using the BLAST tool (NIH) and BLASTp for protein sequences. The two polyene-associated *Sn*NysSV homologs discovered from the database of non-redundant protein sequences were named as KfuSV (60 % identity) from *S. kasugaensis* and MycS3 from *S. netropsis* (55 % identity). The KfuSV and MycS3 associated putative polyene biosynthetic gene clusters (BGC) were named the kasufungin and mycoheptin BGCs, respectively. The genes, individual modules, domain composition, activity and specificity of each of the BGC was compared to the corresponding modules involved in selvamicin biosynthesis. The putative functions of each gene were predicted based on similarity to homologs from selvamicin and nystatin BGCs. To predict the structures of kasufungin and mycoheptin congeners, the individual AT, KR, DH and ER domains within all polyketide synthase (PKS) proteins were analysed at sequence level to predict domain activity and specificity and activity. Sequence alignments were performed using the Clustal Omega (EMBL-EBI) multiple sequence alignment tool. Bioinformatics-guided structure prediction and annotation of novel previously uncharacterised polyene BCGs was performed using antiSMASH (bacterial versions 5.0-8.0). The tailoring enzymes encoded within the polyene BGCs, were compared with the tailoring steps from the predicted selvamicin. AntiSMASH analysis of the *S. kasugaensis and S. netropsis* genomes revealed that KfuSV and MycS3 were part of TDP-sugar subclusters found within and downstream of the polyene PKSs, respectively.

Further analysis to explore wider range of polyene BGCs

To identify possible sugar-producing BGCs, with homologs to the C35 modifying GT *Sn*NysSV, the NCBI database was queried using cblaster version 1.3.20[57] against *Sn*NysSV, *Sn*NysSVI, and *Sn*NysSVII. Separate BLAST searches were also conducted using various mycosamine-installing GTs **(Supplementary Table 3)** to identify possible second-sugar containing polyene producing BGCs. Both sets of searches were cross-referenced to identify common organisms. The available genomes of organisms common to both searches were analysed by antiSMASH[58] to confirm the presence of possible polyene-forming BGCs. The *Sn*NysSV homologue GTs from organisms that appeared to possess polyene BGCs were then aligned using MEGA12 software with the ClustalW

algorithm, and a phylogenetic tree was produced using the construct neighbour joining tree setting. The phylogenetic tree was analysed and visualised using the interactive tree of life tool (iTOL). The genome neighbourhood of *Sn*NysSV and homologs was analysed using EFI-GNT (Enzyme Function Initiative Genome Neighbourhood Tool) **(Supplementary Fig. 3)**.[59]

Cloning, expression and purification of glycosyltransferases

An *E. coli* optimized gene for the *selSV* sequence was amplified with primers *selSV*_fwd and *selSV*_rev **(Supplementary Table 4)**. The *selSV* gene was subcloned into pET28a using the *NdeI* and *EcoRI* restriction enzymes. *SnnysSV* was amplified from *S. noursei* genomic DNA using primers *SnnysSV*_fwd and *SnnysSV*_rev **(Supplementary Table 4)**. The *SnnysSV* was subcloned into pET28a using NEB HiFi assembly. *SanysSV* was amplified from *S. albulus* genomic DNA using primers *SanysSV*_fwd and *SanysSV*_rev **(Supplementary Table 4)** and subcloned into pET28a using NEB HiFi assembly. *kfuSV* was amplified from *S. kasugaensis* using primers *kfuSV*_fwd *and kfuSV*_rev. *mycS3* was amplified from *S. netropsis* using primers *mycS3*_fwd *and mycS3*_rev **(Supplementary Table 4)**. Both *kfuSV* and *mycS3* were subcloned into pET28a with an N-terminal His tag using the restriction sites *NdeI* and *EcoRI*. *E. coli* BL21(DE3) cells were subsequently transformed with each GT-containing construct for overexpression of the recombinant GTs. *kfuSV* was also obtained as *E. coli* codon optimised gene for improved expression. Luria-Bertani (LB) broth containing kanamycin (50 µg/mL) was inoculated with transformants and incubated at 37 °C overnight. The cells were diluted 1:100 in 2x yeast extract tryptone (2xYT) media and incubated with shaking at 37 °C until the suspension reached an OD_{600} of 0.5-0.7. Protein expression was then induced with the addition of Isopropyl ß-D-1-thiogalactopyranoside (IPTG, 1 mM) and the cultures were incubated at 18 °C for a further 16 hrs before cells were harvested by centrifugation. Cell pellets were resuspended in lysis buffer (50 mM Tris-HCl, 150 mM NaCl, 10 mM imidazole, 10% glycerol, pH 7.5) and lysed by sonication on ice (2 x 6 min, 50% power, 30% cycle). The cell lysates were centrifuged (13,700 rpm, 1 hour) and soluble cell extract was applied to Ni-NTA agarose, pre-equilibrated with lysis buffer. This was then washed with 10 column volumes of wash buffer (lysis buffer with 40 mM imidazole) and eluted using 3 column volumes of elution buffer (lysis buffer with 250 mM imidazole). The eluted protein was subject to buffer exchange using PD-10 desalting columns (GE healthcare) into storage buffer (50 mM Tris-Cl, 150 mM NaCl, 10% glycerol, pH 7.5) and concentrated using Vivaspin 20 centricons (10,000 MWCO, Sartorius Stedim Biotech). Protein aliquots were stored at -80 °C until use.

Scaled up production of polyene sugar derivatives from enzymatic reactions

For scaled up production of glucosylated AmB and Nys, assays contained GT enzyme (15 µM), TDP-D-glucose (2 mM), $MgCl_2$ (2 mM), and polyene (1 mM in DMSO, 10% final DMSO concentration), incubated in a total reaction volume of 10 x 1 mL in Tris-HCl buffer (50 mM, pH 7.5). Reactions were incubated at 30 °C for 18 hrs and were subsequently quenched by the addition of 1 reaction volume of methanol. Quenched reactions were pooled and centrifuged to remove precipitated protein. The clear supernatant was evaporated *in vacuo* to dryness. Dry material (AmB-glc and Nys-glc) was dissolved in DMSO (1-5 mL) and used for purification by semi-preparative RP-HPLC (Shimadzu Prominence HPLC with a Phenomenex Gemini C18 column 250 x 10 mm, 5 µm particle size) using a 5 mL/min flow rate, with a solvent system of water and methanol and gradient of 60%-95% methanol over 30 min.

KfusSV structural modelling, docking and site directed mutagenesis

KfuSV was modelled using the Proteinix web server[60] with additional ligands, TDP-glucose and AmB, to identify the putative substrate binding pocket. The generated structure predictions were visualised using PyMOL software[61], the structure of NysA1 was mapped to the position of the bound AmB to produce the final model. Primers were designed for use with the NEB Site directed mutagenesis kit **(Supplementary Table 5)** and PCR products were processed using NEB KLD as per the manufacturer's instructions to get circularised DNA for transformation into DH5a. Transformation plates were incubated at 37 ºC overnight before relevant colonies were picked for inoculation into

LB media (10 ml) with the addition of 50 μg/ml of kanamycin. The media was incubated at 37 ºC overnight. Plasmids from overnight cultures were isolated using QIAGEN MiniPrep kit, as per the manufacturer's instructions. Successful mutagenised plasmids were used to transform BL21(DE3) for protein production. The KfuSV variants were purified and assayed for activity using TDP-glucose and either NysA1 or AmB under standard *in vitro* GT glycosylation assay conditions as described in the *in vitro* GT glycosylation assays section above.

PcsA tagging, expression and purification

Given that the structure of PcsA is unknown and previous attempts to purify this enzyme were unsuccessful,[48] we used an *in-silico* homology modelling guided intra-loop His-tagging approach. An *E. coli* optimized gene for the PcsA sequence was ordered and the flexible loop regions were targeted for His-tag insertion at 5 different positions using specific primers **(Supplementary Table 4)**; *PcsA*-Tag1 (T67-V72), *PcsA*-Tag2 (R233-T236), *PcsA*-Tag3 (L296-M305), *PcsA*-Tag4 (F300-R301), and *PcsA*-Tag5 (D303-N304) using Q5 site-directed mutagenesis kit and protocol (New England Biolabs). *E. coli* BL21 (DE3) cells were subsequently transformed with PcsA-Tag1-5-pET28a constructs for over expression. LB (10 mL) containing kanamycin (50 μg/mL) was inoculated with transformants and incubated at 37 °C overnight. 2xYT medium containing kanamycin (50 μg/mL, 1200 mL) was inoculated with 2% of the starter culture and incubated at 37 °C until OD_{600} 0.5-0.6 was reached. Protein expression was then induced with the addition of IPTG (0.5 mM), and the cultures were incubated at 18 °C with shaking. After 18 hrs, cultures were centrifuged (5000 rpm, 10 min), the cell pellets resuspended in ice cold lysis buffer 4 x 40mL (50 mM Tris-HCl, 250 mM NaCl, 10 mM imidazole, 10% glycerol, pH 7) and sonicated on ice (70 W power, 3 x 20 min on and off cycle). The samples were centrifuged (10,000 rpm, 45 min), the resultant supernatant was mixed with Ni-NTA agarose resin and incubated at 4 °C for 45 min. The protein bound resin was washed in buffer (50 mM Tris-HCl, 250 mM NaCl, 40 mM imidazole, 10% glycerol, pH 7) and eluted in 2 mL elution buffer (50 mM Tris-HCl, 250 mM NaCl, 300 mM imidazole, 10% glycerol, pH 7). The resulting elutions of PcsA-Tag1-5 were analyzed by SDS-PAGE. The elutions (PcsA-Tag1-5) were tested for activity with Pim and L-glutamine using the conditions described below (*in vitro* PcsA reaction conditions). Significant activity was displayed by the Tag1 variant with an intra-loop *N*-terminus surface His-Tag, whereas no activity was observed for Tags 2, 3, 4 and 5.

NMR structural characterization of polyene amide derivatives

The PcsA assays were scaled up to 20-40 mL using each polyene (1 mM). To assist in NMR characterisation of polyene amides, PcsA assays were scaled up and ^{15}N-labelled L-glutamine was used to produce ^{15}N-labelled $Pim^{15}NH_2$ and $Nys^{15}NH_2$.The assays after quenching contained 1:1 water to methanol, were dried *in vacuo* to complete dryness at room temperature, and dissolved in DMSO for purification by semi-preparative RP-HPLC (Shimadzu Prominence HPLC with a Phenomenex Gemini C18 column 250 x 10 mm, 5 μm particle size). ^{15}N-labelled $Pim^{15}NH_2$ was purified using 5 mL/min flow rate with a solvent system of water and methanol with a gradient of 25-55% methanol over 22 min and ^{15}N-labelled $Nys^{15}NH_2$ was purified using 5 mL/min flow rate with a solvent system of water and methanol with a gradient of 55-95% methanol over 23 min. The fractions containing pure compounds were pooled, dried *in vacuo* to complete dryness and dissolved in CD_3OD, 10% D_2O for NMR analysis at 800 MHz frequency. COSY spectra were recorded using the clean, in-phase COSY (CLIPCOSY) pulse program.[62] ROESY spectra were recorded using the EASY-ROESY pulse program using a 200ms spin-lock.[63]

Synthesis of polyene amides

A mixture of polyene (0.03 mmol), amine salt (NH_4Cl, $NH_2OH.HCl$, $NH_2NH_2.HCl$, serinol hydrochloride) each at a concentration of 0.09 mmol, Et_3N (21 μL, 0.15 mmol) in DMF (10 mL) was stirred for 15 minutes at 0 °C before PyBOP (23.4 mg, 0.045 mmol) was added under a nitrogen atmosphere and the mixture stirred for 2 hrs at 0 °C. After completion of the reaction, the product was precipitated and washed with anhydrous diethyl ether (10 ml). The suspension was centrifuged (8000 rpm, 5 min). The solvent was decanted, pellet dissolved in methanol and purified by flash

chromatography (Biotage® Sfär C18 Duo) with a 100 Å, 30µm column using water and methanol gradient of 10% to 100%. Pure fractions were concentrated under reduced pressure and dried using a lyophiliser. Nys12, Nys13, Nys14, Nys31 and Nys34 were re-purified by semi-preparative RP-HPLC using Phenomenex Gemini C18 column (250 × 10 mm, 5 µm particle size). For Nys12, Nys13, Nys14 water and methanol gradient of 70-80 % over 28 min and for Nys31, Nys34 gradient of 70-90 % over 28 min was used. Pure compounds including Pim derivatives were dissolved in DMSO-d6 for NMR analysis at 500MHz frequency.

Solubility Test

NysA1 is soluble in water at 0.1 mg/mL.[64] Fifteen times concentrated saturated solutions (1.5 mg/mL) of NysA1 and NysA3 were prepared in methanol. A 100 µl portion of each stock was dried under vacuum for 2 hrs. Aqueous Tris-HCl buffer (10 mM, pH 7.0) was added to the dry polyenes at room temperature, and the solution was saturated by sonication (15 min) followed by vortexing (30 s). After centrifugation (14,000 rpm, 10 min), undissolved polyene was pelleted and concentrations in the supernatant were determined by measuring UV absorbance at 304 nm, quantified using a standard curve. The assays were repeated in triplicates.

Supplementary Table 1. Organisms used in the study

Organisms	Use in this study
Streptomyces noursei (ATCC 11455)	Nystatin (NysA1) producer
Streptomyces albulus (DSM 40492)	Nystatin (NysA3) producer
Streptomyces nodosus (DSM 40109)	AmB producer
Streptomyces kasugaensis (DSM 40819)	Kasufungin producer
Streptomyces netropsis (DSM 40846)	Mycoheptin and mandimycin producer
Streptomyces rimosus (DSM 40260)	Rimocidin and CE-108 producer
Streptomyces nodosus::Sa-nysSV-sub	Heterologous production of AmB- L-digitoxose
Streptomyces nodosus::kfuSV-sub	Heterologous production of AmB-L-digitoxose
*Streptomyces nodosus:: pSET-ermE**	Used as an empty vector control strain
Streptomyces albus (J1074)	Widely used heterologous host strain
Streptomyces albus::Sa-nysSV-sub	Heterologous production L-ditoxose & GT enzyme SaNysSV
*Streptomyces albus:: pSET-ermE**	Heterologous host with empty vector control
Escherichia coli ET12567 (pUZ8002)	Used for conjugation between *E. coli* and *Streptomyces*
Escherichia coli BL21 (DE3)	Used for protein expression

Supplementary Table 2. *S. noursei* (ATCC 11455) genome wide GT search

GT Families	GT1	GT2	GT4	GT20	GT39	GT28	GT51	GT81	GT87	GT119
	AAF31486.1	ANZ18382.1	ANZ18876.1	ANZ21354.1	ANZ18189.1	ANZ20444.1	ANZ16533.1	ANZ16907.1	ANZ18739.1	ANZ19234.1
	ANZ20999.1	ANZ15605.1	ANZ20673.1	ANZ16908.1		ANZ19235.1	ANZ17510.1	ANZ13498.1	ANZ16883.1	ANZ20345.1
	ANZ20481.1	ANZ18381.1	ANZ18253.1			ANZ16215.1	ANZ18473.1	ANZ21831.1		ANZ18714.1
	ANZ16612.1	ANZ18374.1	ANZ20072.1				ANZ16373.1			ANZ17559.1
	ANZ15651.1	ANZ20636.1	ANZ19547.1				ANZ17222.1			ANZ17969.1
	ANZ15356.1	ANZ18254.1	*ANZ19049.1							ANZ16292.1
	*ANZ15335.1	ANZ20666.1	ANZ19523.1							
	***ANZ14691.1**	ANZ13602.1	*ANZ20681.1							
	ANZ15347.1	ANZ16672.1	ANZ19055.1							
	ANZ18162.1	ANZ14203.1	ANZ18526.1							
	AAF71773.1	ANZ14420.1	*ANZ20242.1							
	*ANZ14075.1									
	(nysDI)	ANZ18159.1	ANZ19184.1							
	ANZ21515.1	ANZ15649.1	ANZ18984.1							
		ANZ14638.1	ANZ16175.1							
		ANZ17324.1	ANZ21121.1							
		ANZ18372.1	ANZ16420.1							
		ANZ19994.1	ANZ18529.1							
		ANZ18334.1	ANZ16006.1							
		ANZ17256.1	ANZ19798.1							
		ANZ21386.1	ANZ20071.1							
		ANZ18296.1	ANZ16950.1							

Key
red SelSV homologs 32-34% amino acid sequence identity
red *Sn*NysSV
*related to secondary metabolism

Supplementary Table 3. The mycosamine-installing GTs used to identify possible polyene BGCs

Mycosamine-installing GT	Accession number
NysDI	AVX51105.1
AmphDI	AAK73511.1
scnK	ADD85130.2
rimE	AAR16517.1
tetrMIII	QIZ24108.1
pimK	CAC20918.1
lcm4	QSE03587.1

Supplementary Table 4. Oligonucleotides used in this study

Primer	Sequence 5' to 3'
*selSV*_fwd	GGGCATATGGTGCGTGTTTTATTCGCAATCAGC
*selSV*_rev	GGGGAATTCTTAACGCGCGGCCAACGCCTCCAG
*Sn-nysSV*_fwd	TGCCGCGCGGCAGCCATATGATGCGCGTCCTGCTGATC
*Sn-nysSV*_rev	GCGGGAGCAGGCGCATAAGCTAGCATGACTGGTGGACA
*Sa-nysSV*_fwd	GCCTGGTGCCGCGCGGCAGCACGCCCGCCCGGCGGGGA
*Sa-nysSV*_rev	CGGCGGGAGCGGGCGCATGAGAGCTCCGTCGACAAGCTTG
*kfuSV*_fwd	CTAGCCATATGCGCGTCCTGATGATCCCGAGC
*kfuSV*_rev	CTAGGAATTCTCAGCGGACTCGGTCGCGGAC
*mycS3*_fwd	CTAGCCATATGCGGGTCCTGATCATCCCG
*mycS3*_rev	CTAGGAATTCTCAGAGGAGTCGCCCGAG
kfuSV-sub fragment 1_fwd	GAGCAACGGAGGTACGGACATATGCATCGAGCGGGTCATCGCC
kfuSV-sub fragment 1_rev	CTGCGCGAGCGGTGACCTGCGATGCCCG
kfuSV-sub fragment 2_fwd	GCGGTGACCTGCGATGCCCGTCCGCGAG
kfuSV-sub fragment 2_rev	CGAGTCCGCTGAGGGGTCGATATGAAGC
kfuSV-sub fragment 3_fwd	CTGAGGGGTCGATATGAAGCTGTCGGAGCGCGCGGAC
kfuSV-sub fragment 3_rev	GCCGCCGGCTGACGCCCCGAATTCGTAATCATGTCATAGCTGT
Sa-nysSV-sub fragment 1_fwd	GAGCAACGGAGGTACGGACATATGGGGGTGTTCACCGACGTGACC
Sa-nysSV-sub fragment 1_rev	GGCCTGCATGAGCACGTGCGGCACCCCG
Sa-nysSV-sub fragment 2_fwd	CGCACGTGCTCATGCAGGCCAAGATGGAACCCG
Sa-nysSV-sub fragment 2_rev	ACAGCTATGACATGATTACGAATTCGCGAGGTTCTGCCCGATG
16S rRNA_fwd	TGCACAATGGGCGAAAG
16S rRNA_rev	CCCTGCTGAAAGAGGTTTAC
*nysSII*_fwd	TGCATGAACTACCTCTGCTC
*nysSII*_rev	GGTCCTTGTCGGACATCAC
*nysSIII*_fwd	ATCACCGACTCCCACATAG
*nysSIII*_rev	TCGAGGACGATGGAGAAC
*nysSVII*_fwd	GCAACATGATCGTGGAGACC
*nysSVII*_rev	ATGTTGACGAGGTTGTCGTG
PcsA-Tag1_fwd	CACCACCACAGCGGCGTGGCAATTACTTATAGTGGG
PcsA-Tag1_rev	ATGATGATGGCTGCCAGTATCAACGCGCATGGG
PcsA-Tag2_fwd	CACCACCACAGCGGCACTGTTACTAAAATTCGTGAATTAC
PcsA-Tag2_rev	ATGATGATGGCTGCCGCGATCATCAGTATGTTC
PcsA-Tag3_fwd	CACCACCACAGCGGCATGCGCGCCACACCTGAT
PcsA-Tag3_rev	ATGATGATGGCTGCCTAATCCGACAAAATCAACAGAAAAGCTAC
PcsA-Tag4_fwd	CACCACCACAGCGGCCGTCCCGATAATATGCGC
PcsA-Tag4_rev	ATGATGATGGCTGCCAAAATTATCCGCTAATCCGAC
PcsA-Tag5_fwd	CACCACCACAGCGGCAATATGCGCGCCACACCT
PcsA-Tag5_rev	ATGATGATGGCTGCCATCGGGACGAAAATTATCCGC

Supplementary Table 5. Primers for KfuSV mutagenesis

**Primer**	**Sequence 5' to 3'**
H13A_fwd	GGCGGCAACCGCTTTTATGCCTTTGGTTCCGCTGGCTTGGG
H13A_rev	GGGGACGGAATCATTAAGACCCGCATCATATGGCTGC
Y140A_fwd	TCACCATCGAGCCGGAGTCGATCCGCTGTCTGGTCCGTTTCTTGC
Y140A_rev	ACGACCGGGATGCCAAGTGCGCCA
T299A_fwd	TGGTACCGGCGCCGGTATGACGGCTACG
T299A_rev	CCATGATGAACCACAGCATCGCAGTCTGCA
T302A_fwd	CACCGGTATGGCGGCTACGAGCTTTGGCT
T302A_rev	CCGGTACCACCATGATGAACCACAGCATCGC
D318A_fwd	CCAACTGGCGGCTCAGTTCGCCTATGGTGAGCAG
D318A_rev	GGCAGTACCAGCTGCGGTAAGCCAAAGC
Q319A_fwd	ACTGGCGGATGCGTTCGCCTATGGTGAGCAGTTAGCGGC
Q319A_rev	TGGGGCAGTACCAGCTGCGGTAAGCCAAA
M229A_fwd	GGGTAGCGCGACCTTAGAATTAAATGGCGTCCCACTGCTGCG
M229A_rev	AGGCTTACGGCCACTCTCCGGACAGGAGA
T297A_fwd	TCATGGTGGTGCCGGCACCGGTATGACGG
T297A_rev	TGAACCACAGCATCGCAGTCTGCAAAAAGAAGATGCA
P280A_fwd	CGATCCCGTTGCGCTGCATCTTCTTTTTGCAGACT
P280A_rev	ACCATTCTAACCCCGTCGGGTACAGGG
H294A_fwd	TGTGGTTCATGCTGGTGGTACCGGCACCGGTATGACGGC
H294A_rev	GCATCGCAGTCTGCAAAAAGAAGATGCAGCGG

Supplementary Table 6. Polyene-sterol complexes at defined molar ratios

Polyene: Sterol	Polyene (μL)	Sterol (μL)	DMSO (μL)	Water (μL)	Total (μL)
1:0	15	0	75	210	300
1:0.5	15	7.5	67.5	210	300
1:1	15	15	60	210	300
1:2	15	30	45	210	300
1:3	15	45	30	210	300
1:5	15	75	0	210	300

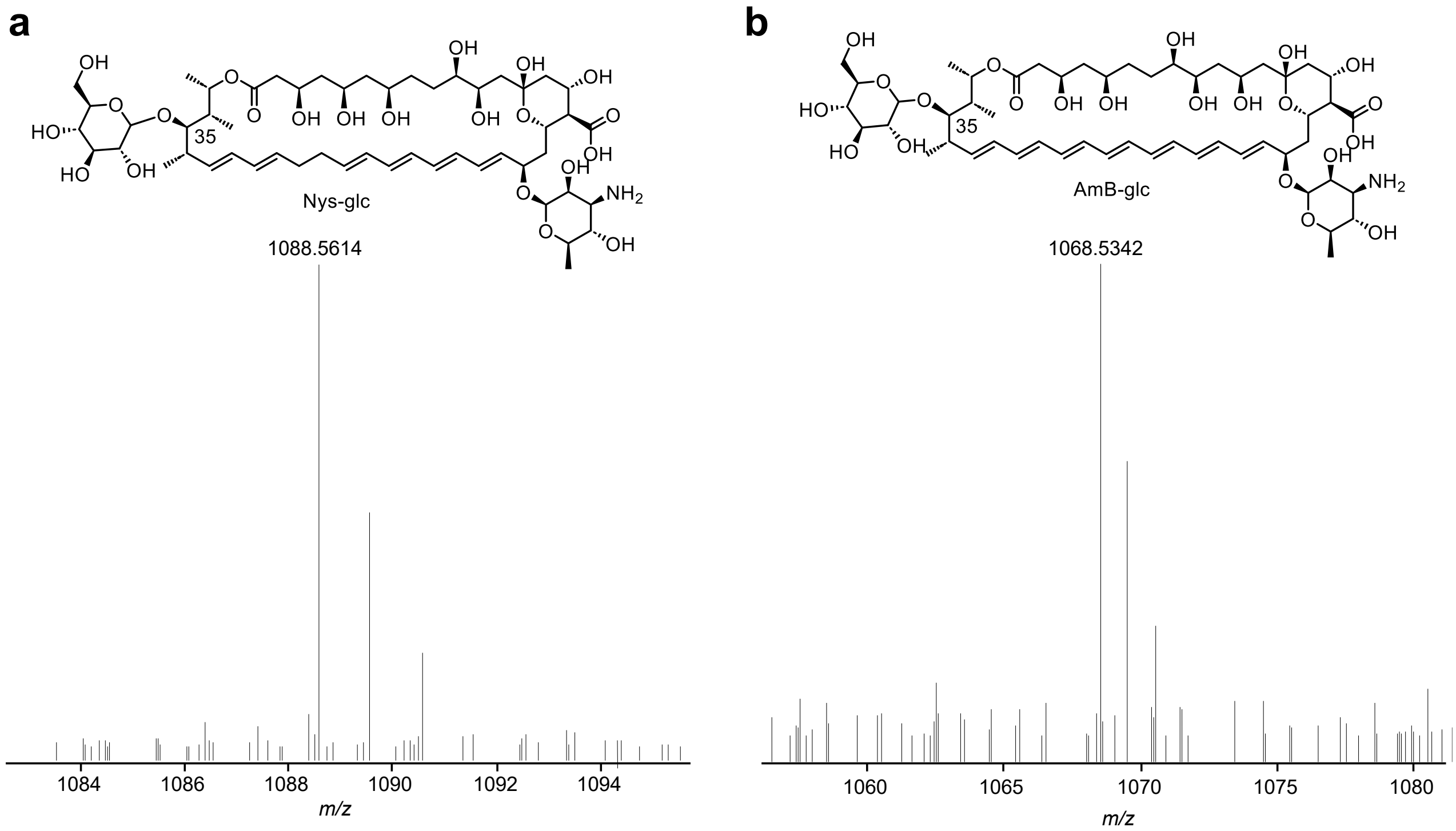

Supplementary Fig. 1. HRMS of SelSV glucosylated NysA1 (Nys-glc) and AmB (AmB-glc). (a) Glucosylated NysA1 calculated *m/z* = 1088.5636 $[M+H]^+$, observed *m/z* = 1088.5614 $[M+H]^+$. **(b)** Glucosylated AmB calculated *m/z* = 1068.5374 $[M+H - H_2O]^+$, observed *m/z* = 1068.5342 $[M+H - H_2O]^+$. The C35-regioselectivity of SelSV is confirmed in reverse glycosylation reactions with NysA3 **(Fig. 3f)**.

a

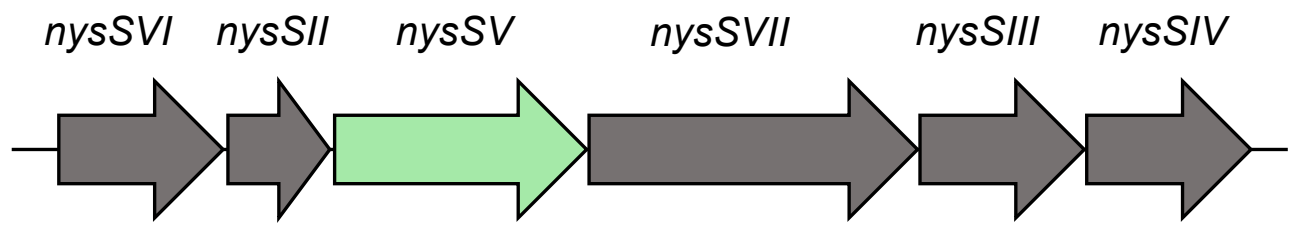

Accession	Gene	nt length	aa length	Predicted function
ANZ14689.1	nysSVI	903	300	NDP-hexose-3-ketoreductase
ANZ14690.1	nysSII	630	209	dTDP-4-keto-6-deoxy-D-glucose epimerase
**ANZ14691.1**	nysSV	1233	410	**Glycosyltransferase**
ANZ14692.1	nysSVII	1365	454	NDP-hexose 2,3-dehydratase
ANZ14693.1	nysSIII	1068	355	Glucose-1-phosphate thymidylyltransferase
ANZ14694.1	nysSIV	996	331	dTDP-D-glucose 4,6-dehydratase

b

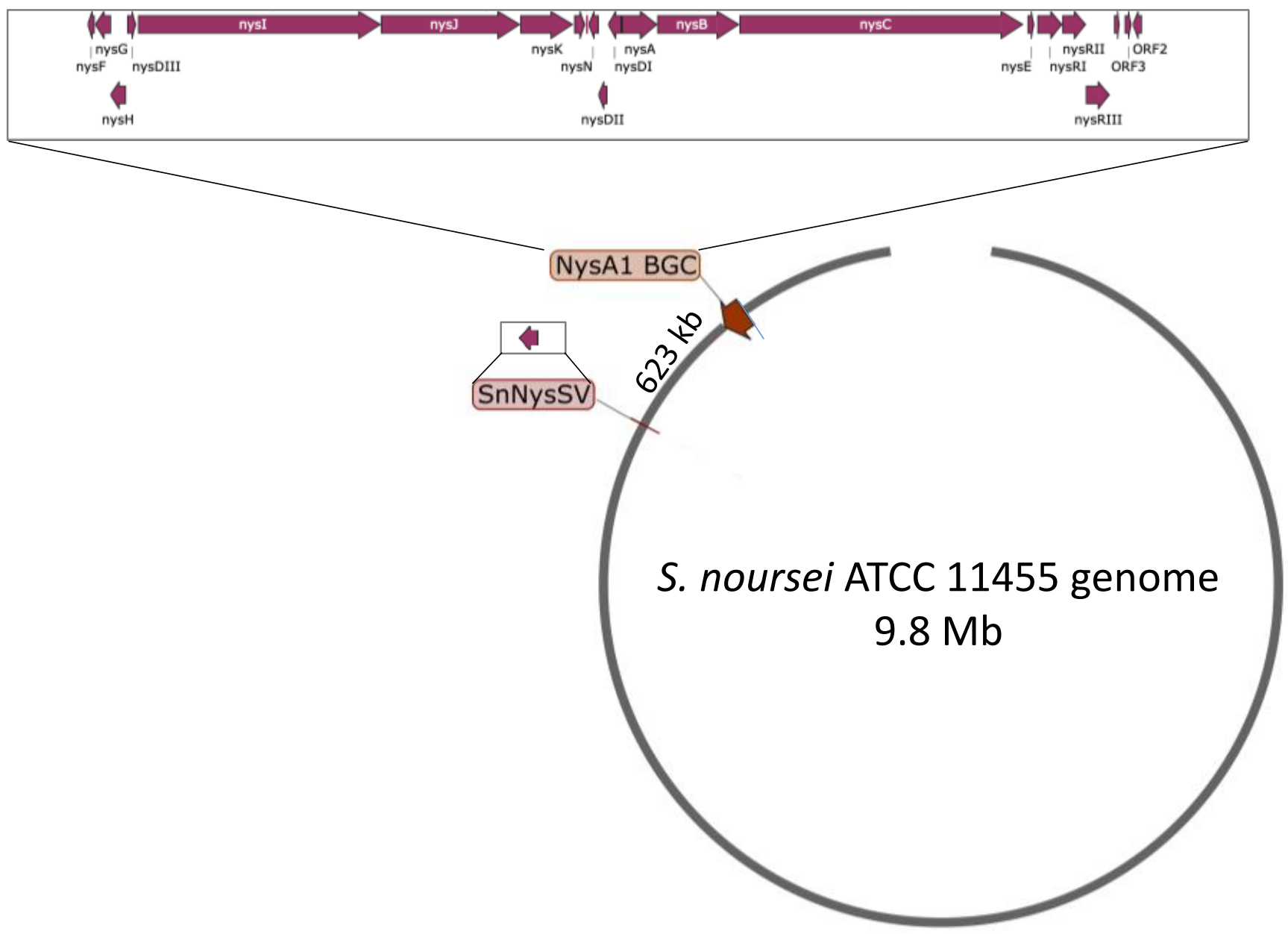

c

Accession	Description	SnNysSV identity	SelSV identity
WP_189859323.1	DUF1205 domain-containing protein *Streptomyces albulus* (SaNysSV)†	98%	35%
MHW4674163.1	DUF1205 domain-containing protein *Streptomyces kasugaensis* (KfuSV)†	60%	33%
MHW4680487.1	DUF1205 domain-containing protein *Streptomyces netropsis* (MycS3)†	55%	31%

Supplementary Fig. 2. Annotation of genes from the *S. noursei* nystatin-associated TDP-deoxysugar cluster. (**a**) Three SelSV homologs are present in *S. noursei*. ANZ15356.1 and ANZ15335.1 are located within erythromycin-like BGC and are predicted to transfer the aminosugar desosamine onto erythromycin (EryCIII). The third *S. noursei* GT (ANZ14691.1), is not associated with any other BGC and was found within a small subcluster that we predicted to encode enzymes for TDP-deoxysugar biosynthesis. (**b**) The small cluster including *nysSV* is 623 kb away from the nystatin BGC in *S. noursei* and is similar to the selvamicin L-4-*O*-methyldigitoxose subcluster, except the putative *O*-methyltransferase (SelSI) is not present. The *S. noursei* cluster contains all the genes necessary for TDP-L-digitoxose synthesis, except a 4-ketoreductase, which is also absent from the related selvamicin subcluster [23] predicted to produce TDP-L-digitoxose. It is possible that a 4-ketoreductase is encoded elsewhere in the *S. noursei* genome and that this subcluster is responsible for the minor product NysA3. We named genes in this *S. noursei* TDP-sugar cluster based on their homology to the genes from the selvamicin BGC.[23] (**c**) Identification of SelSV and NysSV homologues from nonredundant ReqSeq database. †After establishing the function of three homologues they were subsequently named *Sa*NysSV, KfuSV and MycS3. Although *Streptomyces albulus* is reported to produce NysA1, *Sa*NysSV, like *Sn*NysSV, is not associated with a polyene or any other identifiable BGC. KfuSV and MycS3 on the other hand are present in uncharacterised BGCs which we predict to be involved in polyene biosynthesis.

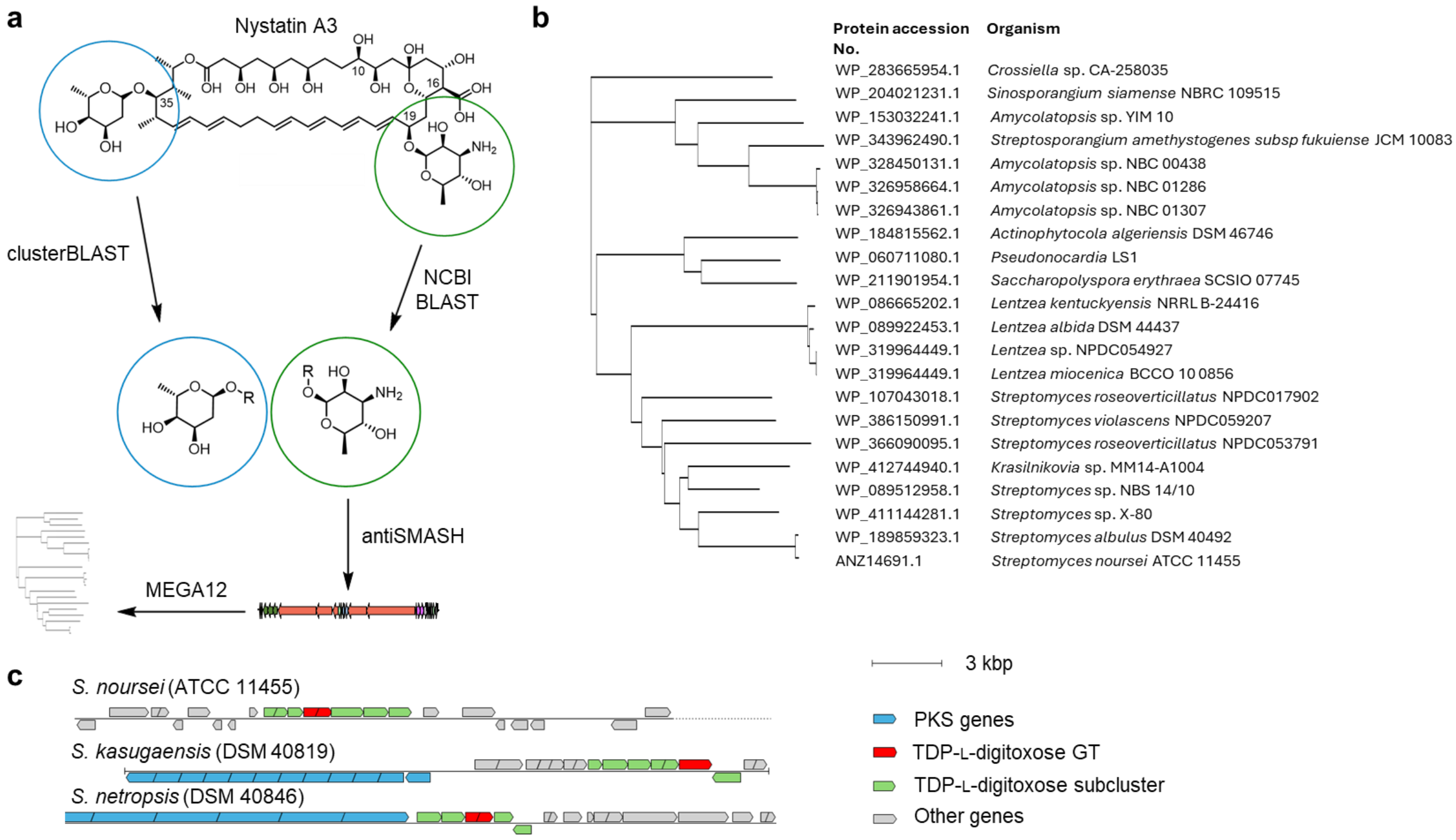

Supplementary Fig. 3. Bioinformatics workflow and output to identify organisms containing homologous sugar synthesis clusters and polyene forming clusters. (a) Bioinformatics workflow showing clusterBLAST of a subset of genes in the *Sn*NysSV subcluster, cross referenced with BLAST searches of NysDI homologues across different polyene forming clusters. The antiSMASH programme was used to identify those species predicted to possess a polyene BGC with sugar subcluster similar to the *Sn*NysSV subcluster. The NysSV homologues from species containing possible polyene BGCs were organised into a phylogenetic tree using MEGA12. **(b)** The phylogenetic tree produced using the workflow from (a), with NysSV homologue accession numbers, and the species names and strain codes. All these species are predicted to possess previously uncharacterised polyene BGCs with two GTs similar to NysDI and NysSV. The strains may therefore have the capability to produce novel polyenes with additional (second) sugars **(c)** Genome neighbourhood diagram for *S. noursei* (ATCC 11455) *Sn*NysSV, *S. kasugaensis* (DSM 40819) and *S. netropsis* (DSM 40846) which show polyene BGCs, including TDP- L-digitoxose subclusters, created using EFI-GNT. It is notable that the three L-digitoxose subclusters have varying numbers of digitoxose biosynthesis genes present, and it has previously been hypothesised that some enzymes in this pathway can have promiscuous activity or that enzymes from elsewhere on the genome can perform the intermediate steps.[23, 65]

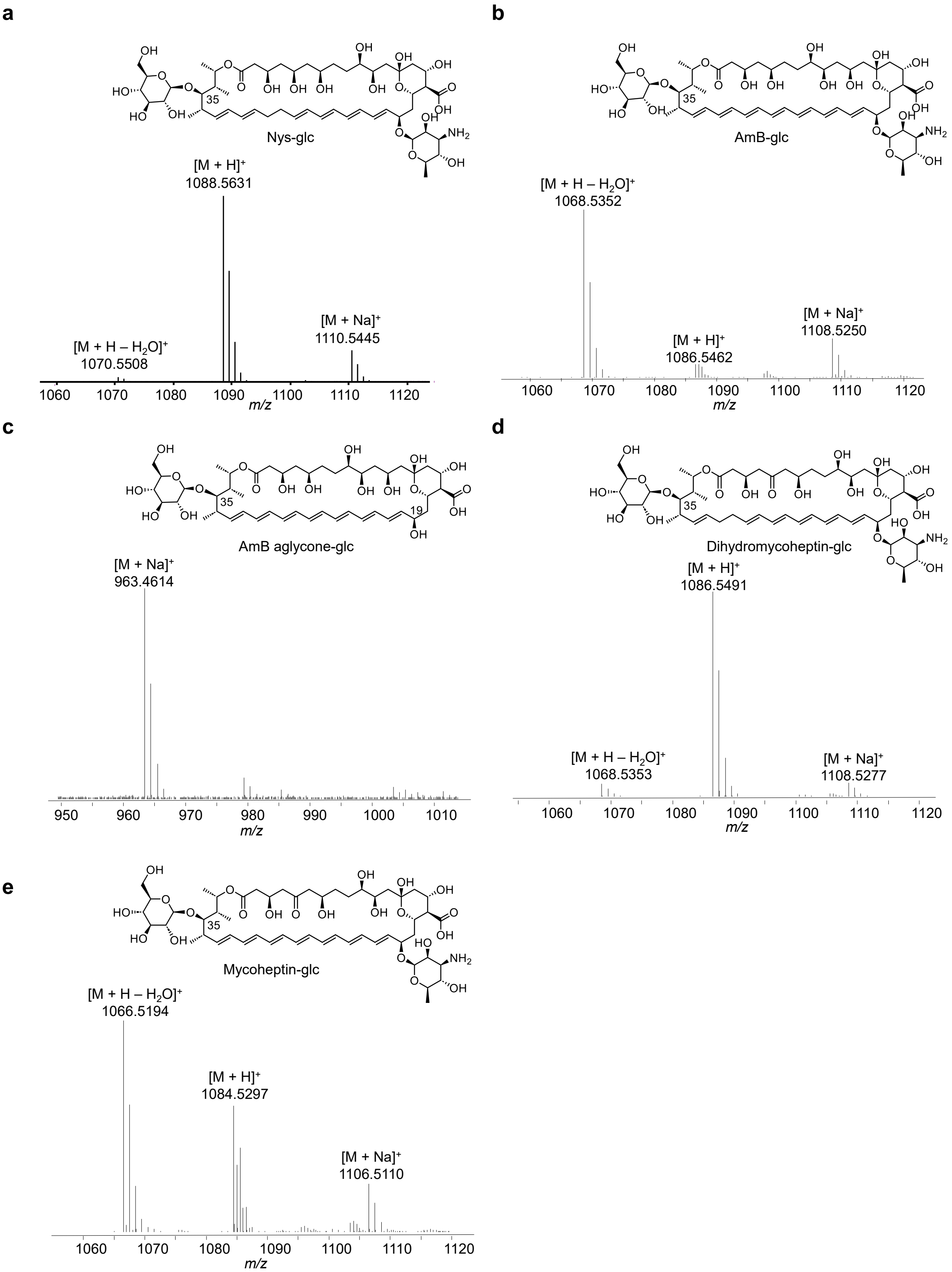

Supplementary Fig. 4. HRMS analysis of glucosylated polyenes produced by KfuSV. (a) Glucosylated NysA1 calculated *m/z* = 1088.5636 $[M+H]^+$, observed *m/z* = 1088.5631 $[M+H]^+$. **(b)** Glucosylated AmB calculated *m/z* = 1086.5479 $[M+H]^+$, observed *m/z* = 1086.5462 $[M+H]^+$. **(c)** Glucosylated AmB-C19-aglycone calculated *m/z* = 963.4560$[M+Na]^+$, observed *m/z* = 963.4614 $[M+Na]^+$. **(d)** Glucosylated dihydromycoheptin calculated *m/z* = 1086.5479 $[M+H]^+$, observed *m/z* = 1086.5491 $[M+H]^+$. **(e)** Glucosylated mycoheptin calculated *m/z* = 1084.5323$[M+H]^+$, observed *m/z* = 1084.5297 $[M+H]^+$.

Amphotericin B					
Sugar	Calculated $[M+H]^+$	Observed $[M+H]^+$	Calculated $[M+H-H_2O]^+$	Observed $[M+H-H_2O]^+$	Δppm
D-Glucose	1086.5479	1086.5462	1068.5374	1068.5360	1.56
D-galactose	1086.5479	---	1068.5374	1068.5360	1.31
D-glcNAc	1127.5745	1127.5715	1109.5639	---	2.66
L-fucose	1070.5530	1070.5535	1052.5425	1052.5413	0.47
2-deoxy-D-glucose	1070.5530	1070.5549	1052.5425	1052.5449	1.77

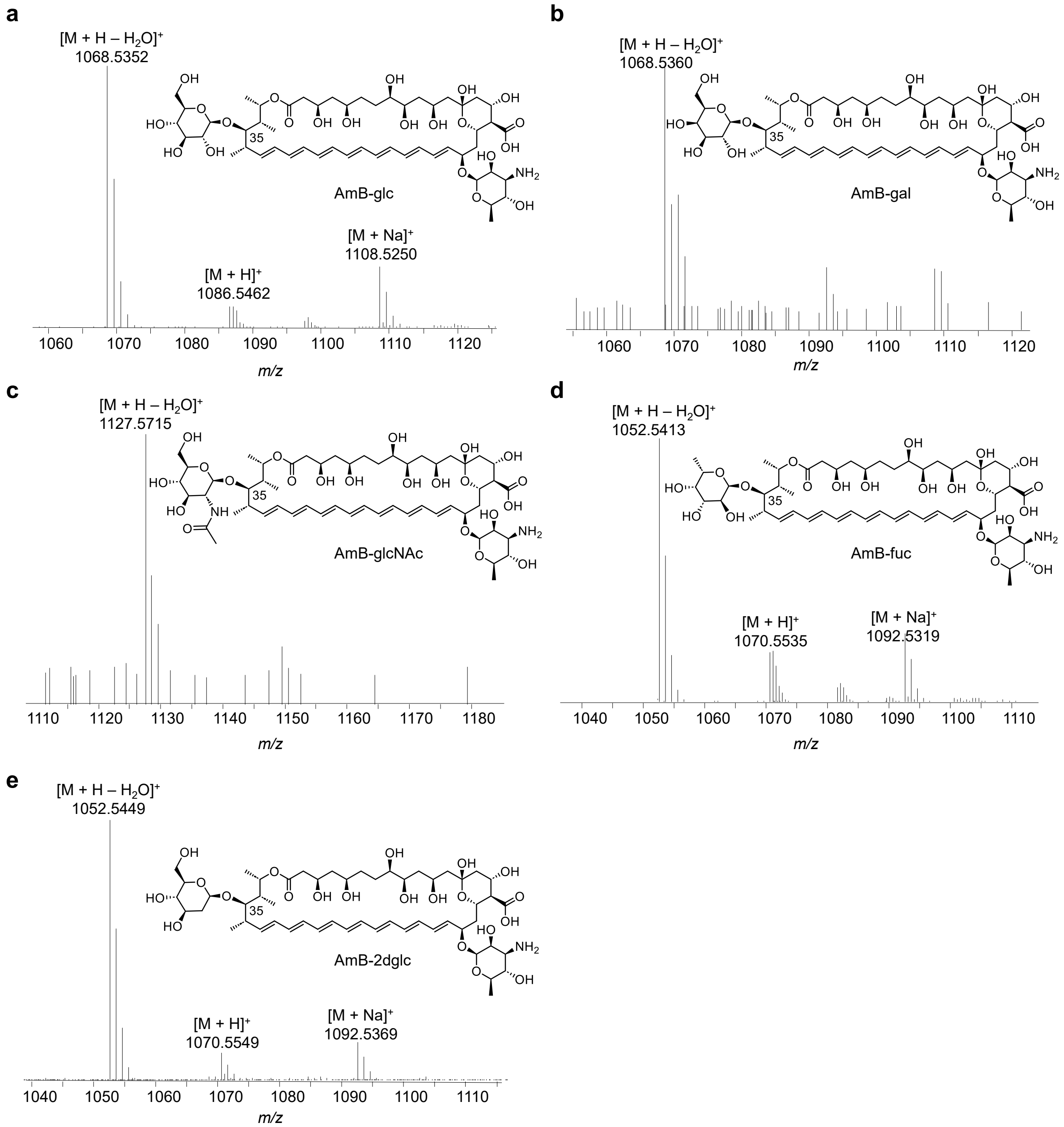

Supplementary Fig. 5. HRMS analysis of glycosylated AmB derivatives produced by KfuSV

Nystatin A1					
Sugar	Calculated $[M+H]^+$	Observed $[M+H]^+$	Calculated $[M+H-H_2O]^+$	Observed $[M+H-H_2O]^+$	Δppm
D-Glucose	1088.5636	1088.5631	1070.5530	1070.5508	0.46
D-galactose	1088.5636	1088.5606	1070.5530	---	2.76
D-mannose	1088.5636	1088.5613	1070.5530	---	2.11
D-glcNAc	1129.5901	1129.5901	1111.5796	---	0
L-fucose	1072.5687	1072.5692	1054.5581	1054.5549	0.47
2-deoxy-D-glucose	1072.5687	1072.5747	1054.5581	1054.5624	5.59
6-deoxy-D-glucose	1072.5687	1072.5769	1054.5581	---	7.65
D-xylose	1058.5530	1058.5599	---	1040.5491	6.43
2-deoxy-D-galactose	1072.5687	1072.5722	1054.5581	1054.5622	3.26
L-glucose	1088.5636	1088.5689	1070.5530	---	4.88
L-mannose	1088.5636	1088.5657	1070.5530	---	1.93

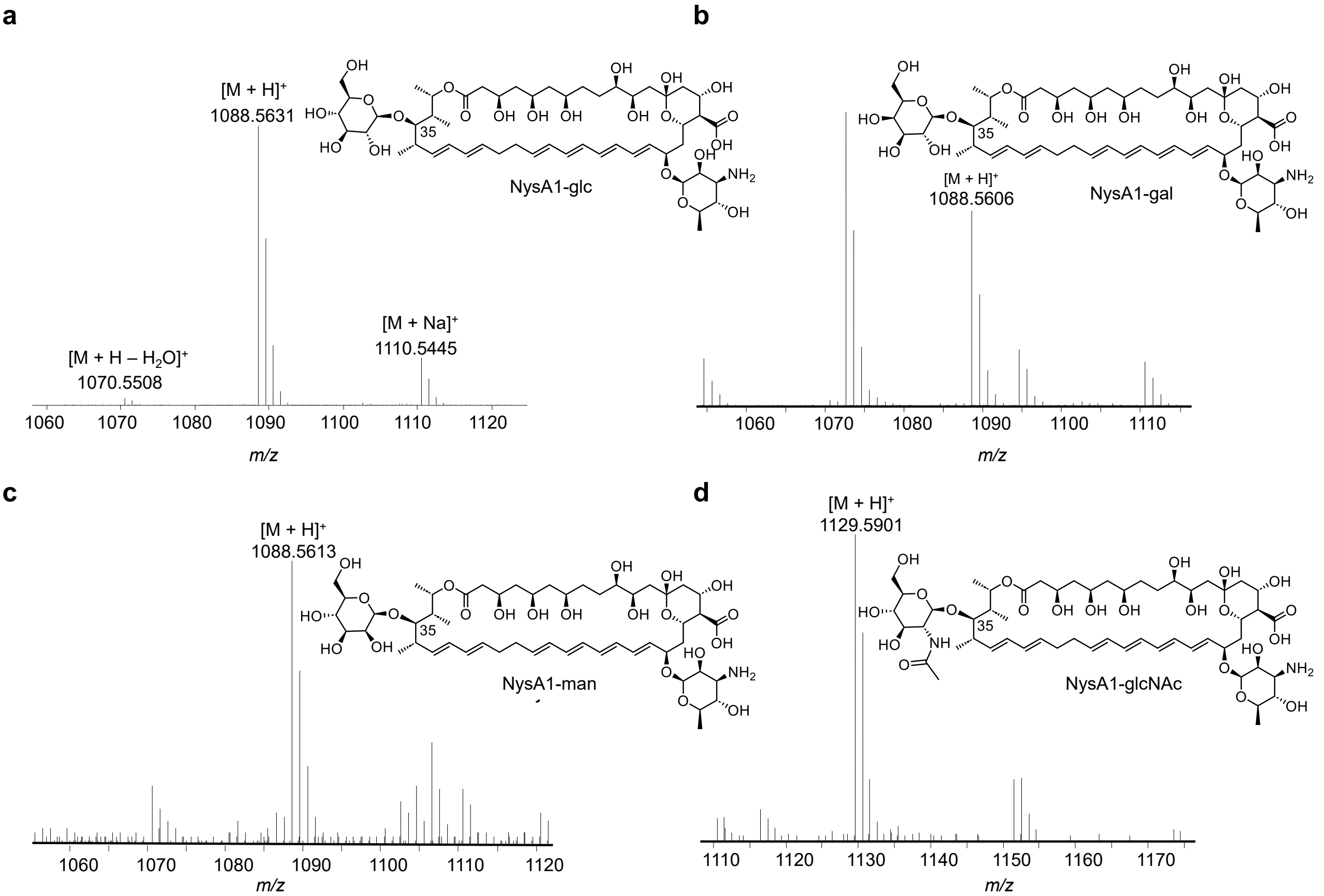

Supplementary Fig. 6. (a-d) HRMS analysis of glycosylated NysA1 derivatives produced by KfuSV

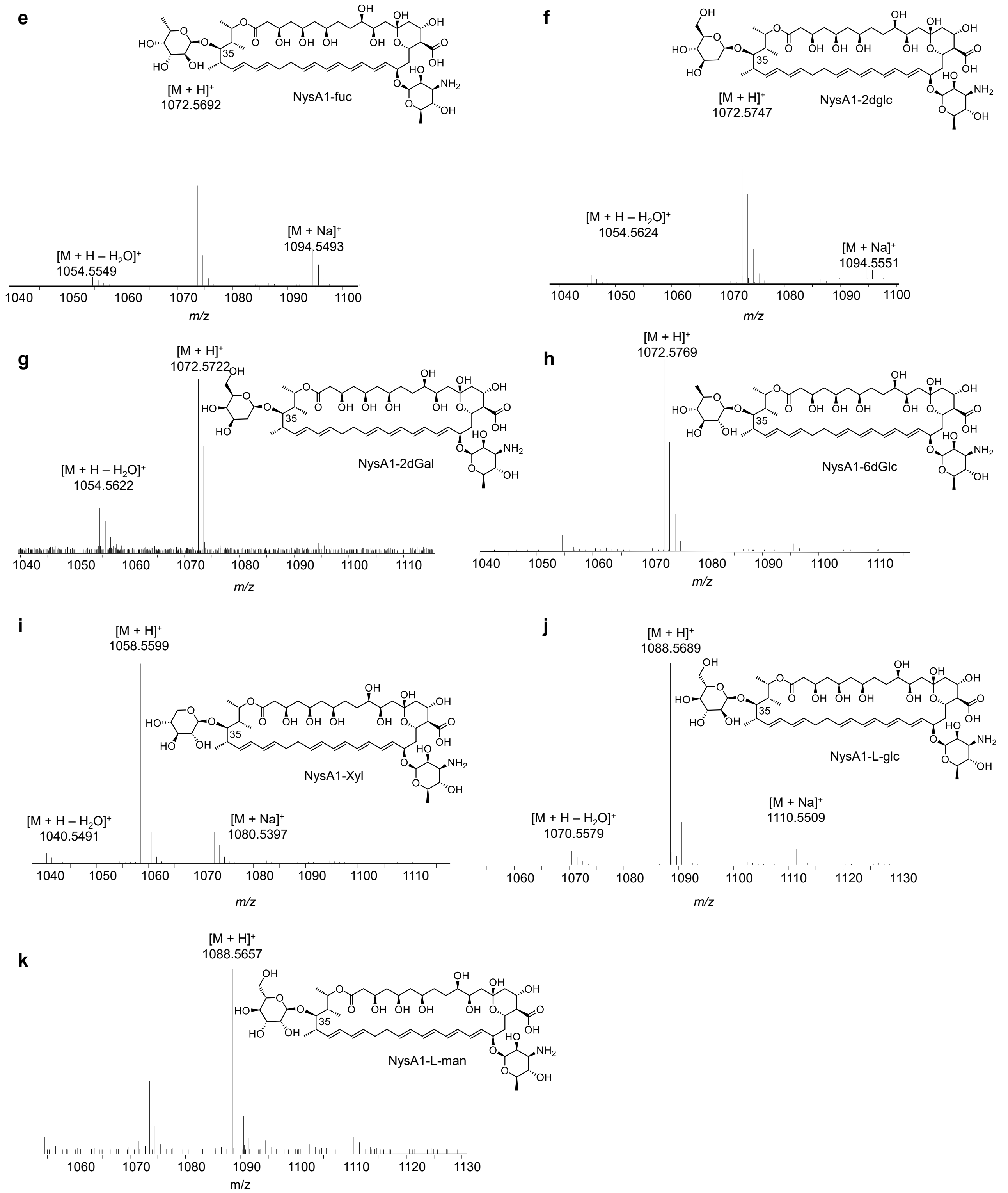

Supplementary Fig. 6 continued. (e-k) HRMS analysis of glycosylated NysA1 derivatives produced by KfuSV

a

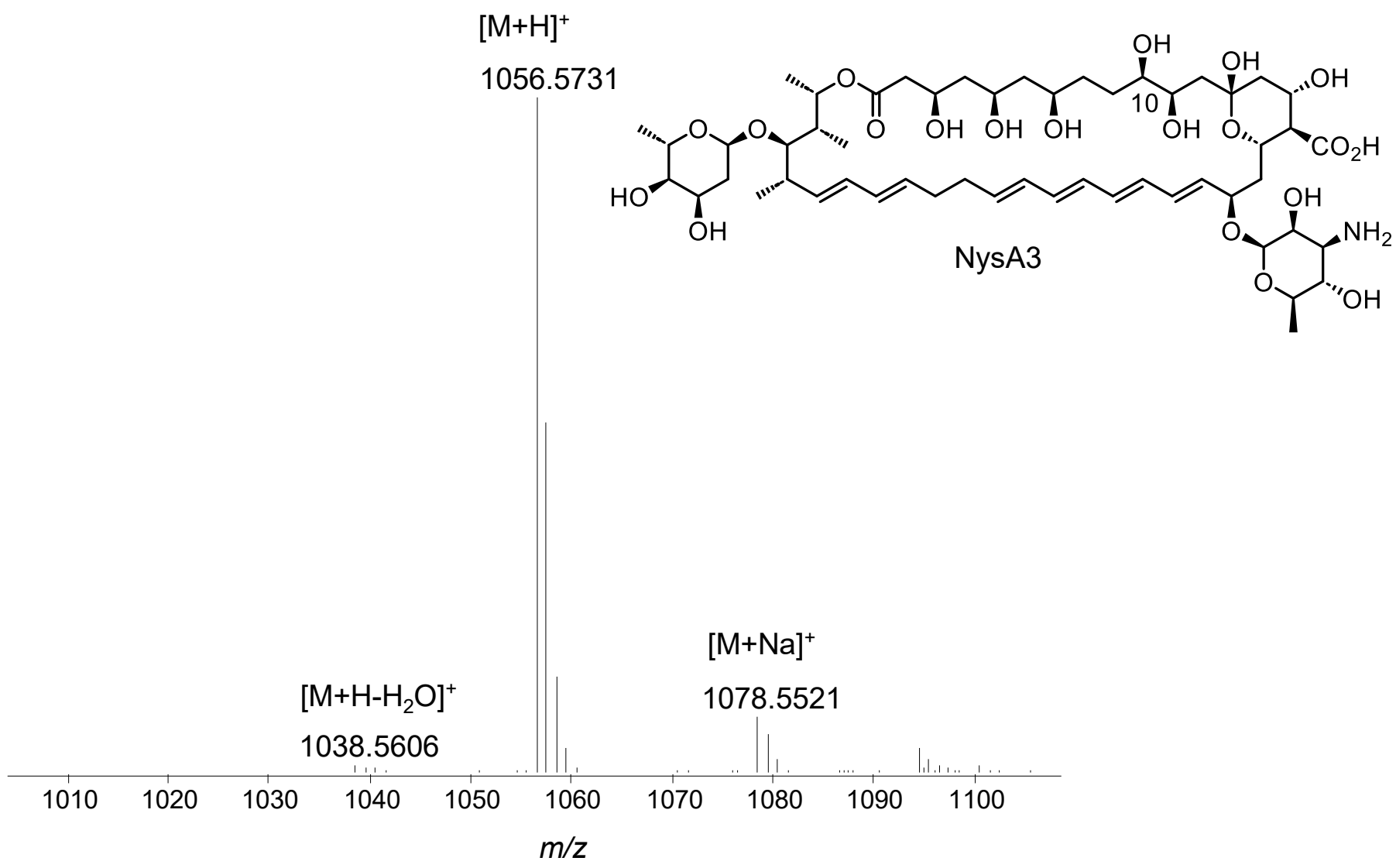

b

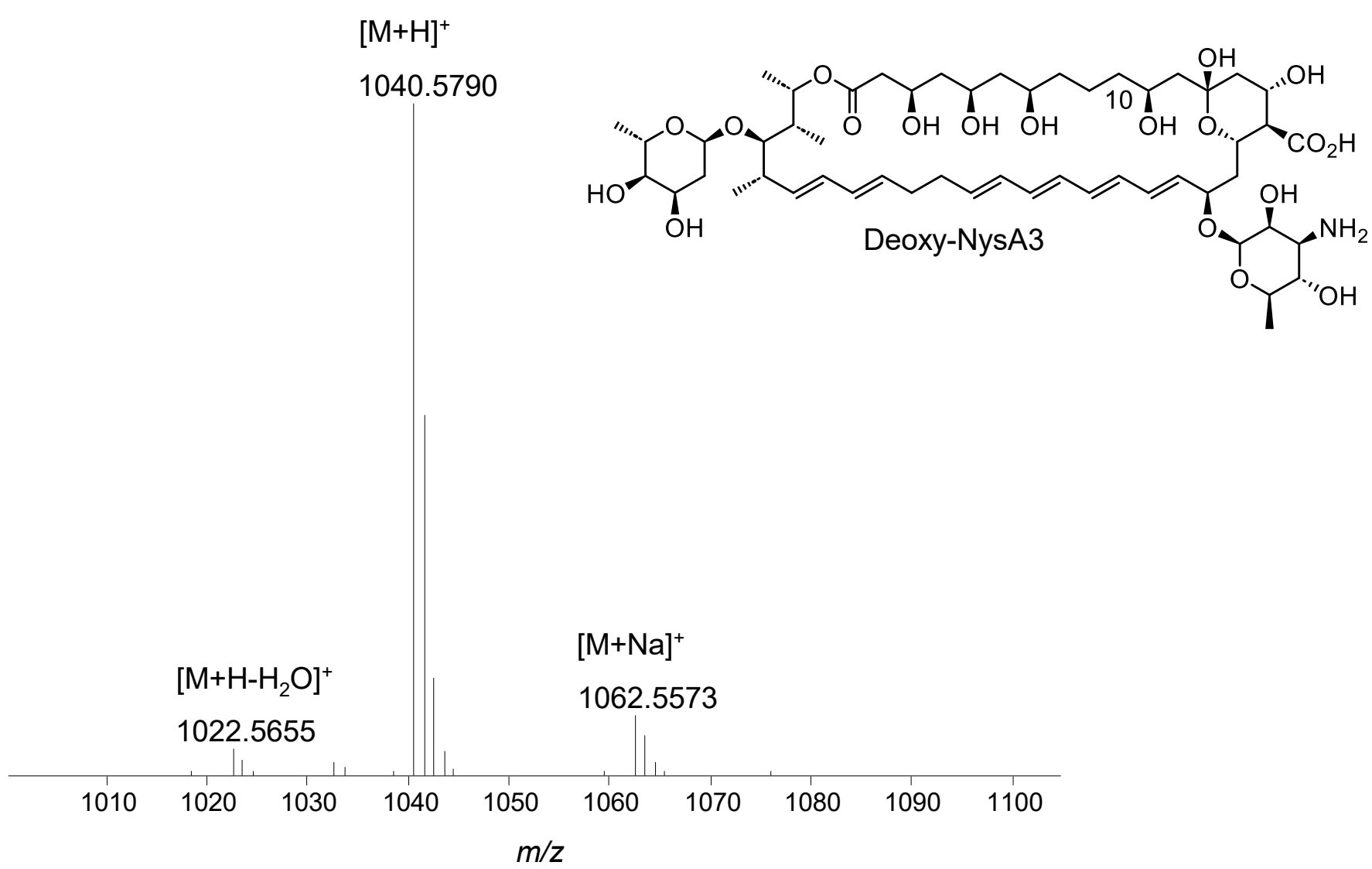

Supplementary Fig. 7. HRMS for tetraenes produced in *S. albulus*. (a) NysA3, calculated mass *m/z* $[M+H]^+$ 1056.5738, observed mass *m/z* $[M+H]^+$ 1056.5731. **(b)** Deoxy-NysA3, calculated mass *m/z* $[M+H]^+$ 1040.5789, observed mass *m/z* $[M+H]^+$ 1040.5790.

a

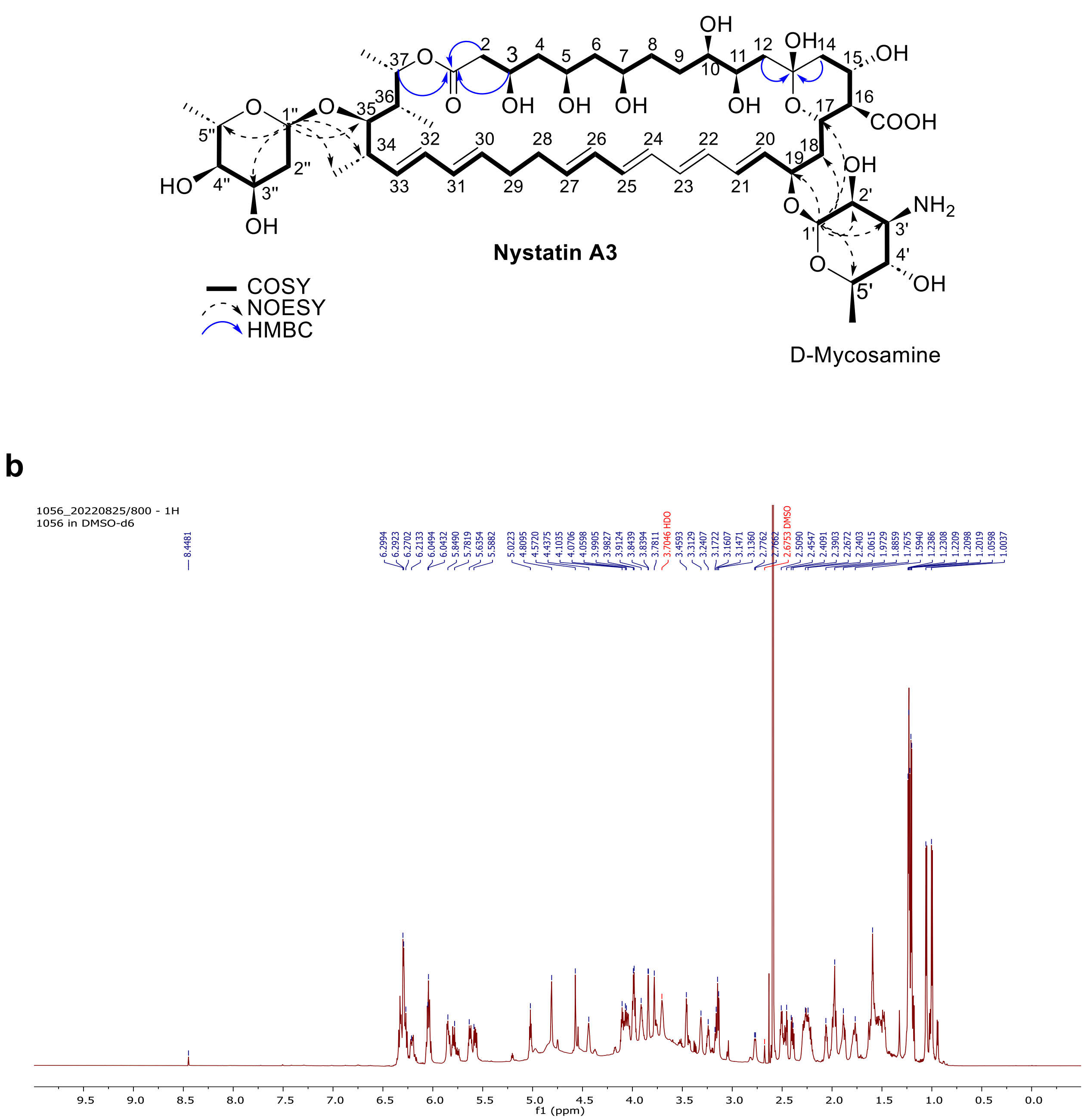

Supplementary Fig. 8. NMR data for NysA3. (a) shows NMR structure of NysA3 established using COSY, NOESY and HMBC correlations. In addition to D-mycosamine at C19, a second sugar is also present which ROESY correlations indicate is at C35. The ^{1}H NMR and COSY data also showed the second sugar is a dideoxy sugar, with coupling constants for H3´´-H4´´ (J = 3.2 Hz) and H4´´-H5´´ (J = 8.9 Hz) consistent with L-digitoxose, which supports the previously proposed structure for NysA3 from *S. noursei*.[33,34] **(b)** shows ^{1}H NMR spectrum of NysA3 in DMSO-*d6*.

c

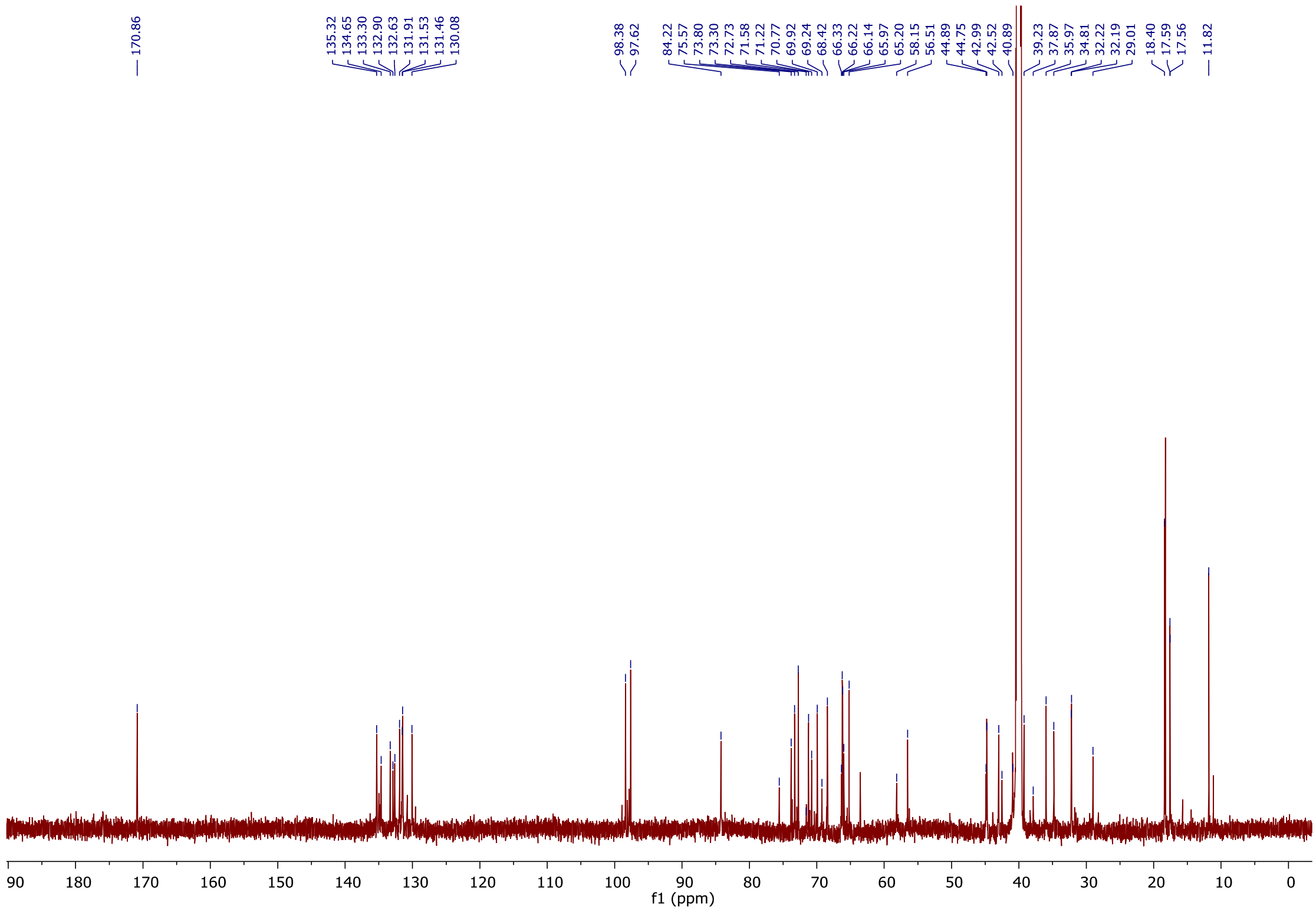

d

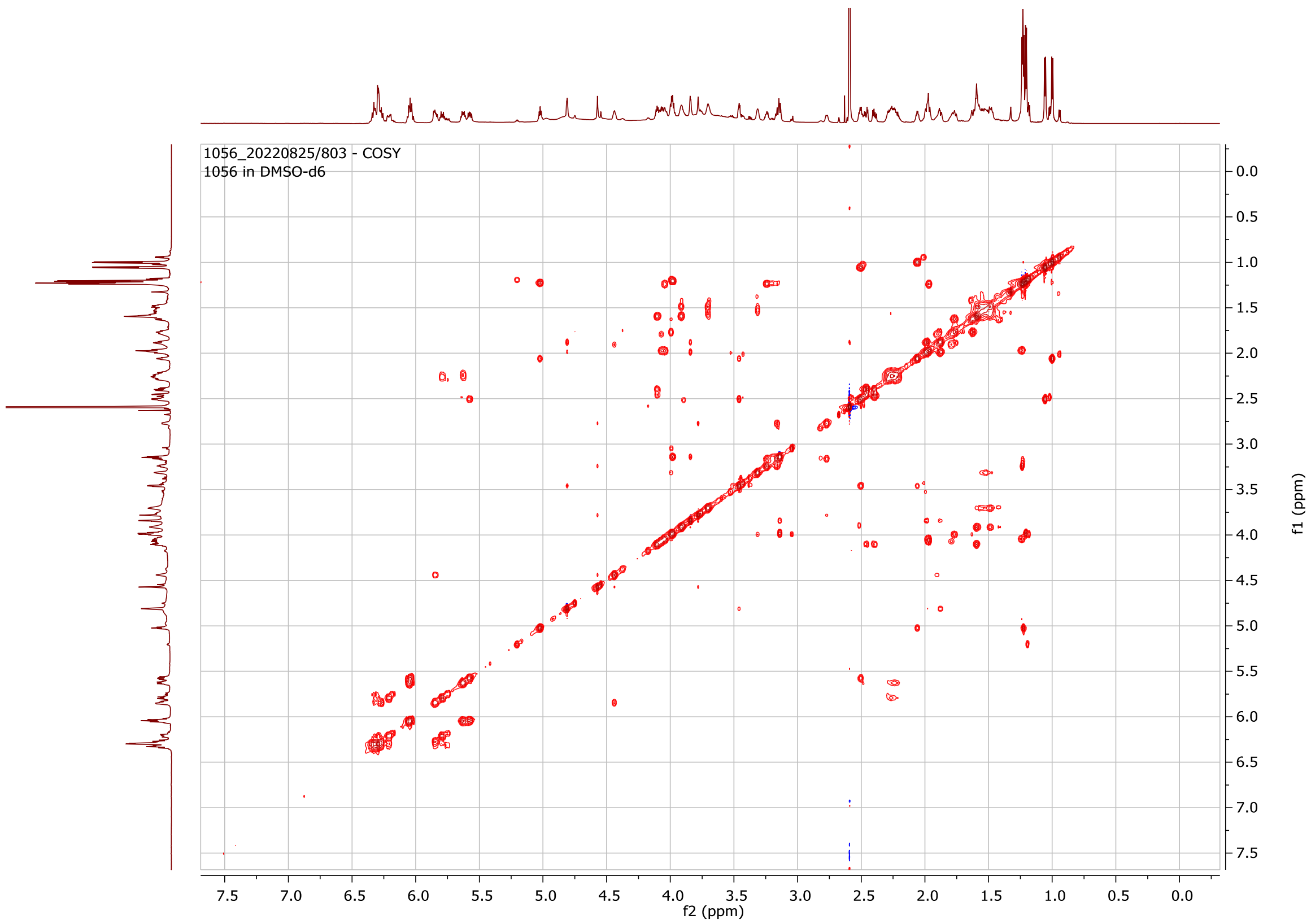

Supplementary Fig. 8. NMR data for NysA3 continued. (c) shows ^{13}C NMR spectrum of NysA3 in DMSO-*d6*. **(d)** shows ^{1}H-^{1}H COSY spectrum of NysA3.

e

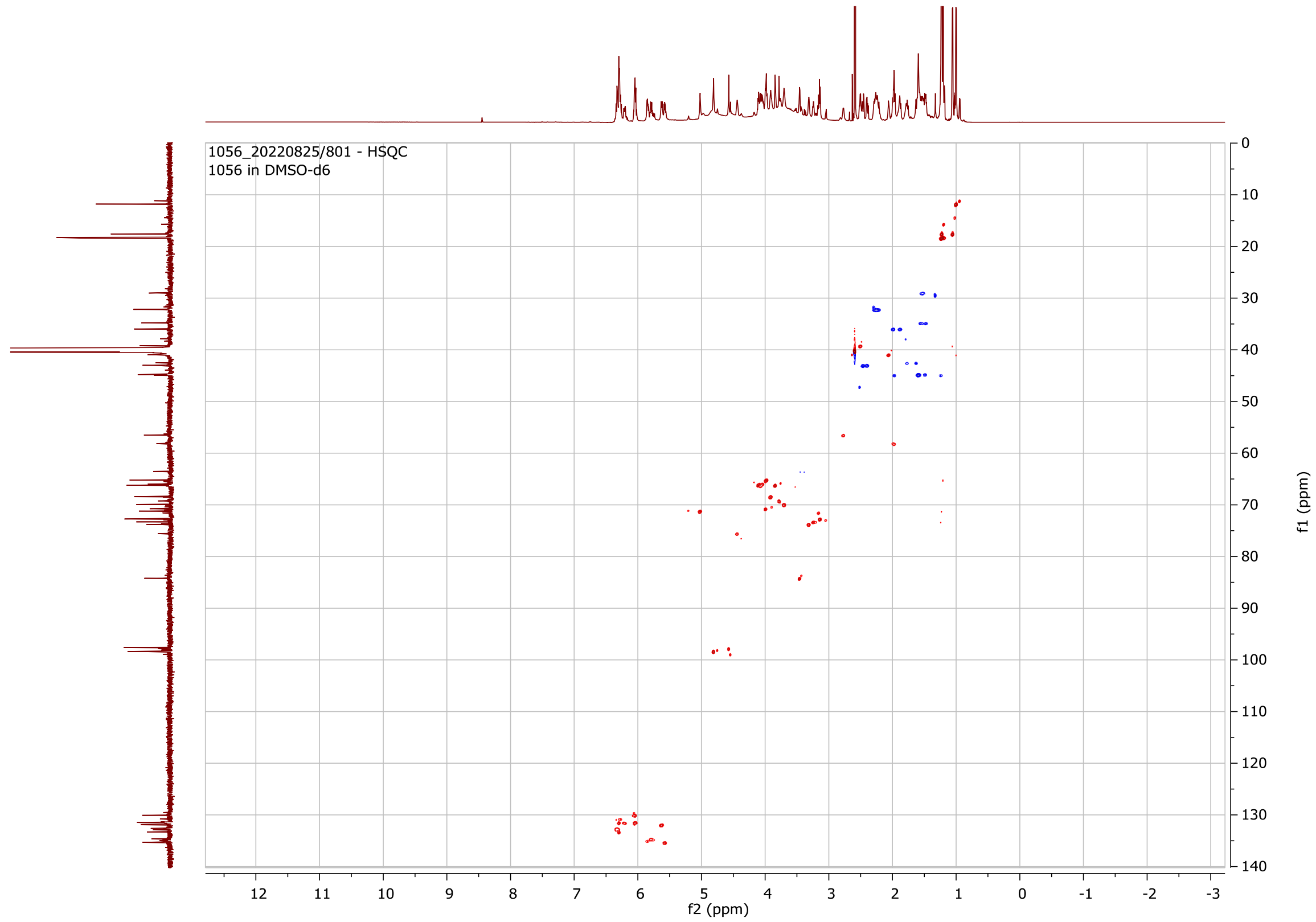

f

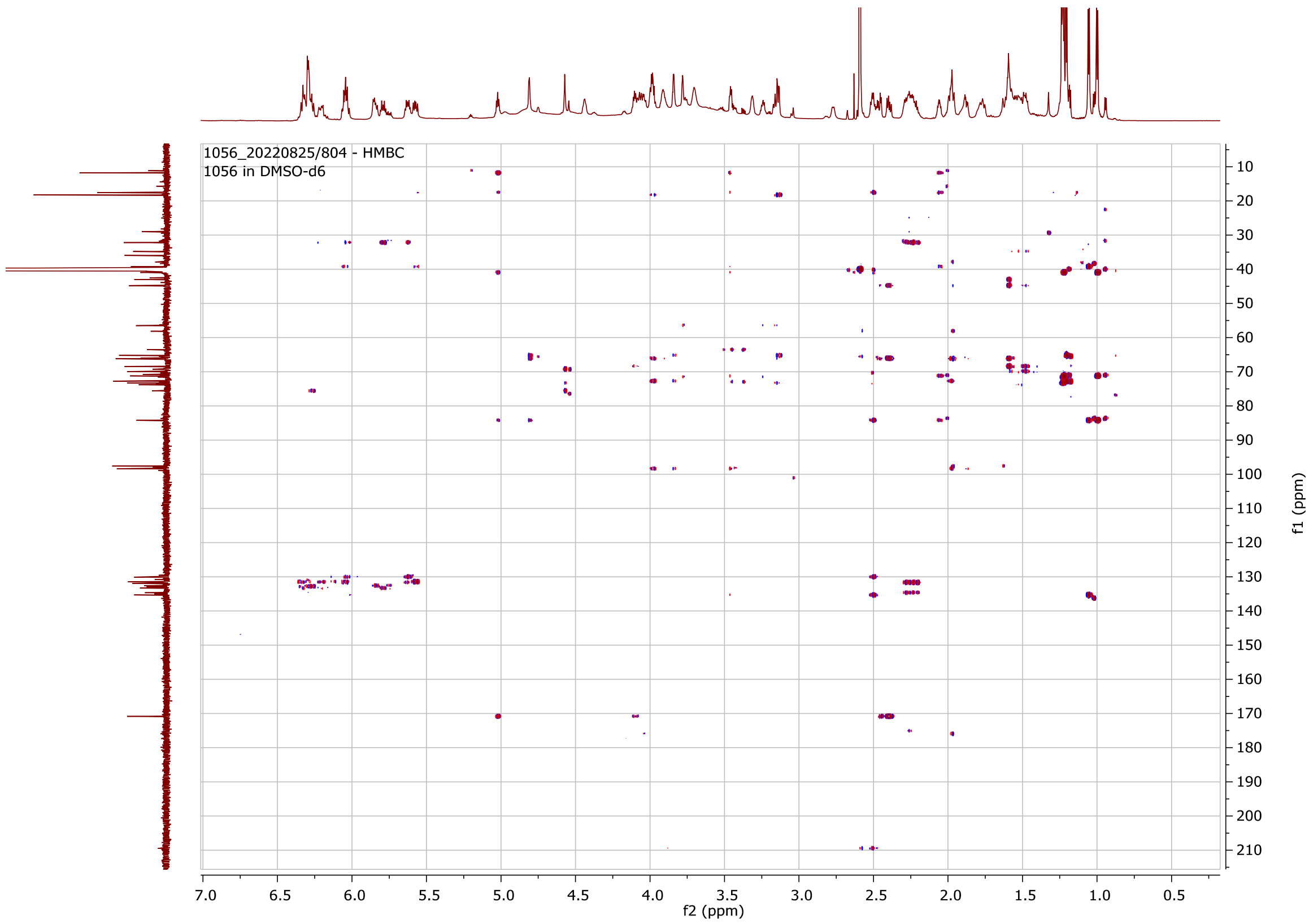

Supplementary Fig. 8. NMR data for NysA3 continued. (e) shows ^{1}H-^{13}C HSQC spectrum of NysA3. **(f)** shows ^{1}H-^{13}C HMBC spectrum of NysA3

g

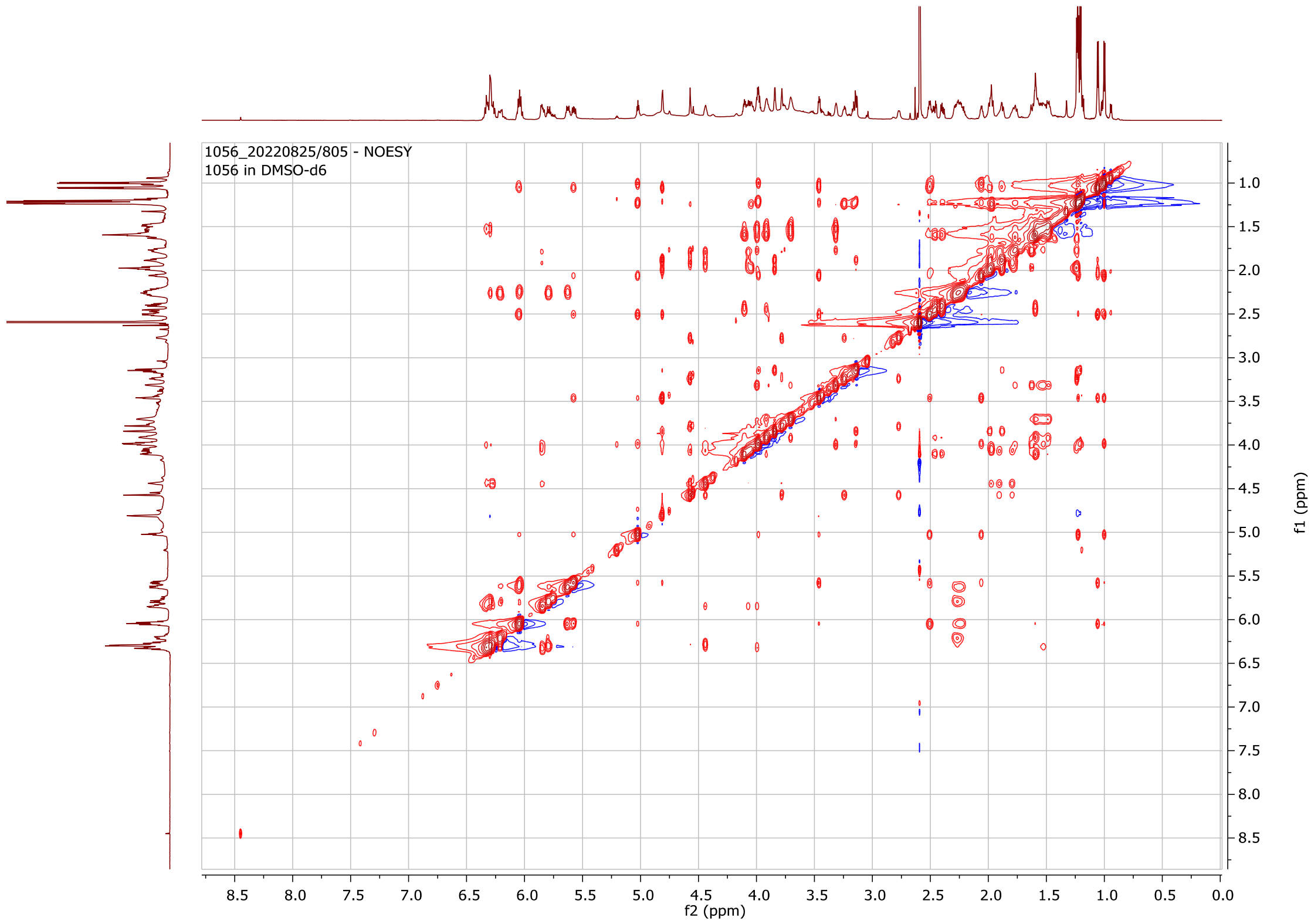

h

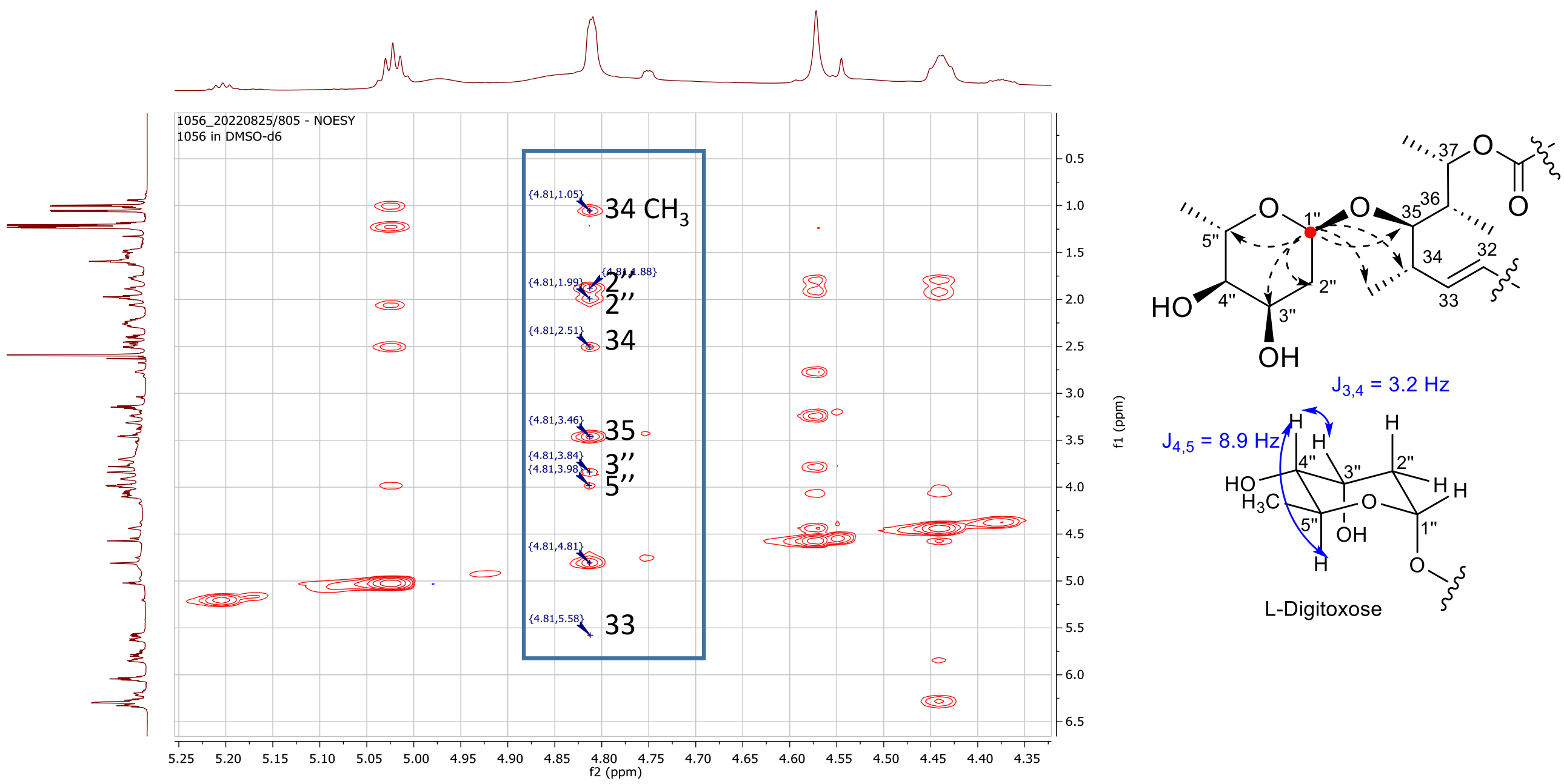

Supplementary Fig. 8. NMR data for NysA3 continued. (g) shows ^{1}H-^{1}H ROESY spectrum of NysA3. **(h)** shows expansion of NysA3 ROESY spectrum. NOESY correlation from H1´´ (δ 4.81) to H35 (δ 3.46), H34 (δ 2.50), C34-CH_3 (δ 1.05) confirm the position of the second sugar. The observed coupling constants H3´´-H4´´ (J = 3.2 Hz) and H4´´-H5´´ (J = 8.9 Hz) are consistent with L-digitoxose.

i

Position	^{1}H-Reported NYST1068 [25]	^{13}C-Reported NYST1068 [25]	^{1}H-Found NysA3	^{13}C-Found NysA3
1				170.86
2	2.20	43.0	2.43	42.99
3	4.04	66.6	4.10	66.14
4	1.42	45.4	1.60	44.75
5	3.81	70.5	3.91	68.42
6	1.35	45.4	1.49	44.79
7	3.60	71.1	3.70	69.92
8	1.42	28.4	1.55	34.81
9	1.42	28.4	1.52	29.01
10	3.07	74.4	3.31	73.80
11	3.79	71.7	3.99	70.76
12	1.42, 1.77	43.8	1.63, 1.77	42.52
13	(6.02)			97.62
14	1.14, 1.87	45.3	1.24, 1.97	44.89
15	3.99	66.2	4.05	65.97
16	2.00	57.9	1.97	58.15
17	4.17	66.1	4.07	66.34
18	1.58, 1.98		1.79, 1.90	37.87
19	4.41	75.0	4.44	75.57
20	5.91		5.84	134.98
21-26	6.11		6.21 – 6.29	130.15 – 133.37
27			5.79	134.65
28			2.27	32.19
29			2.24	32.23
30			5.63	131.91
31	6.15		6.04	131.55
32	6.01		6.04	131.55
33	5.51		5.58	135.32
34	2.42		2.50	39.23
34-CH_3	0.94	18.7	1.05	17.59
35	3.36		3.46	84.22
36	1.88		2.06	40.94
36-CH_3	0.92	12.0	1.00	11.82
37	4.98	71.9	5.02	71.22
37-CH_3	1.15	18.7	1.23	18.40
1’	4.38	96.3	4.57	97.84
2’	3.77	68.0	3.78	69.23
3’ (3’-NH_2)	2.95 (7.96)	55.9	2.77 (8.45)	56.51
4’	3.25	69.5	3.16	71.56
5’	3.17	73.8	3.24	73.30
6’	1.17	18.6	1.23	17.56
1”	4.75	100.8	4.81	98.38
2”	1.93, 1.72	43.1	1.88, 1.98	35.97
3”	-	70.0	3.84	66.22
3”-CH_3	1.11	28.3	N/A	N/A
4”	2.83	78.0	3.14	72.74
5”	3.81	67.0	3.98	65.20
6”	1.13	19.6	1.20	18.25

Supplementary Fig. 8. NMR data for NysA3 continued. (i) shows ^{1}H and ^{13}C NMR spectral data for NysA3 in DMSO-*d6.* Comparison with NMR data for the related Nys1068. [25]

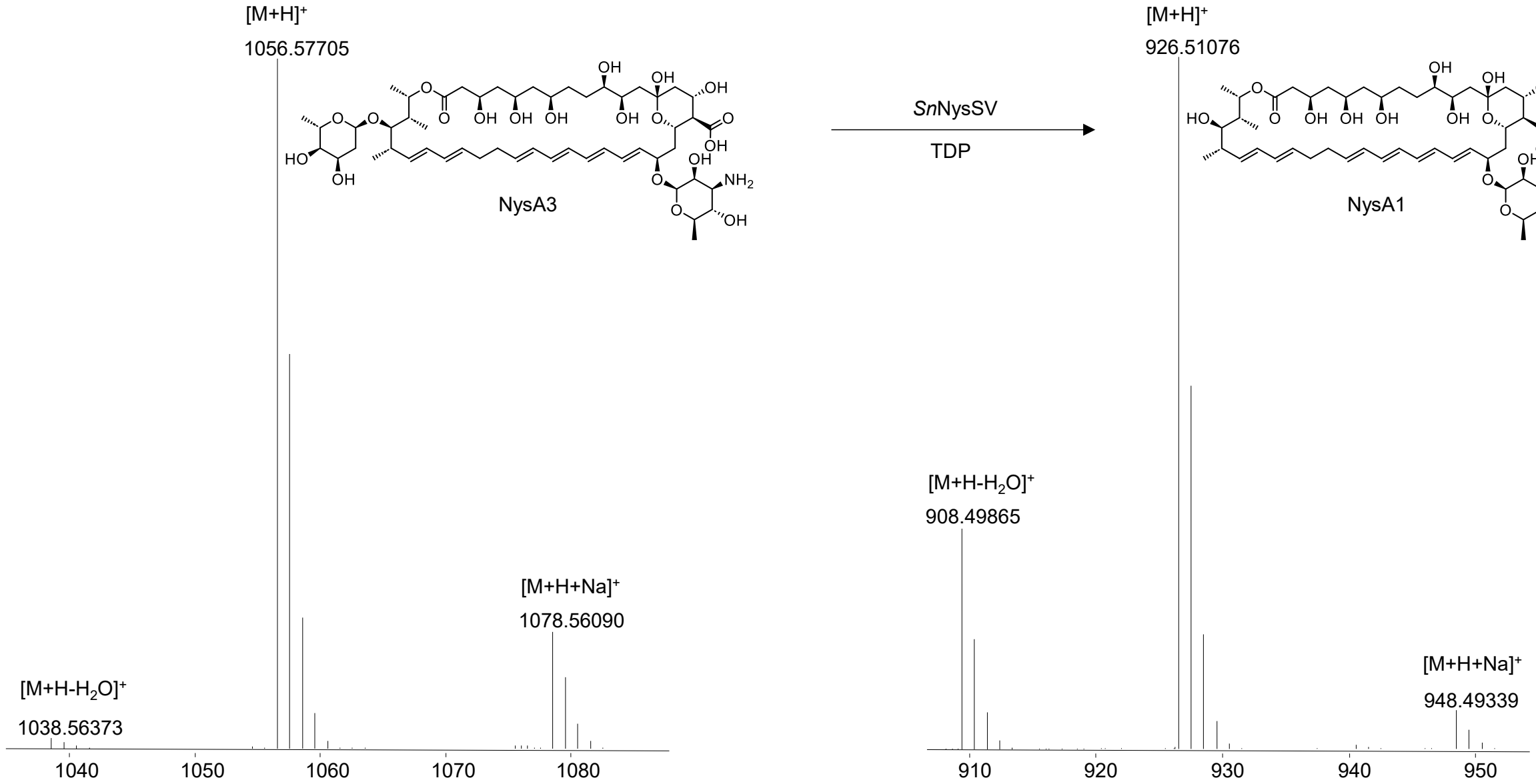

Supplementary Fig. 9. *Sn*NysSV catalysed reverse glycosylation of NysA3. The figure shows accurate mass of NysA3, calc. *m/z* = 1056.5738 $[M+H]^+$, obs. *m/z* = 1056.5770 $[M+H]^+$ and NysA1, calc. *m/z* = 926.5108 $[M+H]^+$, obs. *m/z* = 926.5107 $[M+H]^+$.

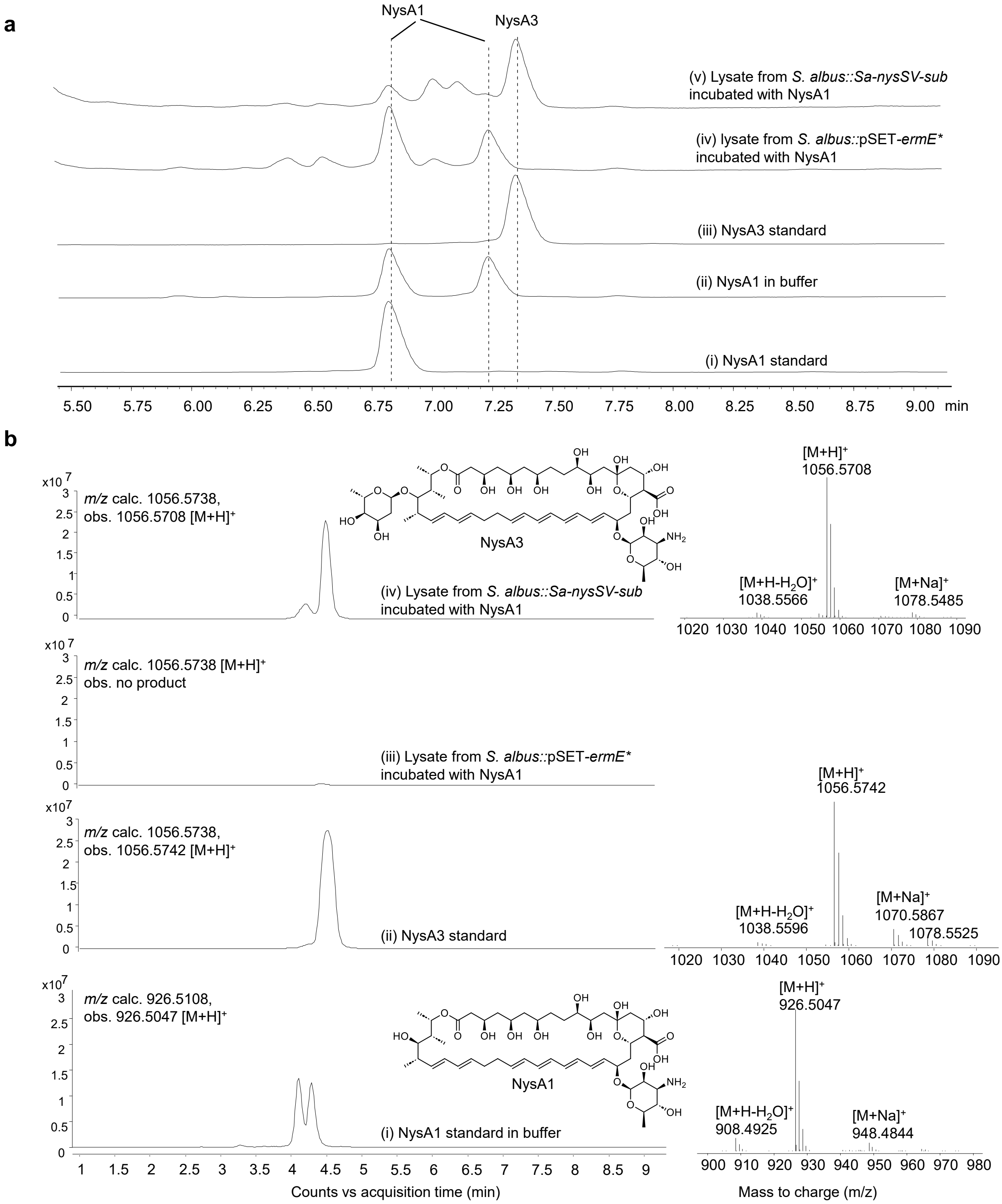

Supplementary Fig. 10. Conversion of NysA1 to NysA3 using *S. albus::Sa-nysSV-sub* lysate. (a) HPLC traces at 304 nm, (i), (ii) and (iii) show NysA1 standard , NysA1 standard in buffer and NysA3 standard, respectively, (iv) incubation of NysA1 with *S. albulus* containing empty pSET-*ermE** plasmid with no NysA3 product formation, (v) NysA1 being converted to NysA3 when incubated with lysate from *S. albulus* containing *Sa-nysSV* sugar subcluster (*S.albus::Sa-nysSV-sub).* **(b)** HRMS, (i) shows extracted ion chromatogram for NysA1 standard calculated *m/z* = 926.5108 $[M+H]^+$, observed *m/z* = 926.5047 $[M+H]^+$, (ii) extracted ion chromatogram for NysA3 standard calculated *m/z* = 1056.5738 $[M+H]^+$, observed *m/z* = 1056.5742 $[M+H]^+$, (iii) shows extracted ion chromatogram of NysA3 for the lysate assay of *S. albus::*pSET-*ermE** incubated with NysA1 with no product formed, (iv) extracted ion chromatogram of NysA3 for the lysate assay *S. albus::Sa-nysSV-sub* incubated with NysA1, *m/z* = 1056.5738 $[M+H]^+$, observed *m/z* = 1056.5708 $[M+H]^+$.

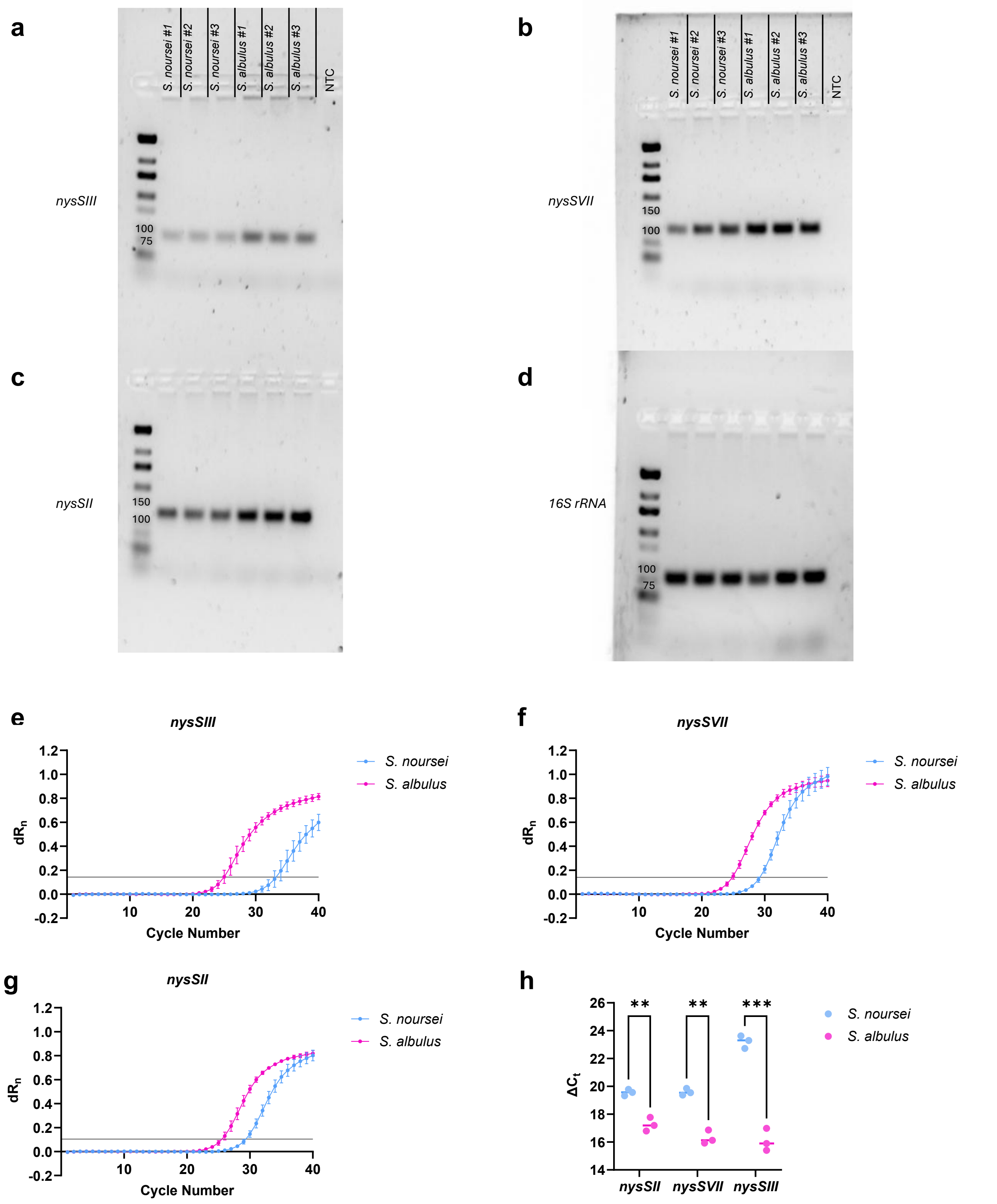

Supplementary Fig. 11. Comparison of the transcription level of genes from *SaNysSV* and *SnNysSV* sugar subclusters. (a-d) shows products of RT-qPCR assays ran on a 4% (w/v) agarose gel for **(a)** *nysSIII*, **(b)** *nysSVII*, **(c)** *nysSII* and **(d)** 16S rRNA internal control, alongside no-template controls (NTC). Expected band sizes are 84 bp, 109 bp, 124 bp and 80 bp, respectively. **(e-h)** shows amplification plots of the genes **(e)** *nysSIII*, **(f)** *nysSVII* and **(g)** *nysSII* as determined by RT-qPCR. Data displayed as means with ± SD from n = 3 biological replicates. Threshold used to determine Ct shown as a grey line. **(h)** Differential expression of *nysSV* subcluster genes. ΔCt refers to Ct (gene of interest) – Ct (16S rRNA) using 16S rRNA as an internal standard. 1 µg RNA was used as input. Data from n = 3 biological replicates shown with means as lines. ** and *** indicate statistical significance at P ≤ 0.01 and P ≤ 0.001 respectively.

a

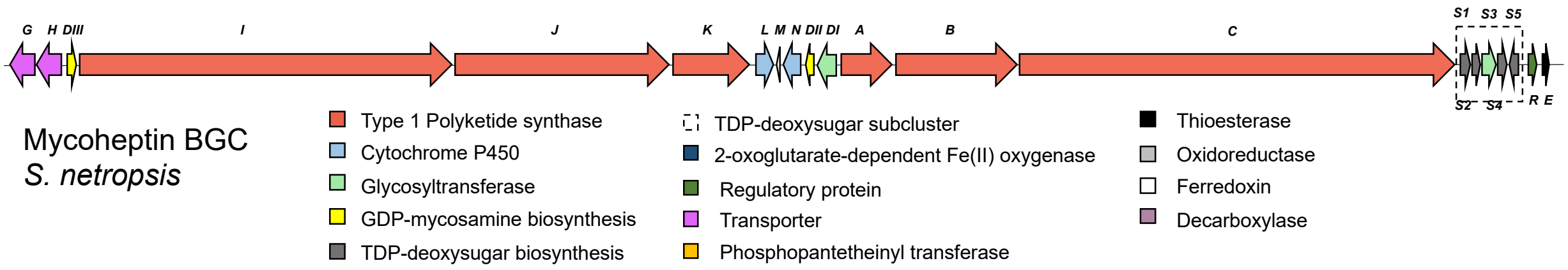

Gene	Protein function	% similarity to Nys/Sel protein
mycG	ABC Transporter	NysG, AAF71763.1 (65%)
mycH	ABC Transporter	NysH, AAF71764.1 (68%)
mycDIII	GDP-mannose 4,6 dehydratase	NysDIII, AAF71765.1 (83%)
mycI	T1 PKS (modules 9-14)	NysI, AAF71766.1 (69%)
mycJ	T1 PKS (modules 15-17)	NysJ, AAF71767.1 (65%)
mycK	T1 PKS (termination modules)	NysK, AAF71768.1 (65%)
mycL	Cytochrome P450	NysL, AAF71769.1 (86%)
mycM	Ferrodoxin	NysM, AAF71770.1 (56%)
mycN	Cytochrome P450	NysN, AAF71771.1 (79%)
mycDII	Aminotransferase	NysDII, AAF71772.1 (80%)
mycDI	Glycosyltransferase	NysDI, AAF71773.1 (78%)
mycA	T1 PKS (loading module)	NysA, AAF71774.1 (50%)
mycB	T1 PKS (modules 1-2)	NysB, AAF71775.1 (69%)
mycC	T1 PKS (modules 3-8)	NysC, AAF71776.1 (61%)
mycS1	Glucose-1-phosphate thymidylyl transferase	SelSIII, ANY10596.1 (37%)
mycS2	TDP-glucose 4,6 dehydratase	SelSIV, ANY10595.1 (61%)
mycS3	Glycosyltransferase	SelSV, ANY10594.1 (32 %)
mycS4	*O*-methyltransferase	---
mycS5	4-ketoreductase	---
mycR	LuxR Type regulator	ORF4/NysRIV (76%)
mycE	Thioesterase	NysE, AAF71777.1 (66%)

Supplementary Fig. 12. Bioinformatics analysis of mycoheptin BGC. (a) shows organisation and predicted function and homology of genes in the mycoheptin biosynthetic gene cluster. The proposed function of sugar pathway from mycoheptin BGC was inferred from the homology of its genes to those in the predicted TDP-sugar subcluster of the selvamicin producer.[23] The proposed function of L-digitoxose sugar pathway from mycoheptin BGC was inferred from the homology of its genes to those of selvamicin sugar subcluster.[23] While TDP-L-digitoxose subclusters have been observed in other biosynthetic gene clusters, their enzymes have not been fully characterised *in vitro and* remain to be tested.[65] The mycoheptin sugar biosynthetic pathway encodes for a glucose-1-phosphate thymidyltransferase (MycS1), 4,6-dehydratase (MycS2), glycosyltransferase (MycS3), *O*-methyltransferase (MycS4) and 4-ketoreductase (MycS5). However, this set of genes is incomplete for the formation of TDP-L-digitoxose, as the genes encoding 2,3-dehydratase, 3-ketoreductase and 3,5-epimerase are missing in the *mycS3* sugar subcluster. It is plausible that the missing gene functions are recruited from elsewhere in the genome. For example, in *Saccharopolyspora spinosa*, set of rhamnose biosynthesis genes for spinosad glycosylation are distributed at three different regions in the genome, and are remote (not associated) to the spinosad gene cluster.[66]

b

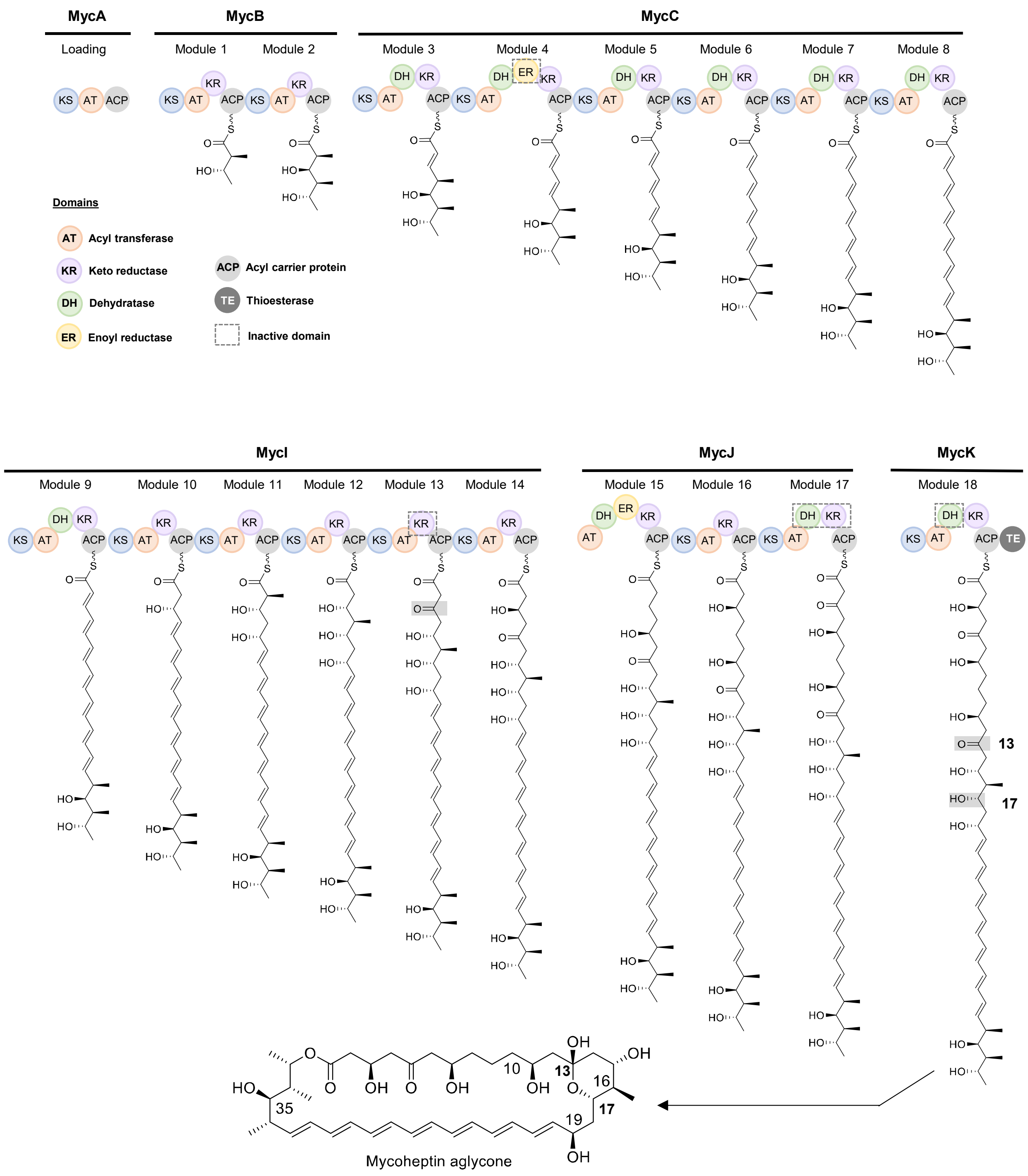

Supplementary Fig. 12. Bioinformatics analysis of mycoheptin BGC continued. (b) shows the proposed biosynthesis of the mycoheptin core macrolide. This includes 6 PKS genes comprised of 19 modules. Biosynthetically inactive domains, shown with dashed boxes were predicted based on the absence of conserved residues (Supplementary Fig. 12c). These predictions were further validated by NMR structural characterisation (Supplementary Fig. 13). For instance, NMR shows the presence of a hemiketal ring in mycoheptin and for this KR in the module 13 of PKS (MycI) must be inactive or skipped otherwise it will fail to form hemiketal ring (intramolecular cyclisation between C13 ketone and C17 OH). Similarly, predictions show the interdomain linker sequence truncations in module 4 would result in a mix of pentaene and heptaene (Supplementary Fig. 12e), validated by NMR (Supplementary Figs. 13 & 14).

c

PKS domains

AT

```
                                   Specificity motif                                     Active site
Malonyl-CoA                              HAFH                                              GHS  G
Methymalonyl-CoA                         YASH
Module12      AARFEADGRKTRLLRVSHAFHSPLMDAMLDDFRRVAEGVAYHAPAIPFVSNTTGRLATAE  YLAGHSIGEL
Module9       VARLEADGRKTKRLRVSHAFHSPLMEPMLDEFRAVVSGLTLQAPLMPFVSNLTGEPVTVA  FLAGHSIGEV
Module10      ADRLAAEGRKTRRLRVSHAFHSPRMDAMLDDFRRVAESLTYAAPAIPLVSTLTGRLATEE  YVTGHSIGEL
Module13      AGRFAADGRRTTRLRVSHAFHSPLMDPMLDAFRAVADGLTYTAPRIPVVSNLTGALADAE  FVGGHSIGEI
Module14      AGHFTDLGRKTTRLPVSHAFHSPLMDPMLDGFRRVAEELTYHKPVVPVVSNLTGETSTGE  FVGGHSIGEV
Loading       AGRLAADGRKTSRLRVTMAAHSPLMEPMLDEFRAVLEGLTFSAPHLPVVSNLTGRVAEQG  FVAGHSIGEV
Module18      AARLSAQGRKTSRLSISIASHSPLMDAVLDDFRAVVRGLSPEAPAIPVVSHLTGDVAVAE  FVAGHSIGEV
Module5       ASRLAADGRKTSRLSVSHAFHSPLMEPMLEDFRAVVDSLSFAEATVPVVSNLTGRPATSQ  FLAGHSIGEL
Module17      AAKLAAEGRKTSRLSVSHAFHSPLMEPMLEDFHEVVSGLSFSAPSVPVVSNLTGRLATSE  YVAGHSIGEV
Module16      AGRLAAEGRKTSRLSVSHAFHSPLMEPMLEDFHAVVSGLSFSAPSVPVVSNLTGRLATSE  FLAGHSIGEV
Module6       AGRLSADGRKTSRLSVSHAFHSPLMEPMLDDFRAVVEGLVFAEPSVPVVSNLTGRVAAAQ  FVAGHSIGEV
Module4       AGRLSAEGRKTSRLAVSHAFHSPLMEPMLEDFRAVVEGLAFAEPSVPVVSNLTGRLATSE  FLAGHSIGEI
Module7       AGRLSAEGRKTSRLSVSHAFHSPLMEPMLEDFRAVVEGLAFAEPSVPVVSNLTGRVAAAE  FLAGHSIGEV
Module8       AAKLAAEGRKTSRLSVSHAFHSPLMEPMLEDFRAVVDGLTFNAPSVPVVSNLTGRIATSE  FVAGHSIGEI
Module3       AGRLAADGRKTSRLSVSHAFHSPLMEPMLDDFRTVVTGLTFQAPSIPVMSNLTGRMATSE  FVAGHSIGEI
Module15      AARLAADGRKTSRLSVSHAFHSPLMEPMLDDFRTVVTGLAFTEPAIPVVSTLTGALAGIG  FVAGHSIGEI
Module2       KDQCATEKIRARTVAVDYASHGSQVELIQDELAHLLASVTPRAPKVPFYSTVTGERLDTP  AVMGHSQGEI
Module1       TARLTDEGVRVRRIAVDYASHSAQVEDLHEELLSELGPIRPRASDVPFLSTVTGAWLDTS  AVVGHSQGEI
Module11      TARLTDEGVRVRRIAVDYASHSAQVEDLHEELLSELGPIRPRASDVPFLSTVTGAWLDTS  AVVGHSQGEI
```

KR

```
                                    S              Y   N
Module1       ARHLDELLDDEELDAFVLFSSITGMWGSGRHAAYGAGNAYLSALADHRRARGATATSLHW
Module11      AQNLDELLDDAELDAFVLFSSAAGMWGSGQHAAYVAGNAFVSALAENRRARGVTATSLHW
Module13      VTTLDAALGDRPLDAFVLFSSIAGVWGVRGQGDGAAACALLDAFATRRRARGATALTVSW
Module17      AWHLHEATREMNLSAFVLCSSVSGVLGSPERAGTSAADAFLDALAQYRRNLGLAGMSVAW
Module18      AWHLHEATRGLDLSAFVLYSSVSGVLGAPGQGNYAAANAFLDALVRHRRGLGLPGTSLAW
Module4       AWHLHELTRDLDLAAFVTFSSVSGTFGGAGQGNYAAGNAFLDALAHHRAAQGLPTVSLAW
Module15      AWHLHELTRDLDLAAFVLYSSTAGVMGSPGQGNYAAGNTFLDALAHHRHSQGLPATSLAW
Module5       AWNLHELTRDKNLSAFVVFSSMSGLLGGAGQGNYAAGNAFLDALMEHRRAEGLPGVSLAW
Module3       VWNLHELTRDADLTAFAVFSSVSATLGSAGQGNYAAGNAFLDALAAHRRAAGLPGLSLAW
Module8       VWNLHELTRDLDLSAFVVFSSVAGTFGGAGQGNYAAGNAFLDALVERRHAEGLAGVSLVW
Module9       VWNLHELTRDADLTAFVVFSSVAGTFGGAGQGNYAAGNAFLDALVQRRREEGLPGVSLVW
Module6       AWNLHELTRDLGLSAFVVFSSVAGTFGGAGQANYAAGNAFLDALVQRRRQEGMPGISLVW
Module7       VWNLHELTRDLDLAAFVVFSSVAGTFGGAGQGNYAAGNAFLDALVQRRRQEGLPGVSLVW
Module2       ALHLHEATSDLDLDAFVLFSSGAGIWGSGGQAGYAAANAFLDTLAEHRRARGLTATAVSW
Module14      AFLLDELTRELDLSVFVLFSSASAAVGNPGQANYAAANAVLDALAEHRRADGFPATSIAW
Module16      ALLLDELTRELDLSVFALFSSIAGAVGNPGQANYAAANVVLDTLAERRRAAGLPATSIAW
Module10      AANLHALLGDRELDLFVLFGSIAGVWGSGGQSAYGAANAYLDALAEHRRAHGLAATCVAW
Module12      ARHLHELTQDTALSAFVLFSSTAGTLGAAGQGNYAAANAYLDALAEHRRAAGLTATSLAW
```

DH

```
                                                 H   G    P                  Y
Module17      HPMLGAAVIMAGSDEVVLTGRLSLATHPWLADHQVGGAVSLPGAGFAELAV    GVAHGP
Module9       HPLLSAAVSLADSDGALLTGRLSLRSHPWLADHAVRGTVLLPGTAFLELAI    GFGYGT
Module5       HPLLNGAVELADGEGVLFTGRLSLQSHPWLADHAVLGRVLLPGTALLELAI    GFAYGP
Module6       HPLLNGSVELAVGEGVLFTSRLSVRSHPWLADHVVMGRVLLPGTAFVELAI    GFQYGP
Module8       HPLLGAAVELAGGEGVLFTGRLSVQSHPWLADHVVMGRVLLPGTALVELAI    GFAYGP
Module3       HPLLGAAVELANGEGALFTSRLSVQSHPWLADHVVQGRVLLPGTAFVELAI    GFDYGP
Module4       HPLLGAAVELADGEGALFTNRLSVRSHPWLADHAVMGRVLLPGTAFVELAI    GFAYGP
Module7       HPLLGAAVELAGGEGVLFTGRLSVQSHPWLADHVVMGRVLLPGAALVELAI    GFVYGP
Module15      HPLLGATMVMAGSDELVLTGTLSLRTHPWLGDHVVSGMVFFPGTGFLELAI    GLDYGP
Module18      HPLLGAAMSVAGSDELVLTGTLSAATHPWLADHRVMGRITLPGTAFVELAV    GLAYGP
```

ER

```
                                                               K          D
Module4       SRTTDFEAEFAAVAGDGGIDVVLNSLAGEFVDASLRLLPPSGRFIEMGKTDIRD    VSFDLGWV
Module15      SRTTDFEHAFAAVAGDGGIDVVLNALSGEFVDASLRLLAPGGRFLEMGKTDIRD    RSFDLGFA
```

Supplementary Fig. 12. Bioinformatics analysis of mycoheptin BGC continued. (c) shows predicted activity and selectivity of the mycoheptin PKS. AT domains: Acyl-CoA specificity of AT domains analysed by comparison of specificity motif to the commonly seen malonyl-CoA HAFH motif (blue) and methylmalonyl-CoA YASH motif (green). Divergences from this sequence are highlighted in purple. KR, DH and ER domains: Conserved residues associated with active domains are shown in bold. Variations from these residues are shown in red and suggest catalytic inactivity.

d

Mycoheptin	PKS module	Mycoheptin	NysA1
MycA	Loading	KS-AT-ACP	KS-AT-DH-ACP
MycB	1	KS-AT(mmal)-KR-ACP	KS-AT(mmal)-KR-ACP
	2	KS-AT(mmal)-KR-ACP	KS-AT(mmal)-KR-ACP
MycC	3	KS-AT(mal)-DH-KR-ACP	KS-AT(mal)-DH-KR-ACP
	4	KS-AT(mal)-DH-ER-KR-ACP	KS-AT(mal)-DH-KR-ACP
	5	KS-AT(mal)-DH-KR-ACP	KS-AT(mal)-DH-ER-KR-ACP
	6	KS-AT(mal)-DH-KR-ACP	KS-AT(mal)-DH-KR-ACP
	7	KS-AT(mal)-DH-KR-ACP	KS-AT(mal)-DH-KR-ACP
	8	KS-AT(mal)-DH-KR-ACP	KS-AT(mal)-DH-KR-ACP
MycI	9	KS-AT(mal)-DH-KR-ACP	KS-AT(mal)-DH-KR-ACP
	10	KS-AT(mal)-KR-ACP	KS-AT(mal)-KR-ACP
	11	KS-AT(mmal)-KR-ACP	KS-AT(mmal)-KR-ACP
	12	KS-AT(mal)-KR-ACP	KS-AT(mal)-KR-ACP
	13	KS-AT(mal)-KR-ACP	KS-AT(mal)-KR-ACP
	14	KS-AT(mal)-KR-ACP	KS-AT(mal)-KR-ACP
MycJ	15	KS-AT(mal)-DH-ER-KR-ACP	KS-AT(mal)-DH-ER-KR-ACP
	16	KS-AT(mal)-KR-ACP	KS-AT(mal)-KR-ACP
	17	KS-AT(mal)-DH-KR-ACP	KS-AT(mal)-DH-KR-ACP
MycK	18	KS-AT(mal)-DH-KR-ACP-TE	KS-AT(mal)-DH-KR-ACP-TE

Supplementary Fig. 12. Bioinformatics analysis of mycoheptin BGC continued. (d) shows PKS module comparison between mycoheptin and NysA1. Catalytically inactive domains shown in red. mal = malonyl-CoA, mmal = methylmalonyl-CoA.

e

Dehydratase domain (DH)

```
MycC   ...RIEDLALAAPLVLPERGAVQLQVRVGGADDAGRRSVGVYSRIEGAVDVPWTQHAMGVLVP   120
NysC      RVEELTLAAPLVLPERGAVQTQVRVGVADDTGRRTVTVHSRPEHATDVSWTQHATGTLTM   120
AmphC     RVEELTLAAPLVLPERGAVQTQVRVGVADDTGRRTVTVHSRPEDATDVSWTQHATGTLMP   120

MycC      GTDAADAAFDATVWPPAGAEPVDLTGFYEARAEDGFAYGPEFQGLRAAWLRDGEVFAEVG   180
NysC      GSAPADTGFDATAWPPADAEPLATDDCYARFTTLGFAYGPVFQGLRAAWRAGDVLYAEVA   180
AmphC     GGTARDTGFDATVWPPTGAEALDTEGCYERFTALGFGYGPVFQGLKAAWRSGDVLYAEVV   180

MycC      LPDAA-QAAPDAFGLHPALLDAVLHAGGLLG---SERQAVPFSWEGVSLHASGASAVRVR   236
NysC      LAEST-GDEATAFGLHPALLDAALHASLVAHEGEESNGGLPFSWEGATLYATGATALRVR   239
AmphC     LQEAGAADDEAGFGLHPALLDAALHASLVAHEGEEGNGGLPFAWEGAVLHATGATALRVR   240

MycC      LSRAAD--GALAIDIADTSGAPVATVDSLLAREISADQLAEADTRDRDSLFRLEWSPVAA   294
NysC      LTPTGTDGRSVAIAVADTAGRPVAAIDNLVSRRVSGDQLTGAAGLARDALFTLDWNPVPE   299
AmphC     LTPTDEARQSVAIAVCDTAGQPVASIDRLLVRAVTGDQLNEAAGIARDALFALGWTPLPQ   300

MycC      DIA---------------------VVGPVAVVGTDVLGLTEALRAADVDVTVHPDLGALA   333
NysC      NLVPENPVPENTGGGHAQDQDGRPAAATVALVGADGTAIAADLTAAGIHTTLHPDLTTLA   359
AmphC     DGD---------------------IPAAVALVGPDTTGLAADLTAAGAAVTAHQDLAALA   339

MycC      ASDGSVPGVVLVPVVGDADA-----------------------TVIDATHEATALALGRL   370
NysC      TTDADVPKTVLIPLTGTGTGTGTGTESTDGIGTGAAESDASAPSPAEVAHTLSTAALALV   419
AmphC     ADGTDIPGTVVVTVDGTA------------------------APVAEAAHTVAAEALALA   375

MycC      QQWLADERFGGSRLVFVTRGAVAVDAGGVADLAAASVWGMVRSAQTENPGCFGLVDLDAS   430
NysC      QEWTAQERFAGSRLAFVTTGATAAGGTDVMDVAAAAVWGLVRSAQSEAPDTFVLIDRDPG   479
AmphC     QRWTAEDRFAGARLVFATCGAVEAGGSQVTDVAAAAVWGLVRSAQSESPDTFVLVDREAA   435

MycC      VSS----------AAGLPGALGTDEPQVIVRDDELMAGRLARTASSGGLVPPADGPWRLG   480
NysC      PAGTHDRTAAAERGQLLLRALHTDEPQLALRDGGVLAARLARFDTAAALTPPADRAWRLD   539
AmphC     DAA----GDAAERGSLFLAALGSGEPQLALRDDVVLAGRLARFDAAGAMTLPAERAWHLD   491

MycC      SRAKGSLGGLELAPCPEVLEPLTGRDVRIGVRAAGLNFRDVLNALDMYPGD-AGVFGSES   539
NysC      STAKGSLNGLALTPYPAALAPLTGHEVRVEVRAAGLNFRDVLNALGMYPGDDVGSFGSEA   599
AmphC     STTPGSINGLALTPHPEALEPLTGHQARIEVRAAGLNFRDVLKALGMYPGD-AGRYGHEA   550

MycC      SGVVLEVGPDVTGLRPGDRVMGVVVGGFGSLVVADERRLVRVPDAWSWETAASVPLVFLT   599
NysC      AGVVVEVGPEVTGLAPGDQVMGMITGSFGSLAVDDARRLARLPEDWSWETGASVPLVFLT   659
AmphC     AGVVVEVGPDVTHIAPGDRVMGMVSGSFASFAVTDARRLT-HPEQCTWEIGASLPLVFLT...  609
```

Interdomian linker sequence truncations (-------)

Enoyl reductase domain (ER)

Supplementary Fig. 12. Bioinformatics analysis of mycoheptin BGC continued. (e) shows alignment of DH-linker-ER regions of MycC, NysC and AmphC. Alignment of the DH (blue)-linker (black)-ER (gold) regions from module 4 of MycC and module 5 of NysC and AmphC. The *myc* PKS has a truncated linker region between the dehydratase (DH) and enoyl reductase (ER) domains of module 4, which, based on earlier observations,[67] may cause the ER domain to be partially skipped. This would result in a mix of pentaene and heptaene products with a C30-31 single and double bond respectively.

a

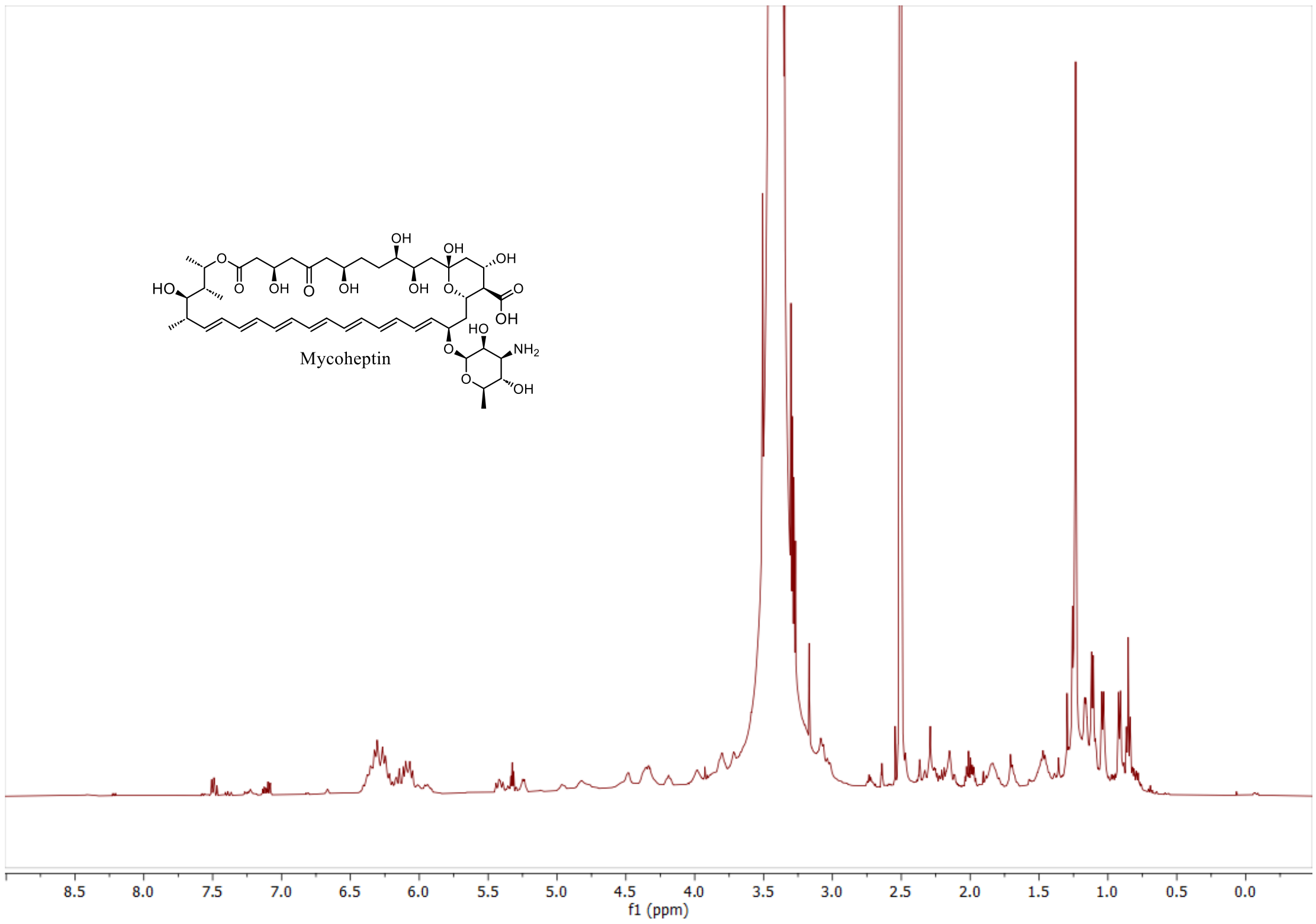

b

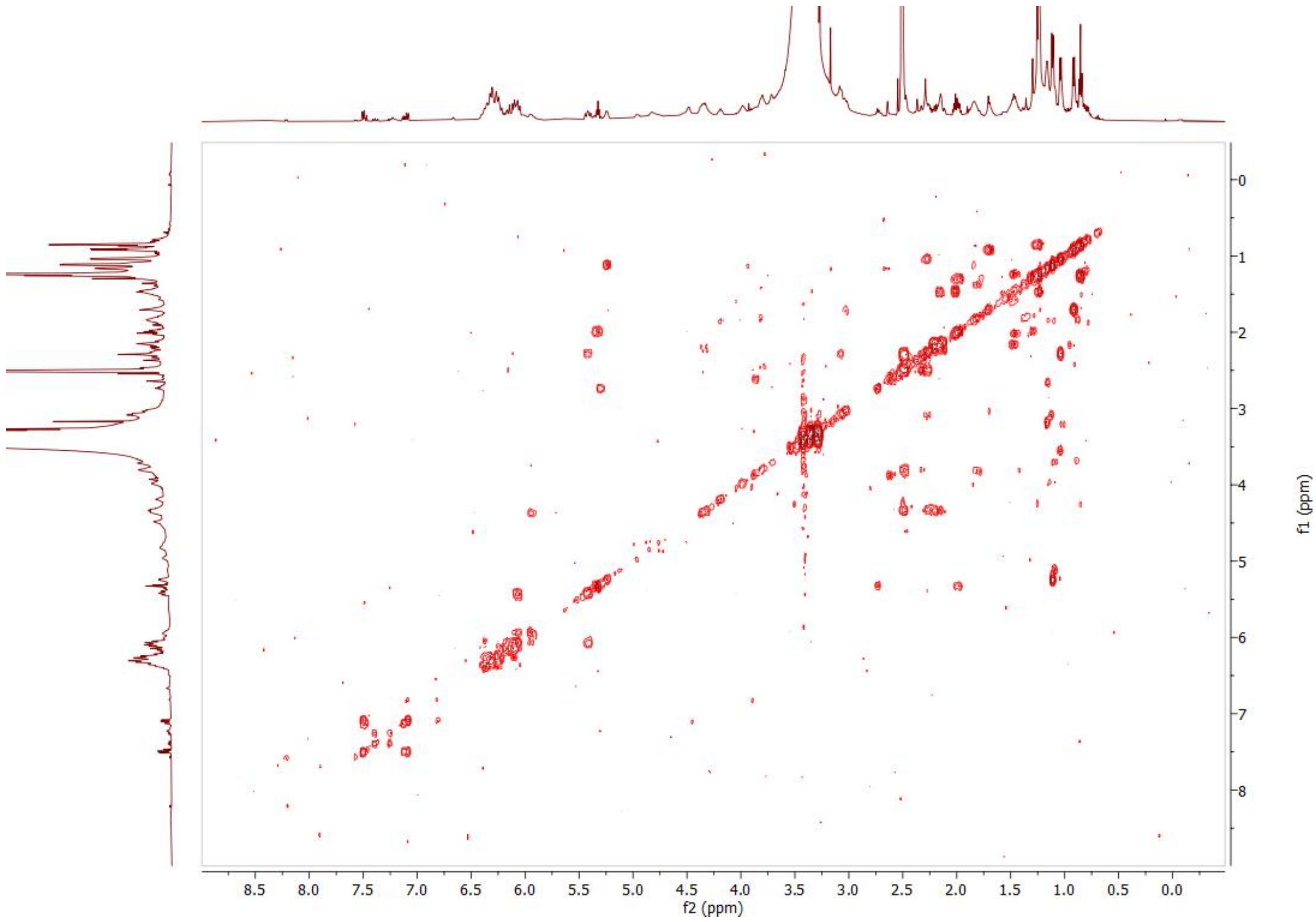

Supplementary Fig. 13. NMR data for mycoheptin. (a) shows ^{1}H NMR spectrum of mycoheptin in DMSO-*d6.* **(b)** shows ^{1}H-^{1}H COSY NMR of mycoheptin in DMSO-*d6*.

c

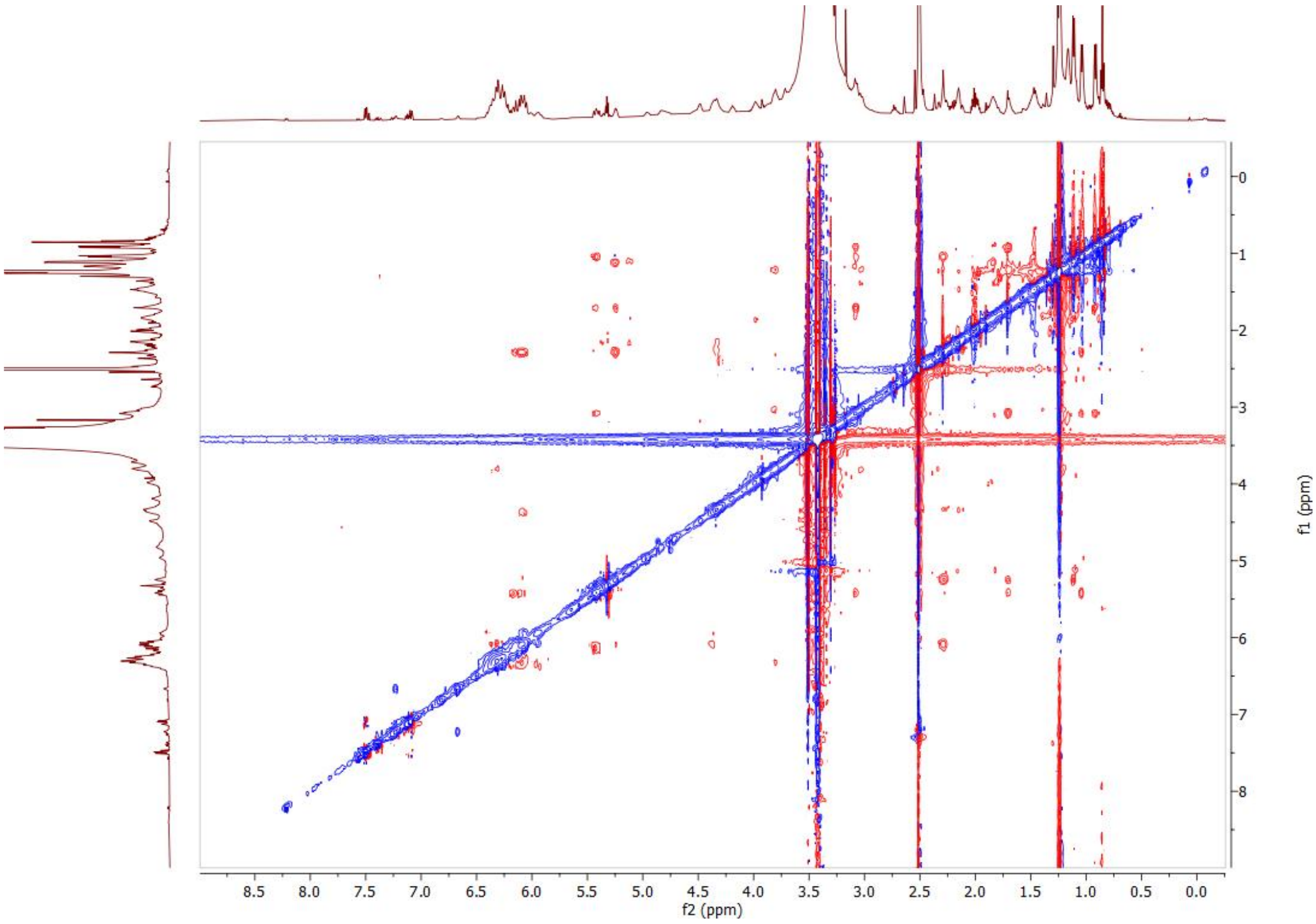

d

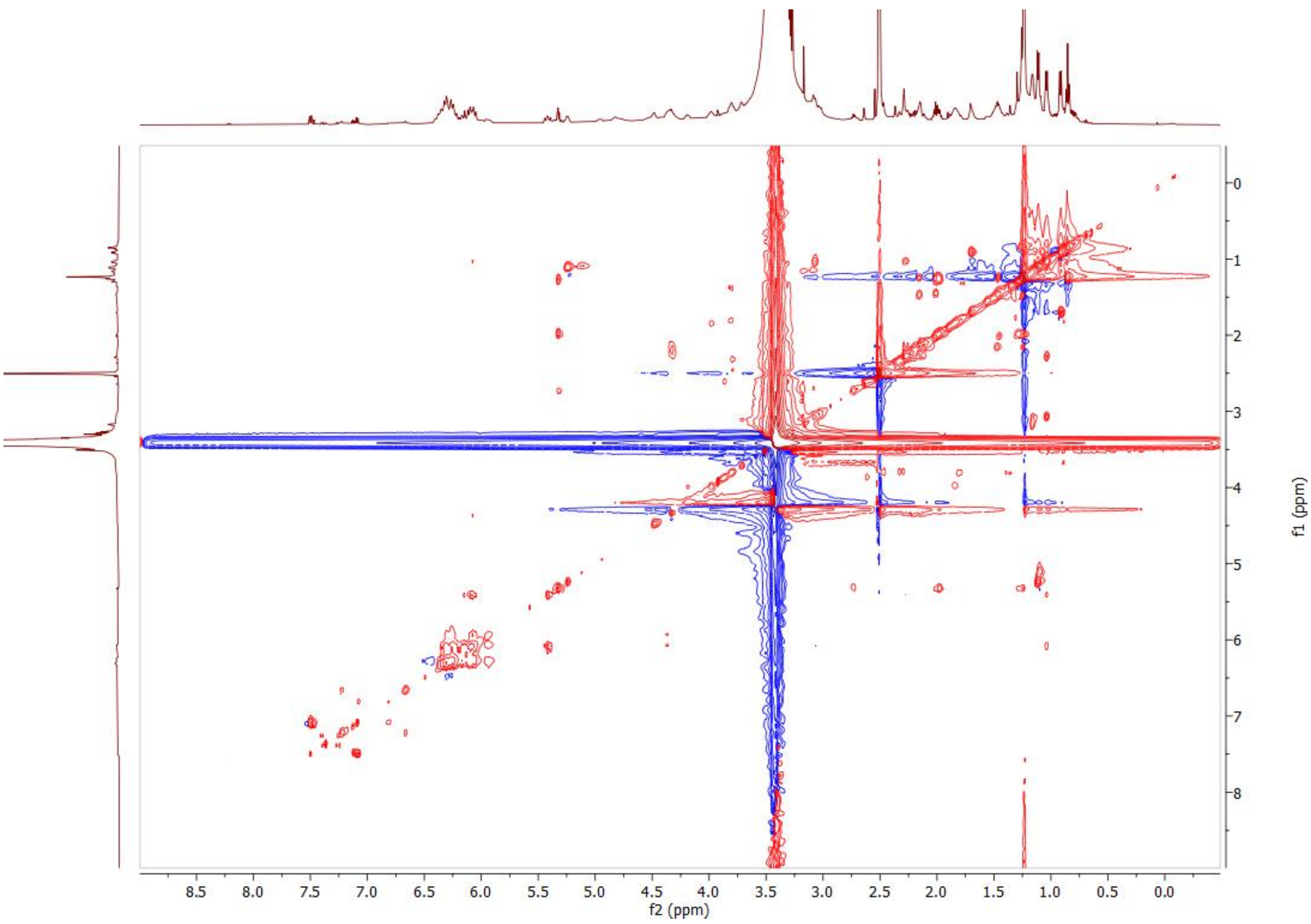

Supplementary Fig. 13. NMR data for mycoheptin continued. (c) ^{1}H-^{1}H ROESY NMR of mycoheptin in DMSO-*d6.* **(d)** ^{1}H-^{1}H TOCSY NMR of mycoheptin in DMSO-*d6*.

e

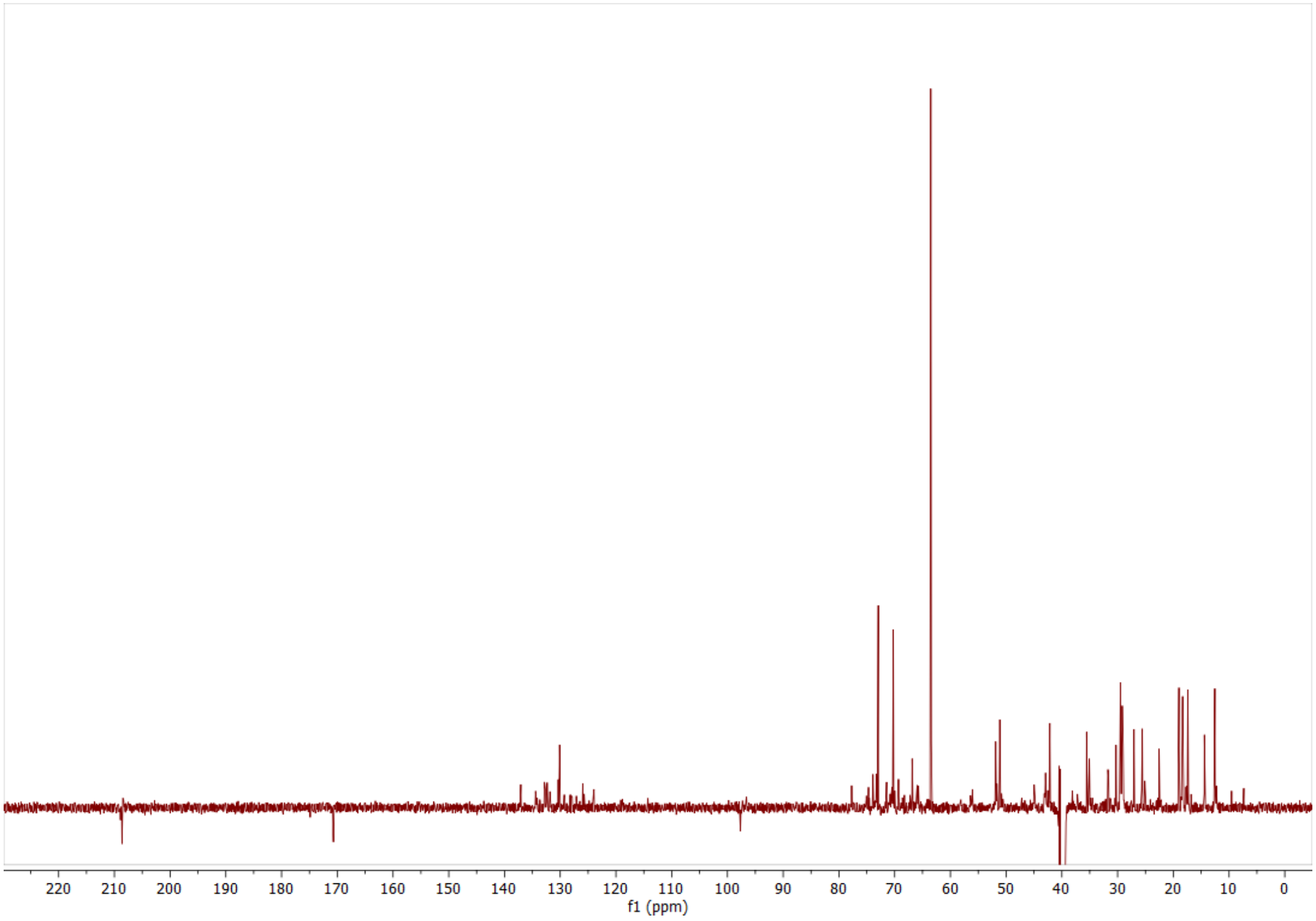

f

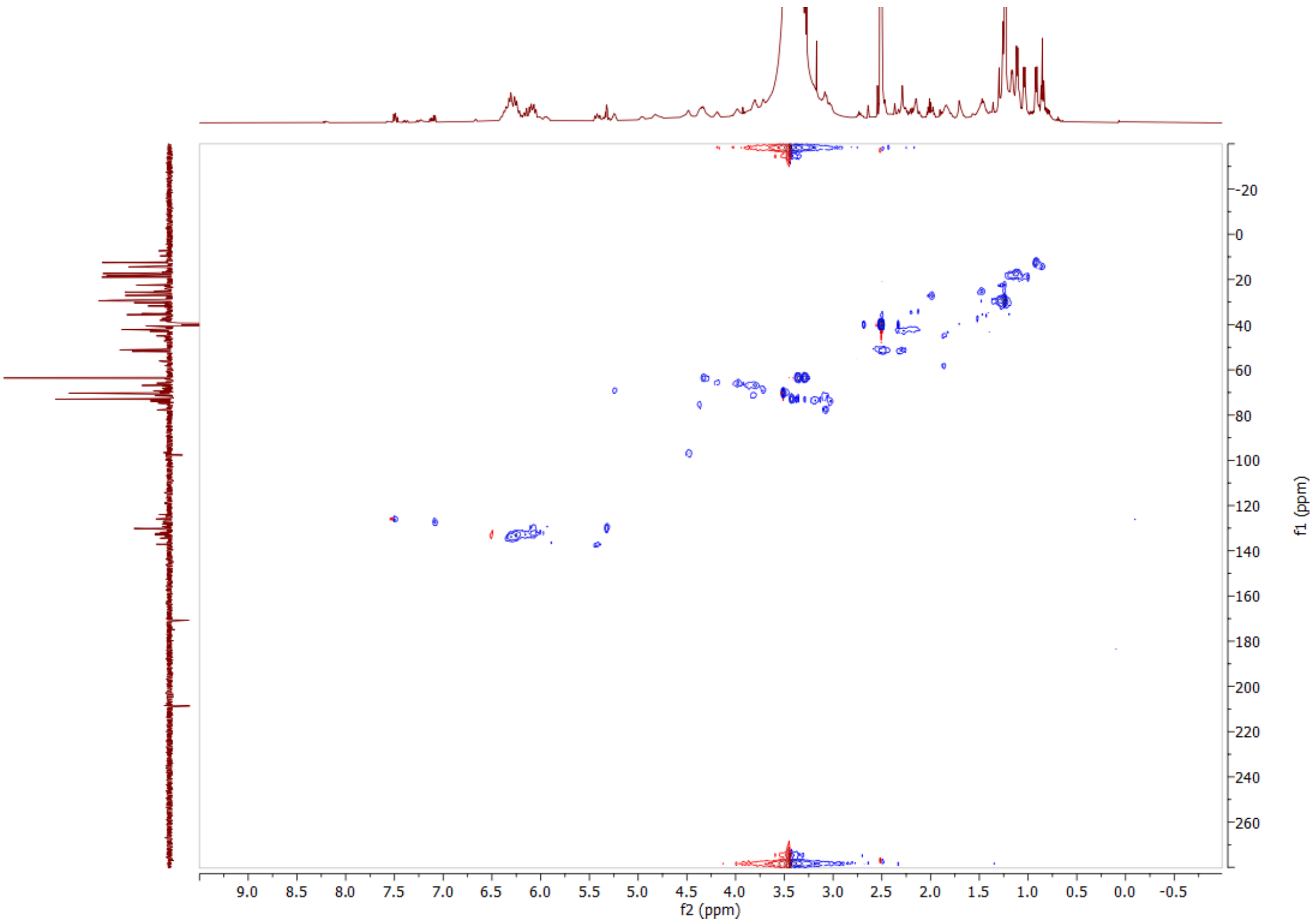

Supplementary Fig. 13. NMR data for mycoheptin continued. (e) ^{13}C DEPTQ, positive multiplicity edited NMR of mycoheptin in DMSO-*d6*. **(f)** ^{1}H-^{13}C- HSQC NMR of mycoheptin in DMSO-*d6*.

g

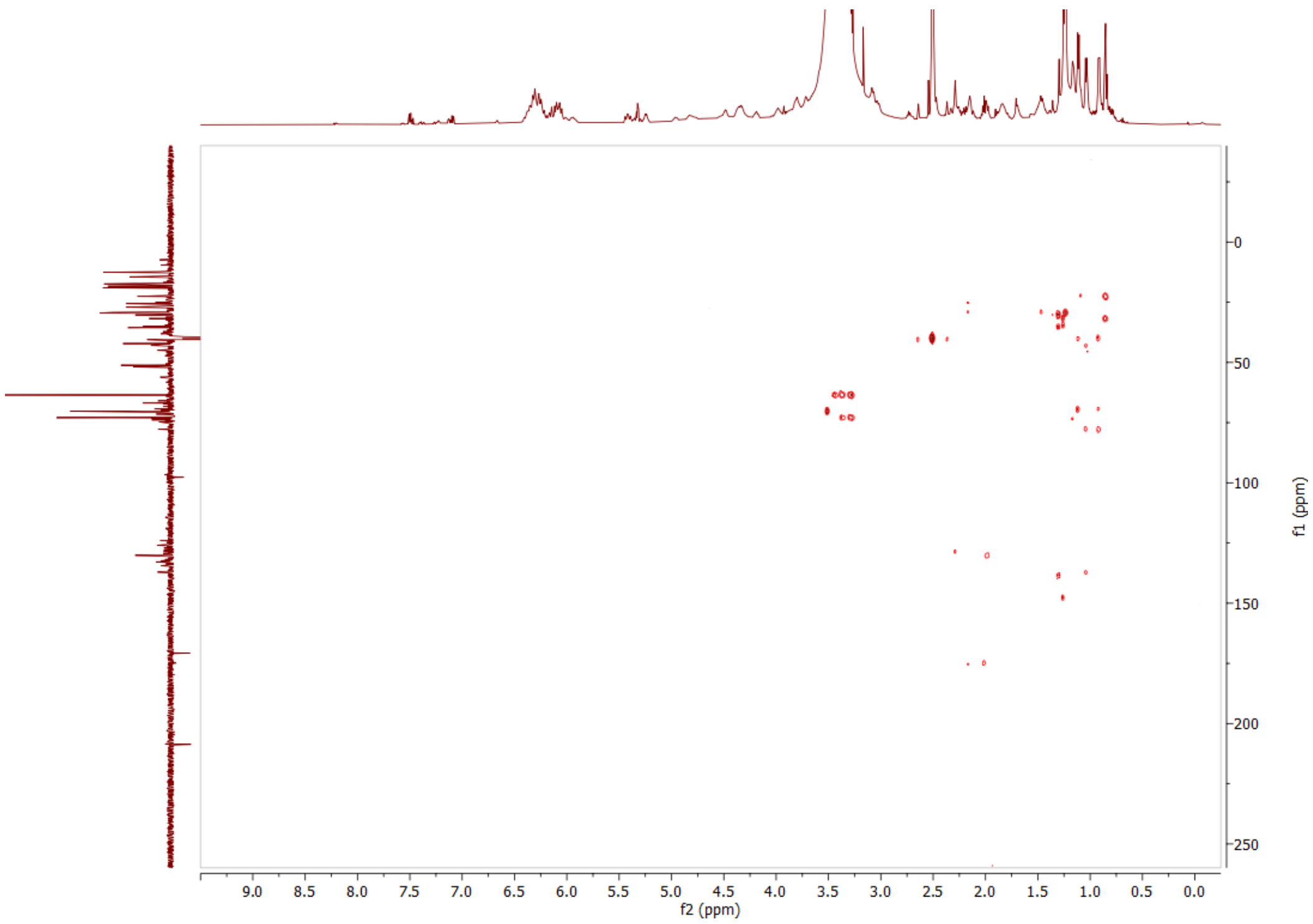

Supplementary Fig. 13. NMR data for mycoheptin continued. (g) shows ^{1}H-^{13}C- HMBC NMR of mycoheptin in DMSO-*d6*.

h

Position	^{1}H found mycoheptin in DMSO-*d6*	^{13}C found mycoheptin in DMSO-*d6*	Position	^{1}H found mycoheptin in DMSO-*d6*	^{13}C found mycoheptin in DMSO-*d6*
1		170.73	32	5.94-6.40	129.02-134.41
2	2.49, 2.67	41.00	33	5.43	137.03
3	4.30	63.84	34	2.49	38.44
4	2.43, 2.54	50.94	35	3.16	85.02
5		208.63	36	1.92	38.73
6	2.30; 2.48	51.34	37	5.24	69.31
7	3.95	66.82	16-COOH		174.94
8	1.39; 1.44	35.74	34-CH_3	0.92	13.13
9	1.35; 1.48	28.82	36-CH_3	0.85	14.71
10	3.81	67.04	37-CH_3	1.05	19.23
11	4.00	66.32	1′	4.47	96.92
12	1.15; 1.85	45.12	2′	3.73	68.74
13		97.74	3′	2.76	56.73
14	1.36; 1.82	42,62	4′	3.18	73.32
15	3.96	65.91	5′	3.27	71.91
16	1.84	58.24	6′	1.17	17.83
17	4.05	65.53			
18	1.71; 2.30	39.22			
19	4.36	75.14			
20	5.41	137.03			
21	6.06	129.31			
22	5.94-6.40	129.02-134.41			
23	5.94-6.40	129.02-134.41			
24	5.94-6.40	129.02-134.41			
25	5.94-6.40	129.02-134.41			
26	5.94-6.40	129.02-134.41			
27	5.94-6.40	129.02-134.41			
28	5.94-6.40	129.02-134.41			
29	5.94-6.40	129.02-134.41			
30	5.94-6.40	129.02-134.41			
31	5.94-6.40	129.02-134.41			

Supplementary Fig. 13. NMR data for mycoheptin continued. (h) shows ^{1}H and ^{13}C NMR spectral data for mycoheptin in DMSO-*d6*.

a

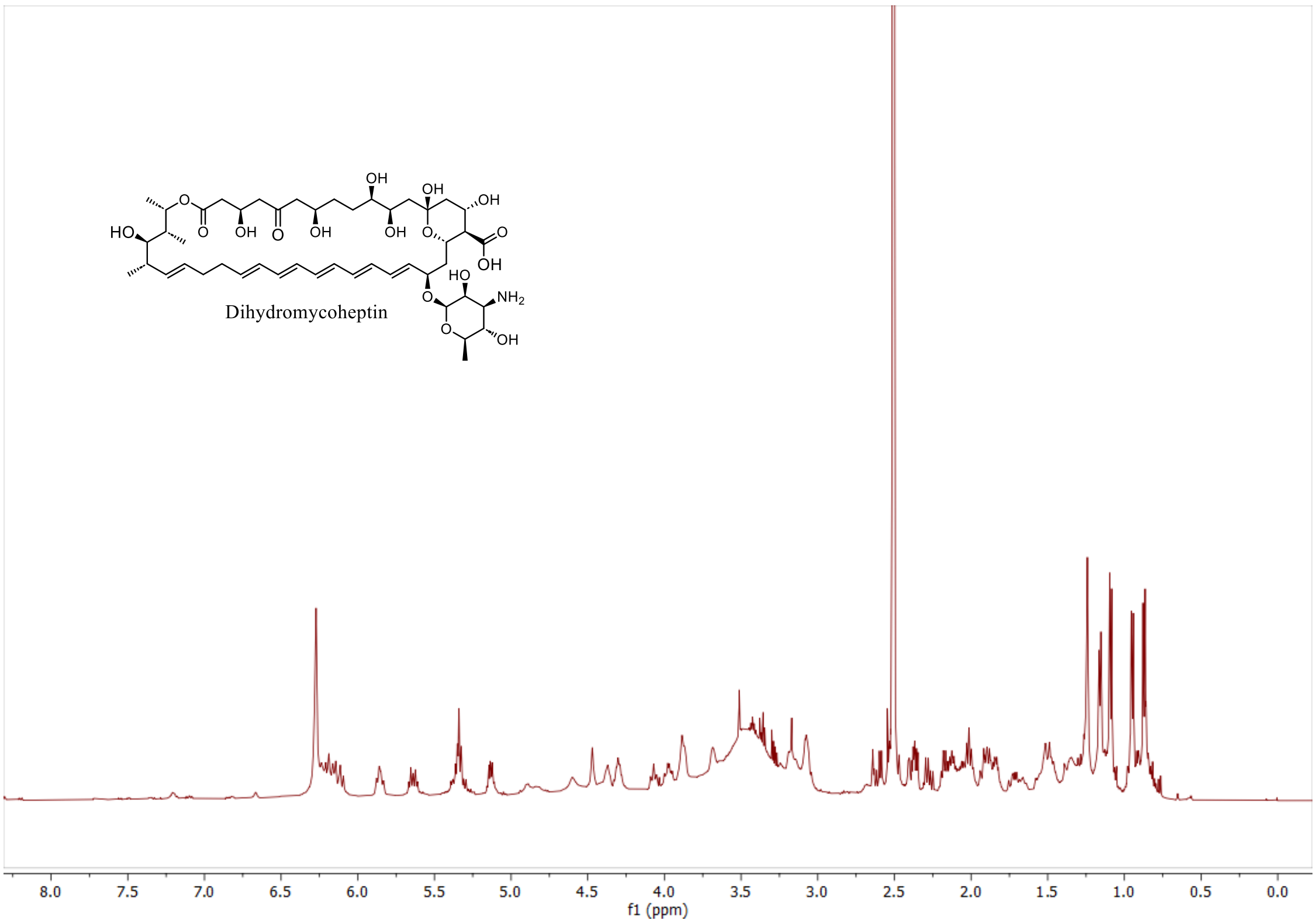

b

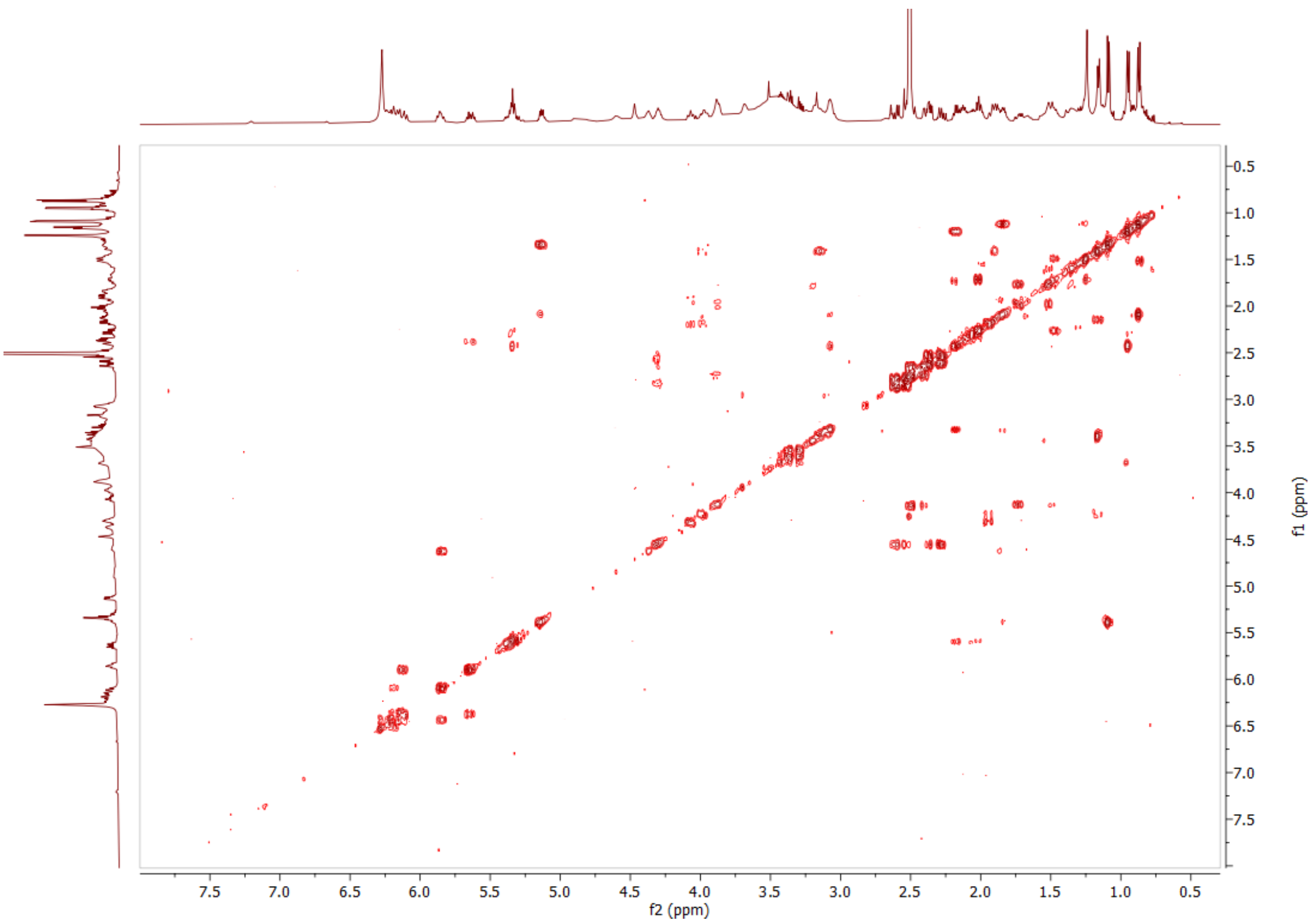

Supplementary Fig. 14. NMR data for dihydromycoheptin. (a) shows ^{1}H NMR spectrum of dihydromycoheptin in DMSO-*d6*. **(b)** shows ^{1}H-^{1}H COSY NMR of dihydromycoheptin in DMSO-*d6*.

c

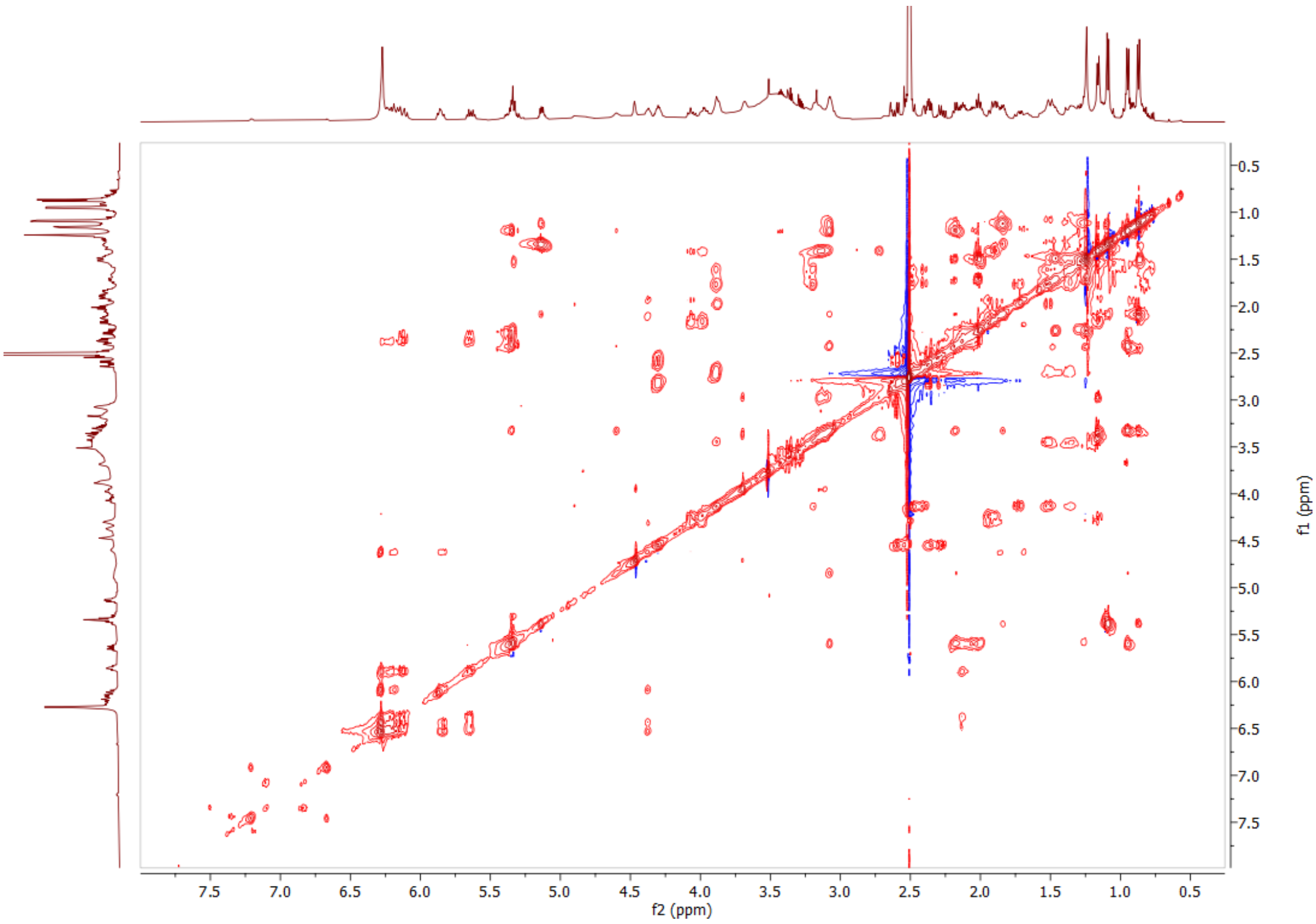

d

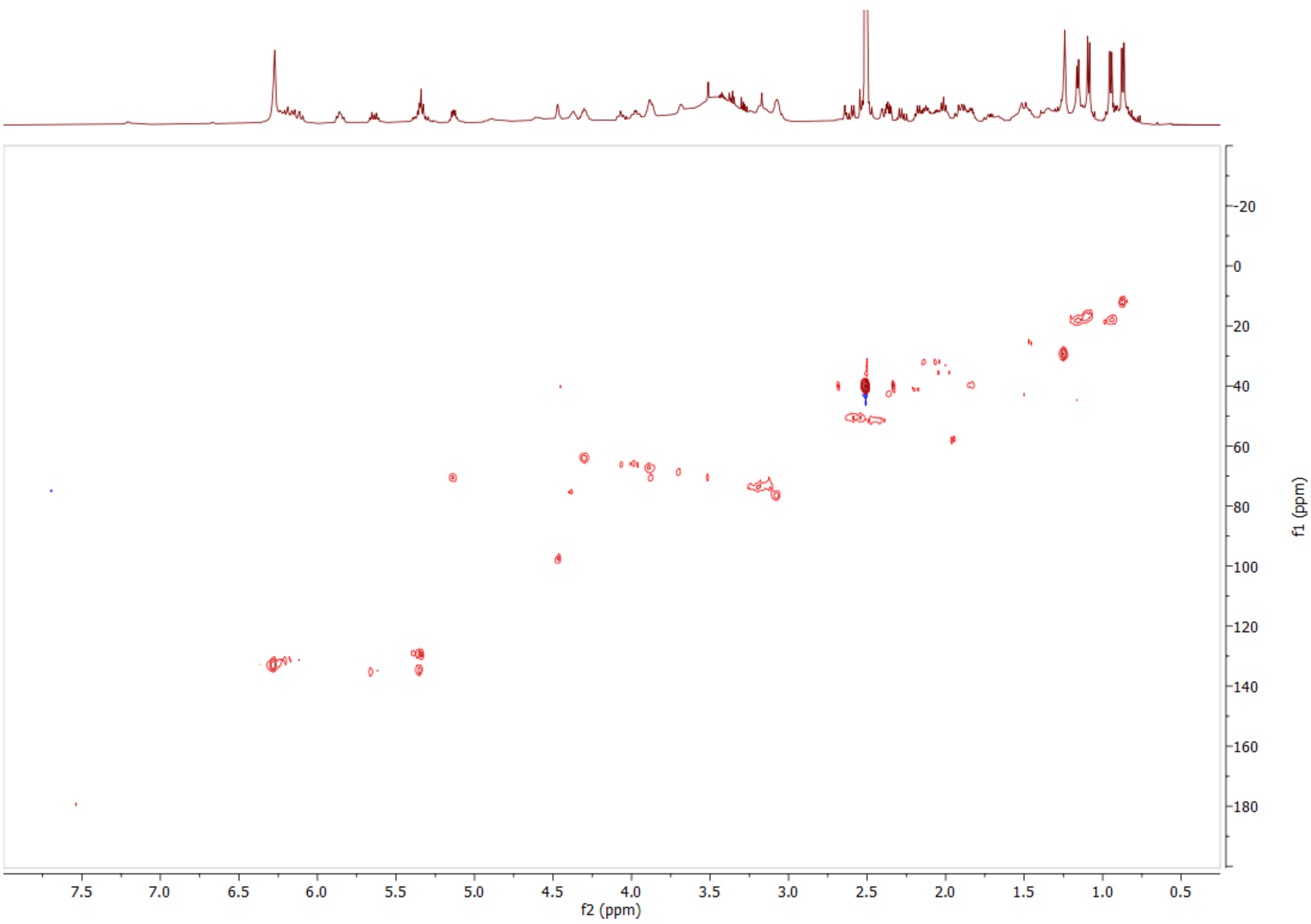

Supplementary Fig. 14. NMR data for dihydromycoheptin continued. (c) shows ^{1}H-^{1}H TOCSY NMR of dihydromycoheptin congener in DMSO-*d6*. **(d)** shows ^{1}H-^{13}C- HSQC NMR of dihydromycoheptin congener in DMSO-*d6*.

e

Position	^{1}H found dihydromycoheptin congener in DMSO-*d6*	Position	^{1}H found dihydromycoheptin congener in DMSO-*d6*
1		32	5.33
2	2.27, 2.35	33	5.34
3	4.30	34	2.34
4	2.54, 2.59	35	3.27
5		36	1.92
6	2.39; 2.47	37	5.13
7	3.86	34-CH_3	0.94
8	1.33; 1.48	36-CH_3	0.86
9	1.37; 1.49	37-CH_3	1.07
10	3.88	1′	4.47
11	4.03	2′	3.67
12	1.15; 1.87	3′	2.62
13		4′	3.18
14	1.50; 1.71	5′	3.27
15	3.96	6′	1.17
16	1.89		
17	4.05		
18	1.67; 1.91		
19	4.36		
20	5.85		
21	6.15		
22	6.05-6.32		
23	6.05-6.32		
24	6.05-6.32		
25	6.05-6.32		
26	6.05-6.32		
27	6.05-6.32		
28	6.11		
29	5.63		
30	2.16		
31	2.03; 2.15		

Supplementary Fig. 14. NMR data for dihydromycoheptin continued. (e) shows ^{1}H NMR spectral data for dihydromycoheptin congener in DMSO-*d6*

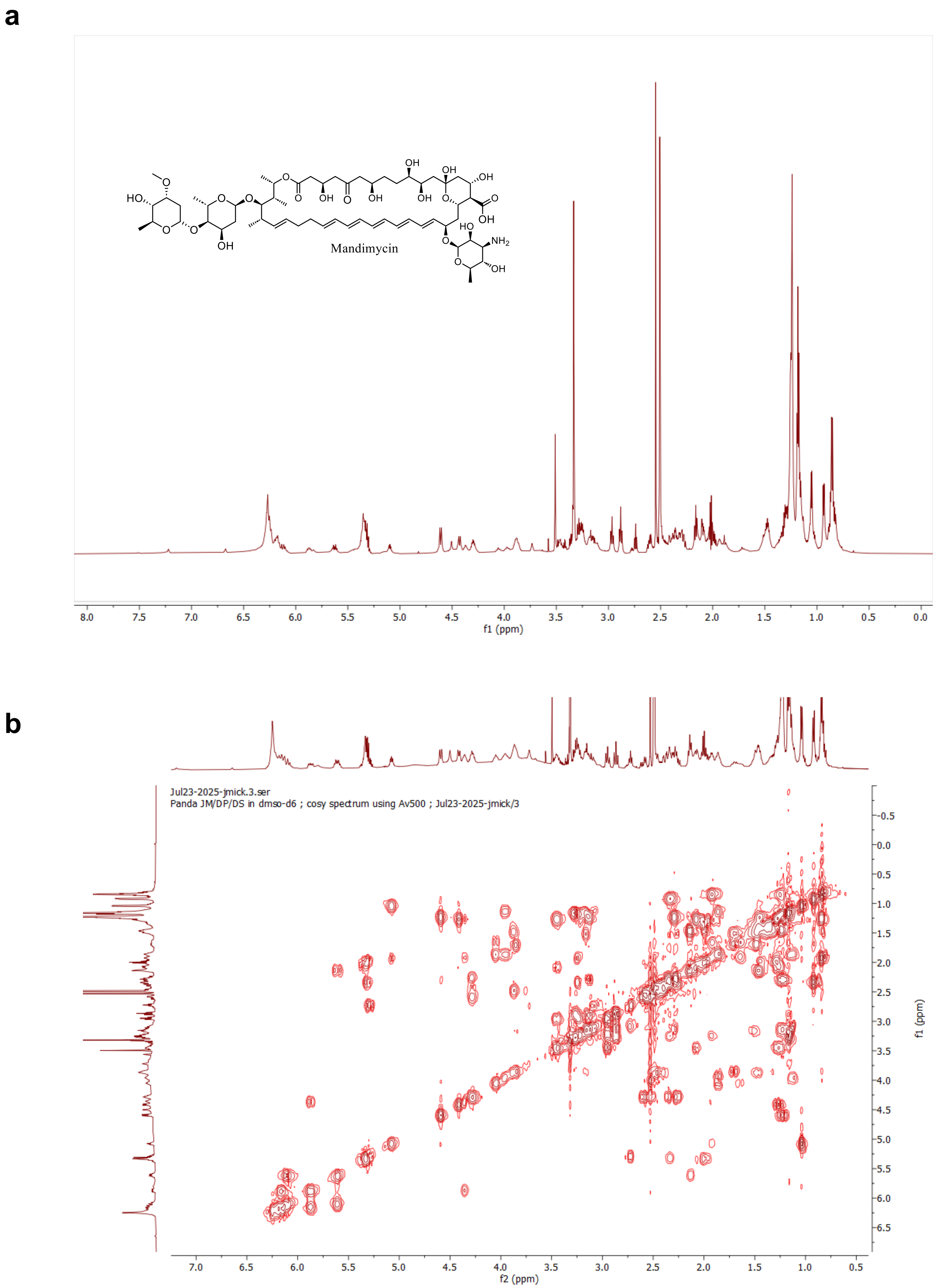

Supplementary Fig. 15. NMR data for mandimycin. (a) shows ^{1}H NMR spectrum of mandimycin in DMSO-*d6*. **(b)** shows ^{1}H-^{1}H COSY NMR of mandimycin in DMSO-*d6*

c

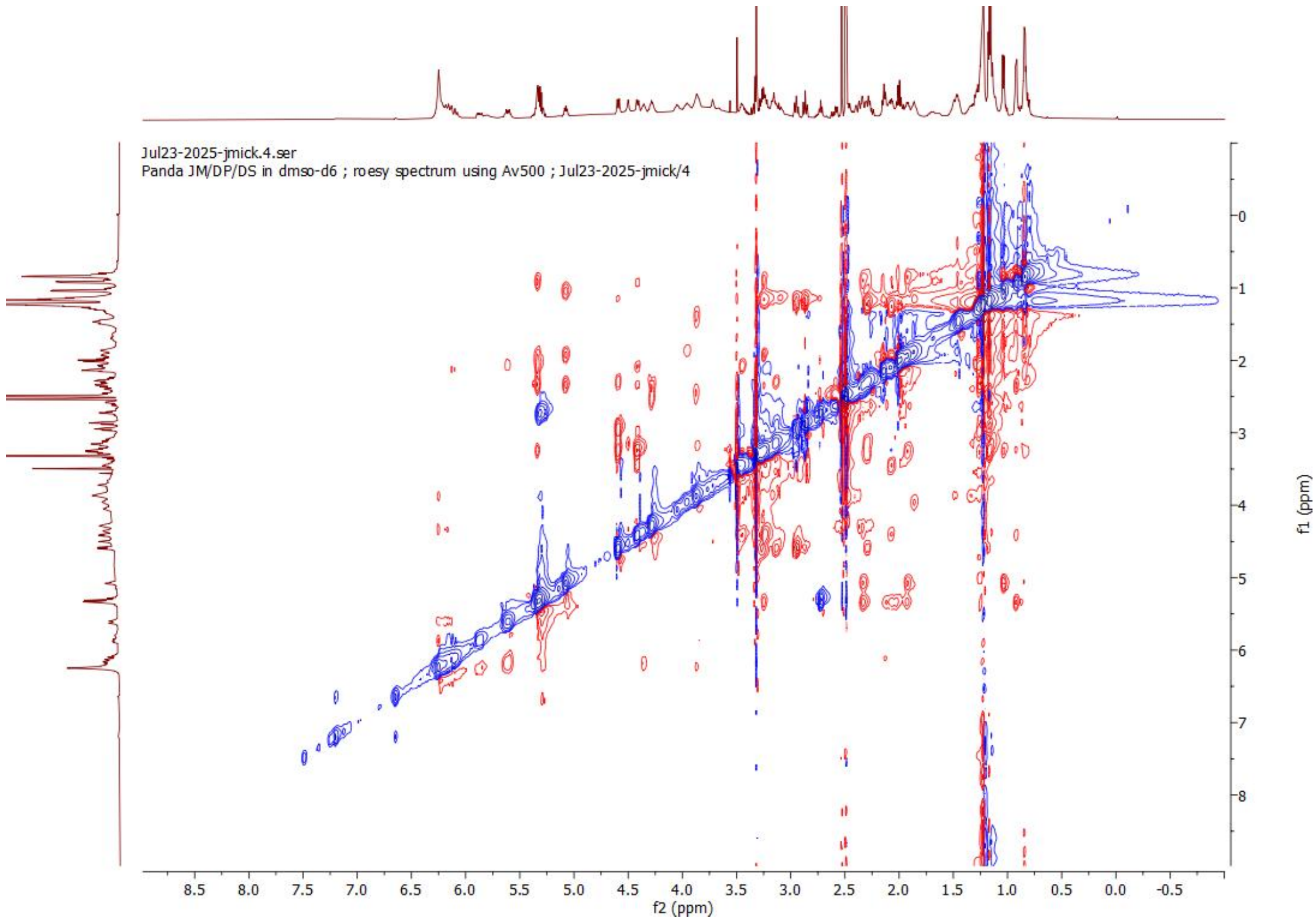

d

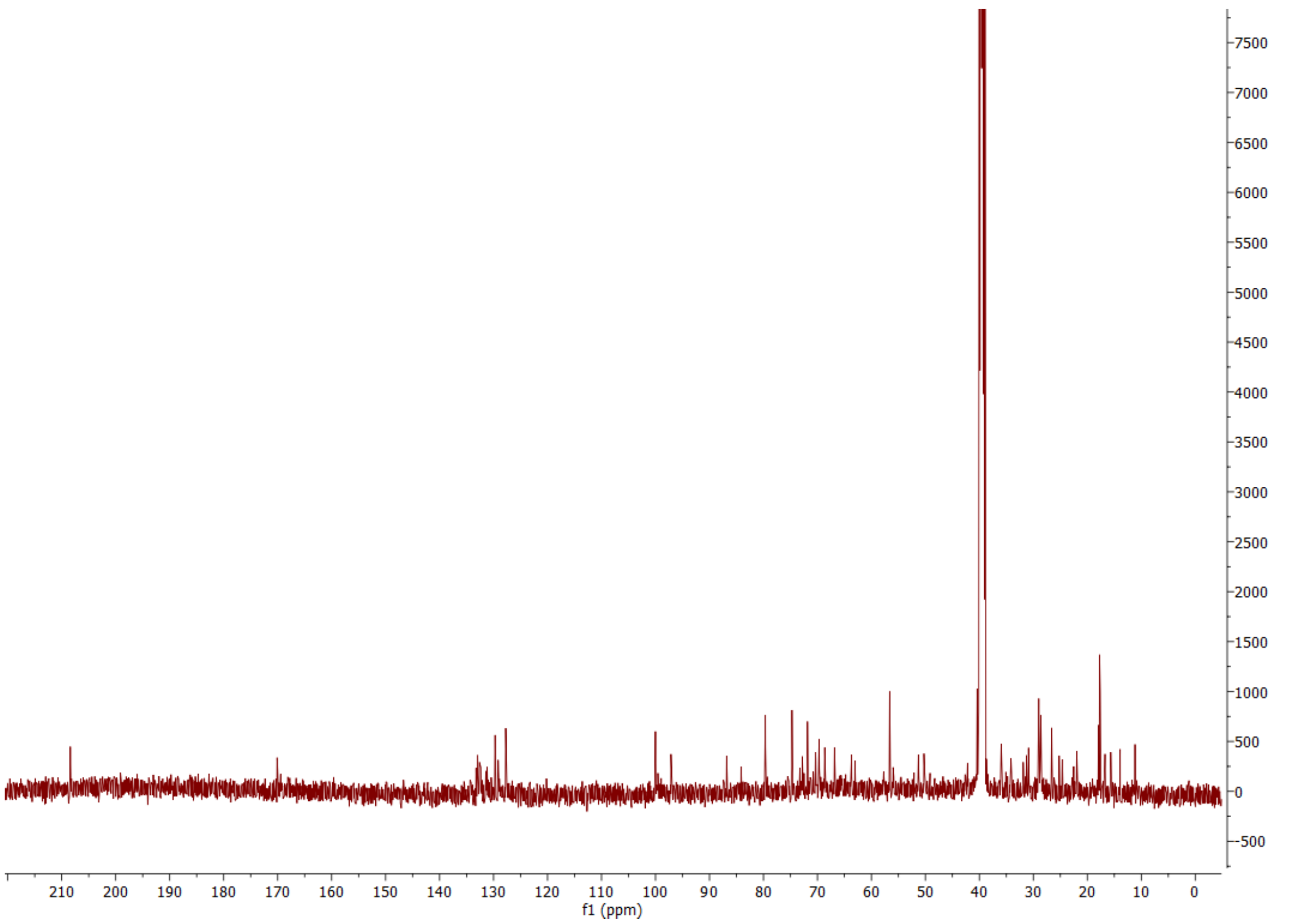

Supplementary Fig. 15. NMR data for mandimycin continued. (c) ^{1}H-^{1}H ROESY NMR of mandimycin in DMSO-*d6*. **(d)** shows ^{13}C NMR of mandimycin in DMSO-*d6.*

e

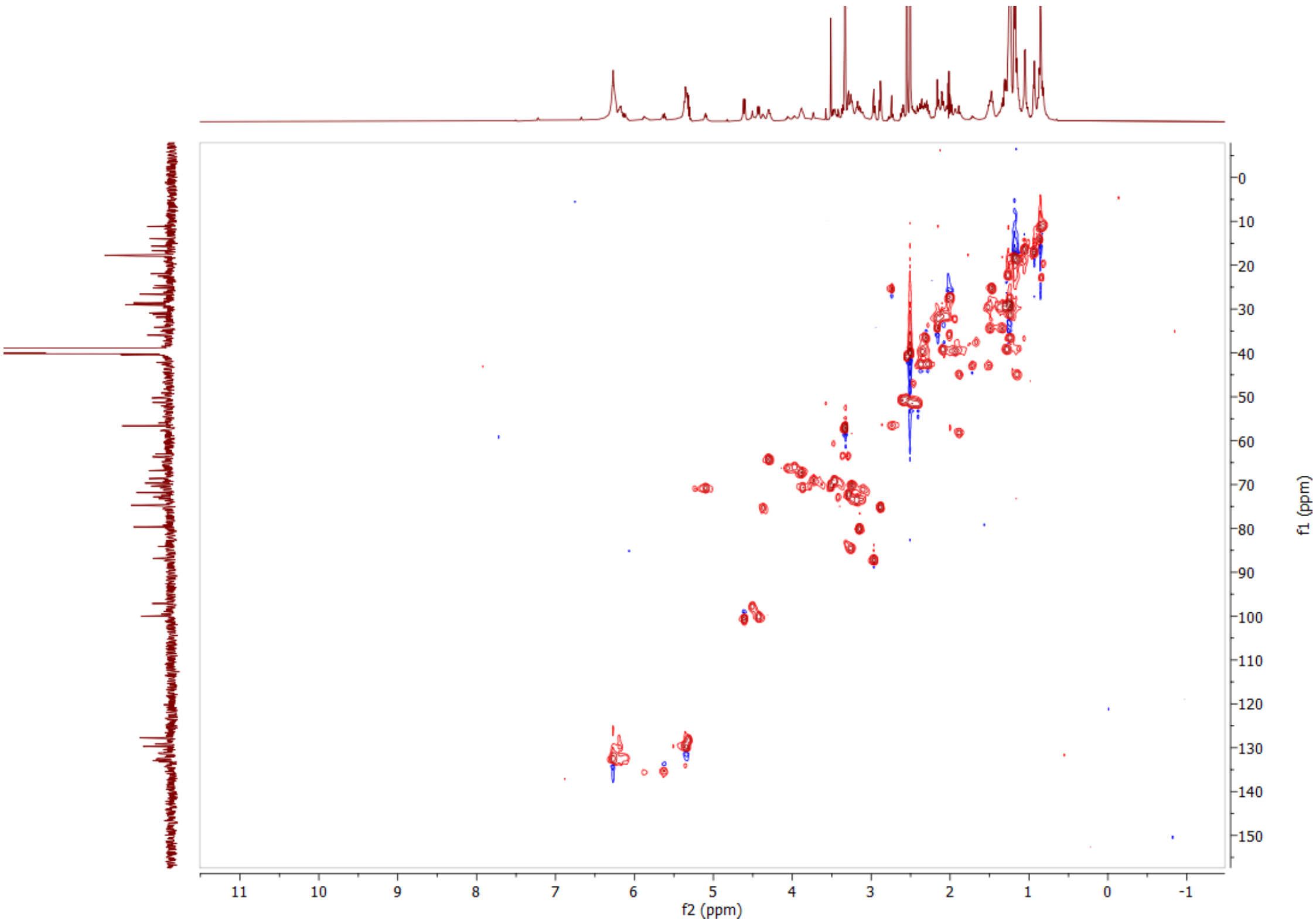

f

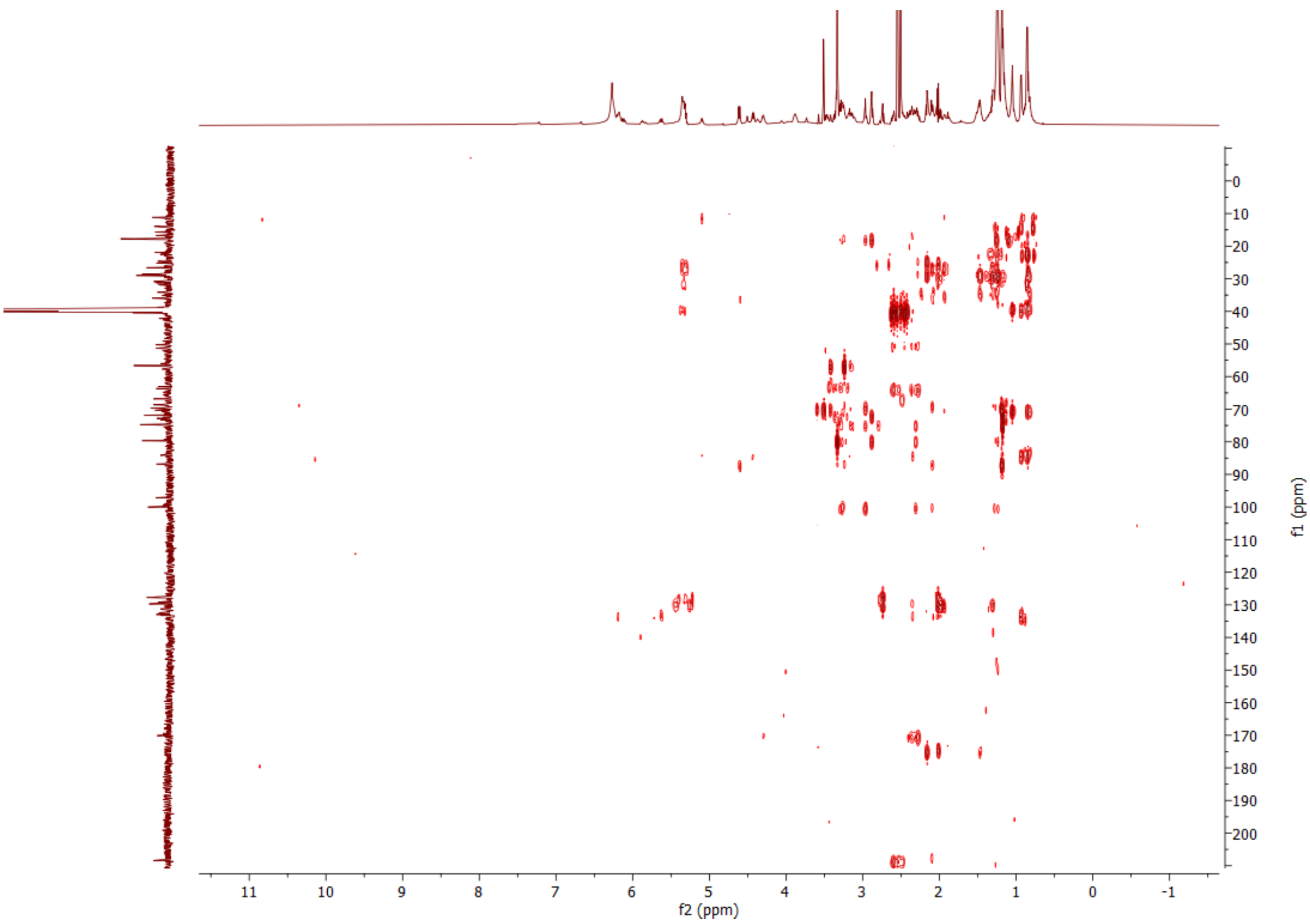

Supplementary Fig. 15. NMR data for mandimycin continued. (e) shows ^{1}H-^{13}C- HSQC NMR of mandimycin in DMSO-*d6.* **(f)** shows ^{1}H-^{13}C- HMBC NMR of mandimycin in DMSO-*d6*.

g

Position	^{1}H found mandimycin in DMSO-*d6*	^{13}C found mandimycin in DMSO-*d6*	Position	^{1}H found mandimycin in DMSO-*d6*	^{13}C found mandimycin in DMSO-*d6*
1		170.13	32	5.33	129.22
2	2.27, 2.36	42.22	33	5.34	133.32
3	4.28	63.73	34	2.34	38.71
4	2.54, 2.59	50.31	35	3.26	84.13
5		208.44	36	1.92	38.74
6	2.42; 2.47	51.34	37	5.08	70.42
7	3.86	66.82	16-COOH		174.91
8	1.33; 1.48	34.23	34-CH_3	0.93	16.72
9	1.37; 1.49	28.82	36-CH_3	0.85	11.11
10	3.88	66.81	37-CH_3	1.05	15.62
11	4.00	66.34	1'	4.51	97.14
12	1.15; 1.87	44.43	2'	3.73	68.72
13		97.11	3'	2.73	56.63
14	1.50; 1.71	42,62	4'	3.18	73.33
15	3.98	65.63	5'	3.27	71.92
16	1.89	57.72	6'	1.17	17.84
17	4.05	65.54	1''	4.42	99.53
18	1.67; 1.91	37.52	2''	1.28; 2.08	38.52
19	4.36	74.73	3''	3.45	68.74
20	5.87	135.52	4''	2.96	86.82
21	6.15	131.22	5''	3.24	69.73
22	6.05-6.32	127.71-133.74	6''	1.17	17.83
23	6.05-6.32	127.71-133.74	1'''	4.61	100.00
24	6.05-6.32	127.71-133.74	2'''	1.23; 2.30	35.72
25	6.05-6.32	127.71-133.74	3'''	3.14	79.73
26	6.05-6.32	127.71-133.74	4'''	2.88	75.10
27	6.05-6.32	127.71-133.74	5'''	3.28	71.93
28	6.11	131.43	6'''	1.17	17.62
29	5.62	134.41	3'''-OCH_3	3.32	56.70
30	2.16	31.92			
31	2.03; 2.15	31.54			

Supplementary Fig. 15. NMR data for mandimycin continued. (g) shows ^{1}H and ^{13}C NMR spectral data for mandimycin in DMSO-*d6*.

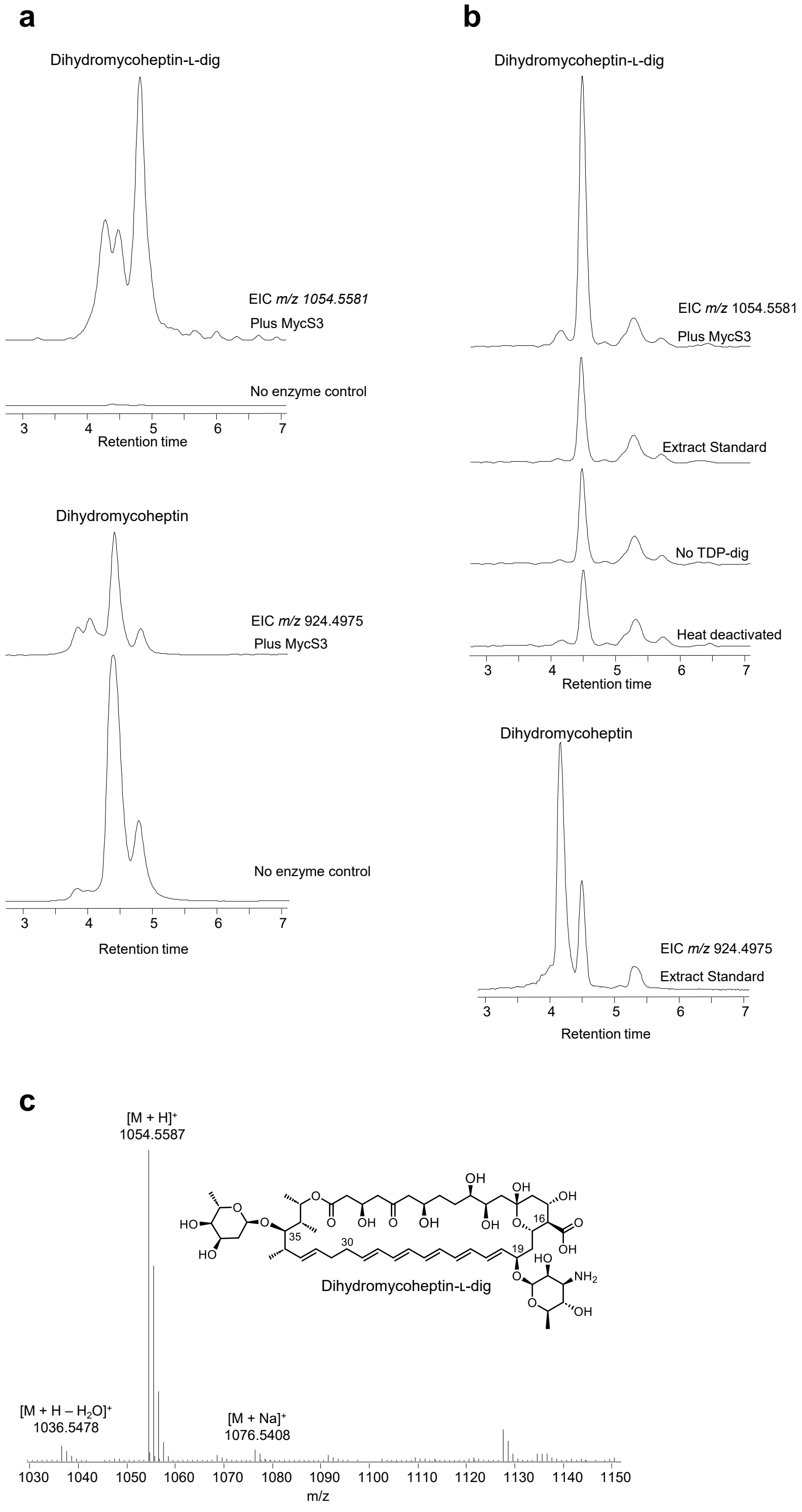

Supplementary Fig. 16. Glycosylation reaction of dihydromycoheptin with TDP-L-digitoxose catalysed by MycS3. (a) Comparison between EIC traces of dihydromycoheptin-L-dig product calc. *m/z* = 1054.5581 $[M+H]^+$, and dihydromycoheptin starting material calc. *m/z* = 924.4975 $[M+H]^+$ showing conversion of semi pure dihydromycoheptin to dihydromycoheptin-L-dig in the presence of TDP-L-digitoxose and MycS3, vs control reaction without enzyme. **(b)** Comparison between EIC traces of dihydromycoheptin-L-dig calc. *m/z* = 1054.5581 $[M+H]^+$, and dihydromycoheptin calc. *m/z* = 924.4975 $[M+H]^+$ showing enrichment of dihydromycoheptin-L-dig in methanolic *S. netropsis* extract in the presence of TDP-L-digitoxose and MycS3, vs control reactions. **(c)** EIC trace of dihydromycoheptin-L-dig product calc. *m/z* = 1054.5581 $[M+H]^+$, obs. *m/z* = 1054.5587 $[M+H]^+$.

a

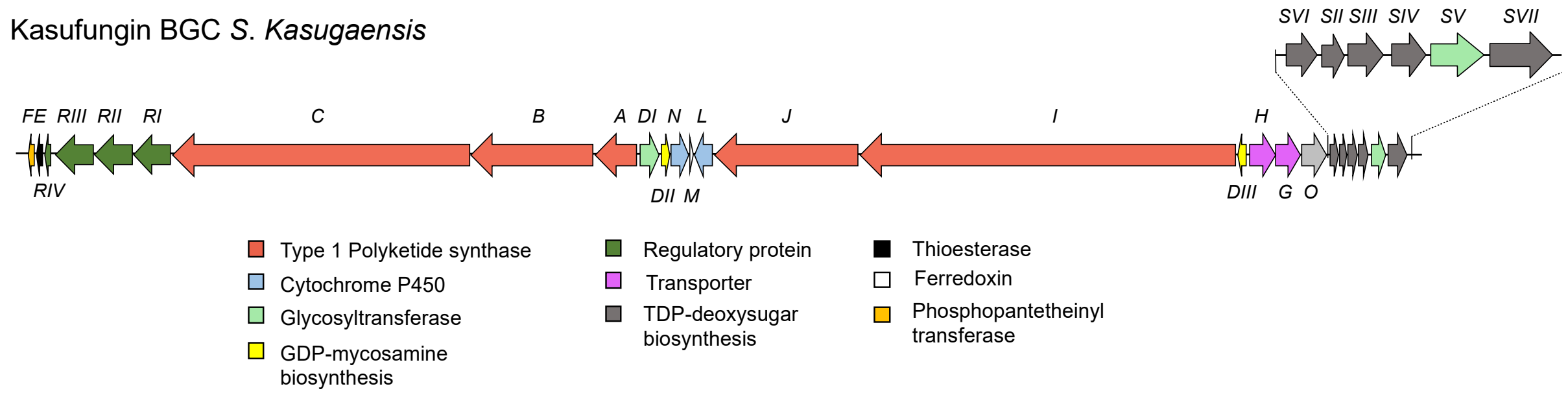

Gene	Putative protein	Putative function	NysBGC (% id.)†	SelBGC (% id.)†
kfuF	Phosphopantetheinyl transferase	Phosphopantetheinyl transferase	NysF (59%)	---
kfuE	Thioesterase	Proofreading thioesterase	NysE (60%)	SelE, ANY10602.1 (56%)
kfuRIV	Transcriptional regulator	Regulation	ORF4 (70%)	SelRIV, ANY10577.1 (47%)
kfuRIII	Transcriptional regulator	Regulation	NysRIII (56%)	SelRIII, ANY10580.1 (40%)
kfuRII	Transcriptional regulator	Regulation	NysRII (49%)	SelRII, ANY10581.1 (35%)
kfuRI	Transcriptional regulator	Regulation	NysRI (59%)	SelRI, ANY10582.1 (45%)
kfuC	T1PKS	Modules 3-6	NysC (62%)	SelC, ANY10589.1 (59%)
kfuB	T1PKS	Modules 1-2	NysB (72%)	SelB, ANY10590.1 (72%)
kfuA	T1PKS	Loading module	NysA (52%)	SelA, ANY10591.1 (56%)
kfuDI	Glycosyltransferase	Mycosaminylation	NysDI (78%)	SelDI, ANY10585.1 (66%)
kfuDII	Aminotransferase	GDP-mycosamine biosynthesis	NysDII (82%)	---
kfuN	Cytochrome P450	Carboxylation	NysN (79%)	---
kfuM	Ferredoxin	Electron transfer	NysM (60%)	---
kfuL	Cytochrome P450	Hydroxylation	NysL (69%)	SelL, ANY10587.1 (55%)
kfuJ	T1PKS	Modules 13-14	NysK (65%)	SelK, ANY10588.1 (57%)
kfuI	T1PKS	Modules 7-12	NysI (64%)	SelI, ANY10600.1 (61%)
kfuDIII	GDP-mannose 4,6 dehydratase	GDP-mycosamine biosynthesis	NysDIII (85%)	SelDIII, ANY10601.1 (78%)
kfuH	ABC transporter	Efflux	NysH (65%)	SelH, ANY10583.1 (31%)
kfuG	ABC transporter	Efflux	NysG (67%)	---
kfuO	Oxidoreductase	Unknown	---	---
kfuSVI	3-ketoreductase	TDP-deoxysugar biosynthesis	NysSVI (63%)*	SelSVI, ANY10593.1 (47%)
kfuSII	3,5 epimerase	TDP-deoxysugar biosynthesis	NysSII (74%)*	SelSII, ANY10597.1 (69%)
kfuSIII	Glu-1-P thymidylyltransferase	TDP-deoxysugar biosynthesis	NysSIII (54%)*	SelSIII, ANY10596.1 (39%)
kfuSIV	4,6 dehydratase	TDP-deoxysugar biosynthesis	NysSIV (65%)*	SelSIV, ANY10595.1 (64%)
kfuSV	Glycosyltransferase	Deoxysugar attachment	NysSV/GT3 (60%)*	SelSV, ANY10594.1 (33%)
kfuSVII	2,3 dehydratase	TDP-deoxysugar biosynthesis	NysSVII (63%)*	SelSVII, ANY10592.1 (50%)

Supplementary Fig. 17. Bioinformatics analysis of kasufungin BGC. (a) shows predicted function and homology of genes involved in kasufungin biosynthesis. †% identity with gene products from the NysA1 and selvamicin BGC. *The nystatin-associated TDP-deoxysugar subcluster is included here as NysSV putatively acts on NysA1 in *S. noursei* to produce NysA3 and nystatin 1070. The proposed function of L-digitoxose sugar pathway from kasufungin BGC was inferred from the homology of its genes to those in the predicted TDP-sugar subcluster of the selvamicin producer.[23] The presence of C27 L-digitoxose on kasufungin B validates this prediction (Supplementary Fig. 18). While TDP-L-digitoxose subclusters have been observed in other biosynthetic gene clusters, their enzymes have not been fully characterised *in vitro and* remain to be tested.[65]

b

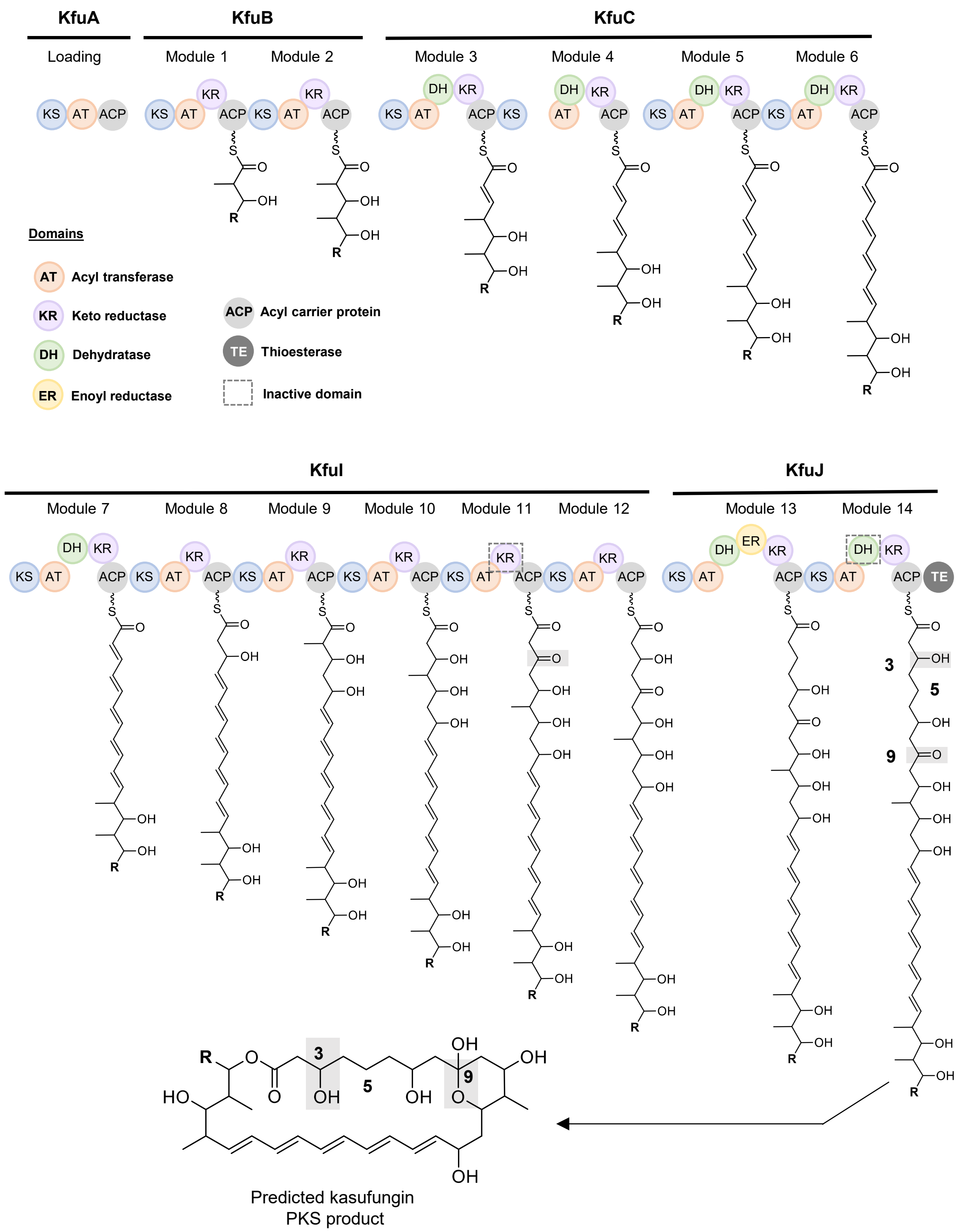

Supplementary Fig. 17. Bioinformatics analysis of kasufungin BGC continued. (b) shows bioinformatically proposed biosynthesis of kasufungin core macrolide. The PKS has 5 genes encoding 15 modules. Biosynthetically inactive domains shown with dashed boxes were predicted based on the absence of conserved residues (Supplementary Fig. 17d). These predictions were further validated by NMR structural characterisation (Supplementary Fig. 18). For instance, a dehydratase (DH) domain of module 11 is present in the PKS (KfuJ) sequence and it must be inactive (or it is skipped) otherwise the C3-OH group in the kasufungin products, which is clear from our NMR experiments, would not be present. The DH if active would have eliminated the C3-OH and there would be a C2-C3 double bond.

c

Kasufungin PKS	PKS module	Kasufungin domains	Selvamicin domains
KfuA	Loading	KS-AT-ACP	KS-AT-DH-ACP
KfuB	1	KS-AT(mmal)-KR-ACP	KS-AT(mmal)-KR-ACP
	2	KS-AT(mmal)-KR-ACP	KS-AT(mmal)-KR-ACP
KfuC	3	KS-AT(mal)-DH-KR-ACP	KS-AT(mal)-DH-KR-ACP
	4	KS-AT(mal)-DH-KR-ACP	KS-AT(mal)-DH-KR-ACP
	5	KS-AT(mal)-DH-KR-ACP	KS-AT(mal)-DH-KR-ACP
	6	KS-AT(mal)-DH-KR-ACP	KS-AT(mal)-DH-KR-ACP
KfuI	7	KS-AT(mal)-DH-KR-ACP	KS-AT(mal)-DH-KR-ACP
	8	KS-AT(mal)-KR-ACP	KS-AT(mal)-KR-ACP
	9	KS-AT(mmal)-KR-ACP	KS-AT(mmal)-KR-ACP
	10	KS-AT(mal)-KR-ACP	KS-AT(mal)-KR-ACP
	11	KS-AT(mal)-KR-ACP	KS-AT(mal)-KR-ACP
	12	KS-AT(mal)-KR-ACP	KS-AT(mal)-KR-ACP
KfuJ	13	KS-AT(mal)-DH-ER-KR-ACP	KS-AT(mal)-DH-ER-KR-ACP
	14	KS-AT(mal)-DH-KR-ACP-TE	KS-AT(mal)-DH-ER-KR-ACP-TE

Supplementary Fig. 17. Bioinformatics analysis of kasufungin BGC continued. (c) shows comparison between the kasufungin and selvamicin PKS modules. Catalytically inactive domains shown in red. mal = malonyl-CoA, mmal = methylmalonyl-CoA. The kasufungin and selvamicin PKSs are similar, except module 13 of the kasufungin PKS is predicted to catalyse a full reductive cycle, whereas inactive domains (DH13 and ER13) in the corresponding selvamicin PKS result in a hydroxyl substituent at position C5. Conversely, module 14 of the kasufungin PKS is predicted to install a hydroxyl group at C3 due to the predicted inactivity of DH14, whereas in the selvamicin PKS active DH14 and ER14 domains result in a C3 methylene group. The acyltransferase (AT) domain within the loading module of both PKSs is predicted to be acetyl-CoA selective, but the C29-ethyl substituent of selvamicin indicates selectivity for a propionyl-CoA starter unit.

d

AT domains:

```
                                         Specificity motif                                         Active site
Malonyl-CoA                              HAFH
Methymalonyl-CoA                         YASH                                                        GHS G
Loading        LAERGRKTSRLRVSHAFHSPLMDPMLDDFRRIAESIAYHAPLLPVVSNLTGGLASAAELC   FVG GHSIGEI
Module3        LAEQGRKTSRLRVSHAFHSPLMDPMLDDFRHLAESITYHAPRIPVISNLTGEPAPSAELC   FVGGHSIGEI
Module6        LAADGRKTTRLRVSHAFHSPLMDPMLDDFRTVVEGLTFHEPDIAVVSHLTGGLAAADELR   FVG GHSIGEV
Module4        LVGEGRKATRLSVSHAFHSPLMDPMLDDFRAVVEGLSFADPLIPVVSNLTGGVADAGELC   FVG GHSIGEV
Module5        LVGEGRKATRLSVSHAFHSPLMDPMLDDFRAVVEGLSFADPLIPVVSNLTGGLAGAGELC   FVG GHSIGEI
Module12       --RLGRKKSRLPVSHAFHSPLMDPVLDEFRAVVAGLTFTPPALPVVSNLTGAVATAEELC   FVA GHSIGEI
Module13       FKSQGRKTSRLSVSHAFHSPLMDPMLDDFRAAVAGLTFAEPTIPVVSNLTGDPASAEELC   FVG GHSIGEI
Module14       FKSQGRKTSRLSVSHAFHSPLMDPMLDDFRAAVAGLTFAEPTIPVVSNLTGDLASAEELC   FVG GHSIGEI
Module7        FAAQGRKTTRLRVSHAFHSPRMDPMLDAFRRVVAGLSLQAPLTPIVSNLTGEPVTVQQVT   FLT GHSIGEI
Module11       FAEQGRRTRRLRVSHAFHSPLMDPMLADFARIAGQLEFRDPQVPVVSNLTGRLATDEELT   FVA GHSIGEL
Module8        FAAEGRKTKRLRVSHAFHSPRMDAMLDDFATVAGSVSYHPPRIPFVSNVTGALATDEQVC   FLA GHSIGEL
Module10       FAAEGRKTKRLRVSHAFHSPRMDAMLDDFATVAGSVSYHPPRIPFVSNVTGALATDEQVC   YLA GHSIGEL
Module2        CERQQVRARRVTVDYASHGAHVEAVRAELADVLGDIRPTPPRVPFYSTVTGGRLETA-AF   AVV GHSQGEI
Module1        LSAEEVRVRRIAVDYASHSAQVEDLREELLDVLAPVQPRRGEVPFLSTVTGDWLDTT-EL   AVI GHSQGEI
Module9        LSAEEVRVRRIAVDYASHSAQVEDLREELLDVLAPVQPRRPEVPFLSTMTGDWLETA-AT   AVIGHSQGEI
```

KR domains:

```
                                   S             Y   N
Module11       LDDLLGDRPLDAFVLFSSVAGVWGVRGQGERAALGARLAAFARQRRDRGVPALSVAWGAW
Module1        LDELLDNDALDAFVLYSSTAGMWGSGRHAAYGAGNAFLGALADNRRARGLTATSLHWGKW
Module9        LDELLADDALDSFVLFSSTAGMWGSGRHAAYVAGNAFLGALADNRRARGLTGTALSWGIW
Module13       LHEATRDLDLAAFVLYSSTAGVLGSPGQANYAAGNAFLDALAHHRHALGLPALSLAWGAW
Module3        LHELTRDLDLSAFVMFSSVAATLGSAGQANYAAGNAFLDALAEHRRRAGLPGLALAWGPW
Module14       LHEATRDLDLAAFVMYSSISGVMGSPGQANYAAGNAFLDALAQHRRAQGLPAVSLAWGPW
Module4        LHELTRDLDLSAFVLFSSAAGTFGSAGQGNYAAGNAFLDALAGHRRAEGLPGVSLAWGPW
Module5        LHELTRDLDLSSFVVFSSVAGTFGGAGQANYAAGNAFLDALVQRRRAEGLPGVSLAWGPW
Module7        LHELTRDLDLSAFVVFSSVAGTFGGAGQANYAAGNAFLDALVQRRRAEGLAGVSLAWGPW
Module6        LHELTRDLDLSAFVVFSSVAGTFGGAGQANYAAGNAFLDALVQRRRAEGLAGVSLAWGPW
Module12       LDELTRDLDLSAFVLFSSATAAVGNPGQANYAAANTVLDALAVRRRALGFPATSIAWGAW
Module8        LDALLGDRDLDLFVLFGSVAGVWGSGGQSAYGAANAYLDALAEHRRARGLTATCVAWGPW
Module2        LHELTQDLDLDAFVLFSSGAAVWGSGGQPGYAAANACLDALAAHRRAAGLTAASVSWGAW
Module10       LDELTEGMDLSAFVLFSSTAGVIGAAGQGNYAAANAYLDALAERRRARGRTATSVAWGPW
```

DH domains:

```
                                                  H   G    P                     Y
Module14       HPLLGAALAVAGSDEVVLTGRVSLRTHPWLADHTVRGQVRLPAAAFAELAV   AATGAARGPVFQGL
Module13       HPLLGAAMAVAGSDELVLTGTLSLATHPWLADHAVGGQALFPGTGFLELAI   ARTGLEYGPVFRGL
Module7        HPLLSAAVSLADSDGALLTGRLSLLTHPWLADHVVQGSVLLPGTAFLELAV   AADGFAYGPVFQGL
Module3        HPLLGAAVELADGEGLLFTSRLSATTHPWLADHGVQGRVLLPGTAFVELAI   AGLGFDYGPVFRGL
Module6        HPLLGAAVPLAGGEGVVFTGRLSVPSHPWLADHAVLGHVLLPGTAFVELAL   AGLGFAYGPVFRGL
Module4        HPLLGAAVELAQGEGVLFTGRLSVQSHPWLVDHAVLGRVLLPGTAFVELAL   AELGFGYGPVFQGL
Module5        HPLLNGSVELAGTDGVLFTGRLSVQSHPWLVDHAVLGRVLLPGTAFVELAL   AELGFGYGPVFQGL
```

ER domains:

```
                                                              K       D
Module13       SRTTAFAETFTAVTGGAGVDVVLNSLSGEYVDASLRLLAEGGRFLEMGKTDI   EAFDLG
NysJ_ER        SRTLDFEAAFAEVAGDRGLDVVLNALSGEFVDASMRLLGDGGRFLEMGKTDI   HSFDLG
```

Supplementary Fig. 17. Bioinformatics analysis of kasufungin BGC continued. (d) shows domain activity and specificity analysis of kasufungin PKS. AT domains: Acyl-CoA specificity of AT domains analysed by comparison of specificity motif to the commonly seen malonyl-CoA HAFH motif (blue) and methylmalonyl-CoA YASH motif (green). The active site GHSxG motif is shown in bold. KR, DH and ER domains: Conserved residues associated with active domains are shown in bold. Variations from these residues are shown in red and suggest domain inactivity. The module 13 ER domain is aligned with the active ER domain from NysJ in NysA1 biosynthesis.

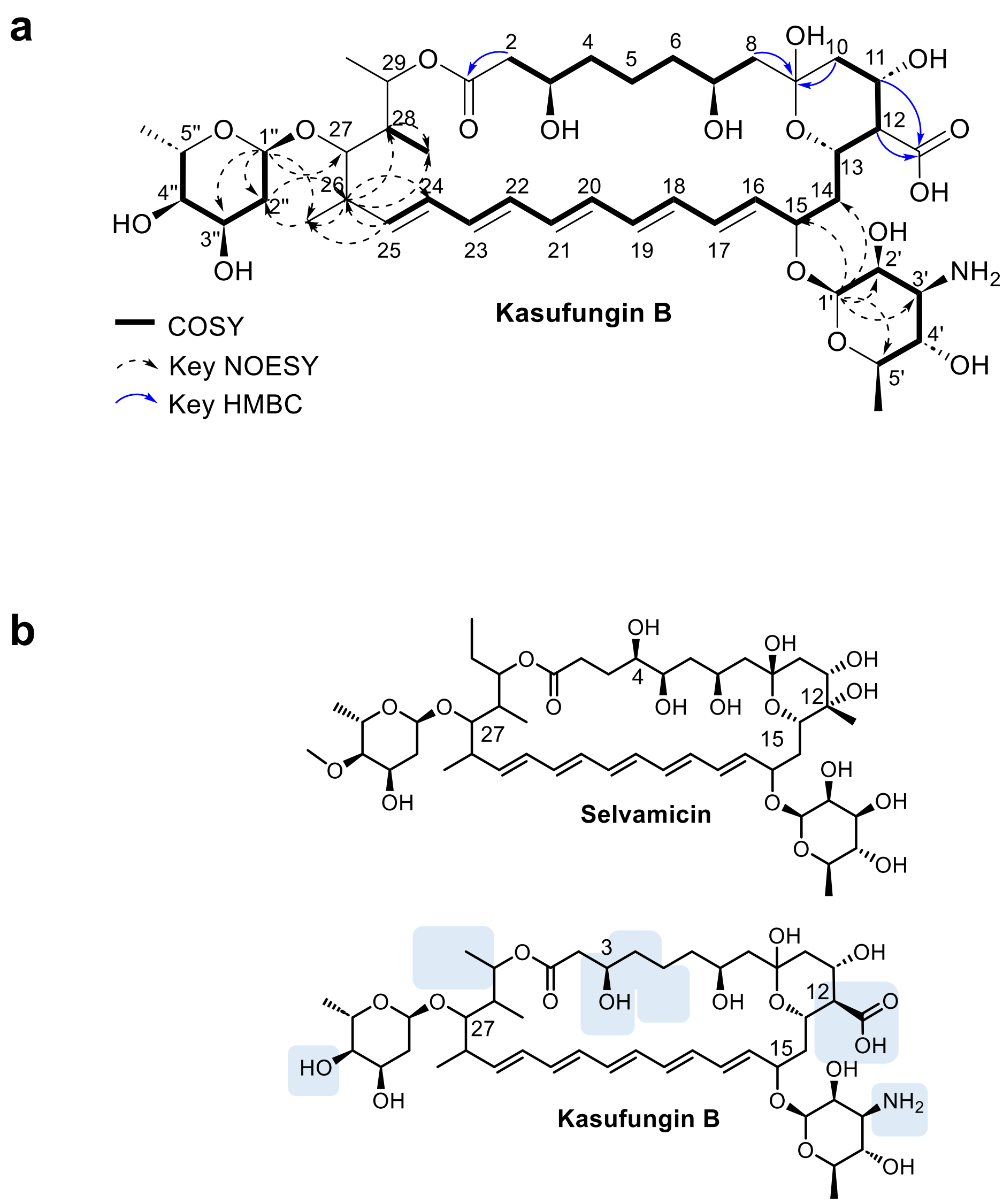

Supplementary Fig. 18. NMR data for kasufungin. **(a)** Key NMR correlations establishing the structure of kasufungin B. **(b)** Comparison of the structures of selvamicin [23] and kasufungin B. Structural differences are highlighted in blue. Unlike selvamicin, kasufungin B has a carboxylic acid moiety at C12, which is evident from the HMBC correlation between H12 and a typical CO_2H signal at δ C 178.30. HSQC experiments showed two distinctive sets of anomeric signals consistent with two sugars. The first is D-mycosamine, which is evident from ROESY correlations (H1′ to H2′, H3′ & H5′) that are the same as we observed for NysA3. Coupling constants for the second sugar, H3′′-H4′′ (J = 3.1 Hz) and H4′′-H5′′ (J = 9.3 Hz), indicate the presence of L-digitoxose. Thus, in addition to differences in the macrolide (at C3, 5, 12 & 29), kasufungin B also has different sugars to selvamicin.

c

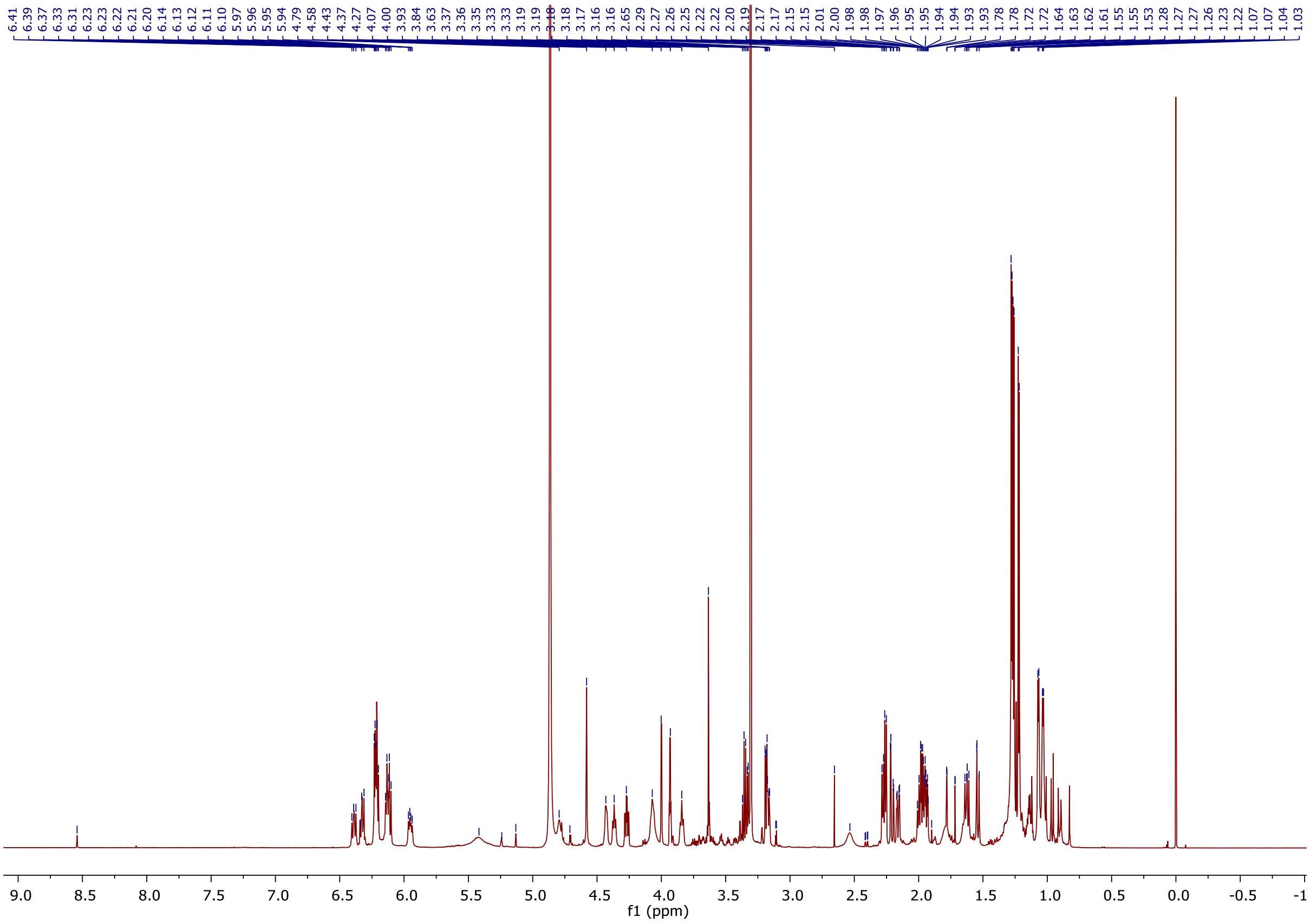

Supplementary Fig. 18. NMR data for kasufungin continued. (c) shows ^{1}H NMR spectrum of kasufungin B in methanol-*d4*.

d

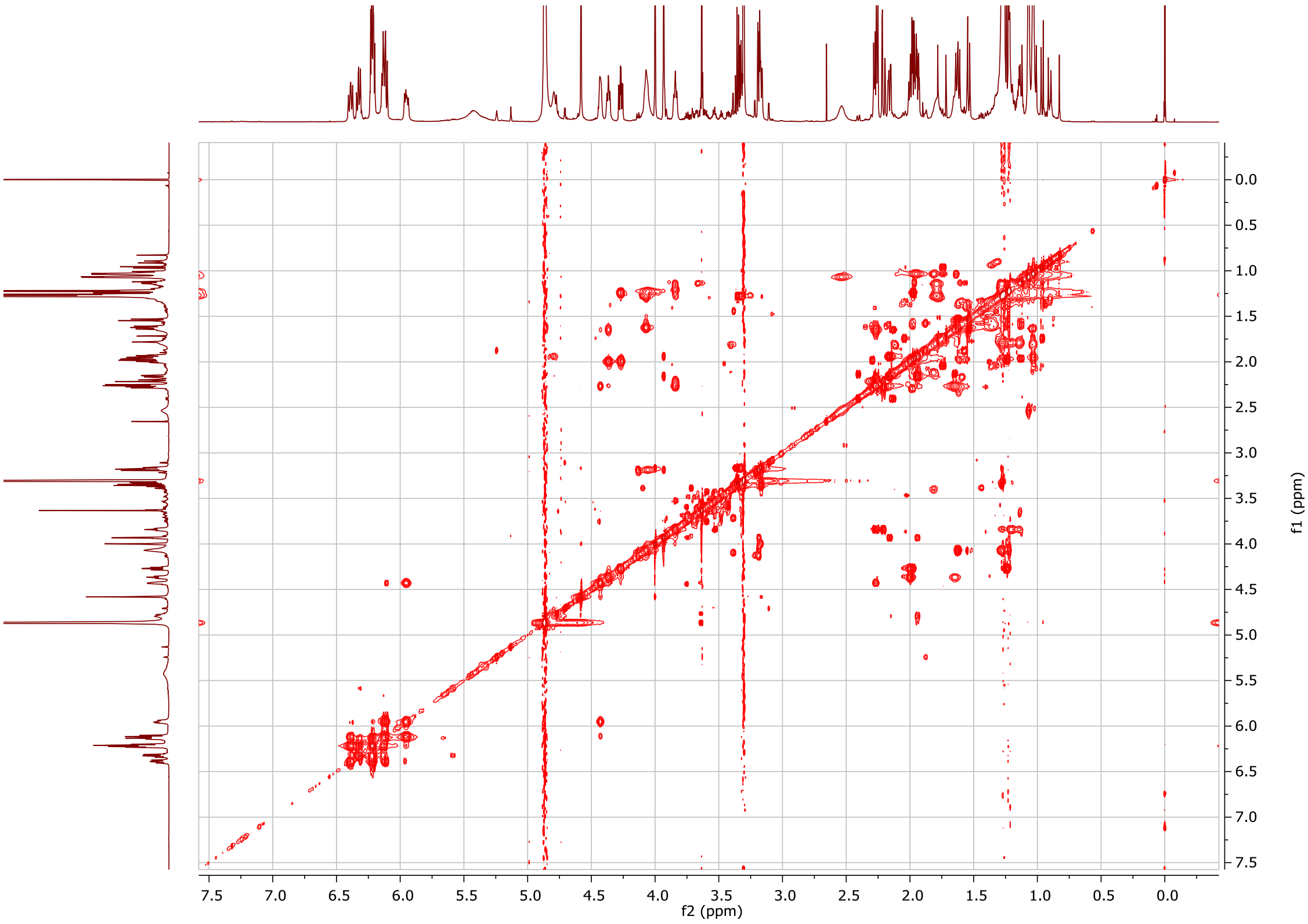

e

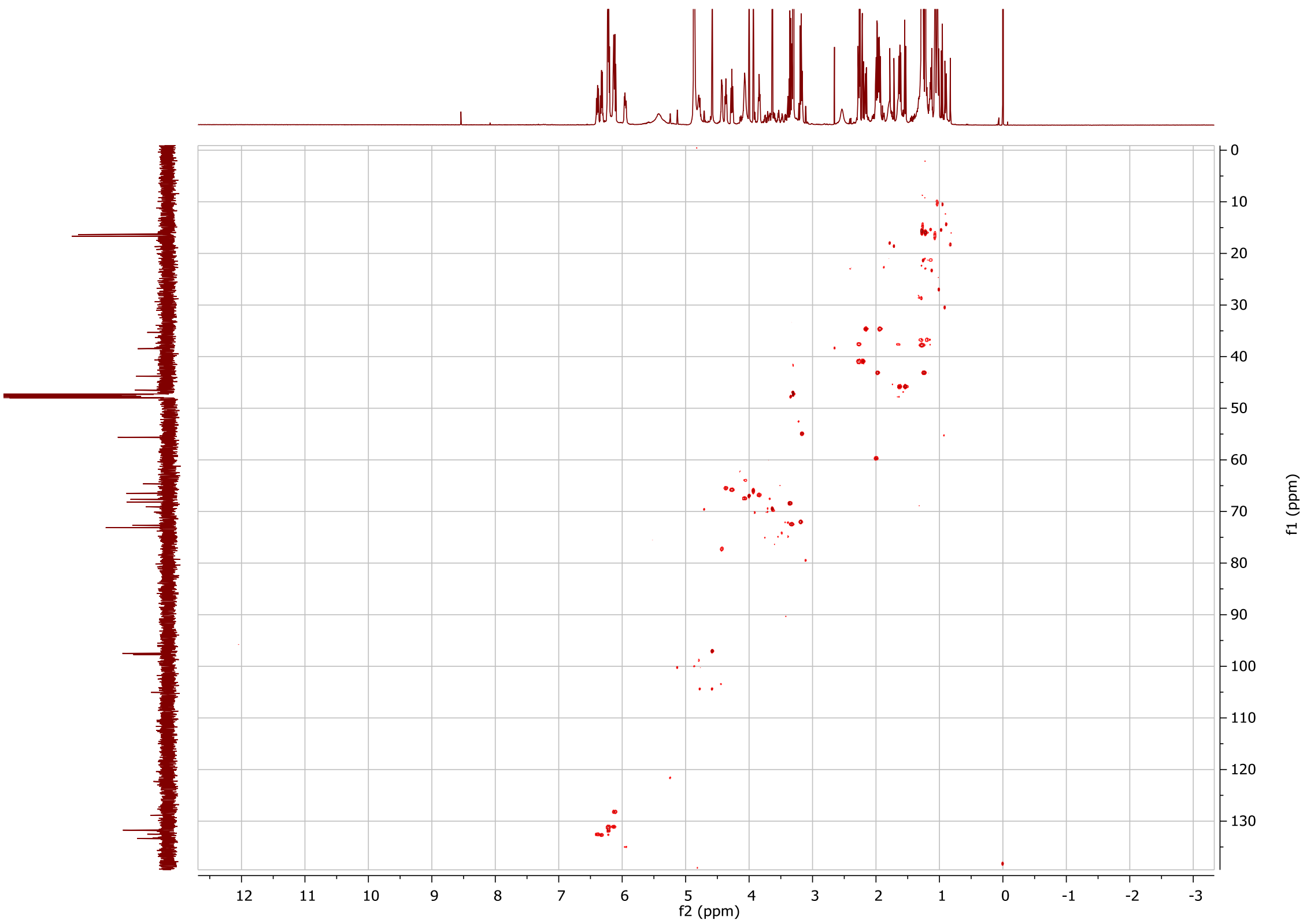

Supplementary Fig. 18. NMR data for kasufungin continued. (d) shows ^{1}H-^{1}H COSY spectrum of kasufungin B. **(e)** shows ^{1}H-^{13}C HSQC spectrum of kasufungin B.

f

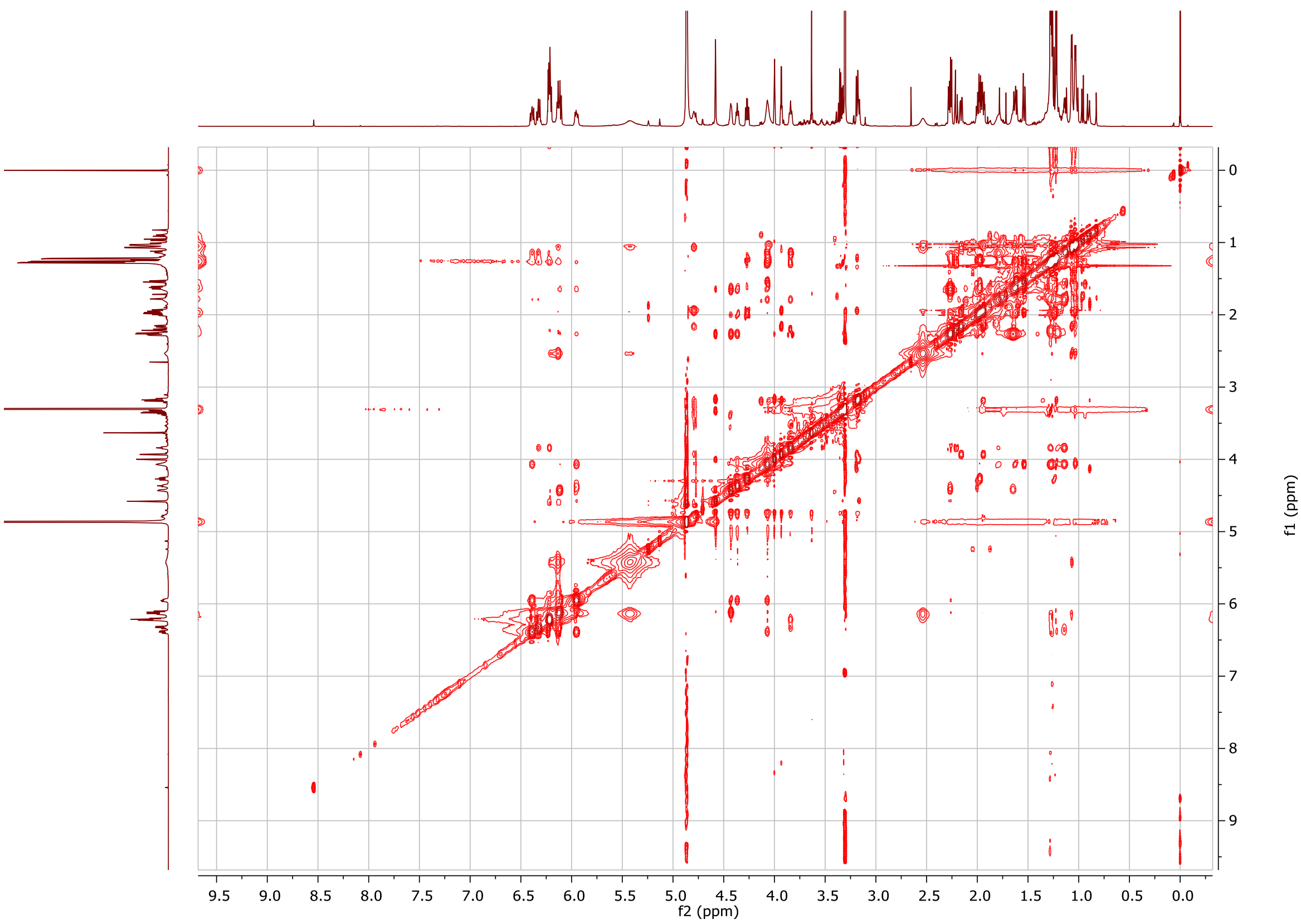

g

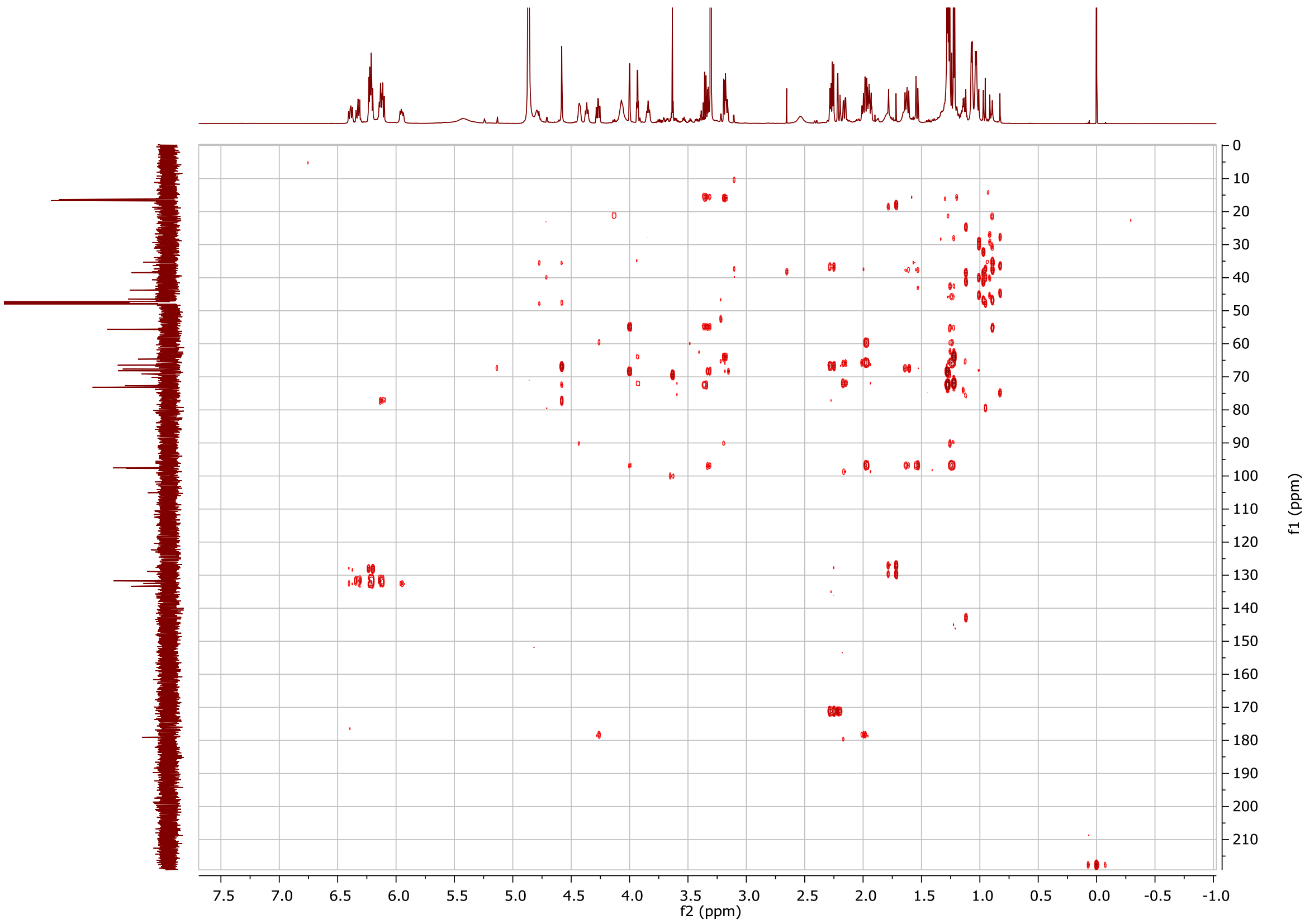

Supplementary Fig. 18. NMR data for kasufungin continued. (f) ^{1}H-^{1}H ROESY spectrum of kasufungin B. **(g)** ^{1}H-^{13}C HMBC spectrum of kasufungin B

h

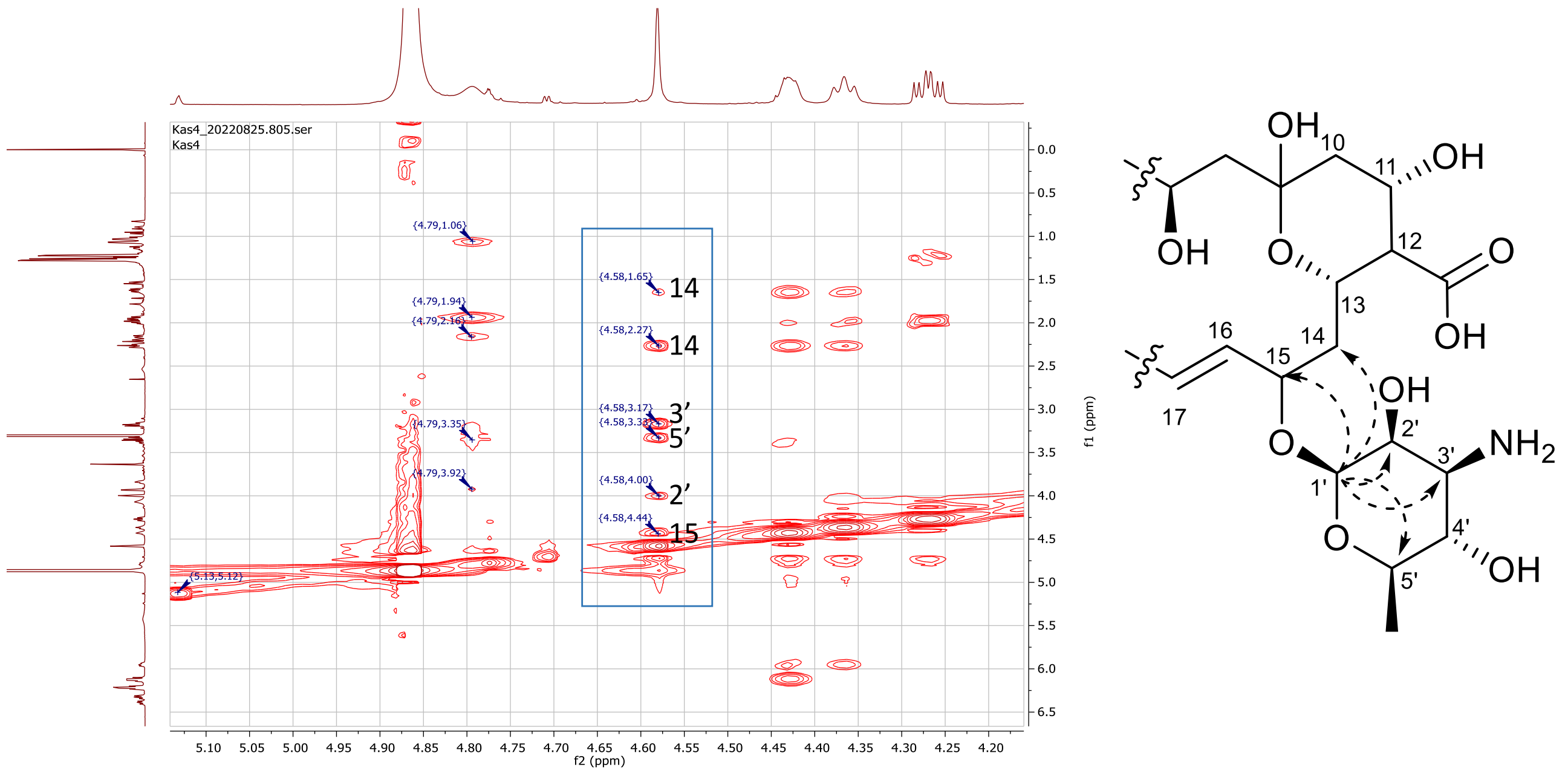

i

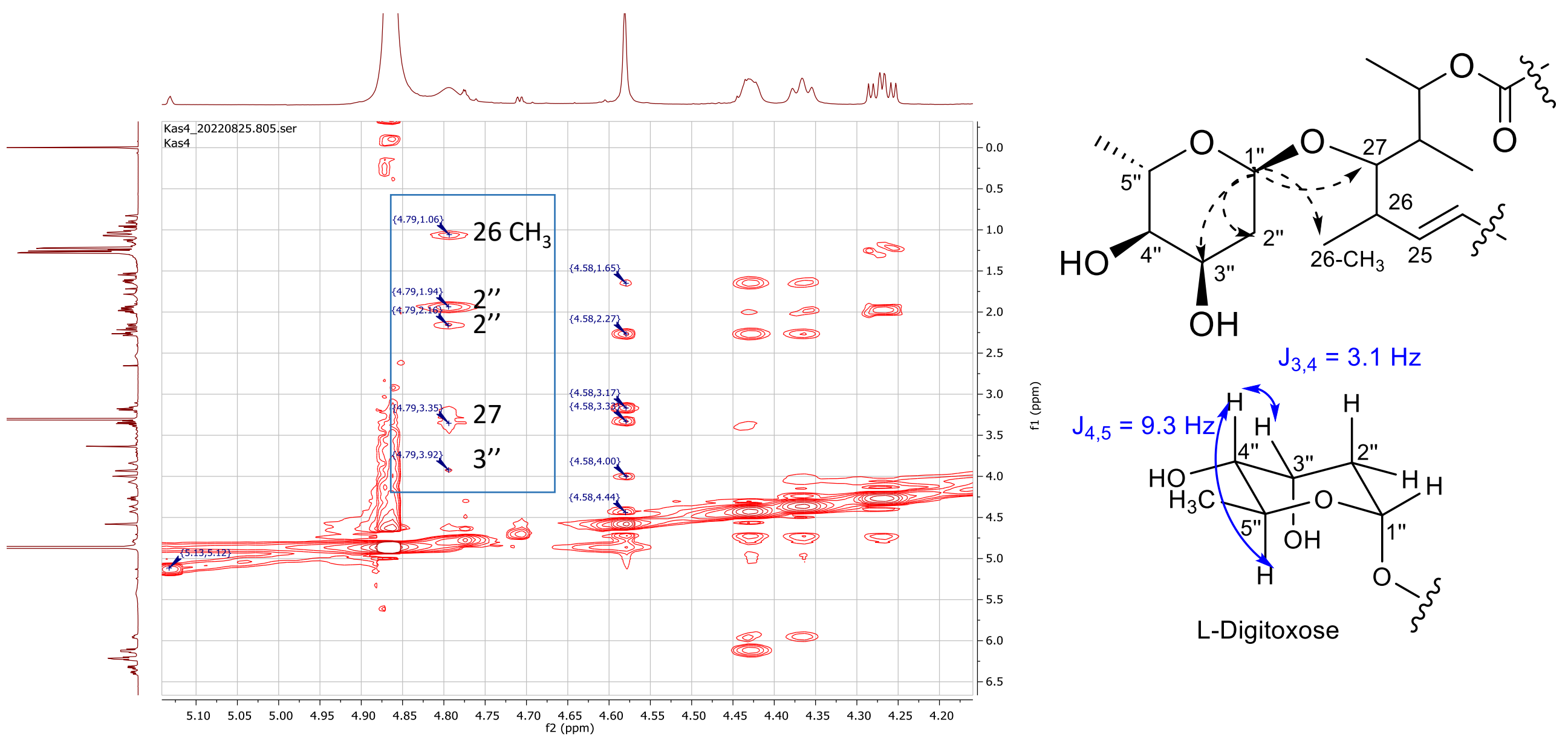

Supplementary Fig. 18. NMR data for kasufungin continued. (h and i) show expansion of ^{1}H-^{1}H ROESY spectrum of kasufungin B. **(h)** The correlation from H1' confirming the position and relative stereochemistry of the D-mycosamine. **(i)** Correlations from C1'' confirm the position of the sugar and coupling constants of $J_{4,3}$ at 3.1 Hz and $J_{4,5}$ at 9.3 Hz suggest that it is a L-digitoxose.

j

Position	^{1}H-Reported selvamicin in DMSO-d_6 [23]	^{13}C-Reported selvamicin in DMSO-d_6 [23]	^{1}H-Found kasufungin B in MeOD	^{13}C-Found kasufungin B in MeOD (Extracted from HSQC)
1	-	172.68	-	171.18 (HMBC)
2	2.52, 2.10	30.40	2.26, 2.21	41.36
3	1.81,1.36	27.79	3.84	67.23
4	3.10	72.00	1.19, 1.28	37.15
5	3.46	74.17	1.14, 1.79	21.70
6	1.63,1.28	39.17**	1.28	38.21
7	4.26	68.27	4.07	67.92
8	1.53	46.16	1.53, 1.62	46.23
9	-	97.32	-	96.80 (HMBC)
10	1.56, 1.52	40.39	1.24, 1.97	43.55
11	3.53	68.27	4.27	66.24
12	-	71.18	1.99	60.14
12-COOH	-	-	-	178.30 (HMBC)
12-CH_3	1.01	21.44	-	-
13	3.96	69.57	4.36	65.90
14	1.42, 2.18	33.38	1.65, 2.26	38.00
15	4.34	76.29	4.43	77.69
16	5.98	136.38	5.95	135.43
17	6.06	128.35	6.12	128.61
18	6.36	132.88	6.39	133.0
19-24	6.08-6.46	131.5 – 133.5	6.13-6.32	131.49-133.10
25	5.35	135.57*	5.43	-
26	2.50	42.85*	2.54	-
26-CH_3	1.01	17.87*	1.07	17.03
27	3.10	73.50*	3.35	68.35
28	1.82	39.30*	1.96	37.71
28-CH_3	0.93	12.17	1.04	10.72
29	5.22	73.53	(4.84)	-
29-CH_2	1.35, 2.06	22.68	-	-
29-CH_3	0.75	10.78	1.26	15.25
1’	4.40	96.80	4.58	97.49
2’	3.57	70.89	4.00	67.40
3’	3.18	73.65	3.16	55.38
4’	3.08	72.00	3.32	72.89
5’	3.06	72.13	3.32	72.89
6’	1.14	17.93	1.28	16.10
1”	4.64	98.66	4.79	99.27
2”	1.79, 1.99	35.38	1.94, 2.16	35.06
3”	4.05	61.41	3.93	66.41
4”-OCH_3	3.28	55.79	-	-
4”	2.81	81.80	3.18	72.45
5”	4.02	63.10	4.06	64.39
6”	1.10	17.61	1.22	16.41

Supplementary Fig. 18. NMR data for kasufungin continued. (j) ^{1}H and ^{13}C NMR spectral data for kasufungin B in methanol-*d4*.

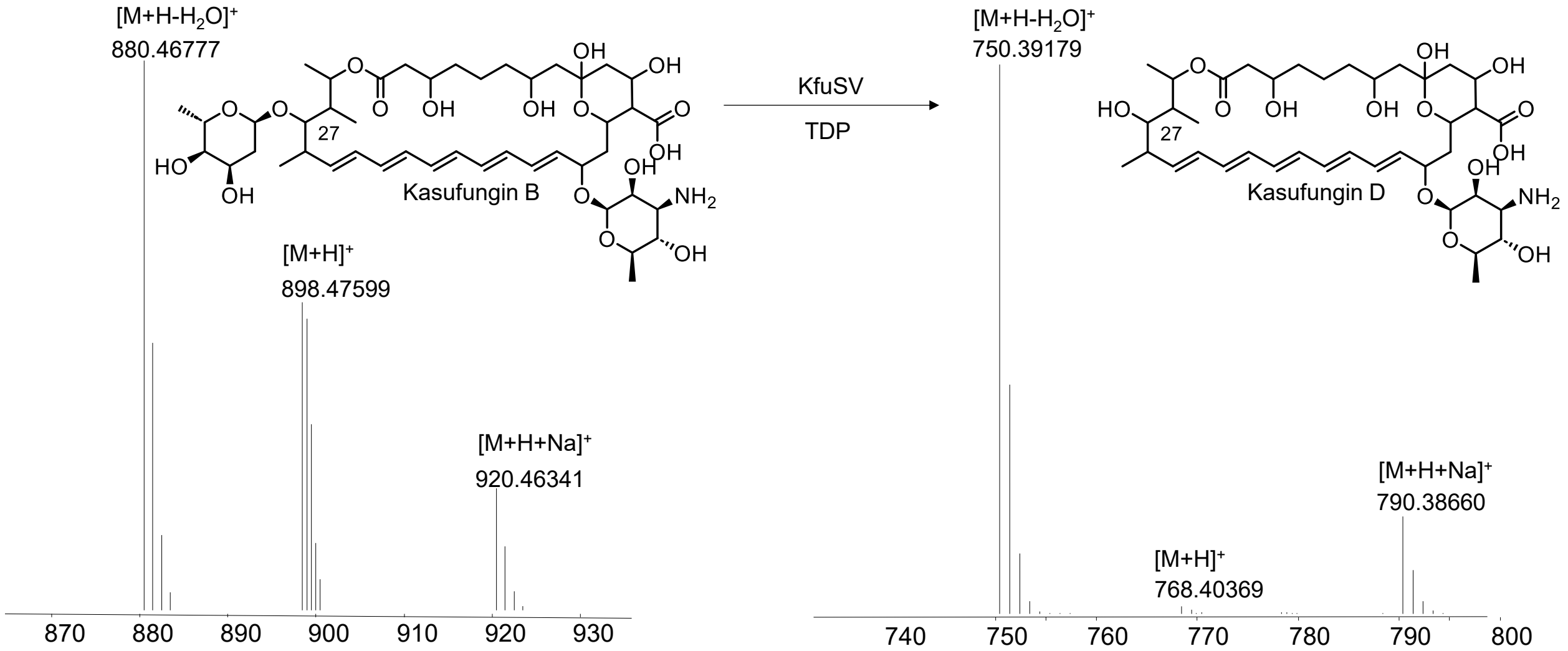

Supplementary Fig. 19. KfuSV catalysed reverse glycosylation of kasufungin B. The figure shows accurate mass of kasufungin B, calc. *m/z* = 898.4795 $[M+H]^+$, obs. *m/z* = 898.4759 $[M+H]^+$ and kasufungin D, calc. *m/z* = 768.4165 $[M+H]^+$, obs. *m/z* = 768.40369 $[M+H]^+$.

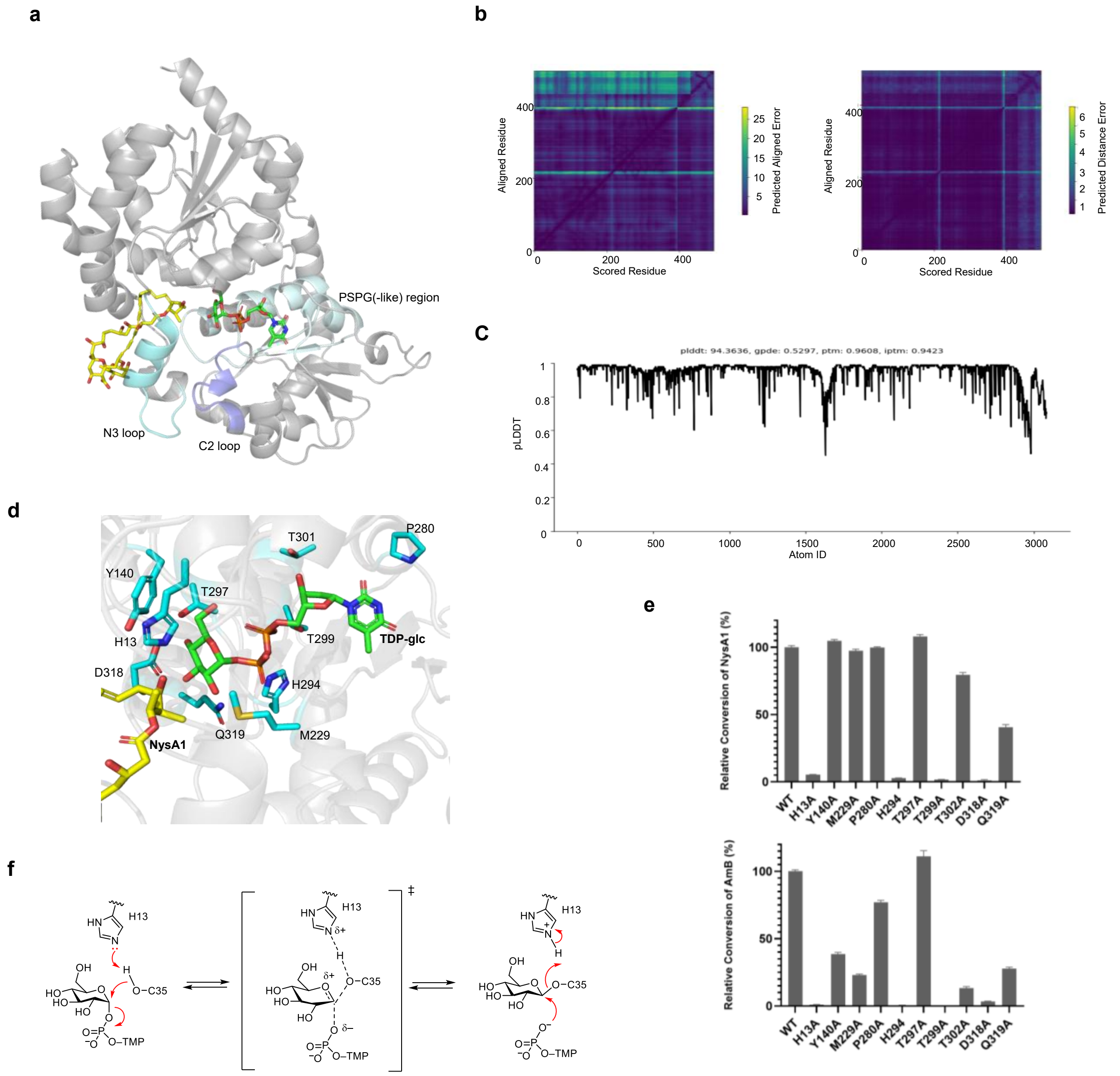

Supplementary Fig. 20. Computational model of KfuSV with TDP-glucose and NysA1. (a) AlphaFold model visualised in PyMOL highlighting structural motifs common to GT-B type 1 family enzymes, including N3 loop, C2 loop, and PSPG/PSPG-like region. **(b)** predicted distance and predicted alignment plots by residue for the computational model. **(c)** Predicted local distance difference (pLDDT) plot of confidence by atom ID. **(d-f)** Probing structure-activity relationships of active site residues in KfuSV. **(d)** Highlighted residues in the active site of KfuSV subjected to an alanine scan. **(e)** Relative conversions of the glucosylationreactions of NysA1 or AmB with each KfuSV mutants, compared to the wild type enzyme. **(f)** Proposed mechanism of action of KfuSV, showing the role of H13 as a general base and the formation of an oxocarbenium ion-like transition state to give inversion of the stereocenter.[42, 68] The H13 residue is positioned within 2.6 Å of the polyene C35-OH.

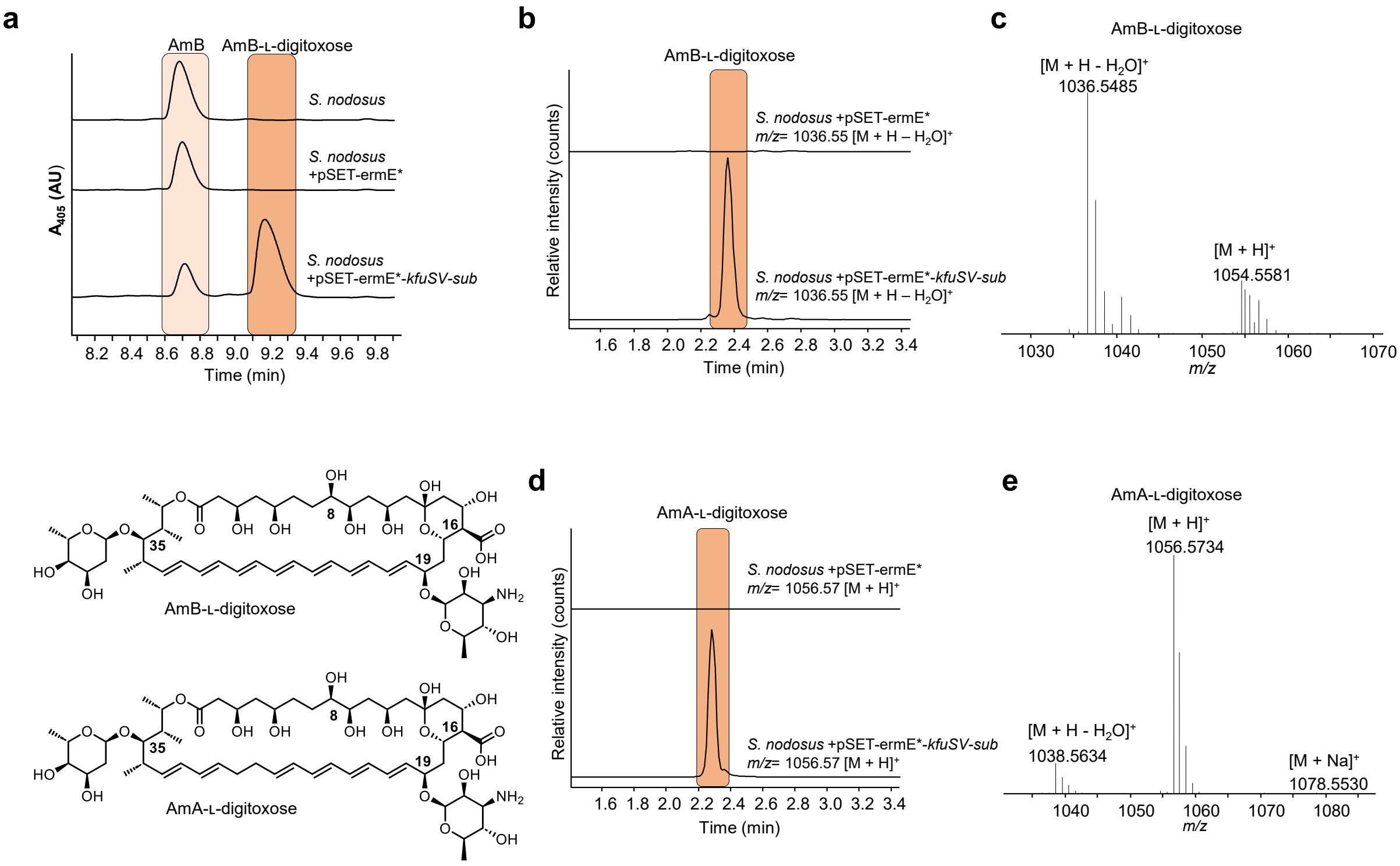

Supplementary Fig. 21. *In vivo* production of AmB-L-digitoxose derivative from heterologous expression of the KfuSV sugar subcluster. **(a)** RP-HPLC analysis of a novel glycosylated AmB derivative produced by *kfuSV-sub* expression in *S. nodosus* (compared to no production in *S. nodosus* alone or *S. nodosus* with an empty plasmid), observed at 405 nm. **(b)** Extracted ion chromatogram (ESI positive mode) corresponding to AmB-L-digitoxose. **(c)** Accurate mass analysis of AmB-L-digitoxose, calculated *m/z* = 1054.5581 $[M+H]^+$. **(d)** Extracted ion chromatogram (ESI positive mode) corresponding to AmA-L-digitoxose production. **(e)** Accurate mass analysis of AmA-L-digitoxose (congener with single C28-C29 single bond), calculated *m/z* = 1056.5738.

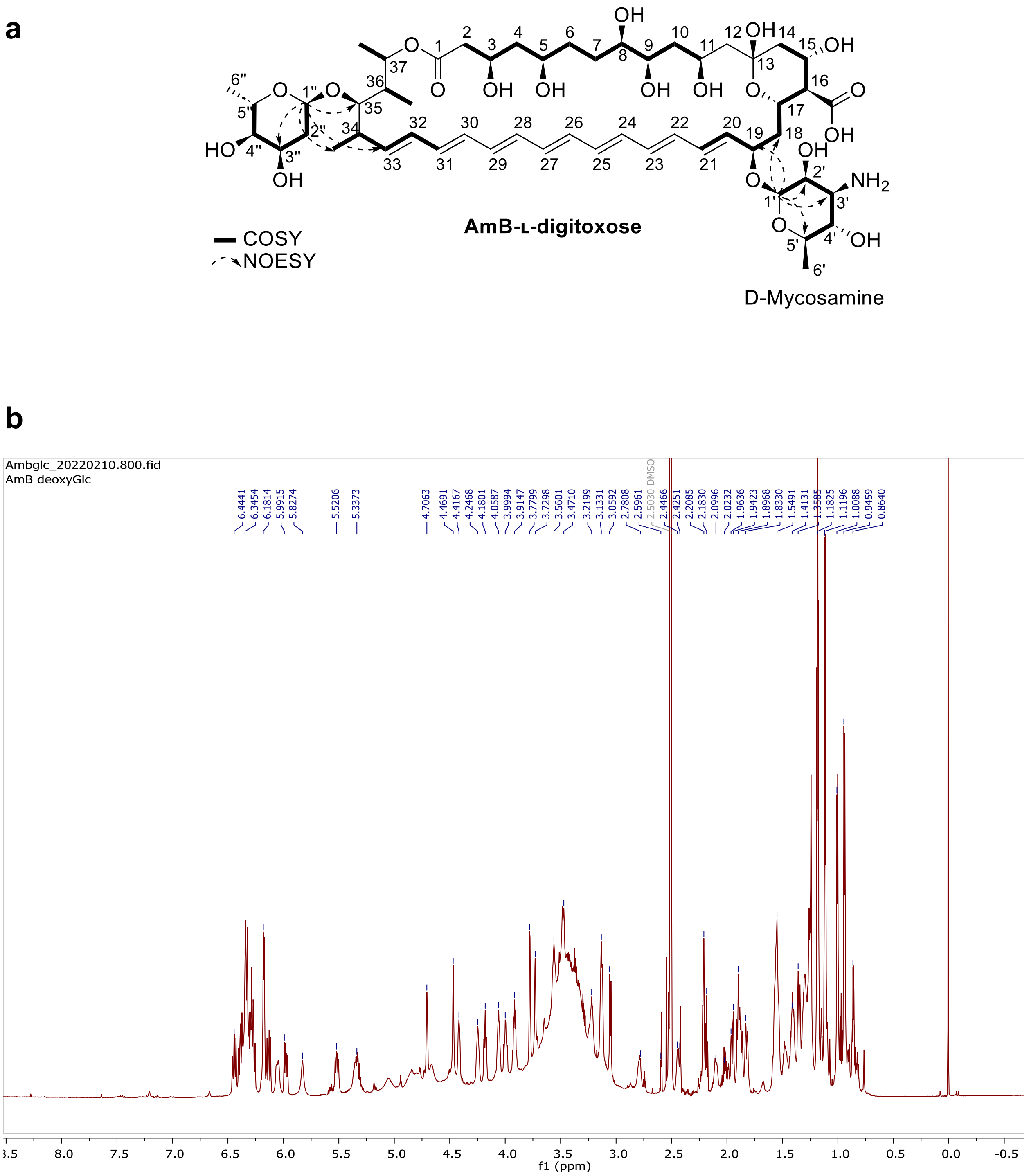

Supplementary Fig. 22. NMR data for AmB-ʟ-digitoxose. (a) Key NMR correlations establishing the structure of AmB-ʟ-digitoxose. **(b)** ^{1}H NMR spectrum for AmB-ʟ-digitoxose in DMSO-*d6*.

c

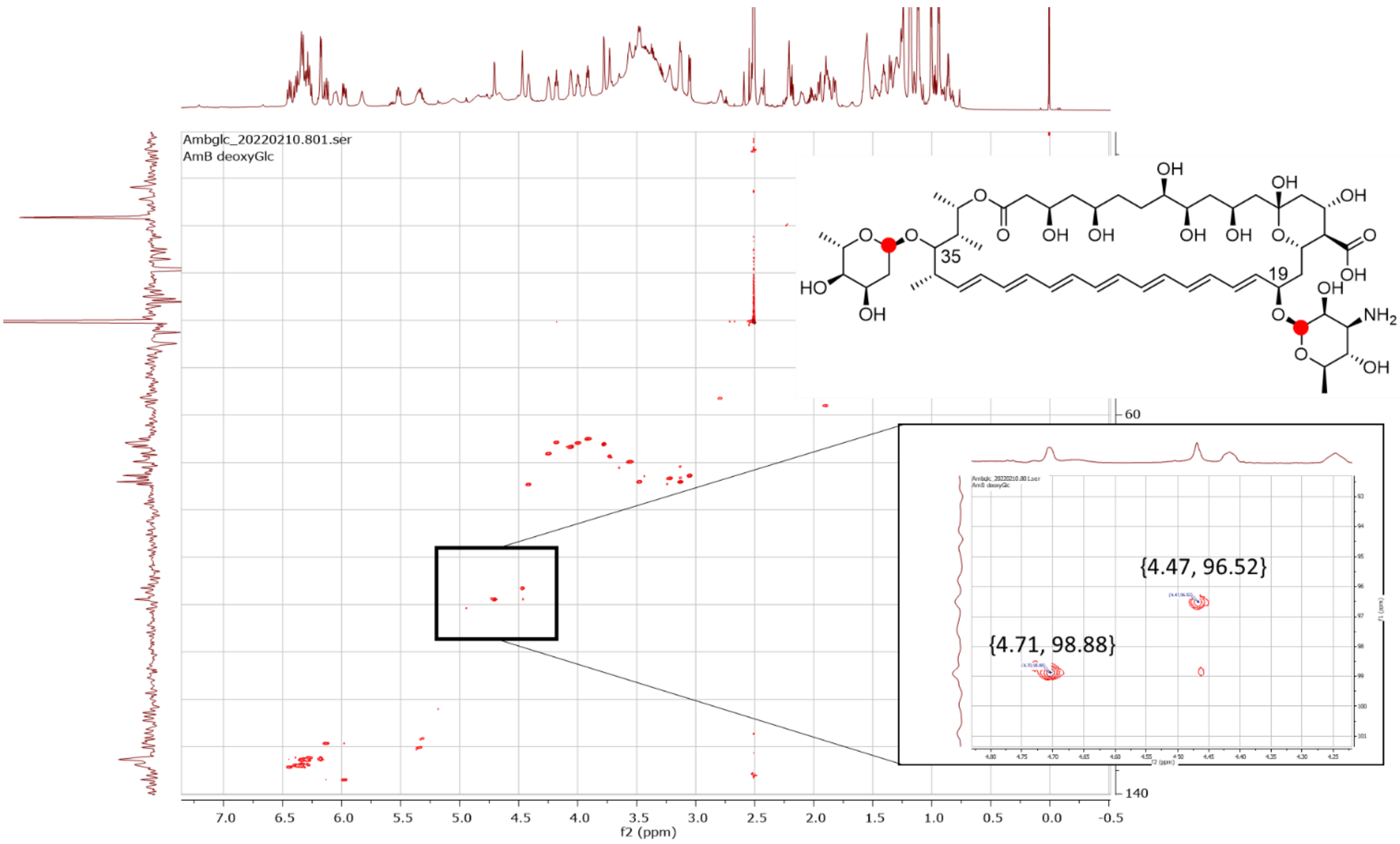

Supplementary Fig. 22. NMR data for AmB-L-digitoxose continued. (c) ^{1}H-^{13}C HSQC spectrum of AmB-L-digitoxose. Two anomeric signals indicate that there are 2 sugar moieties in AmB-L-digitoxose.

d

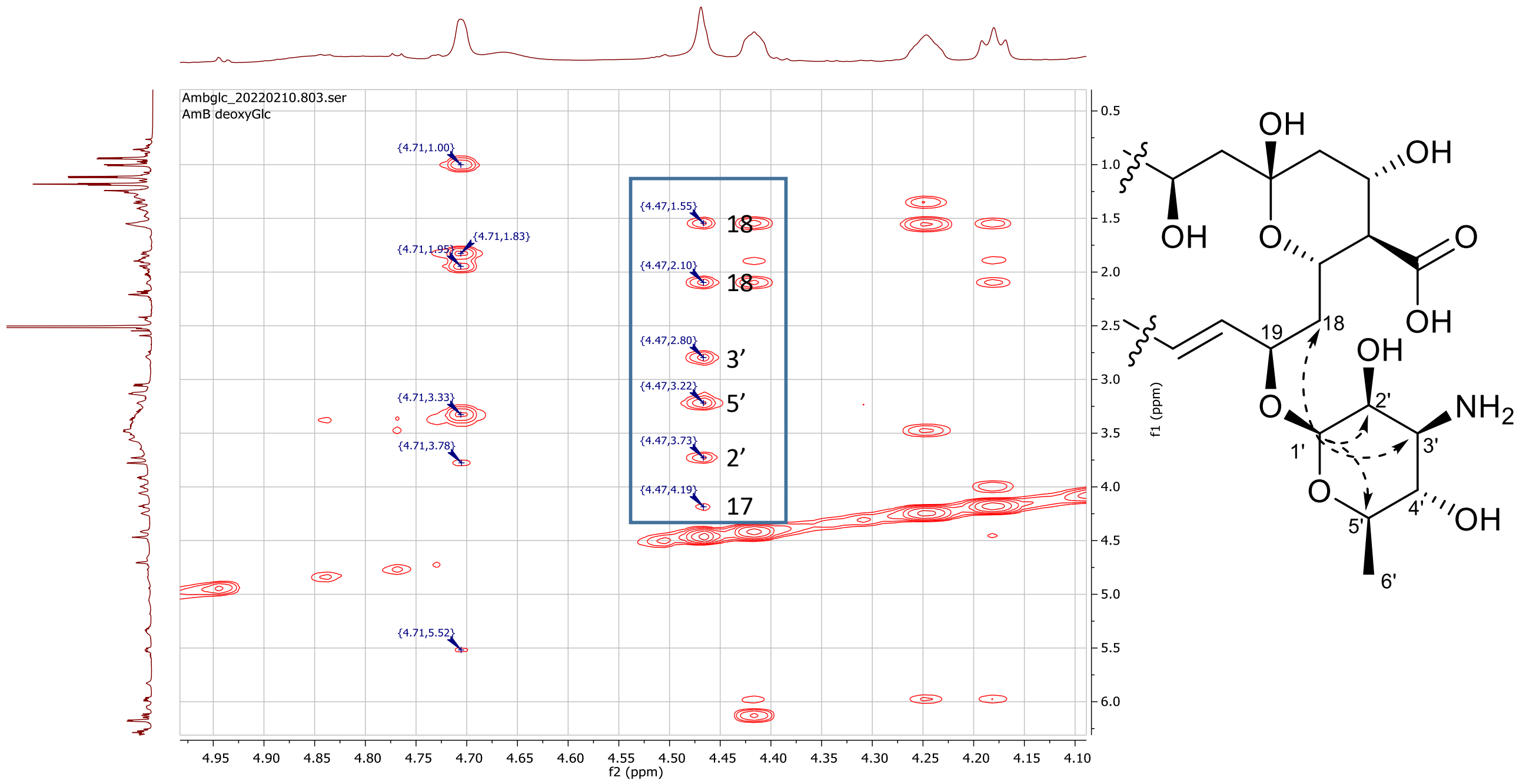

e

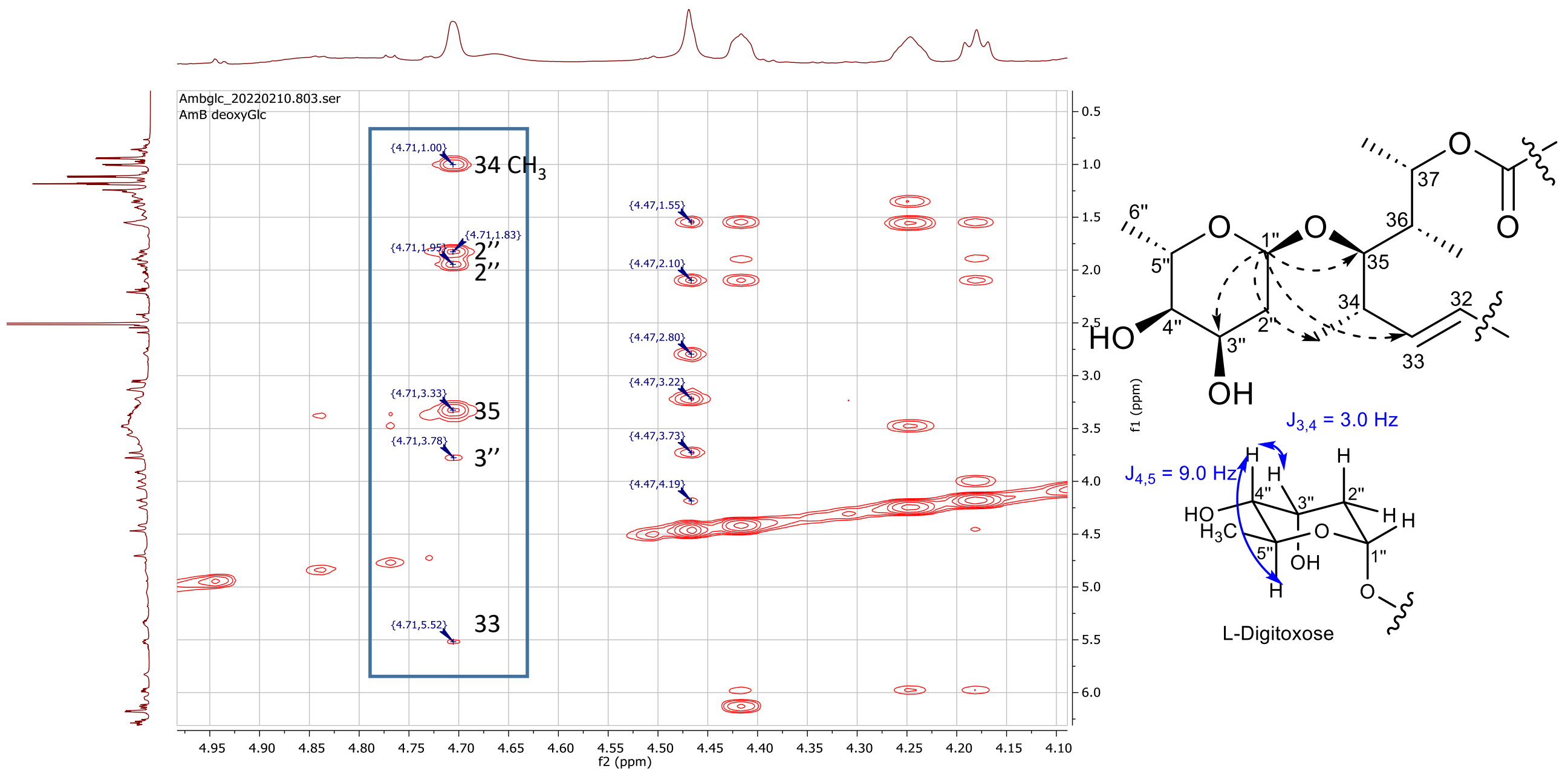

Supplementary Fig. 22. NMR data for AmB-L-digitoxose continued. (d-e) show enlarged sections of ^{1}H-^{1}H ROESY spectrum of AmB-L-digitoxose. **(d)** The correlation from H1′ confirms the position and relative stereochemistry of the D-mycosamine. **(e)** Correlations from H1′′ confirms the position of the sugar and coupling constants of $J_{4,3}$ at 3.0 Hz and $J_{4,5}$ at 9.0 Hz suggest that it is an L-digitoxose.

f

Proton	Reported AmB1 [43]– DMSO-*d6*	Found in AmB-L-digitoxose – DMSO-*d6*
2	2.19	2.21
3	4.07	4.06
4	1.41 , 1.36	1.43, 1.39
5	3.56	3.56
6	1.41, 1.31	1.41, 1.35
7	1.58, 1.30	1.56
8	3.12	3.12
9	3.48	3.47
10	1.57, 1.35	1.55, 1.35
11	4.26	4.24
12	1.56, 1.55	1.55
14	1.87, 1.11	1.86, 1.11
15	4.01	4.01
16	1.87	1.88
17	4.19	4.18
18	2.16, 1.52	1.54, 2.09
19	4.41	4.41
20	6.01	5.97
21		6.13
22-30		6.27-6.38
31		6.17
32	6.12	6.05
33	5.45	5.51
34	2.30	2.44
34-CH_3	1.05	1.00
35	3.11	3.33
36	1.75	1.89
36-CH_3	0.93	0.95
37	5.21	4.95
37-CH_3	1.13	1.17
1’	4.51	4.47
2’	3.75	3.71
3’	2.79	2.79
4’	3.16	3.13
5’	3.25	3.20
6’	1.18	1.18
1’’		4.71
2’’		1.85, 1.95
3’’		3.78
4’’		3.05
5’’		3.91
6’’		1.11

Supplementary Fig. 22. NMR data for AmB-L-digitoxose continued. (f) shows ^{1}H NMR spectral data for AmB-L-digitoxose in DMSO-*d6*

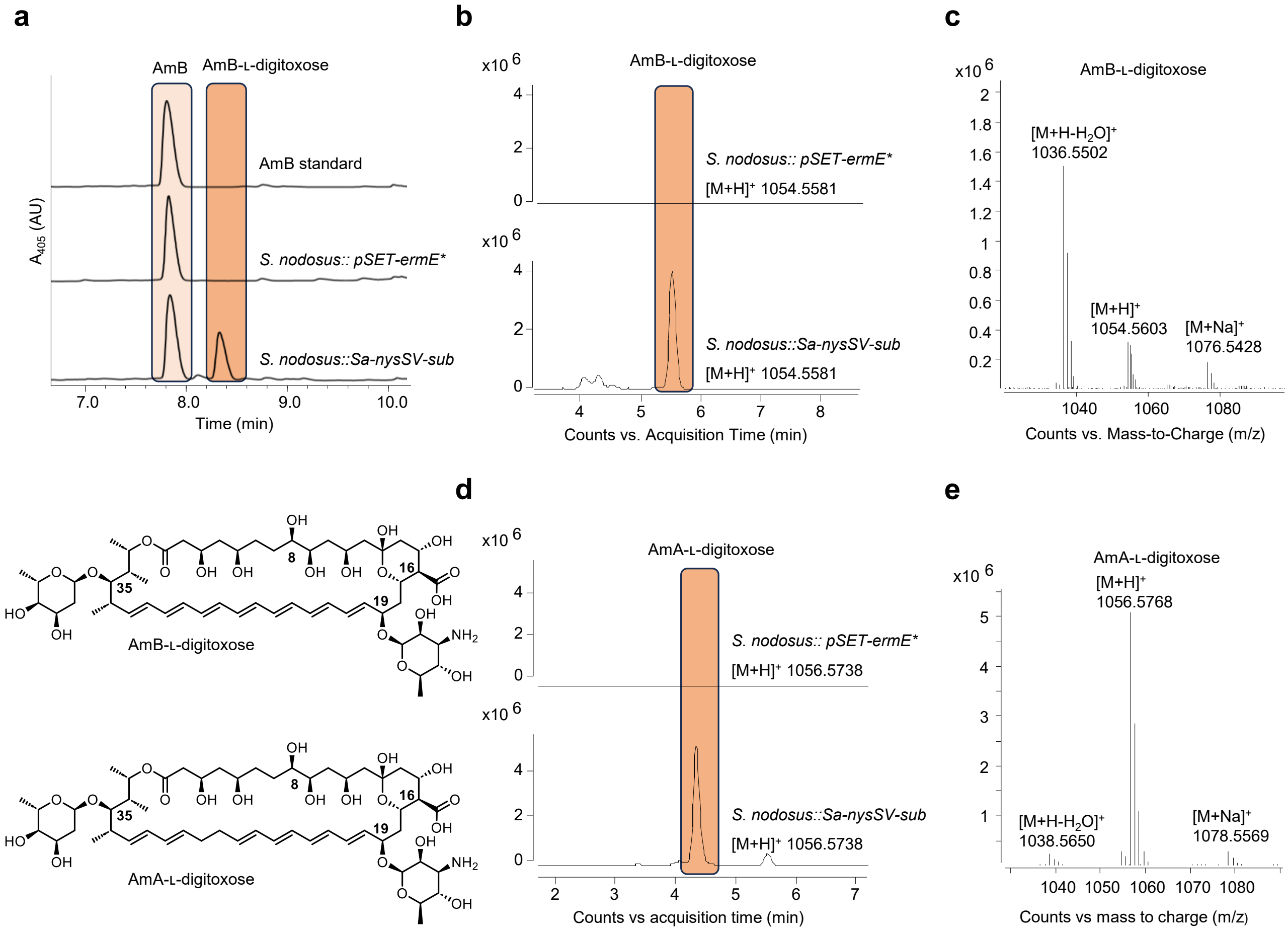

Supplementary Fig. 23. ***In vivo*** **production of AmB-L-digitoxose derivative from heterologous expression of the SaNysSV sugar subcluster**. **(a)** RP-HPLC analysis of a novel glycosylated AmB derivative produced by *Sa-nysSV-sub* expression in *S. nodosus* (compared to no production *S. nodosus* with an empty plasmid and AmB standard), observed at 405 nm. **(b)** Extracted ion chromatogram (ESI positive mode) corresponding to AmB-L-digitoxose. **(c)** Accurate mass analysis of AmB-L-digitoxose, calculated *m/z* = 1054.5581 $[M+H]^+$, observed *m/z* = 1054.5603. **(d)** Extracted ion chromatogram (ESI positive mode) corresponding to AmA-L-digitoxose production. **(e)** Accurate mass analysis of AmA-L-digitoxose (congener with single C28-C29 single bond), calculated *m/z* = 1056.5738, observed *m/z* = 1056.5768.

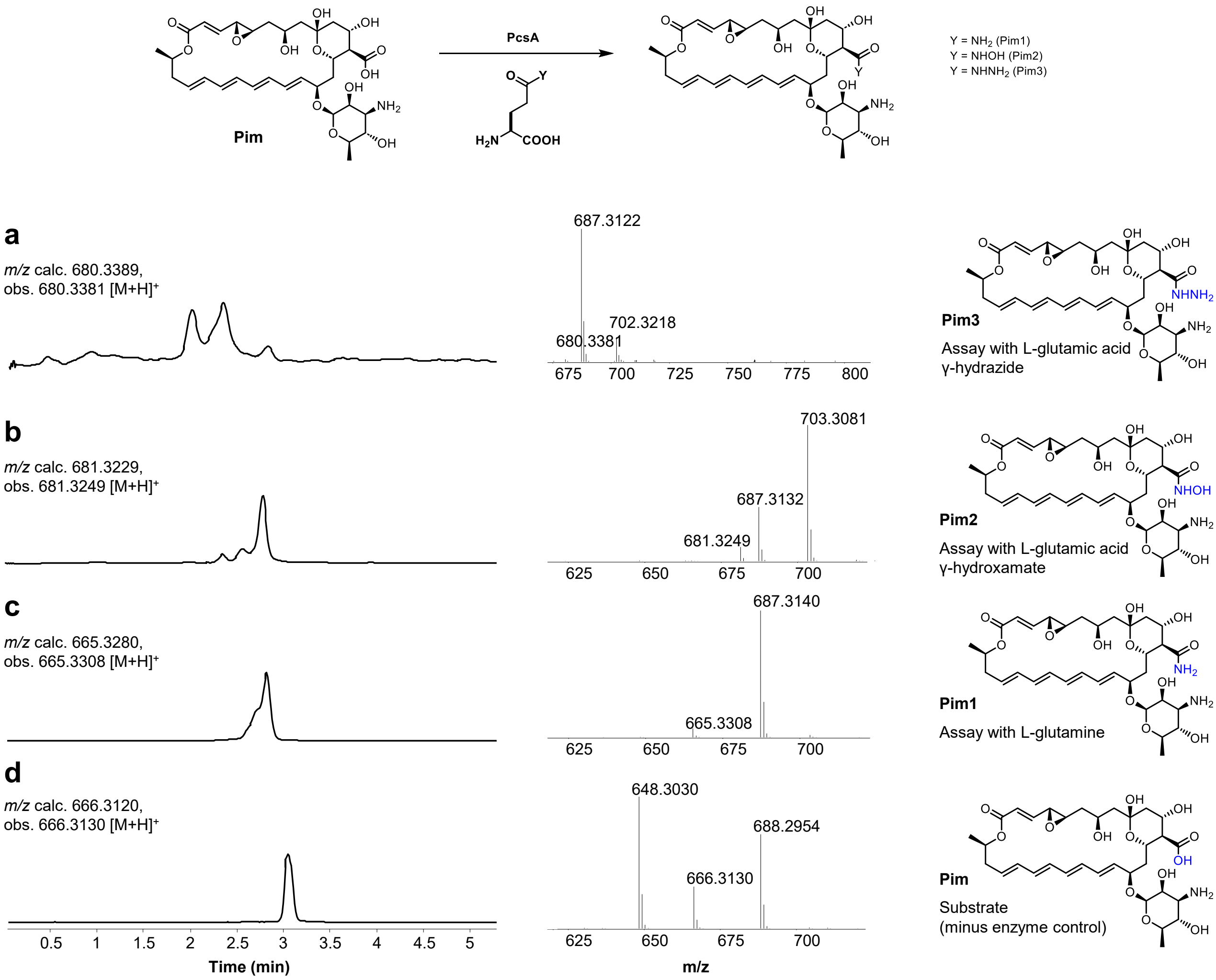

Supplementary Fig. 24. LC-HRMS analysis (ESI +ve) of PcsA catalysed reactions with pimaricin (Pim). Figure shows extracted ion chromatograms of **(a)** Pim3 with observed mass *m/z* $[M+H]^+$ 680.3381, **(b)** Pim2 with observed mass *m/z* $[M+H]^+$ 681.3249, **(c)** Pim1 as positive control with observed mass *m/z* $[M+H]^+$ 665.3308 and **(d)** Pim (substrate) in minus enzyme control with observed mass *m/z* $[M+H]^+$ 666.3130.

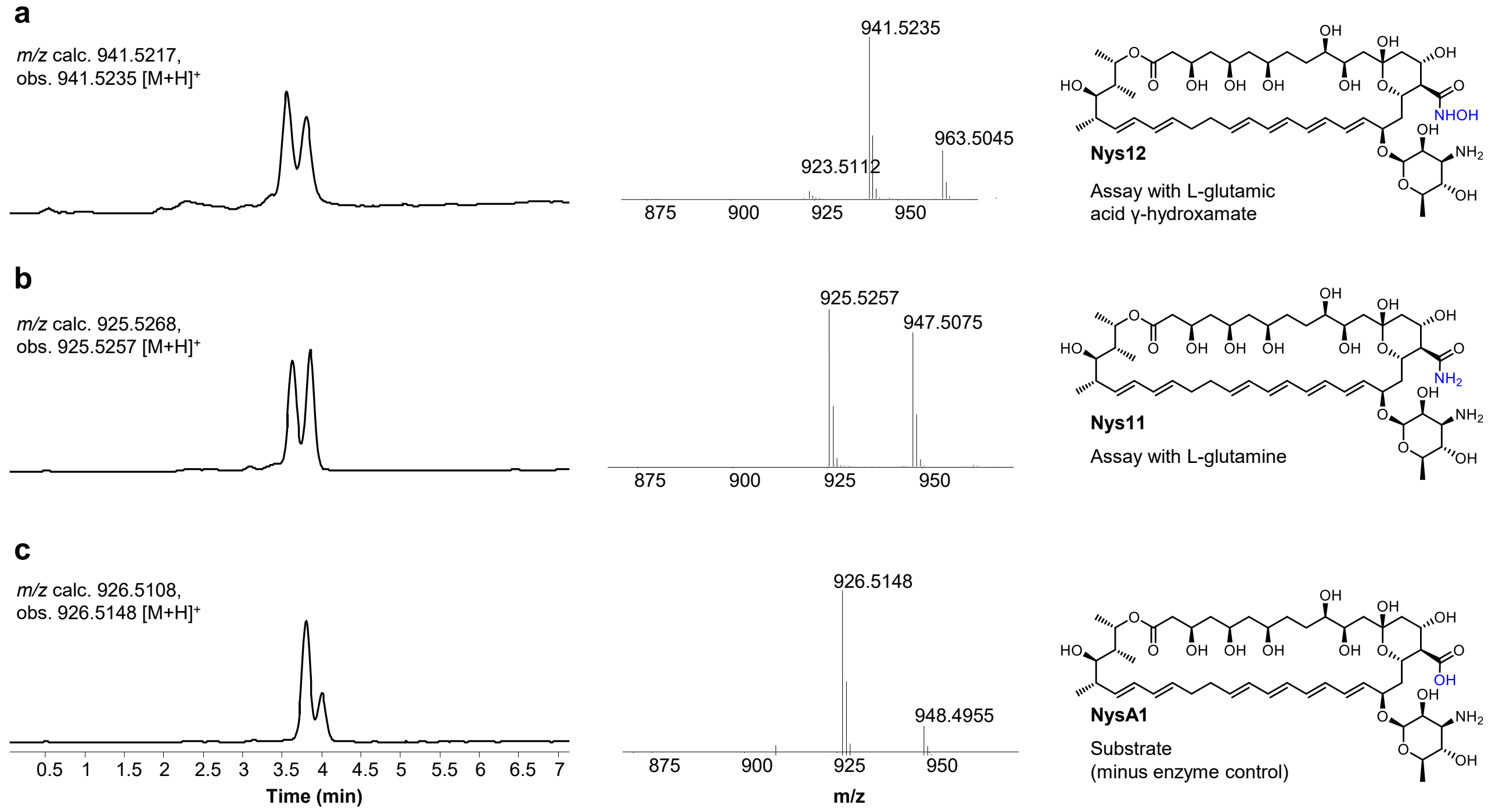

Supplementary Fig. 25. LC-HRMS analysis (ESI +ve) of PcsA catalysed formation of NysA1 derivatives. Figure shows extracted ion chromatograms of **(a)** Nys12 (enzymatic product) with observed mass *m/z* $[M+H]^+$ 941.5235, **(b)** Nys11 (enzymatic product) with observed mass *m/z* $[M+H]^+$ 925.5257 and **(c)** NysA1 (substrate) minus enzyme control with observed mass *m/z* $[M+H]^+$ 926.5148. The two peaks observed in each case are due to formation of the rotamers.

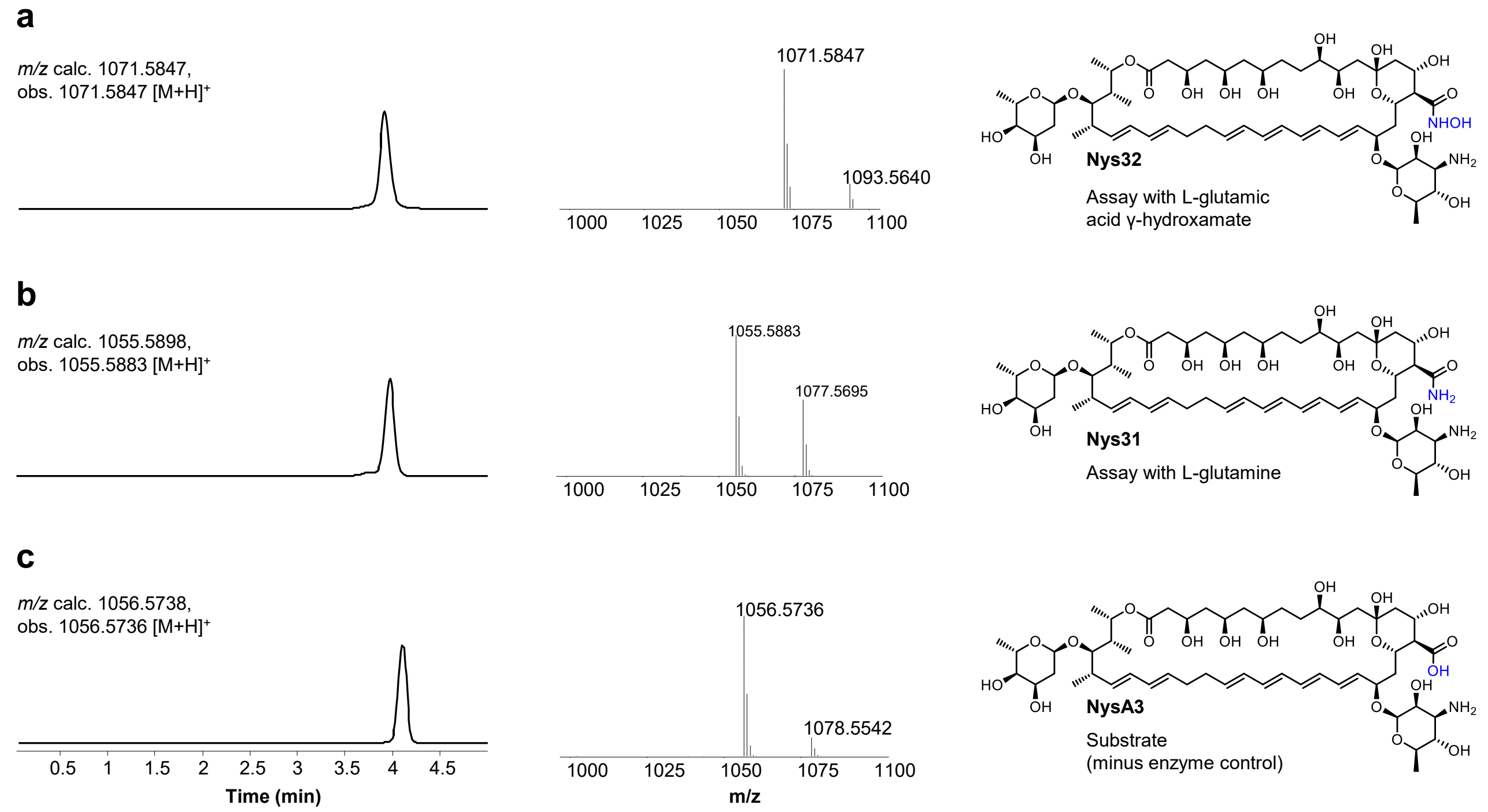

Supplementary Fig. 26. LC-HRMS analysis (ESI +ve) of PcsA catalysed formation of NysA3 derivatives. Figure shows extracted ion chromatograms of **(a)** Nys32 (enzymatic product) with observed mass *m/z* $[M+H]^+$ 1071.5847, **(b)** Nys31 (enzymatic product) with observed mass *m/z* $[M+H]^+$ 1055.5883 and **(c)** NysA3 (substrate) minus enzyme control with observed mass *m/z* $[M+H]^+$ 1056.5736.

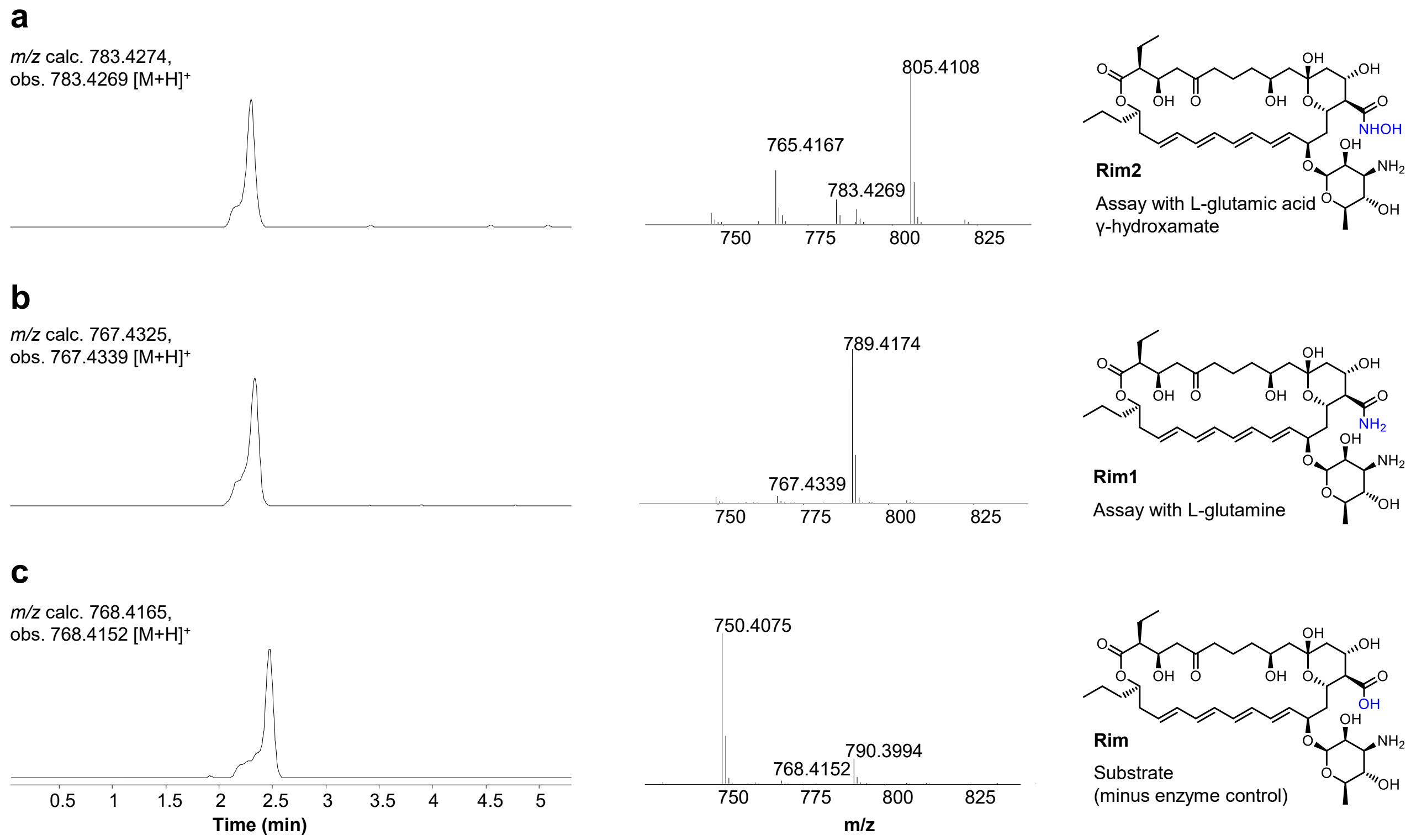

Supplementary Fig. 27. LC-HRMS analysis (ESI +ve) of PcsA catalysed reactions with rimocidin (Rim). Figure shows extracted ion chromatograms of **(a)** Rim2 with observed mass *m/z* $[M+H]^+$ 783.4269, **(b)** Rim1 with observed mass *m/z* $[M+H]^+$ 767.4339 and **(c)** rimocidin (Rim substrate) in minus enzyme control with observed mass *m/z* $[M+H]^+$ 768.4152.

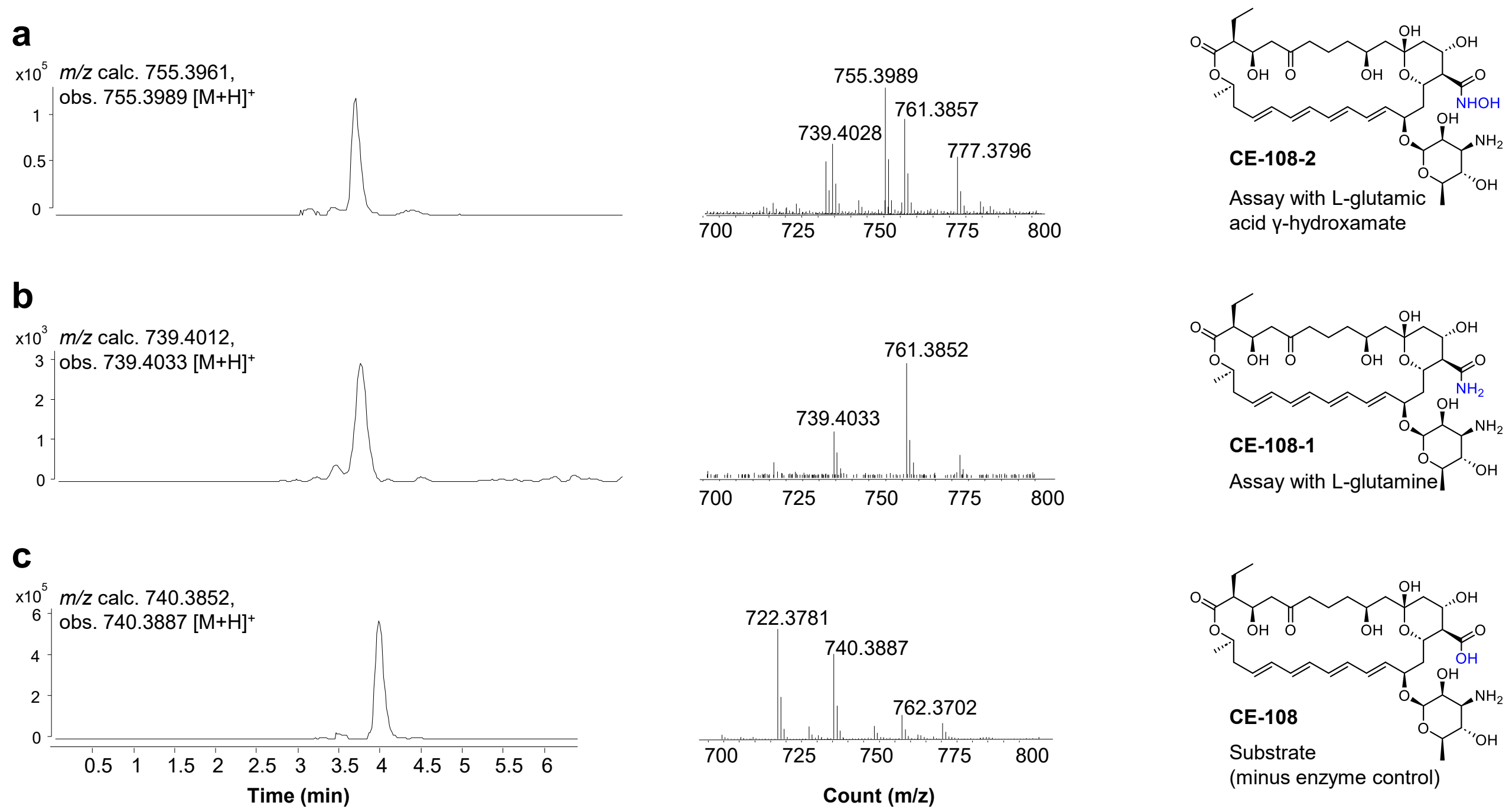

Supplementary Fig. 28. LC-HRMS analysis (ESI +ve) of PcsA catalysed formation of CE-108 derivatives. Figure shows extracted ion chromatograms of **(a)** CE-108-2 (enzymatic product) with observed mass *m/z* $[M+H]^+$ 755.3989, **(b)** CE-108-1 (enzymatic product) with observed mass *m/z* $[M+H]^+$ 739.4033 and **(c)** CE-108 (substrate) minus enzyme control with observed mass *m/z* $[M+H]^+$ 740.3887.

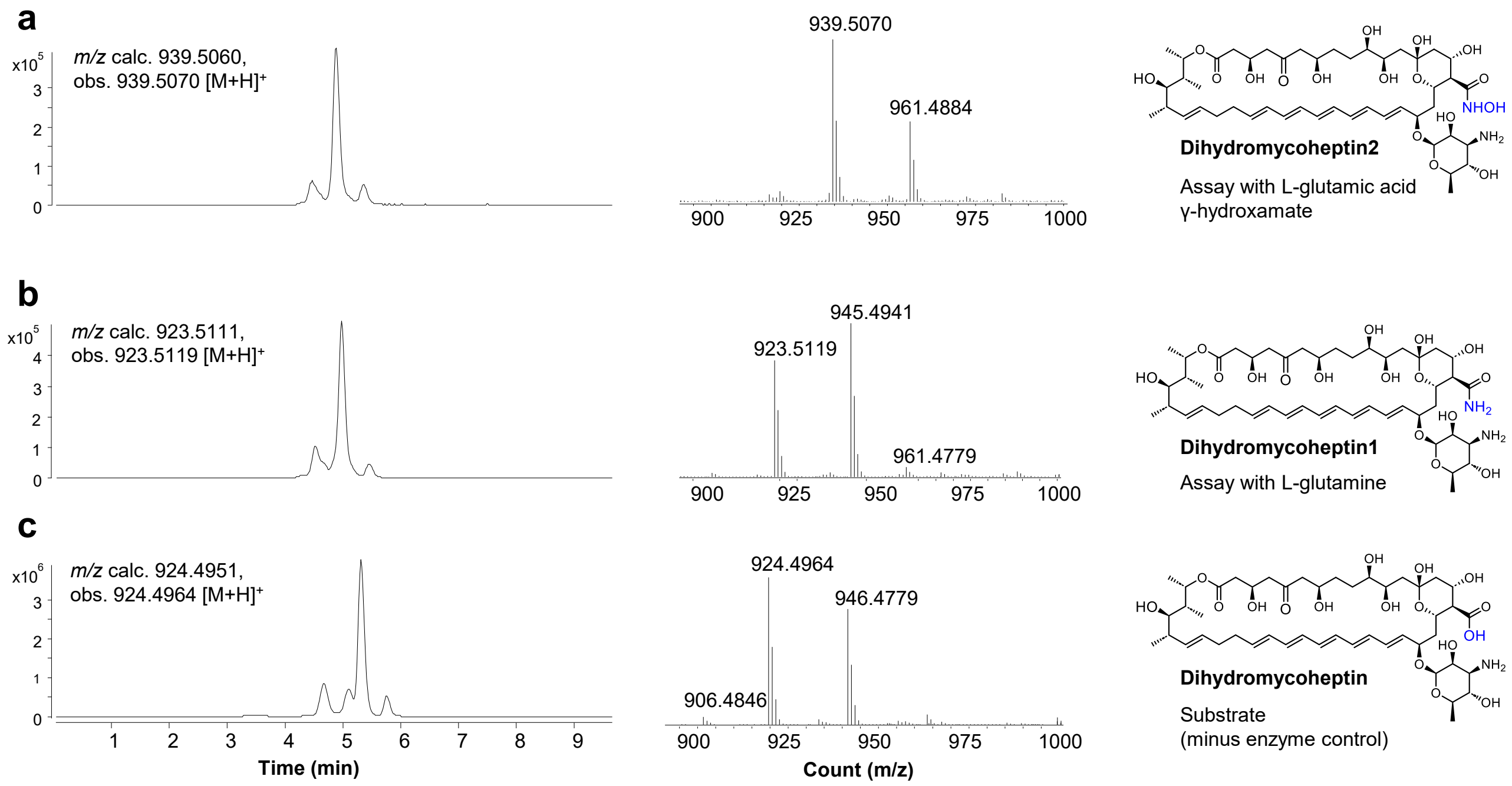

Supplementary Fig. 29. LC-HRMS analysis (ESI +ve) of PcsA catalysed formation of dihydromycoheptin derivatives. Figure shows extracted ion chromatograms of **(a)** dihydromycoheptin2 (enzymatic product) with observed mass *m/z* $[M+H]^+$ 939.5070, **(b)** dihydromycoheptin1 (enzymatic product) with observed mass *m/z* $[M+H]^+$ 923.5119 and **(c)** dihydromycoheptin (substrate) minus enzyme control with observed mass *m/z* $[M+H]^+$ 924.4964.

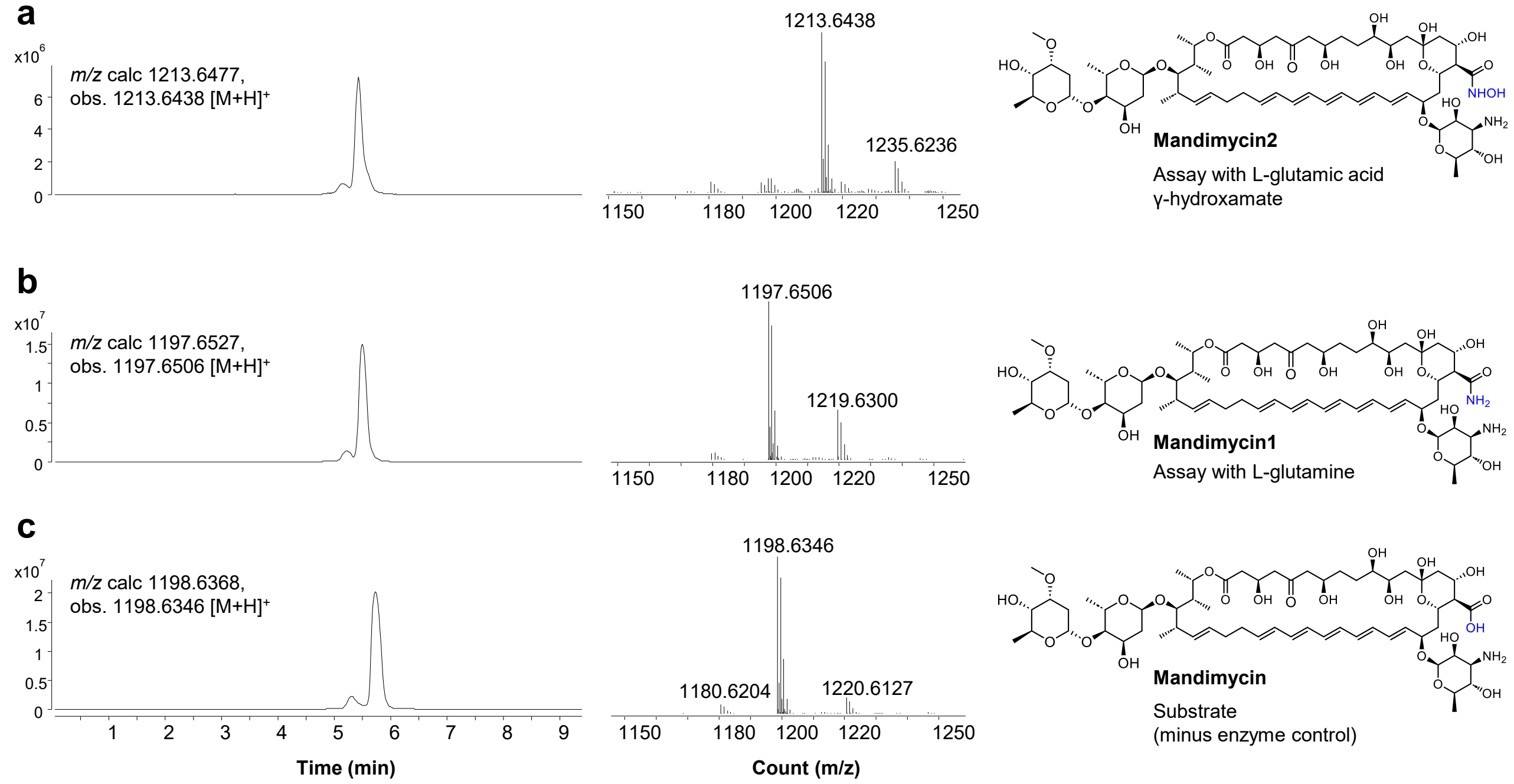

Supplementary Fig. 30. LC-HRMS analysis (ESI +ve) of PcsA catalysed formation of mandimycin derivatives. Figure shows extracted ion chromatograms of **(a)** mandimycin2 (enzymatic product) with observed mass *m/z* $[M+H]^+$ 1213.6438, (**b**) mandimycin1 (enzymatic product) with observed mass *m/z* $[M+H]^+$ 1197.6506 and **(c)** mandimycin (substrate) minus enzyme control with observed mass *m/z* $[M+H]^+$ 1198.6346.

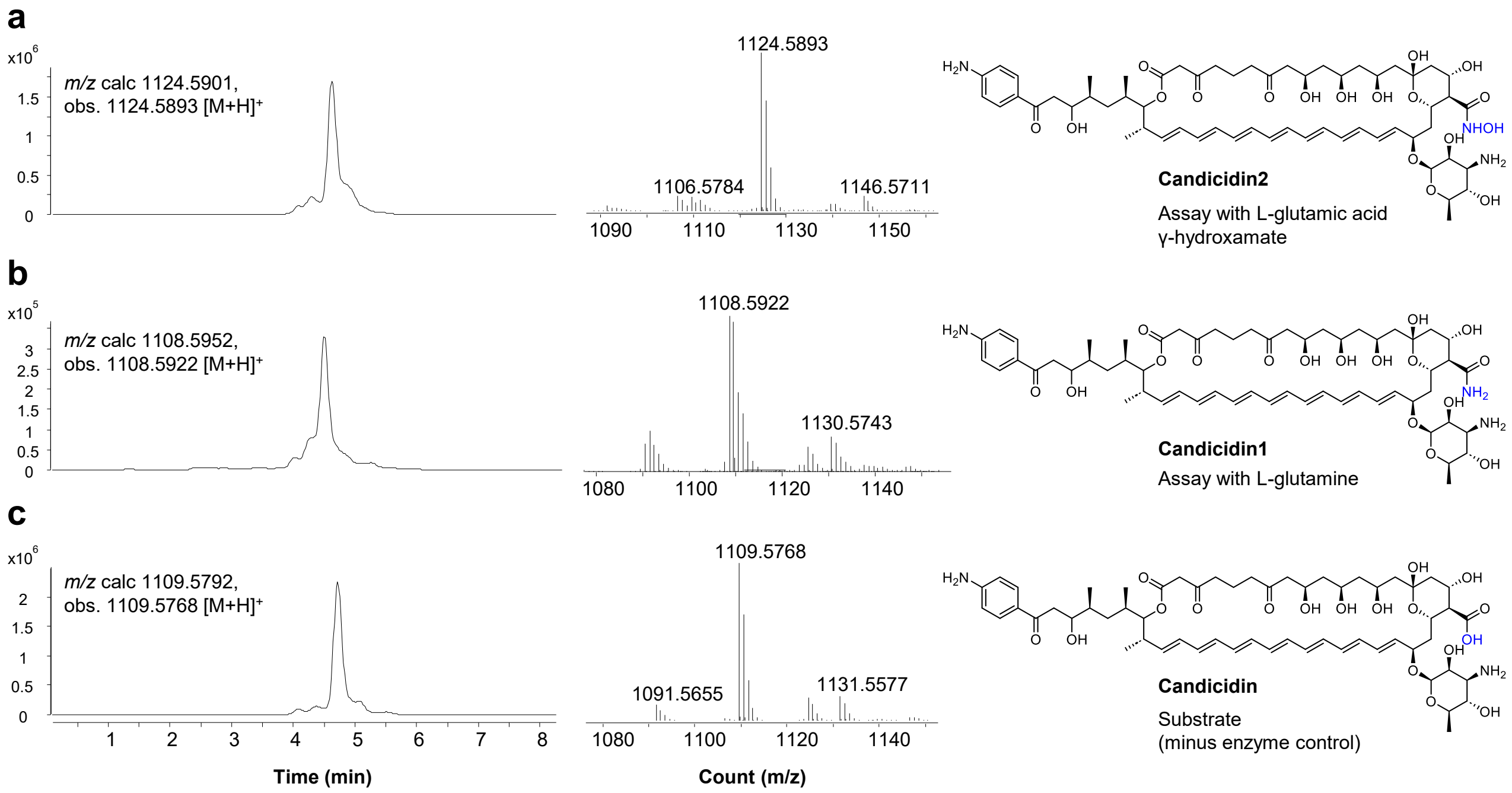

Supplementary Fig. 31. LC-HRMS analysis (ESI +ve) of PcsA catalysed formation of candicidin derivatives. Figure shows extracted ion chromatograms of **(a)** candicidin2 (enzymatic product) with observed mass *m/z* $[M+H]^+$ 1124.5893, **(b)** candicidin1 (enzymatic product) with observed mass *m/z* $[M+H]^+$ 1108.5922 and **(c)** candicidin (substrate) minus enzyme control with observed mass *m/z* $[M+H]^+$ 1109.5768.

a

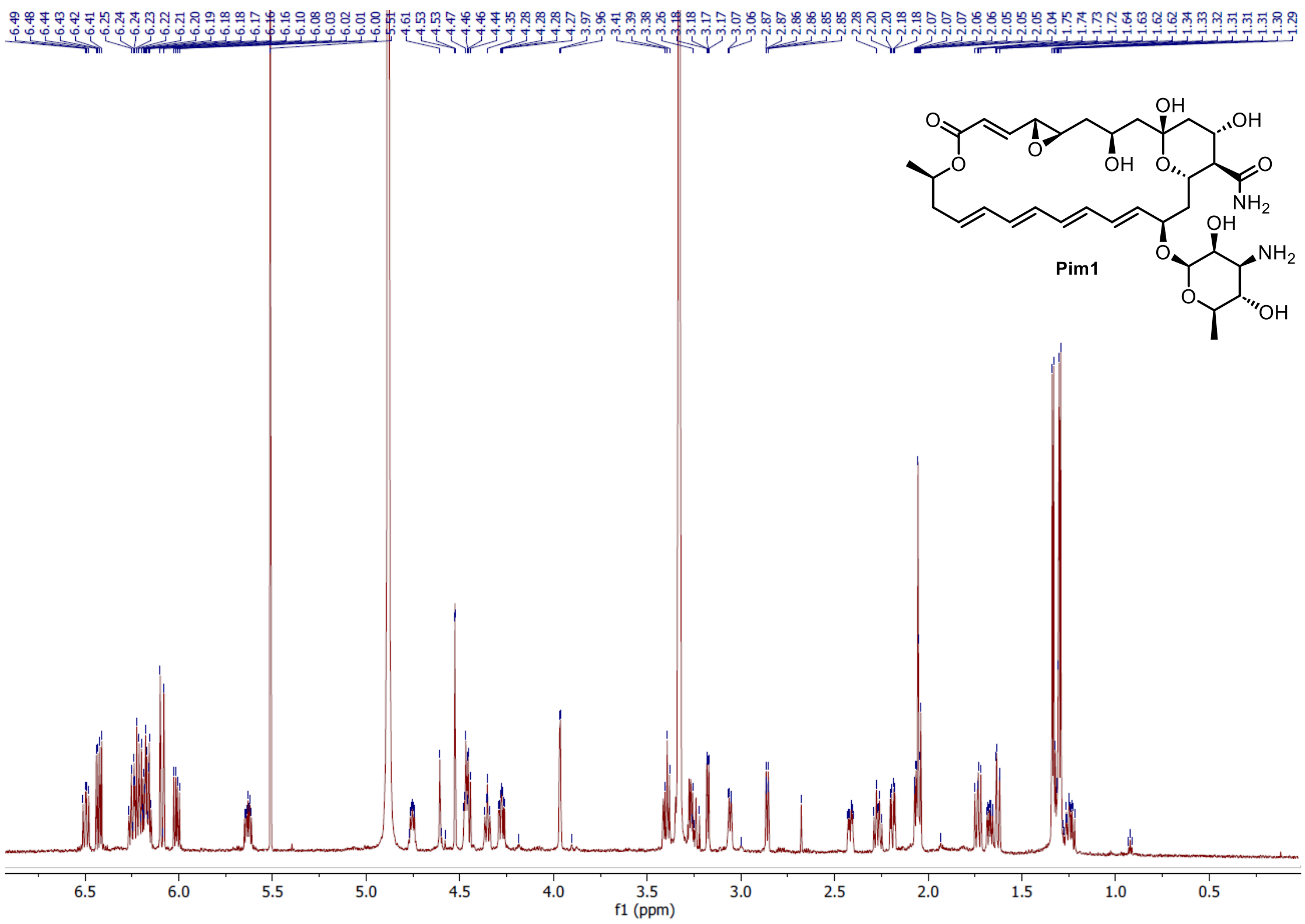

b

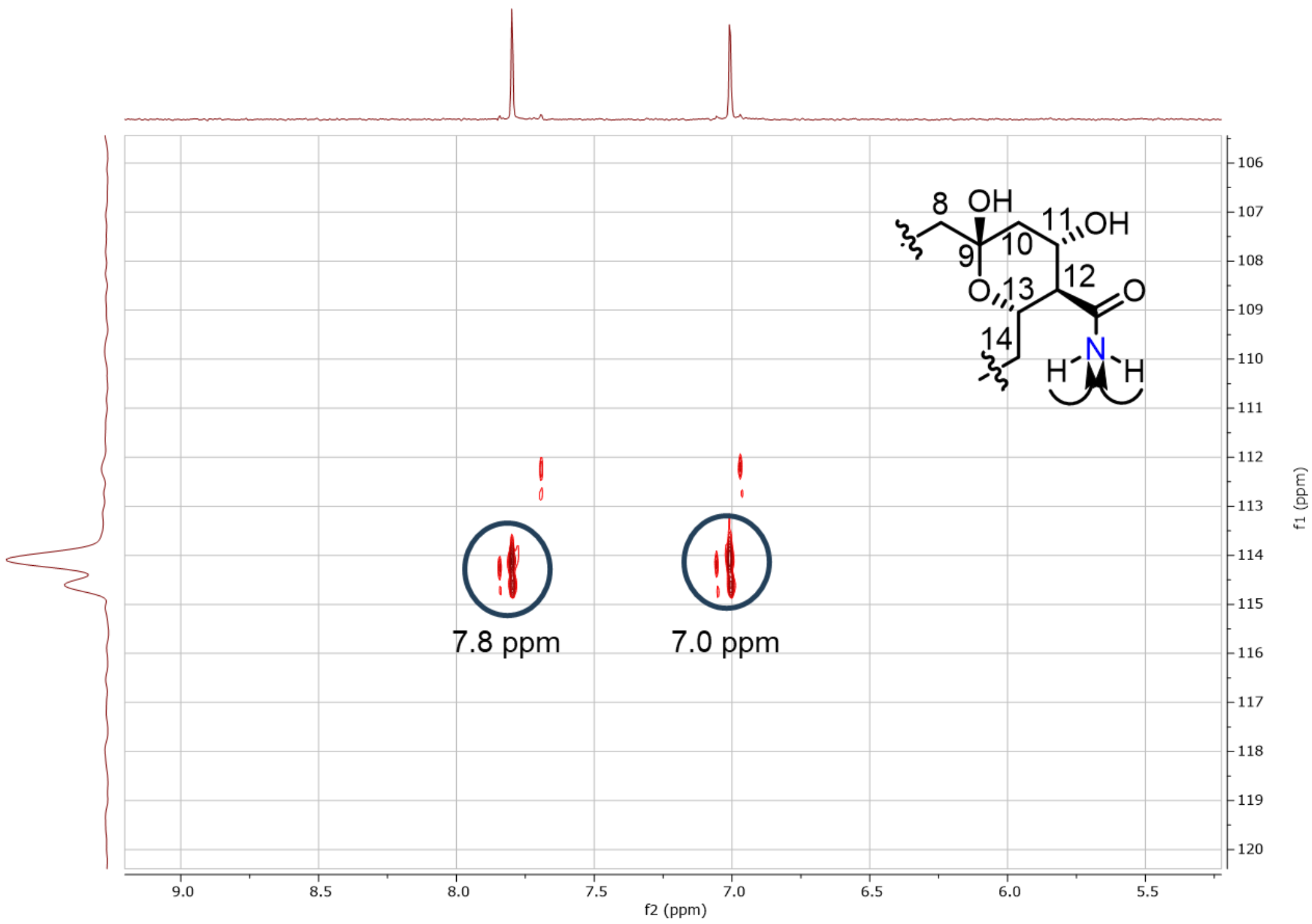

Supplementary Fig. 32. NMR data for Pim1. (a) shows ^{1}H-NMR of Pim1 in MeOD. **(b)** shows ^{1}H-^{15}N HSQC data for $^{15}NH_2$ labelled Pim1 in (10% D_2O, 90% MeOD). ^{15}N (y-axis) at 14 ppm and the corresponding amide protons (x-axis) at 7 and 7.8 ppm confirming NH_2 incorporation.

c

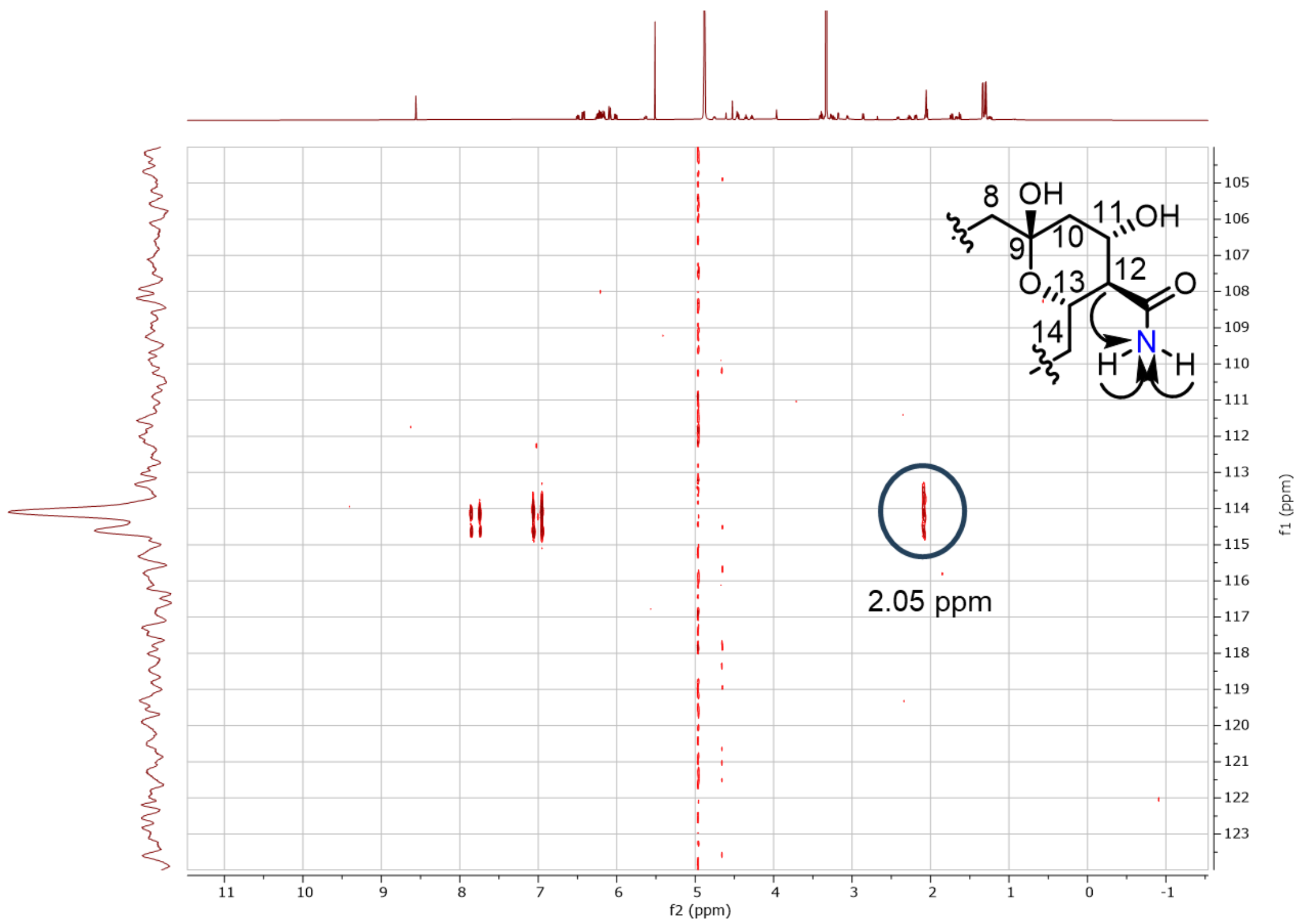

Supplementary Fig. 32. NMR data for Pim1 continued. (c) shows ^{1}H-^{15}N HMBC data for Pim1 in 10% D_2O, 90% MeOD. Cross peak for ^{15}N signal correlates with a proton at 2.05 ppm (signal for $^{1}H12$).

a

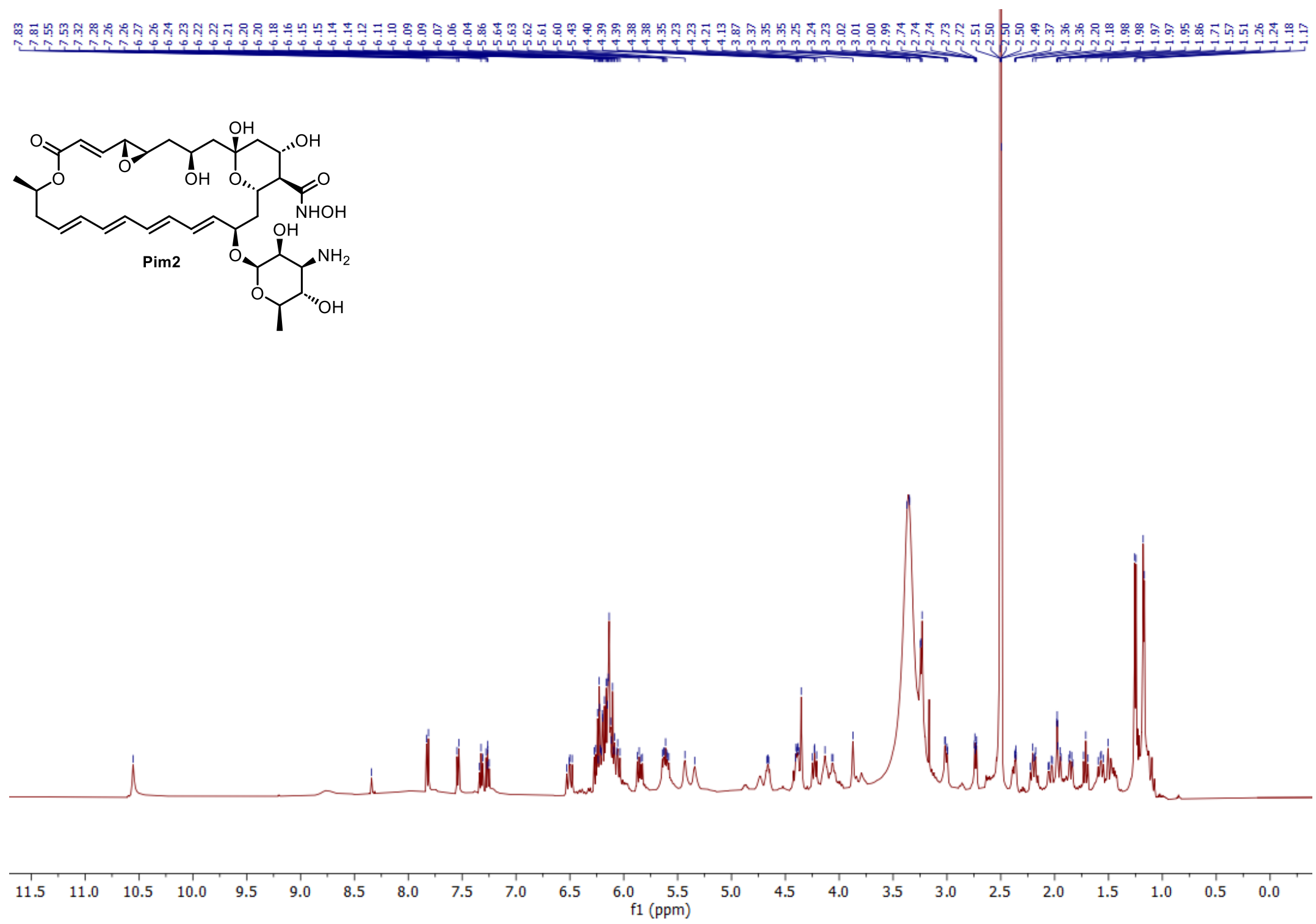

b

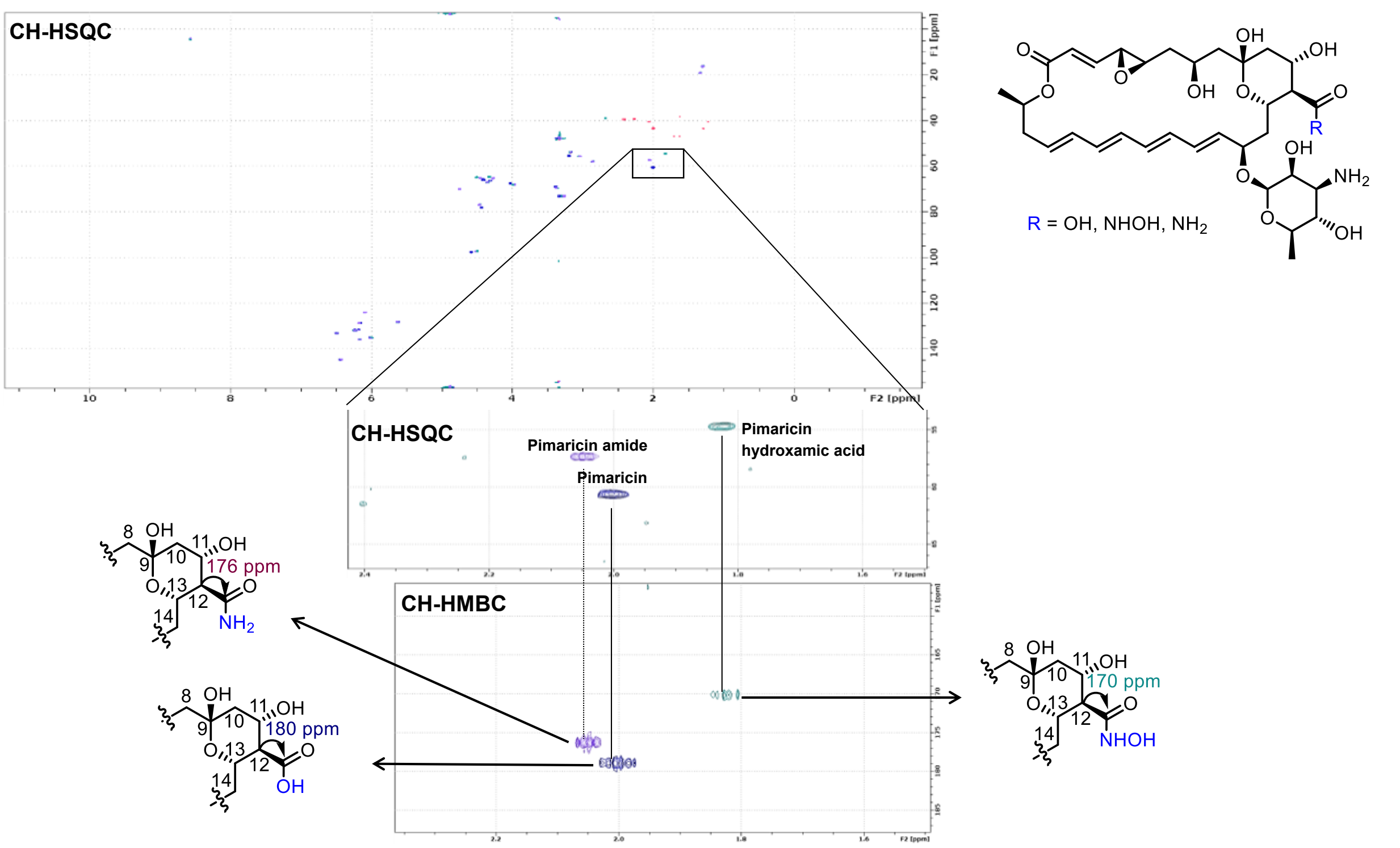

Supplementary Fig. 33. NMR data for Pim2. (a) shows ^{1}H-NMR of Pim2 in DMSO-*d6*. **(b)** shows pimaricin and its derivatives, a comparison of carbonyl chemical shifts. The top panel shows an overlay of CH-HSQC spectra for Pim, Pim1 and Pim2, further zoomed in showing ^{1}H12 signals for Pim2 (cyan), Pim (blue) and Pim1 (purple). The bottom panel shows overlay of CH-HMBC of Pim, its amide and hydroxamic acid derivatives, colour coded, linking ^{1}H12 to C27.

a

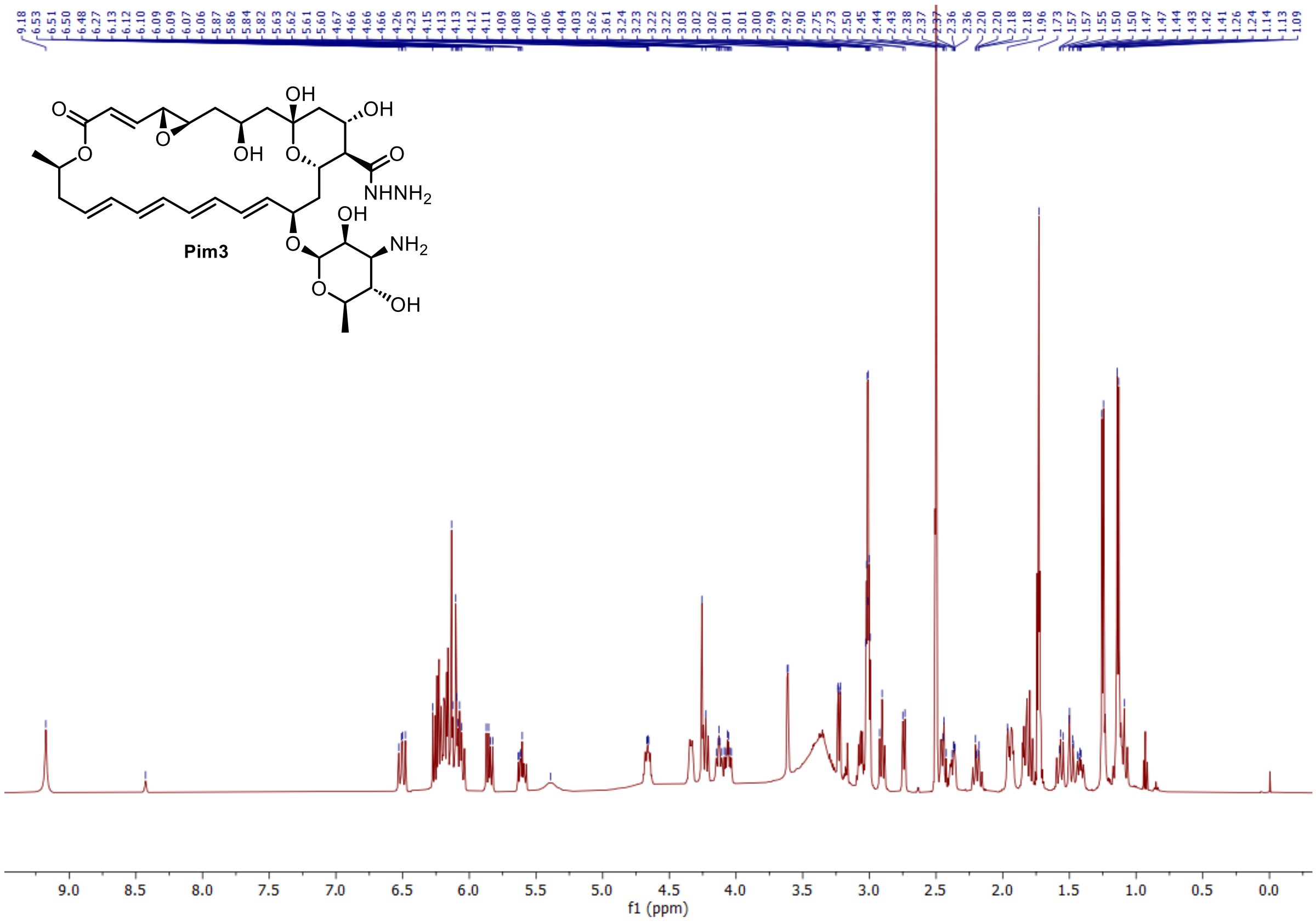

b

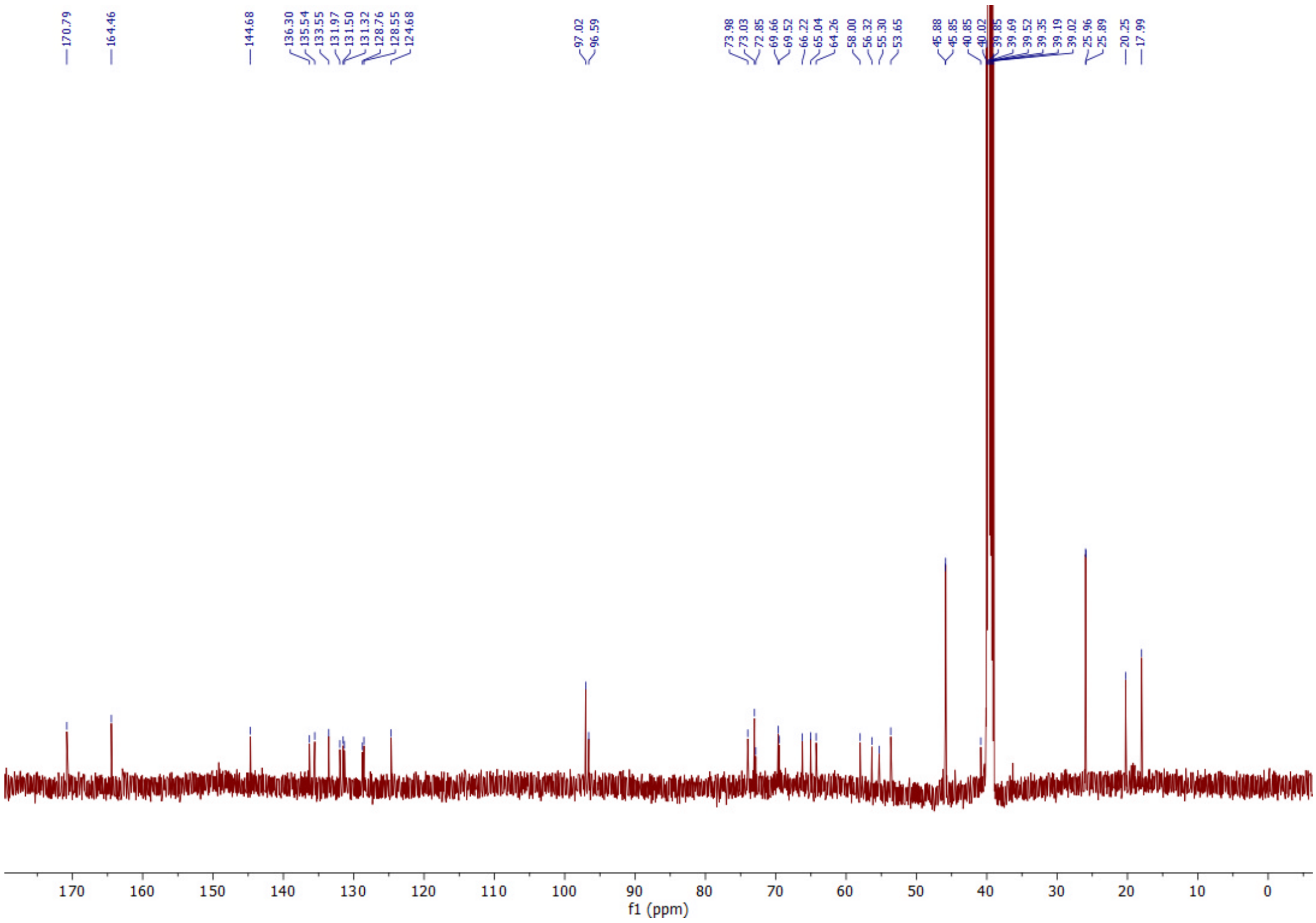

Supplementary Fig. 34. NMR data for Pim3. (a) shows ^{1}H-NMR of Pim3 in DMSO-*d6*. **(b)** shows ^{13}C-NMR of Pim3 in DMSO-*d6*.

c

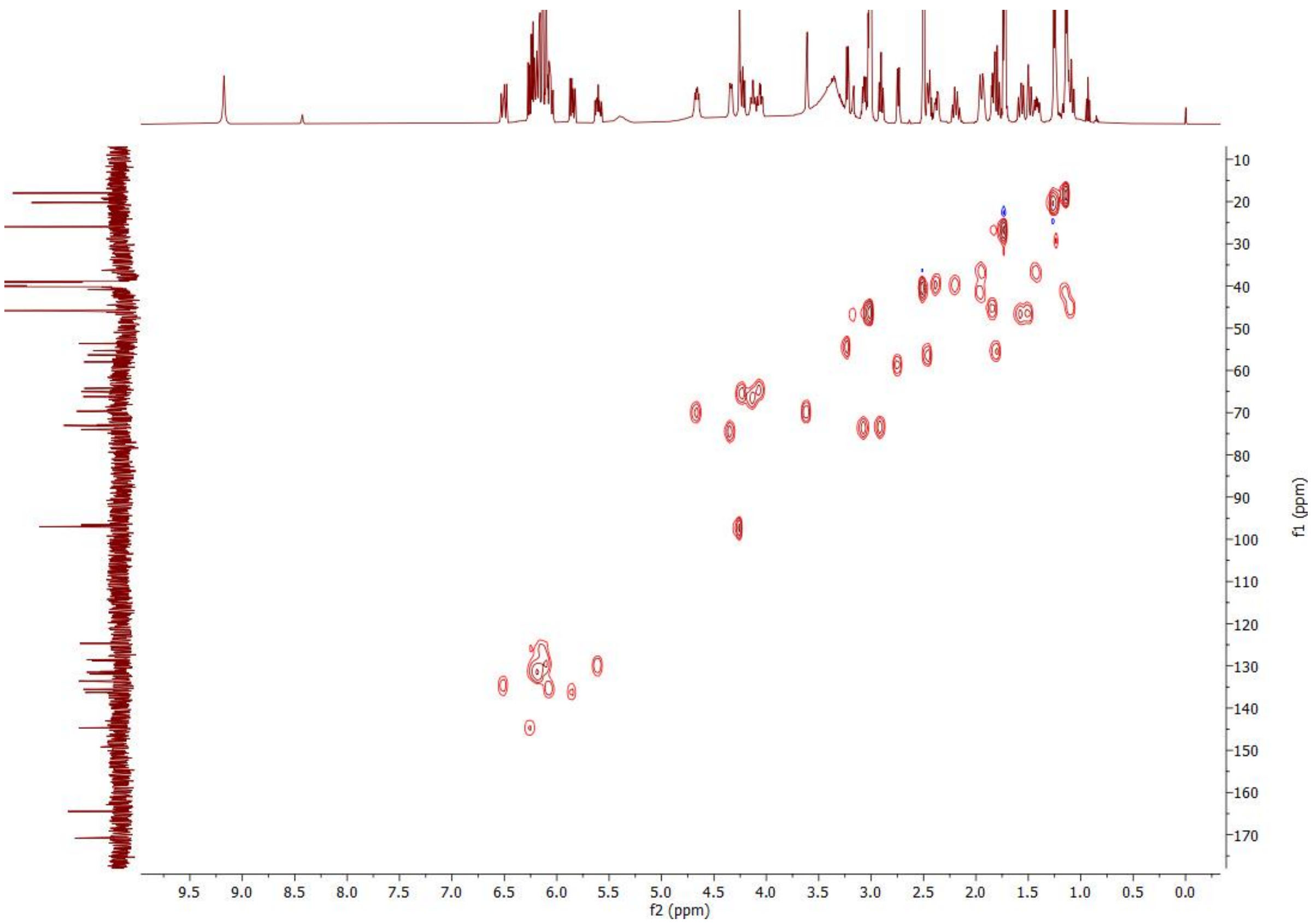

d

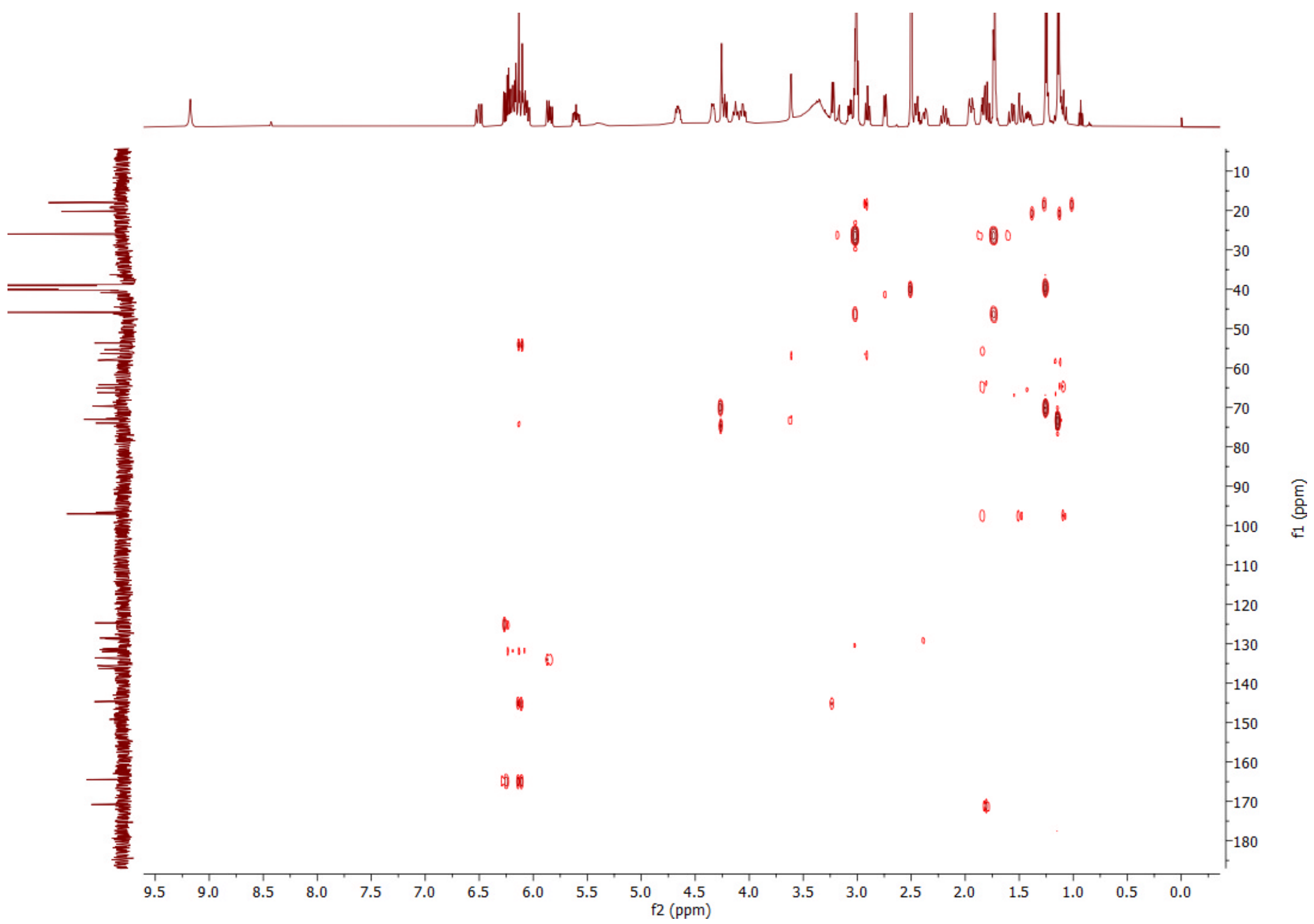

Supplementary Fig. 34. NMR data for Pim3 continued. (c) shows ^{1}H-^{13}C- HSQC NMR of Pim3 in DMSO-*d6*. **(d)** shows ^{1}H-^{13}C- HMBC NMR of Pim3 in DMSO-*d6*.

a

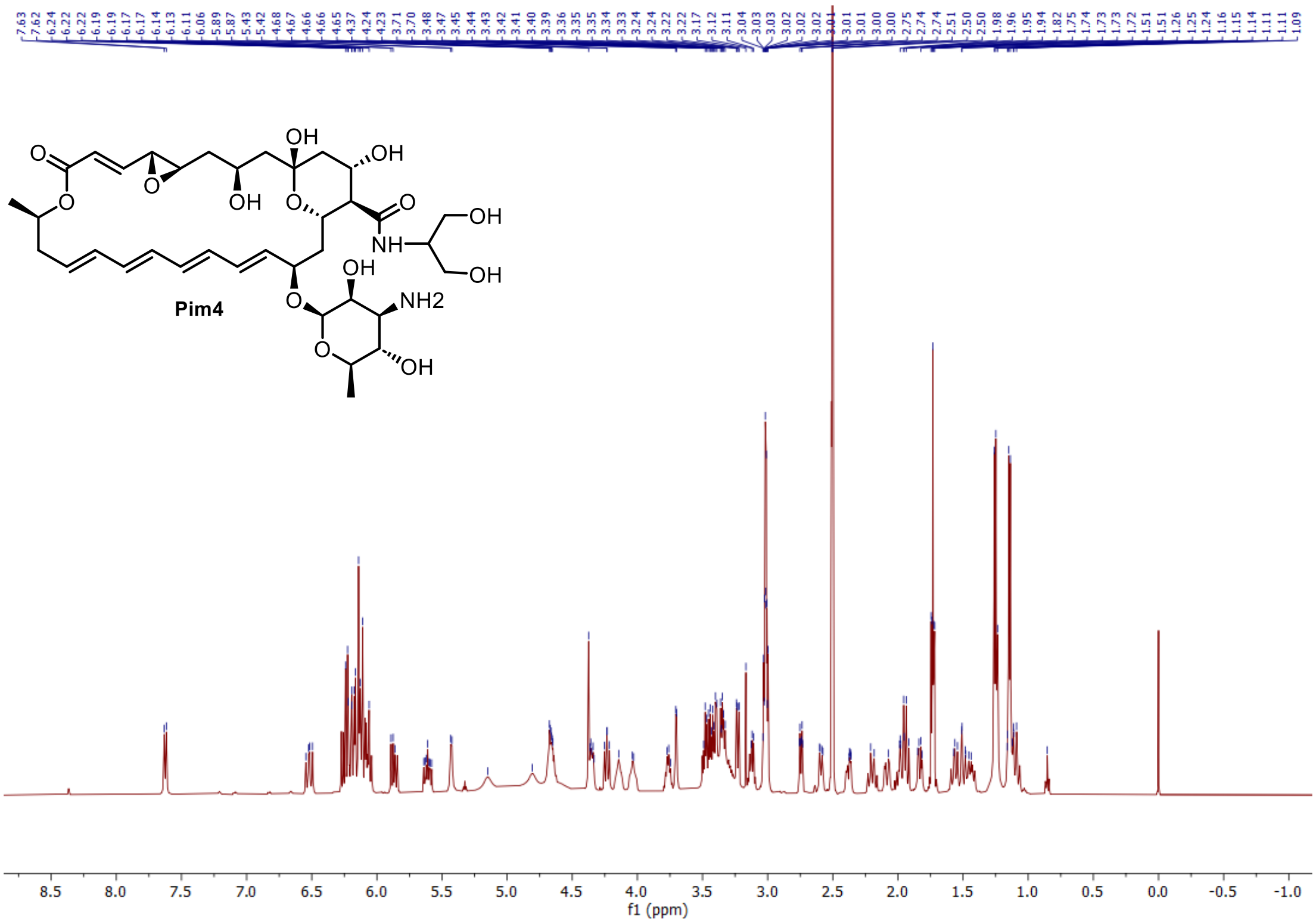

b

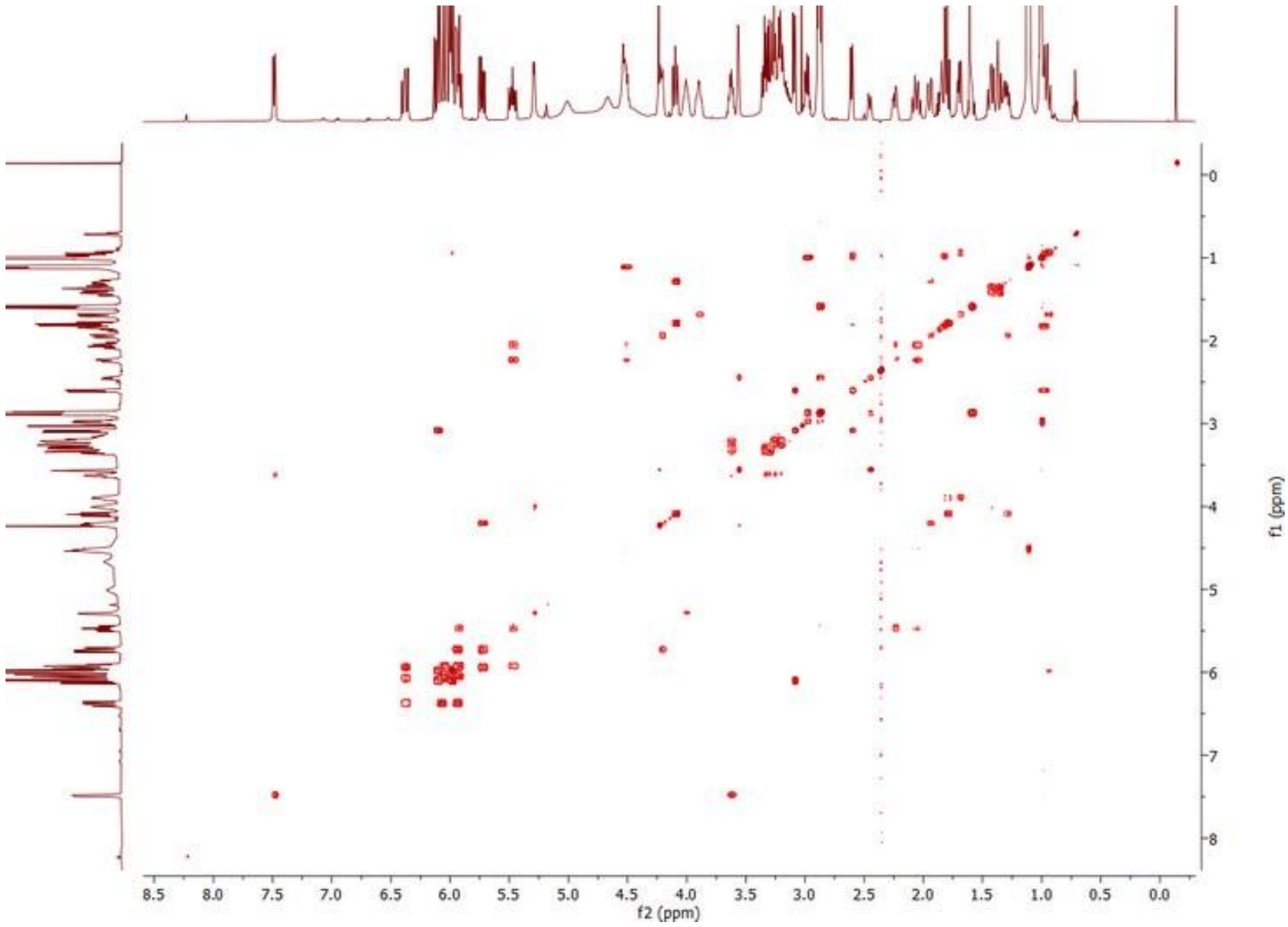

Supplementary Fig. 35. NMR data for Pim4. (a) shows ^{1}H-NMR of Pim4 in DMSO-*d6*. **(b)** shows ^{1}H-^{1}H COSY NMR of Pim4 in DMSO-*d6*.

c

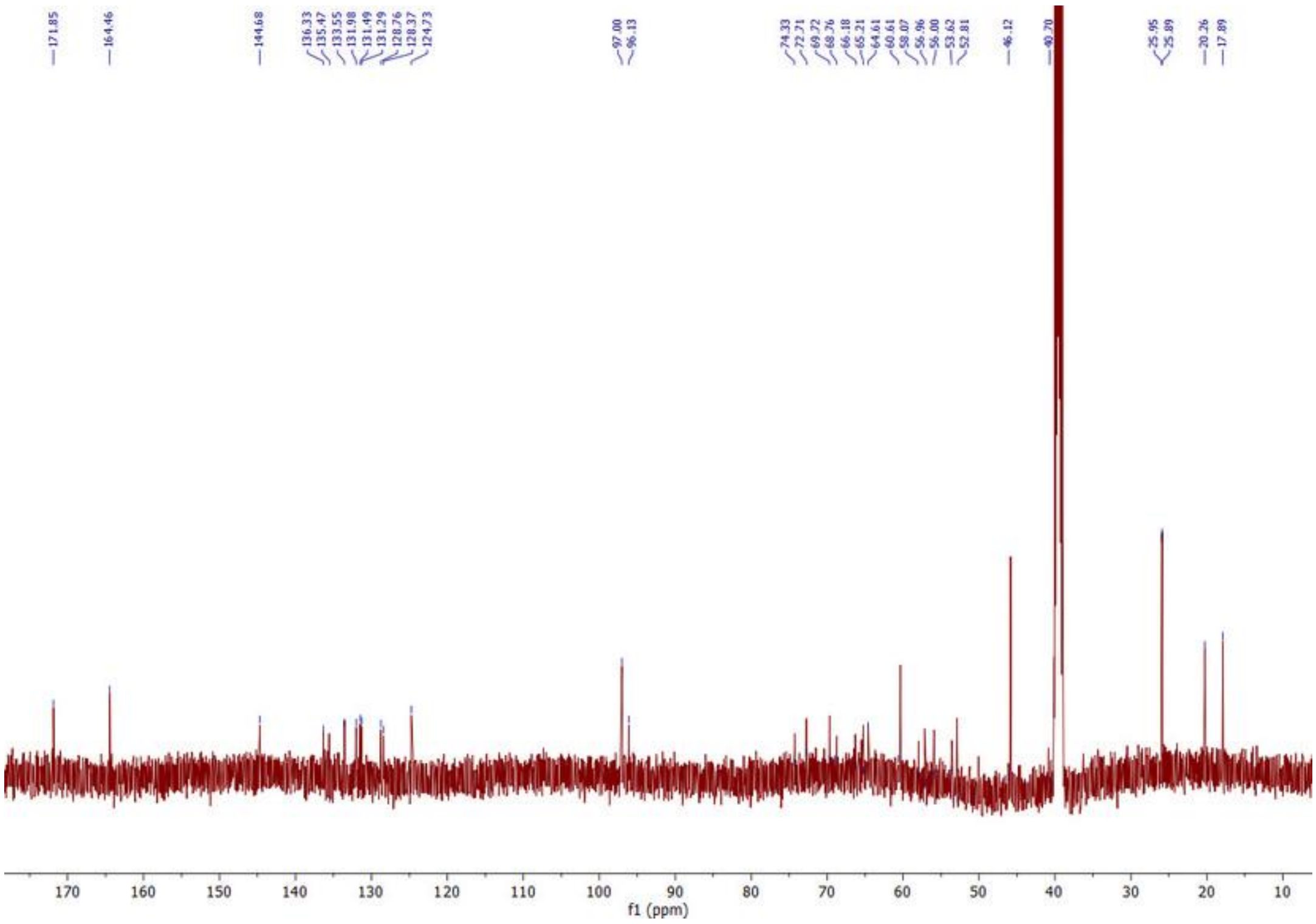

d

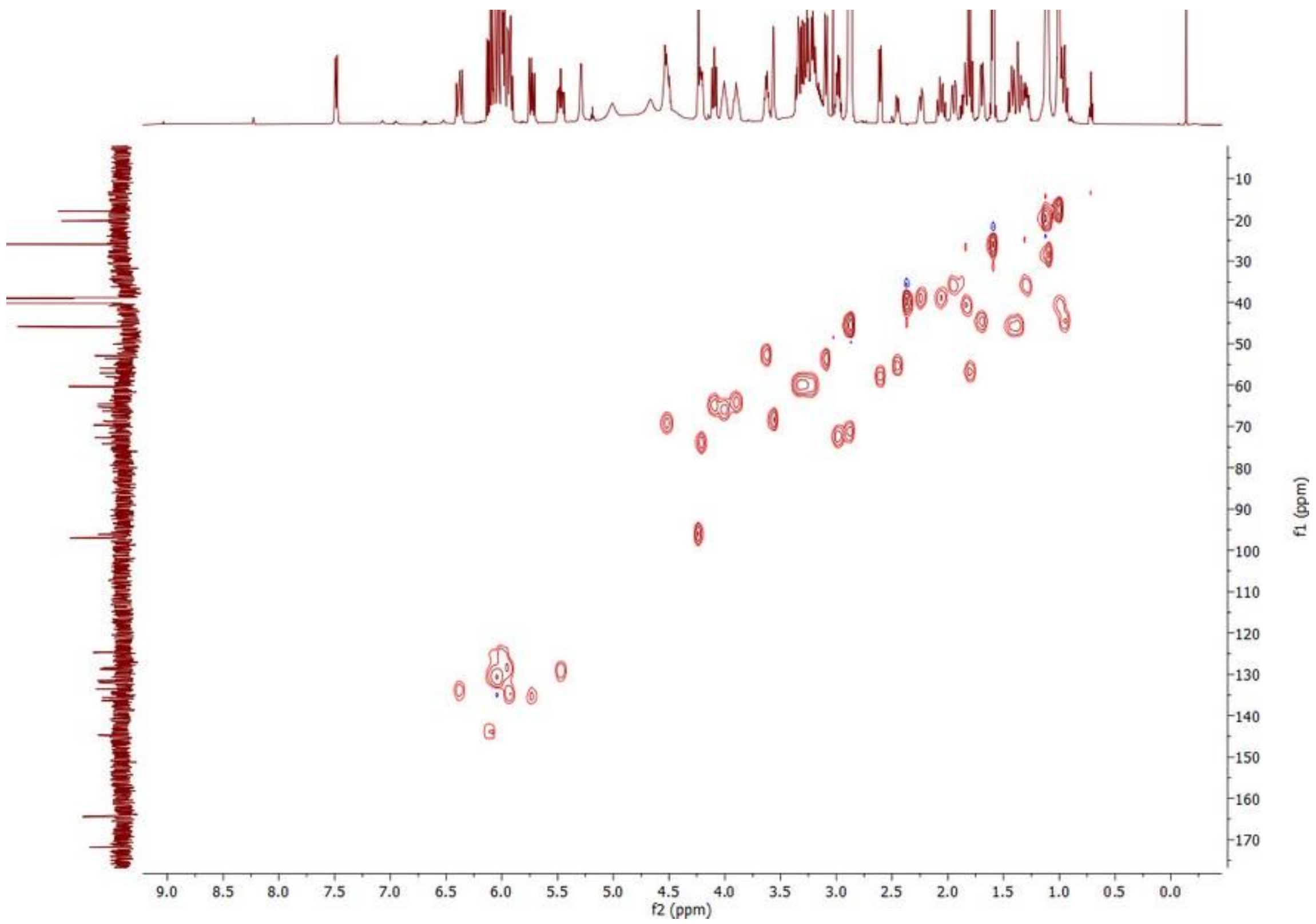

Supplementary Fig. 35. NMR data for Pim4 continued. (c) shows ^{13}C-NMR of Pim4 in DMSO-*d6*. **(d)** shows ^{1}H-^{13}C- HSQC NMR of Pim4 in DMSO-*d6*.

e

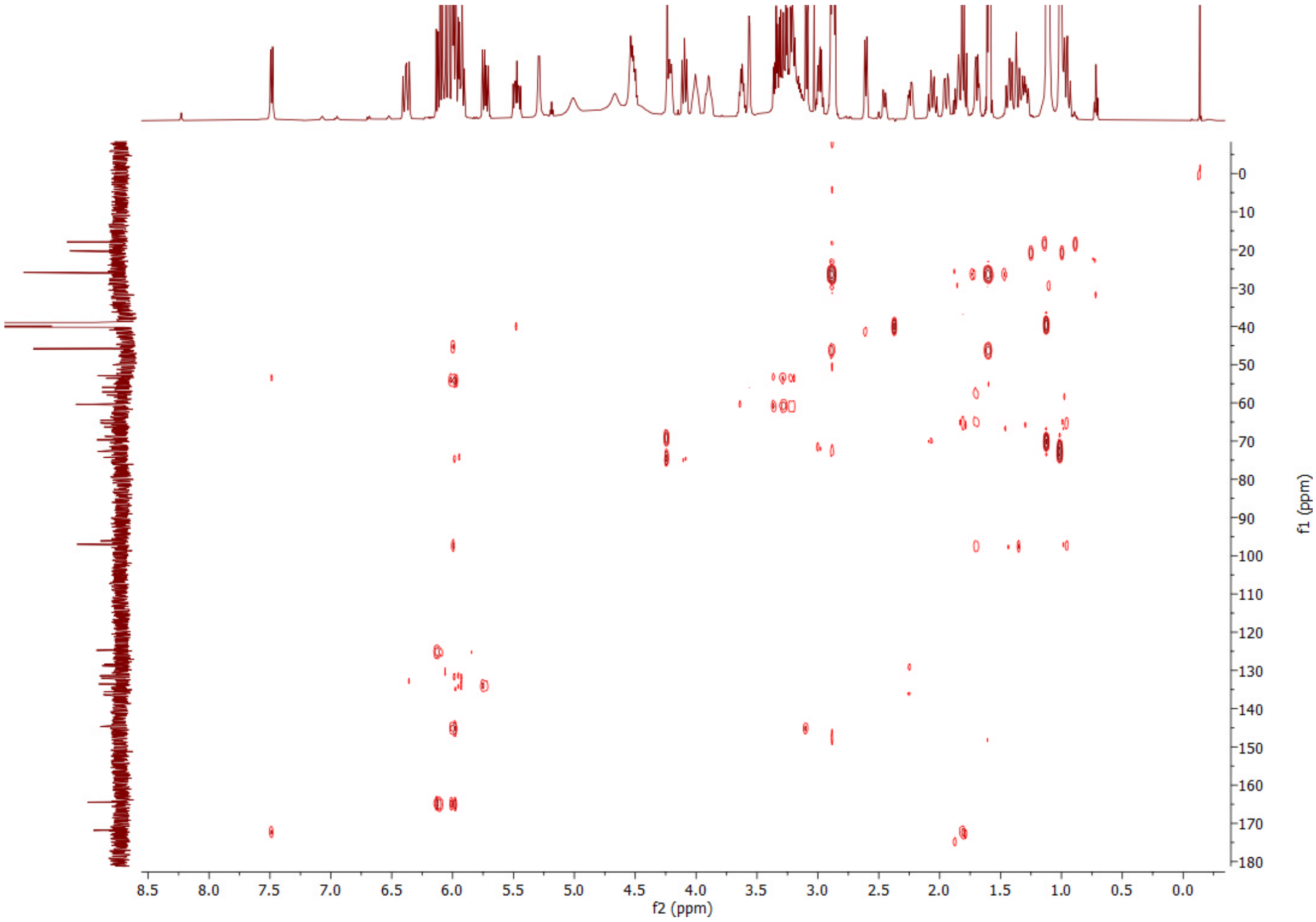

Supplementary Fig. 35. NMR data for Pim4 continued. (e) shows ^{1}H-^{13}C- HMBC NMR of Pim4 in DMSO-*d6*.

Position	^{1}H reported Pim in DMSO-*d6* [69]	^{1}H found Pim1 in MeOD	^{1}H found Pim2 in DMSO-*d6*	^{1}H found Pim3 in DMSO-*d6*	^{1}H found Pim4 in DMSO-*d6*	^{13}C found Pim3 in DMSO-*d6*	^{13}C found Pim4 in DMSO-*d6*
1						164.46	164.46
2		6.09	6.16	6.24	6.24	124.68	124.73
3	6.5229	6.43	6.25	6.53	6.37	133.55	133.55
4	3.2542	3.19	3.23	3.22	3.23	53.65	53.62
5	2.7423	2.86	2.73	2.73	2.74	58.00	58.07
6	1.9623	1.24, 2.06	1.94	1.96	1.94	40.85	39.74
7	4.1609	4.35	4.22	4.22	4.22	66.22	66.18
8	1.5496	1.62, 1.73	1.54	1.55	1.55	45.88	46.12
9	-	-		-	-	97.02	97.00
10	1.7909	1.30, 2.05	1.71	1.73	1.73	25.96	25.95
11	3.9928	4.28	4.12	4.11	4.15	65.04	65.21
12	1.7909	2.05	1.94	1.79	1.95	55.30	56.95
13	4.1609	4.47	4.05	4.06	4.04	64.26	64.61
14	1.4922	1.67, 2.19	1.47	1.42	1.44	25.89	25.89
15	4.3952	4.47	4.39	4.33	4.34	73.98	74.33
16	5.9283	6.01	5.83	5.84	5.87	136.30	136.33
17-20	6.1439	6.20	6.02-6.27	6.10-6.24	6.03-6.26	124.68-135.50	124.73-135.47
21	6.1439	6.50	6.14	6.14	6.14	135.60	135.47
22	6.1439	6.16	6.07	6.07	6.03	136.30	
23	5.6116	5.64	5.59	5.59	5.61	128.80	128.76
24	2.2077	2.27, 2.43	2.20	2.19	2.20	39.63	39.46
25	4.6517	4.75	4.64	4.66	4.66	69.97	69.72
25-CH_3	1.1750	1.33	1.16	1.12	1.13	17.99	17.89
NH_2		8.56	8.32	8.43	8.36		
-CONH-			7.82	9.18	7.36		
-OH			10.56				
1′	4.5043	4.53	4.34	4.26	4.36	96.59	97.00
2′	3.7546	3.96	3.87	3.60	3.70	69.52	68.76
3′	2.8291	3.06	2.37	2.44	2.58	56.28	58.07
4′	3.1765	3.39	2.99	2.90	3.03	73.03	72.71
5′	3.2355	3.27	3.15	3.06	3.11	72.85	74.33
6′	1.2593	1.30	1.24	1.25	1.24	20.25	20.26
-CH_2 of Serinol					3.45		60.61
-CH of Serinol					3.76		53.62

Supplementary Fig. 36. NMR data of Pim and its C16 derivatives

a

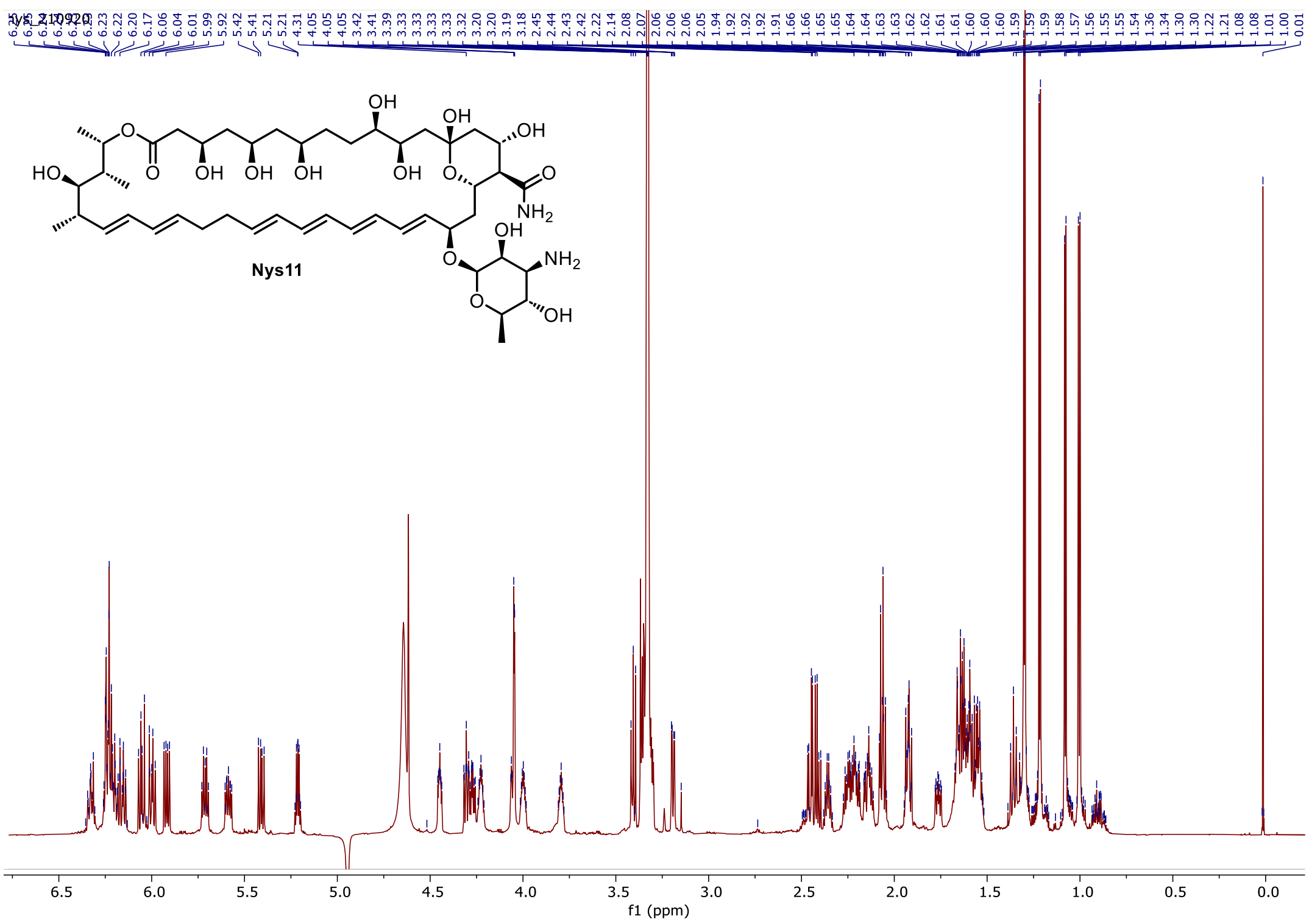

b

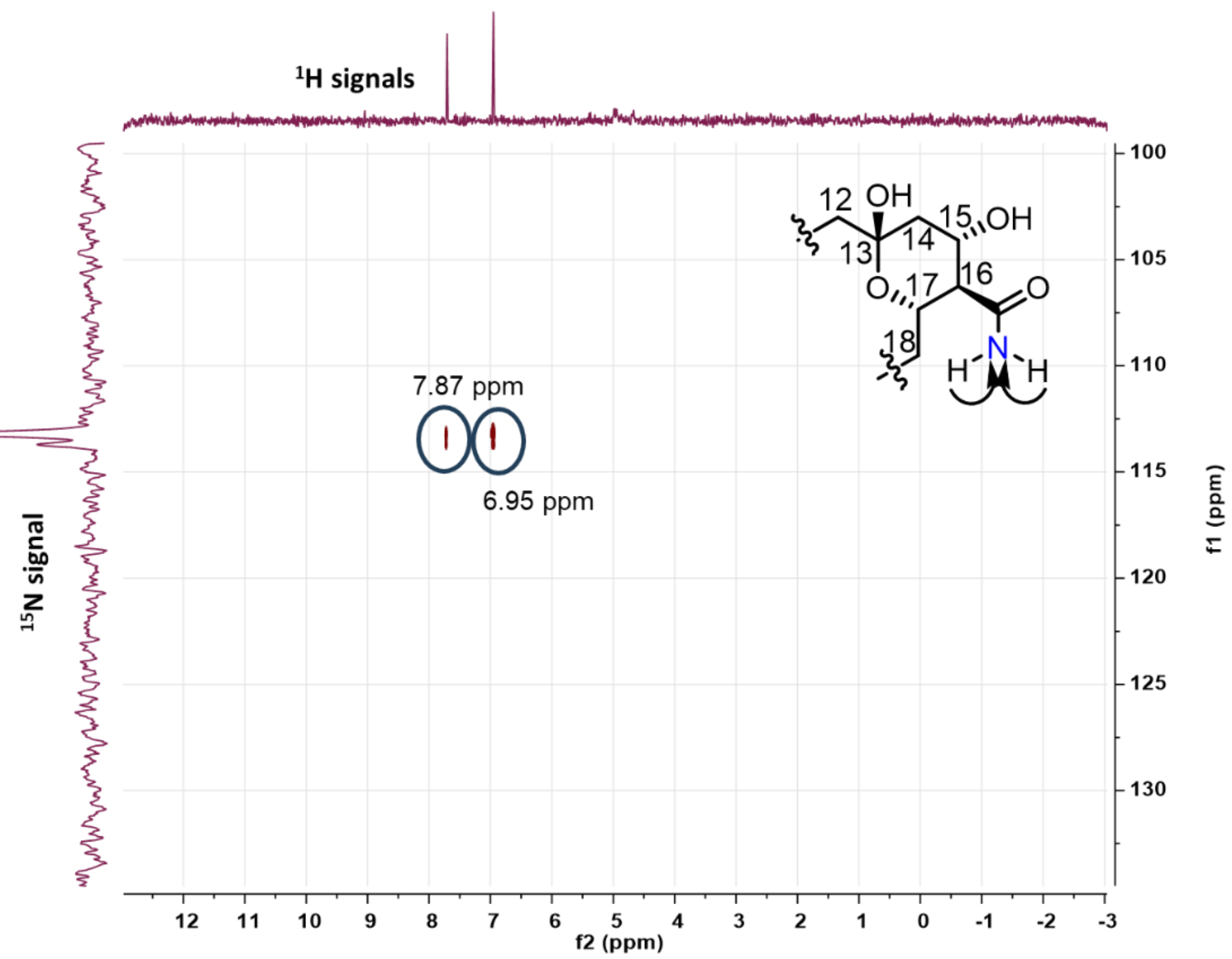

Supplementary Fig. 37. NMR data for Nys11. (a) shows ^{1}H-NMR of Nys11 in MeOD. **(b)** shows ^{1}H-^{15}N HSQC data for $^{15}NH_2$ labelled Nys11 in (10% D_2O, 90% MeOD). ^{15}N shift observed at 113.2 ppm and the corresponding ^{15}N protons at 6.95 and 7.87 ppm confirming $^{15}NH_2$ incorporation in NysA1.

c

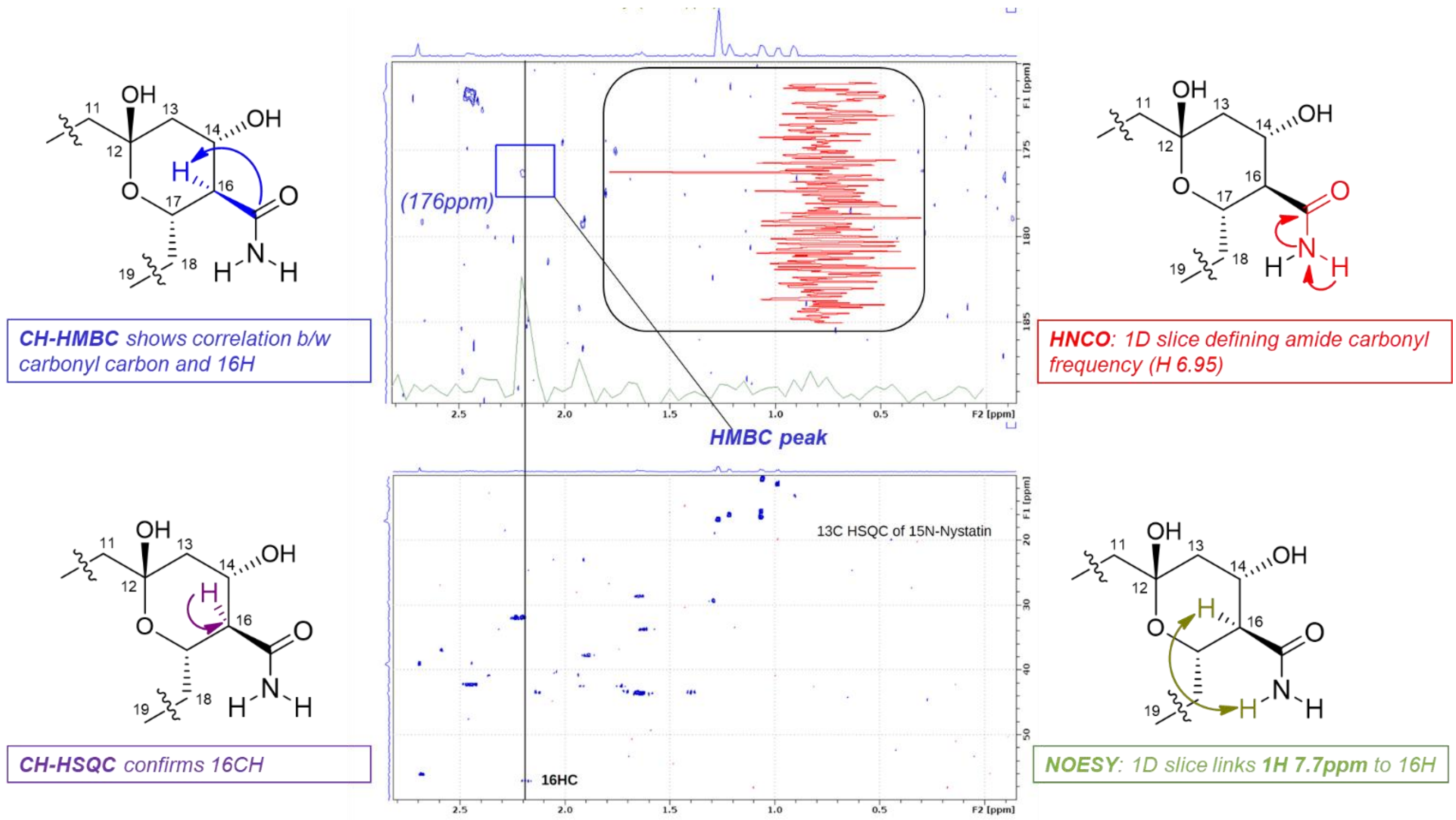

Supplementary Fig. 37. NMR data for Nys11 continued. (c) shows overlay of Nys11 spectra for HNCO, NOESY, CH-HMBC and CH-HSQC NMR experiments.

a

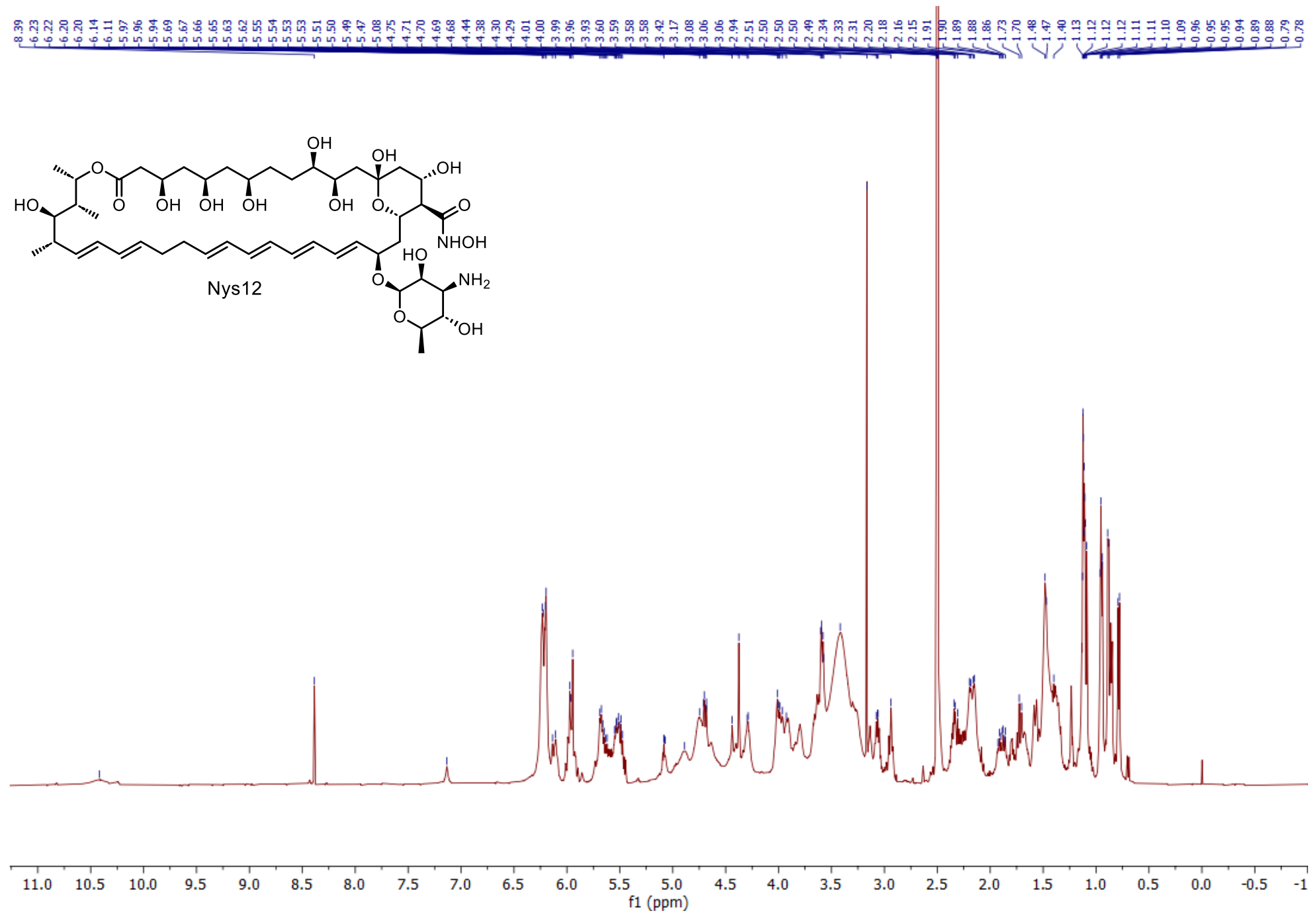

b

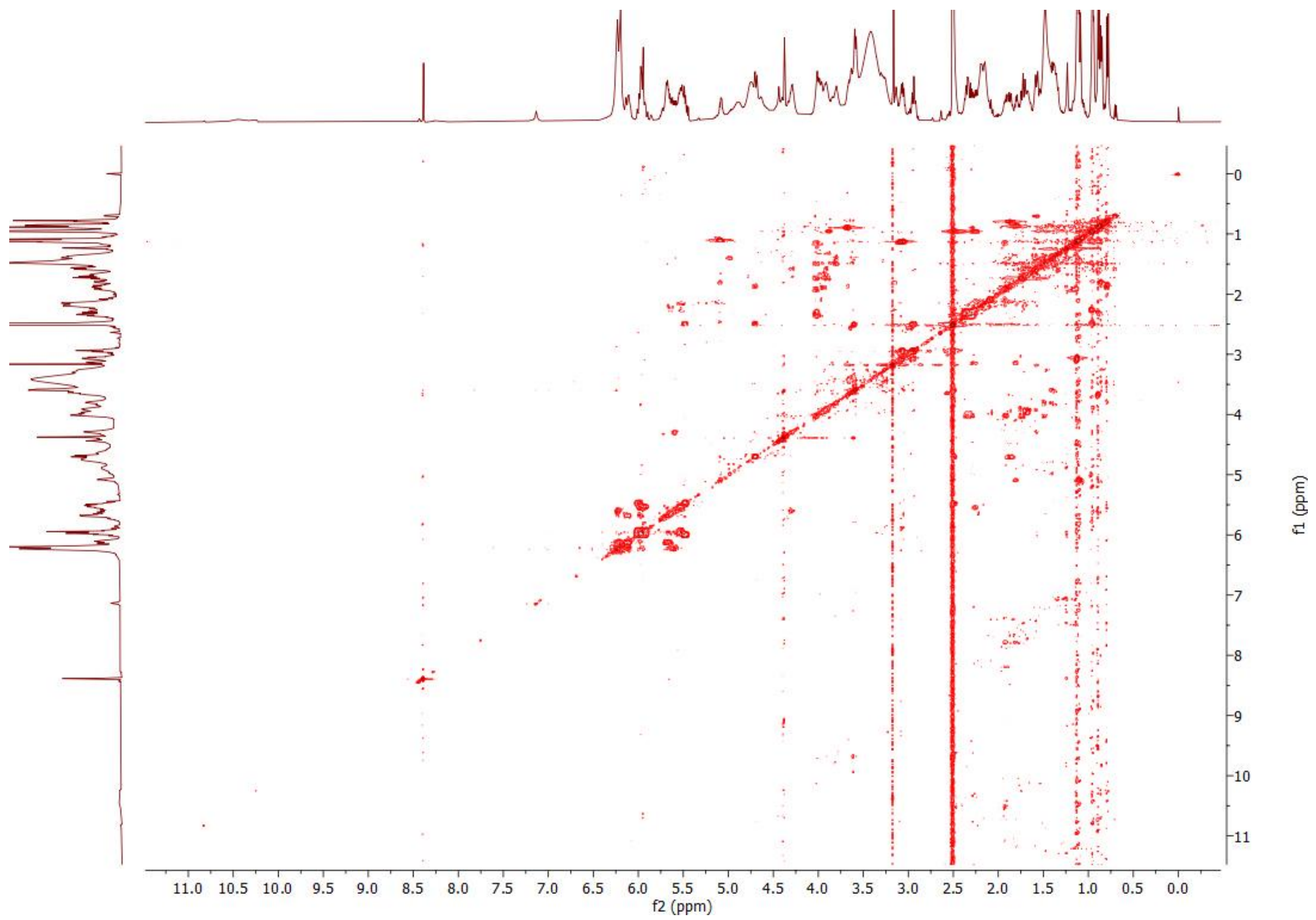

Supplementary Fig. 38. NMR data for Nys12. (a) shows ^{1}H NMR of Nys12 in DMSO-*d6*. **(b)** shows ^{1}H-^{1}H COSY NMR of Nys12 in DMSO-*d6*.

a

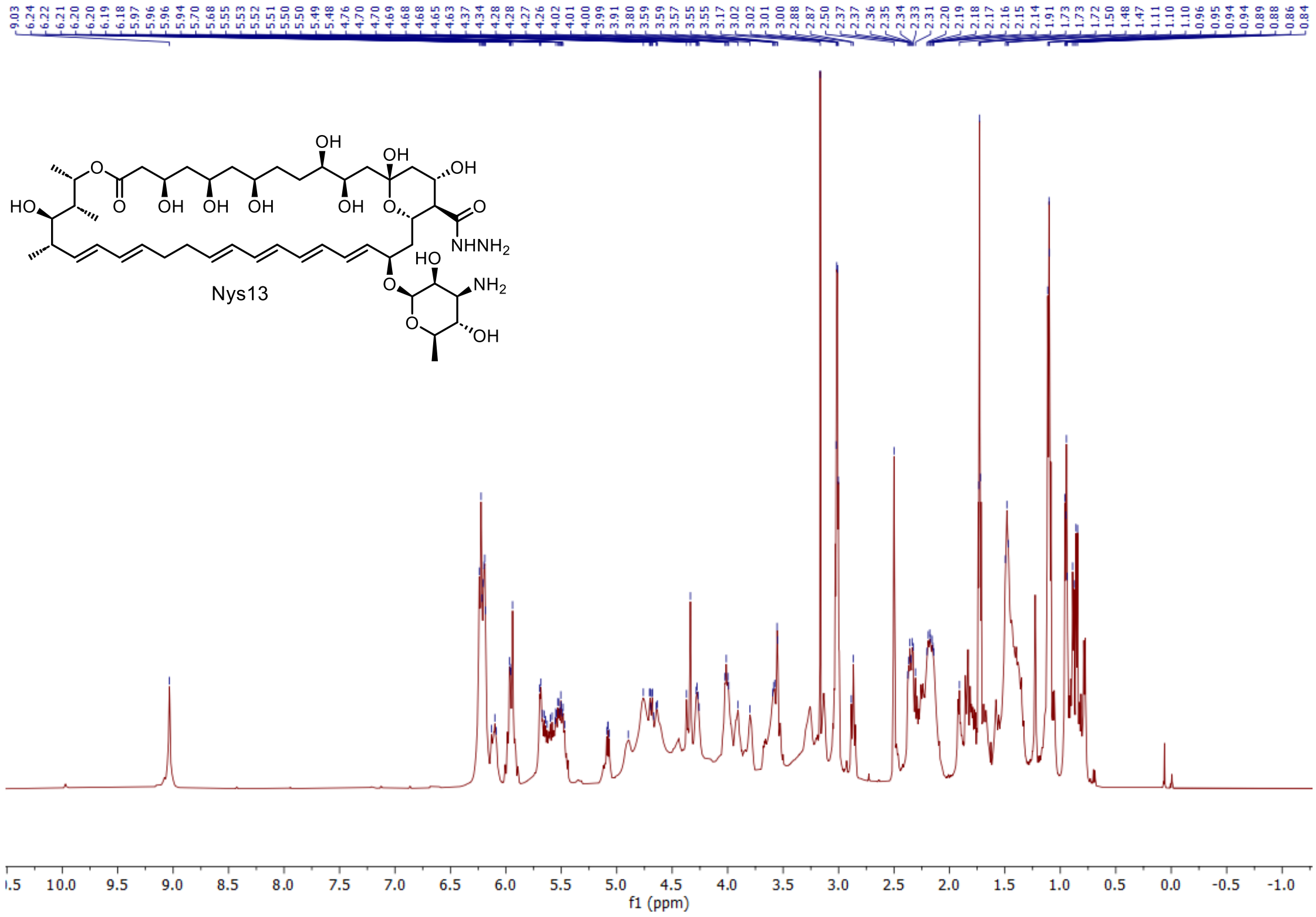

b

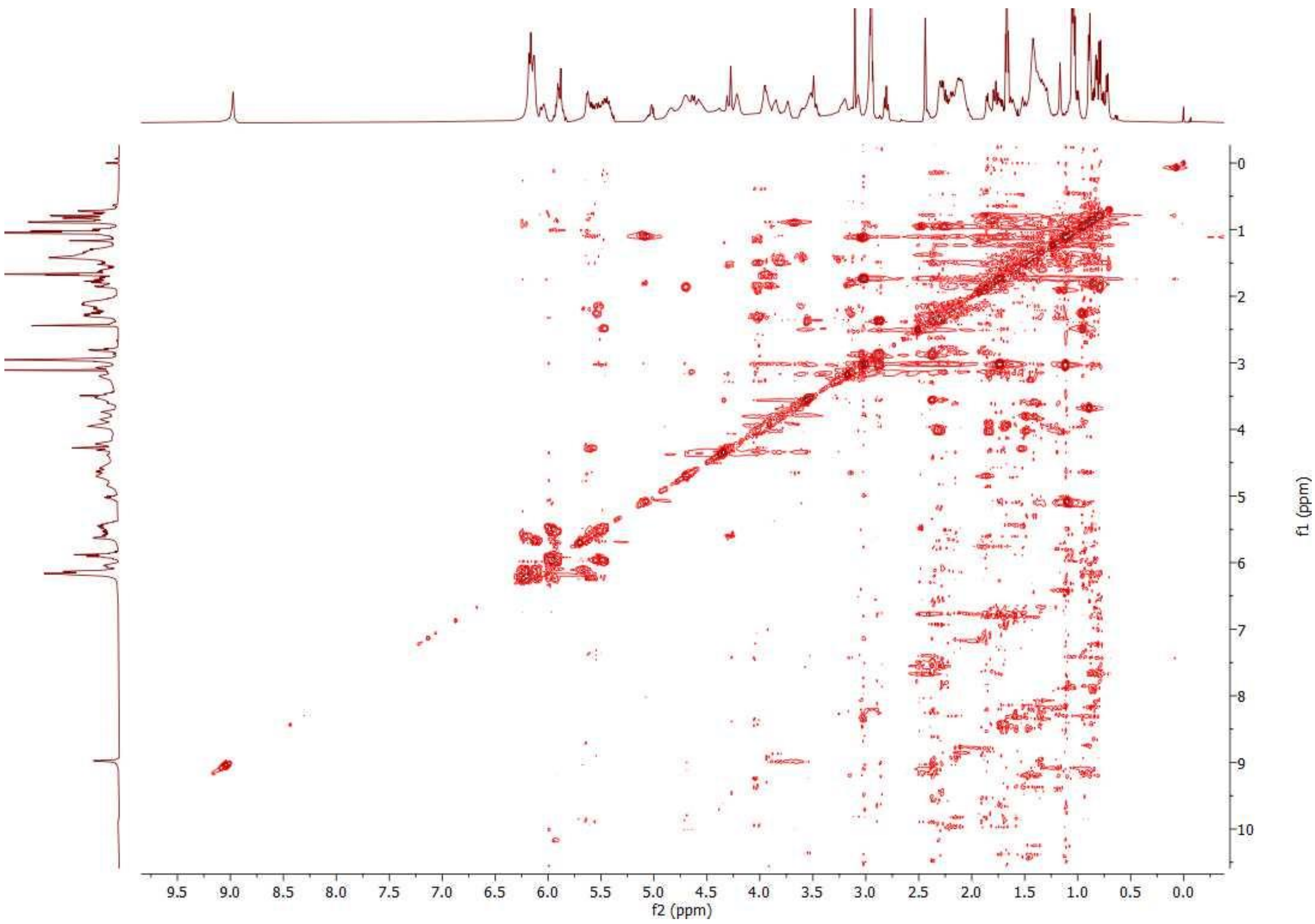

Supplementary Fig. 39. NMR data for Nys13. (a) shows ^{1}H NMR of Nys13 in DMSO-*d6*. **(b)** shows ^{1}H-^{1}H COSY NMR of Nys13 in DMSO-*d6*.

c

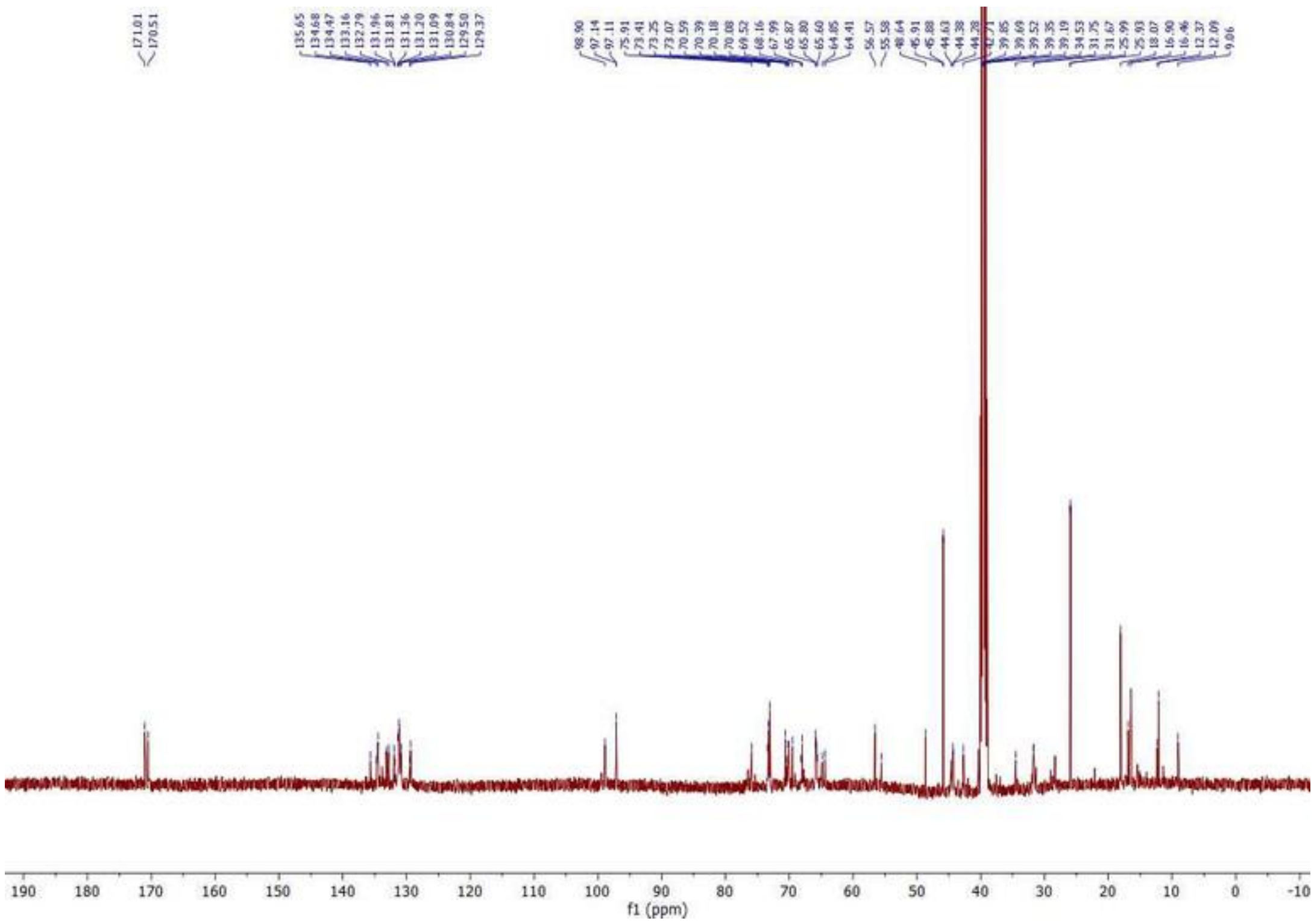

Supplementary Fig. 39. NMR data for Nys13 continued. (c) shows ^{13}C NMR of Nys13 in DMSO-*d6*.

a

b

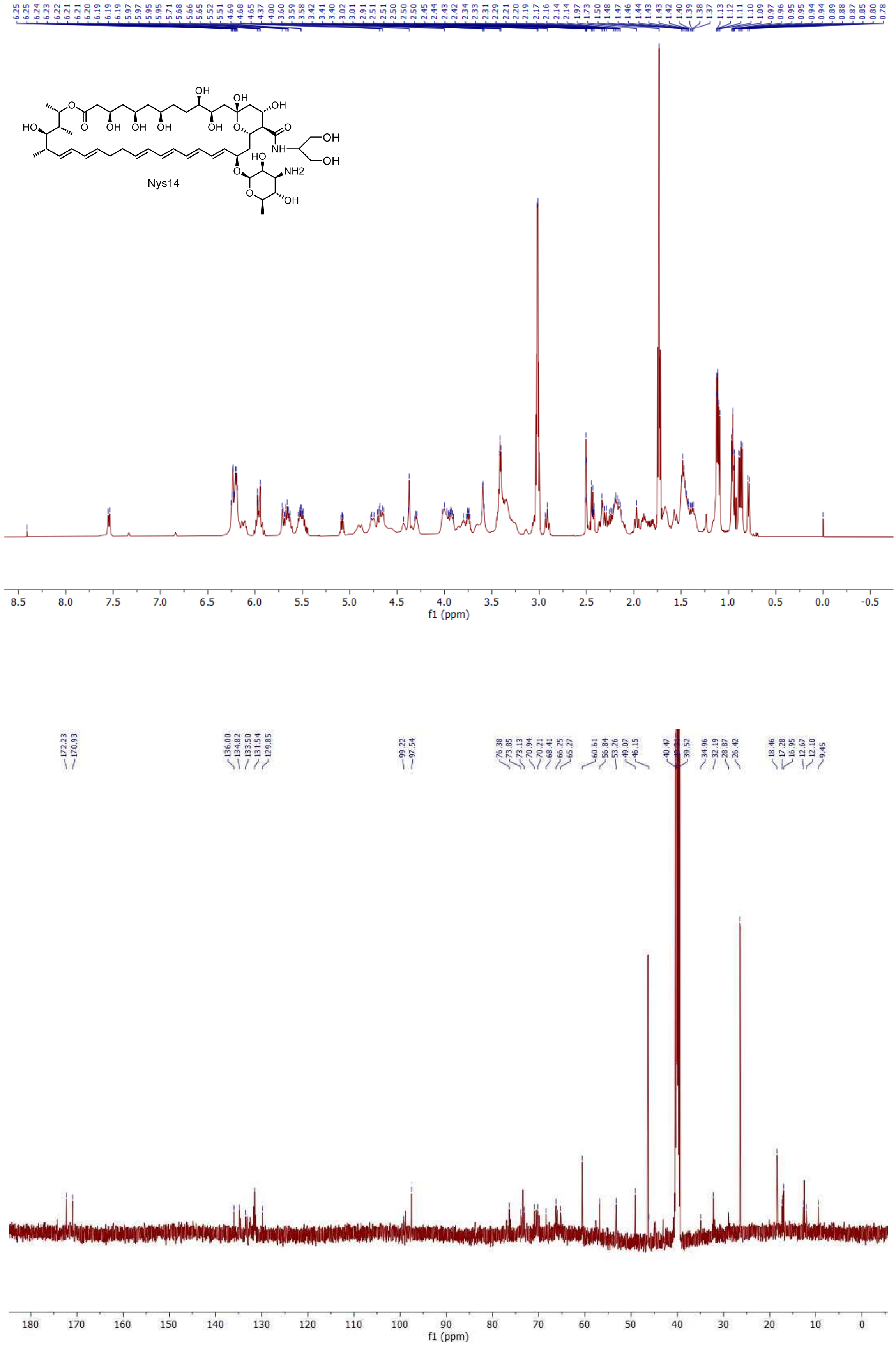

Supplementary Fig. 40. NMR data for Nys14. (a) shows ^{1}H NMR of Nys14 in DMSO-*d6*. **(b)** shows ^{13}C NMR of Nys14 in DMSO-*d6*.

c

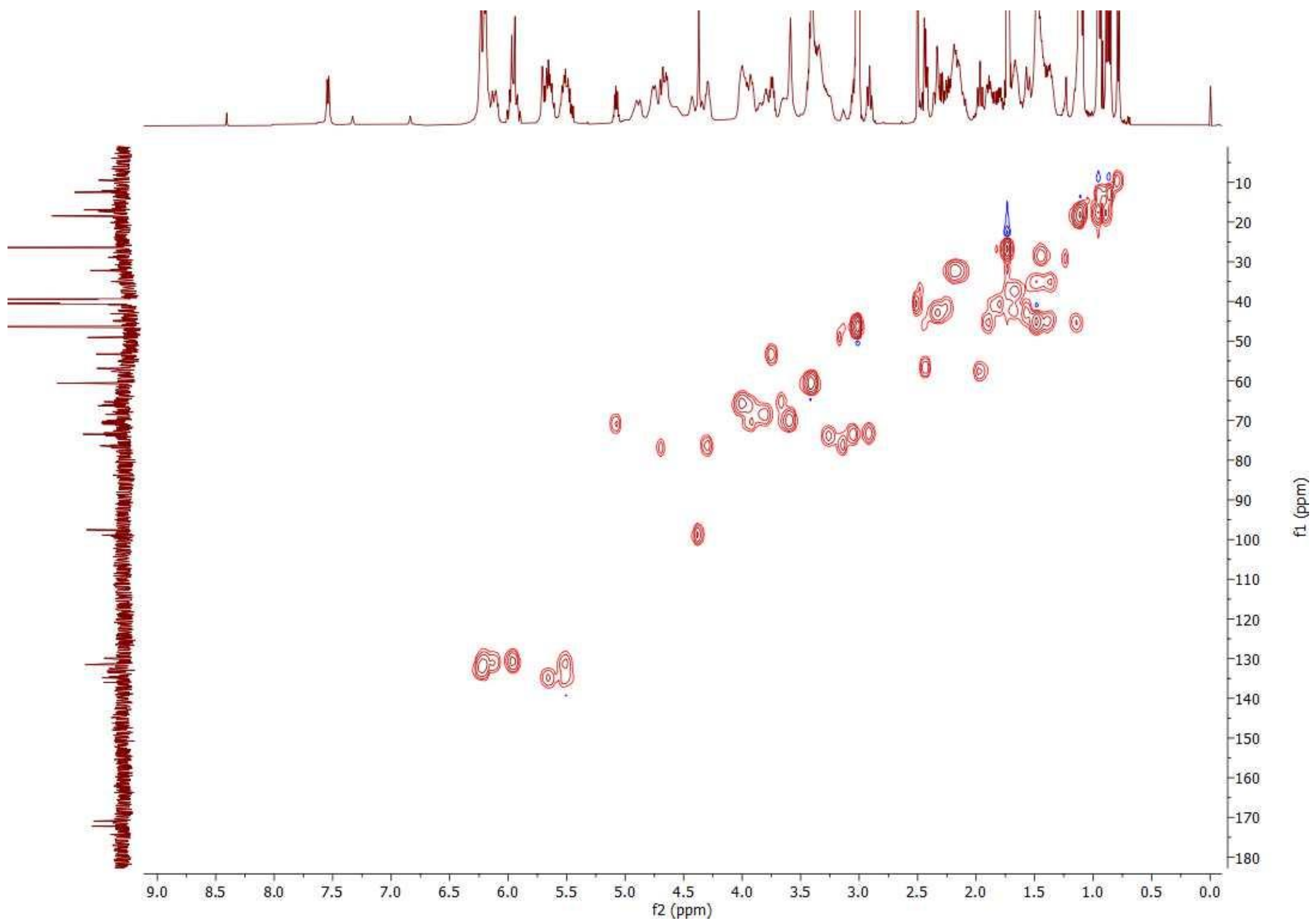

Supplementary Fig. 40. NMR data for Nys14 continued. (c) shows ^{1}H-^{13}C HSQC NMR of Nys14 in DMSO-*d6*.

Position	^{1}H reported NysA1 in MeOD	^{1}H found Nys11 in MeOD	^{1}H found Nys12 in DMSO-d6	^{1}H found Nys13 in DMSO-d6	^{1}H found Nys14 in DMSO-d6	^{13}C reported NysA1 in MeOD	^{13}C found Nys13 in DMSO-d6	^{13}C found Nys14 in DMSO-d6
1								
2	2.36	2.39, 2.44	2.33	2.35	2.34	43.1	43.3	44.58
3	4.17	4.21	3.98	4.03	3.99	67.9	66.12	66.25
4	1.56	1.61	1.47	1.51	1.48	44.1	44.49	44.58
5	3.95	3.99	3.94	3.99	3.92	70.4	70.5	70.25
6	1.51	1.58	1.42	1.57	1.47	44.6	45.1	44.75
7	3.74	3.78	3.78	3.65	3.74	71.5	71.04	70.95
8	1.51	1.52, 1.64	1.48	1.54	1.47	29.9	29.95	28.84
9	1.51	1.53, 1.58	1.48	1.51	1.47	29.9	28.95	28.84
10	3.29	3.34	3.27	3.26	3.28	70.0	73.51	73.29
11	4.03	4.04	3.95	4.00	3.98	71.4	70.82	70.57
12	1.58,1.86	1.64,1.91	1.49, 1.70	1.46, 1.72	1.47, 1.73	43.7	43.70	43.18
13							97.29	97.53
14	1.28,2.02	1.34, 2.05	1.35, 1.90	1.36, 1.92	1.34, 1.95	44.6	44.94	44.92
15	4.25	4.26	4.02	4.03	4.02	67.5	66.34	66.10
16	2.05	2.04	2.15	2.15	1.96	60.9	56.94	56.75
17	4.26	4.29	3.98	3.98	3.97	67.5	66.49	66.25
18	1.69,2.10	1.75, 2.14	1.72, 2.22	1.73, 2.25	1.72,2.27	39.1	39.94	40.15
19	4.39	4.43	4.30	4.27	4.29	78.6	76.19	76.12
20	5.86	5.9	5.70	5.69	5.70	135.5	134.66	134.90
21-26	6.10-6.29	6.13-6.31	6.10- 6.25	6.08-6.26	6.09-6.24	132.2	132.36	131.890
27	5.65	5.70	5.65	5.68	5.64	135.5	134.66	136.11
28	2.13,2.21	2.18, 2.24	2.18	2.18	2.18	33.2	34.65	32.14
29	2.07,2.17	2.11, 2.22	2.14	2.14	2.16	33.2	34.65	32.14
30	5.52	5.57	5.53	5.58	5.47	132.9	132.39	131.89
31	5.94	5.98	5.94	5.94	5.93	132.2	132.2	132.31
32	6.00	6.04	5.98	5.96	5.97	132.2	132.3	132.31
33	5.35	5.39	5.51	5.52	5.54	135.5	136.0	134.94
34	2.30	2.34	2.33	2.33	2.34	42.1		43.04
34-CH_3	1.02	1.07	0.97	0.95	0.95	17.7	17.22	18.27
35	3.23	3.29	3.31	3.25	3.14	78.6	76.98	76.34
36	1.87	1.91	1.90	1.92	1.81	41.4		41.42
36-CH_3	0.95		0.88	0.85	0.86	12.1	12.33	12.55
37	5.16	5.20	5.08	5.08	5.08	72.0		70.06
37-CH_3	1.17	1.20	1.08	1.11	1.09	17.2	16.76	16.84
NH_2			8.38	8.42	8.40			
-CONH-			7.12	9.03	7.55			
-OH			10.40					
1′	4.56	4.61	4.36	4.34	4.36	98.9	97.29	97.48
2′	3.98	4.03	3.60	3.53	3.59	68.5	69.62	69.90
3′	3.14	3.18	2.33	2.36	2.43	56.8	55.98	56.54
4′	3.30	3.39	2.93	2.87	2.90	75.1	73.67	73.63
5′	3.28	3.34	3.05	3.00	3.00	74.0	73.43	73.36
6′	1.24	1.28	1.13	1.10	1.11	17.3	17.27	17.23
-CH_2 of Serinol					3.41			60.70
-CH of Serinol					3.73			53.21

Supplementary Fig. 41. NMR data of NysA1 and its C16 derivatives

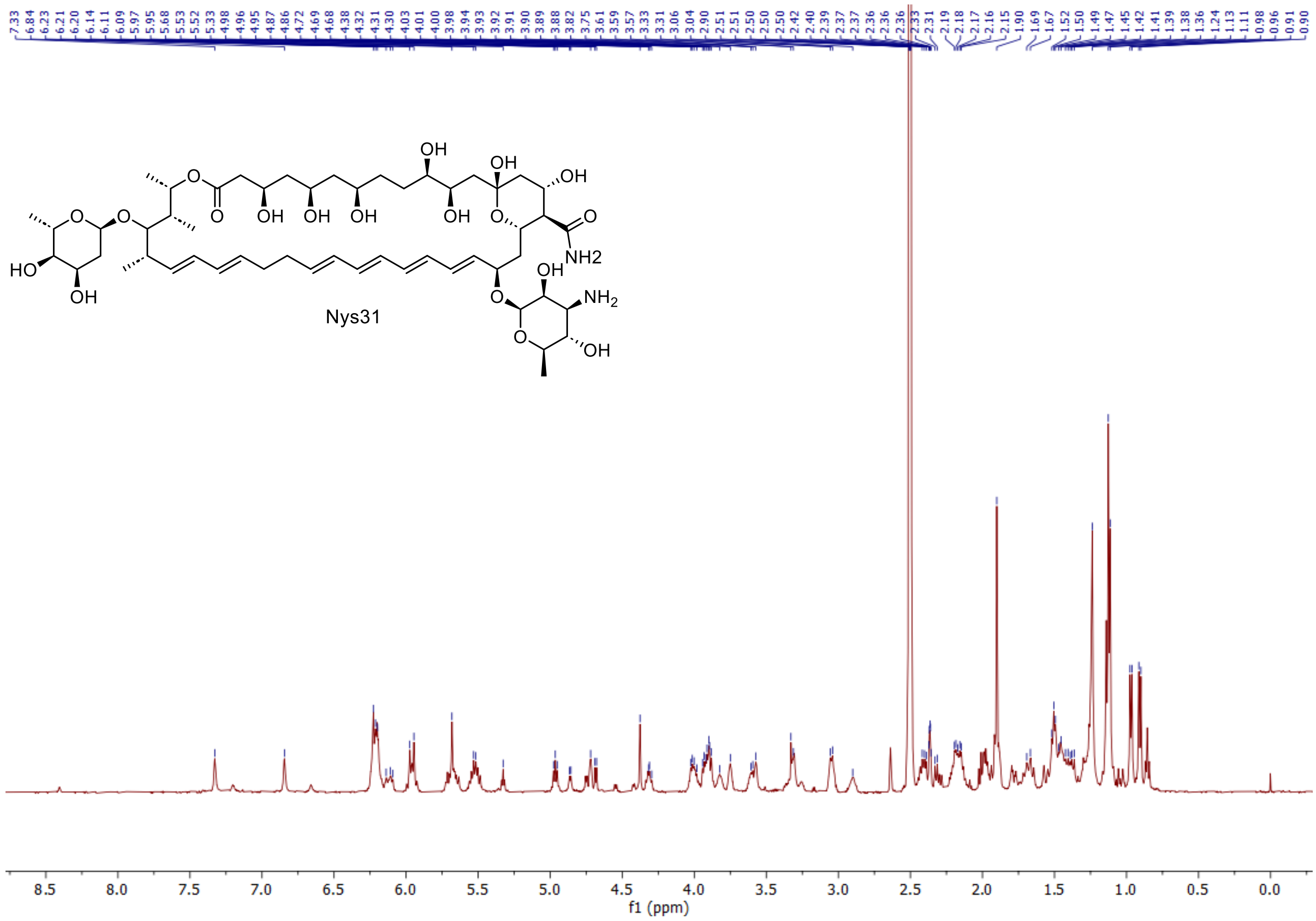

Supplementary Fig. 42. ^{1}H-NMR of Nys31 in DMSO-*d6*

a

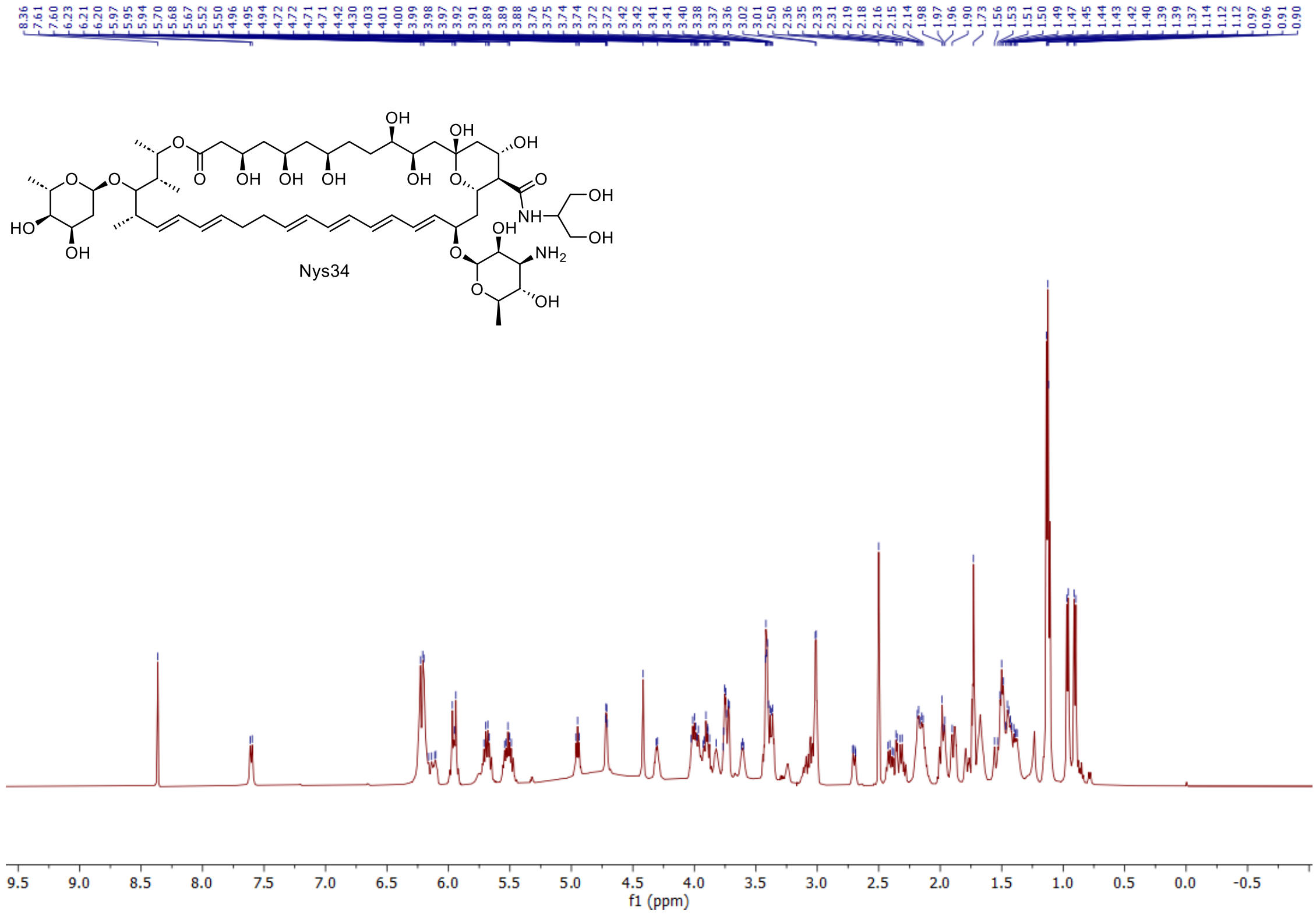

b

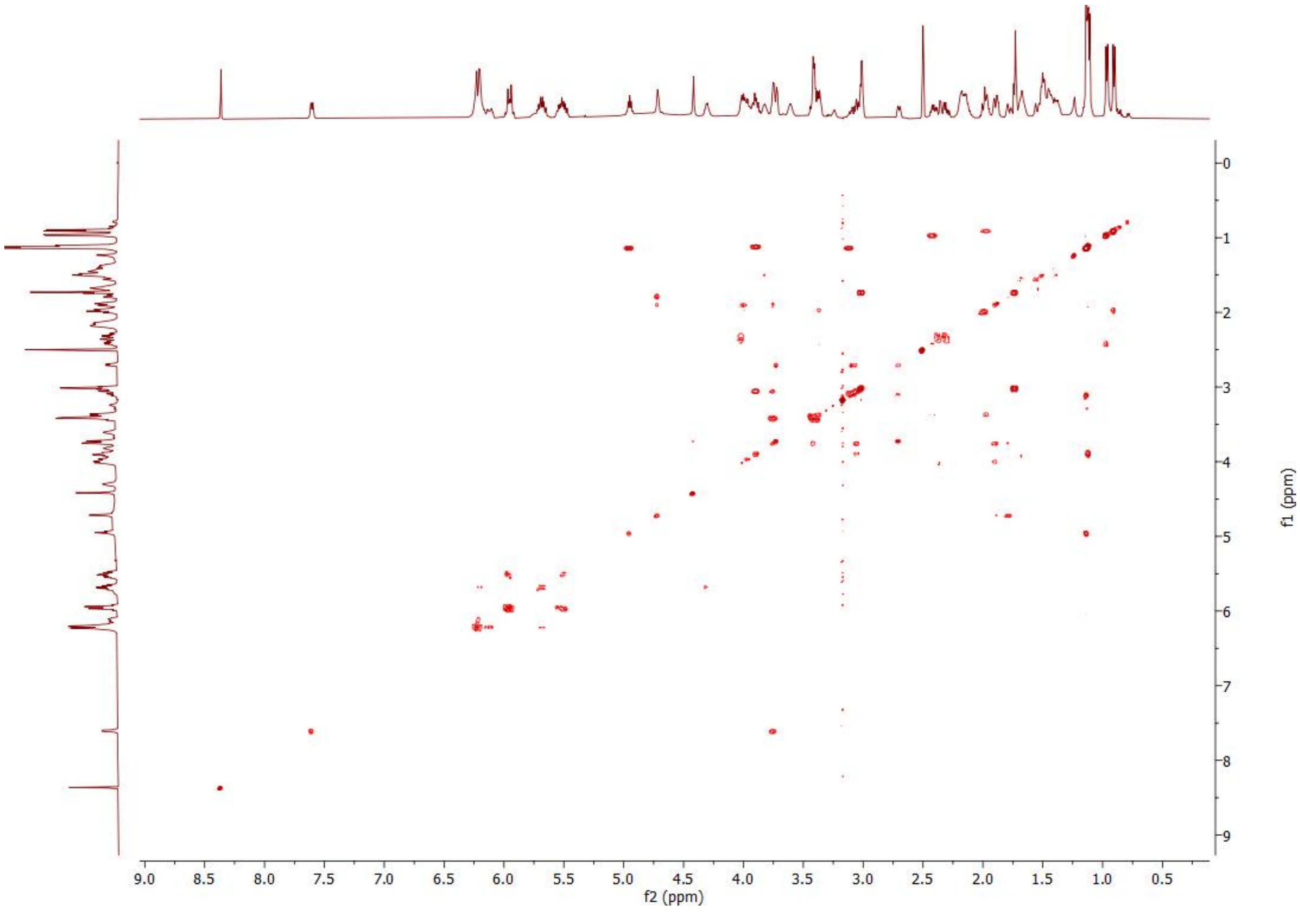

Supplementary Fig. 43. NMR data for Nys34. (a) ^{1}H-NMR of Nys34 in DMSO-*d6*. **(b)** shows ^{1}H-^{1}H COSY NMR of Nys34 in DMSO-*d6*.

c

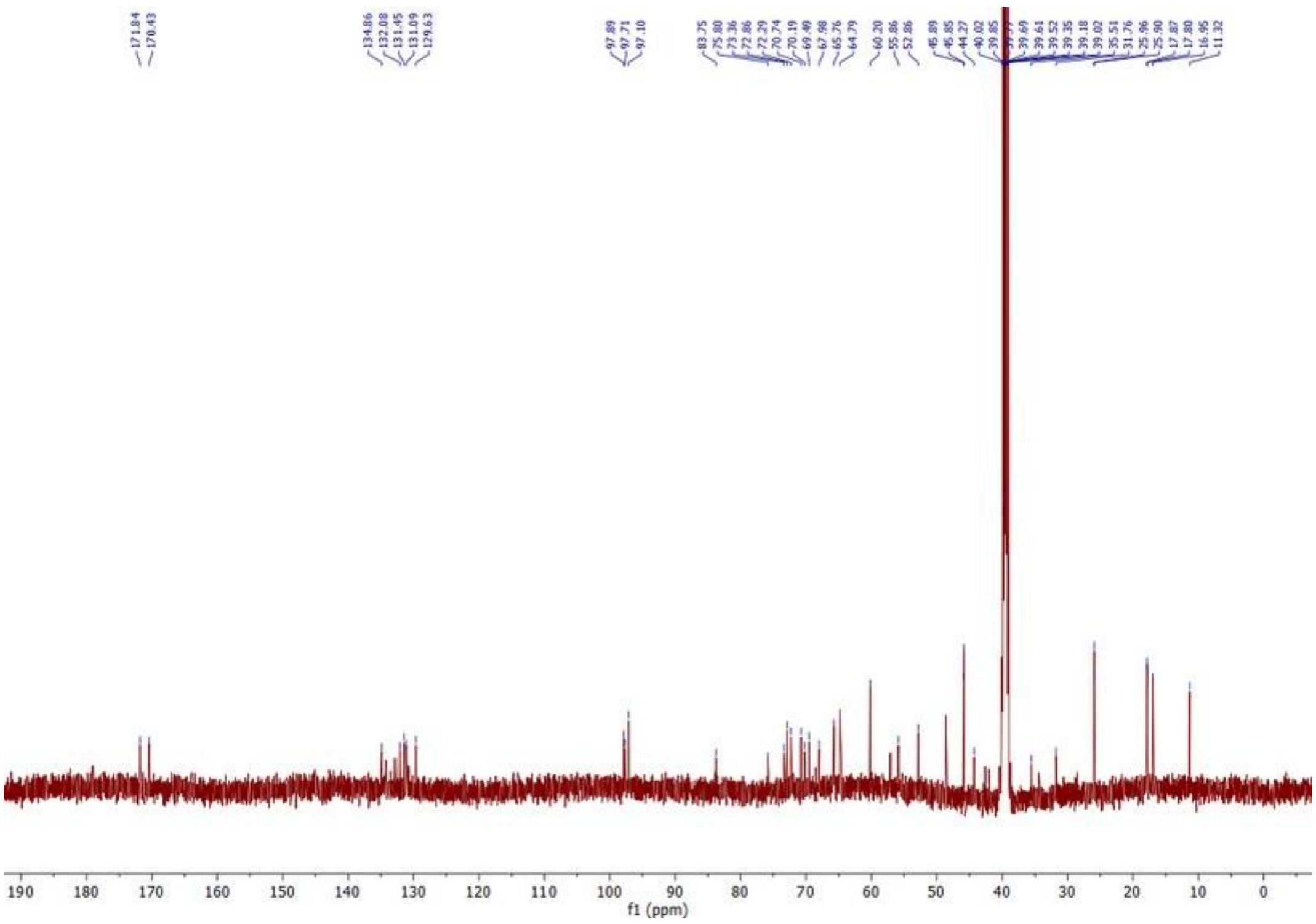

d

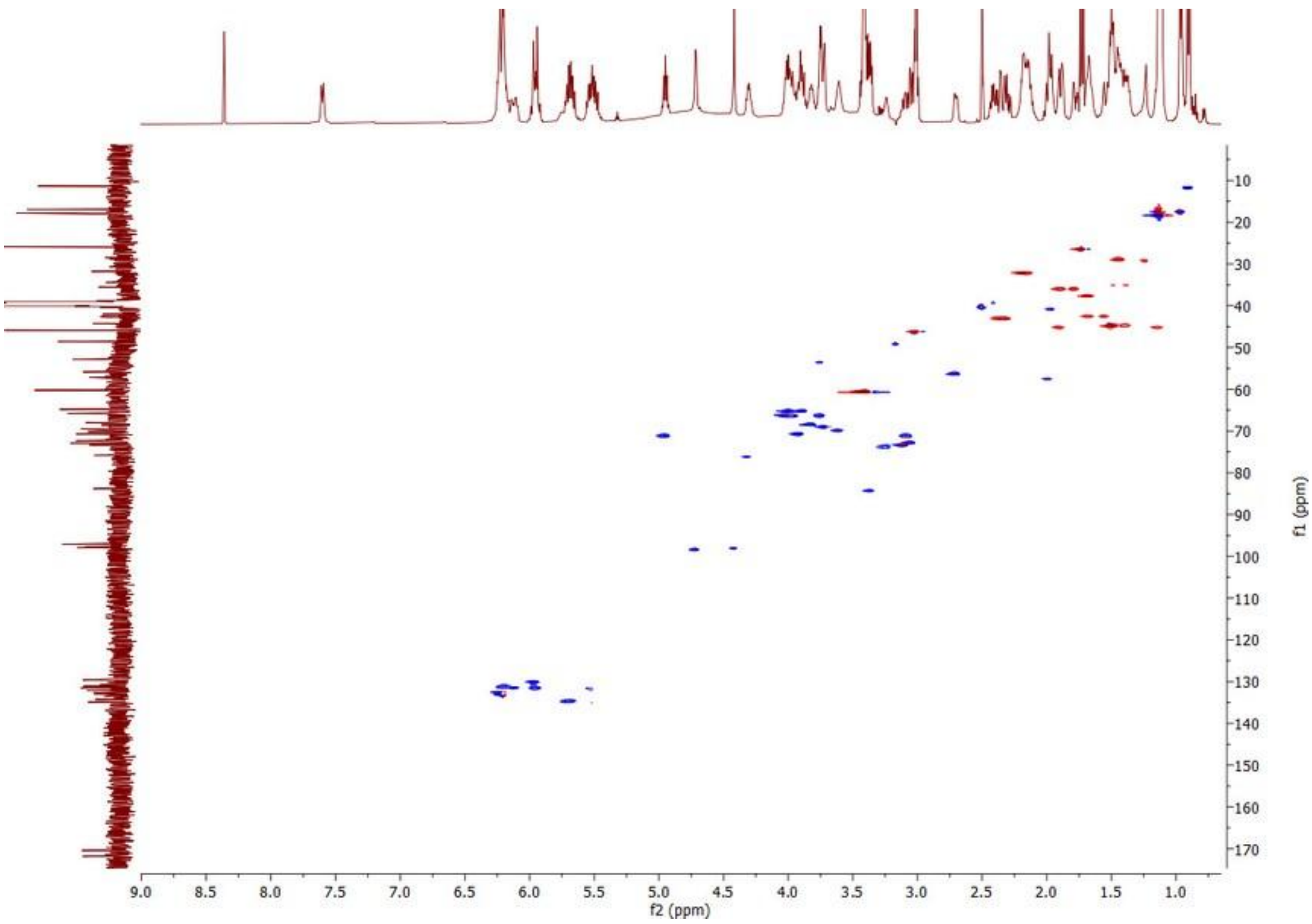

Supplementary Fig. 43. NMR data for Nys34 continued. (c) shows ^{13}C-NMR of Nys34 in DMSO-*d6*. **(d)** shows ^{1}H-^{13}C HSQC NMR of Nys34 in DMSO-*d6*.

e

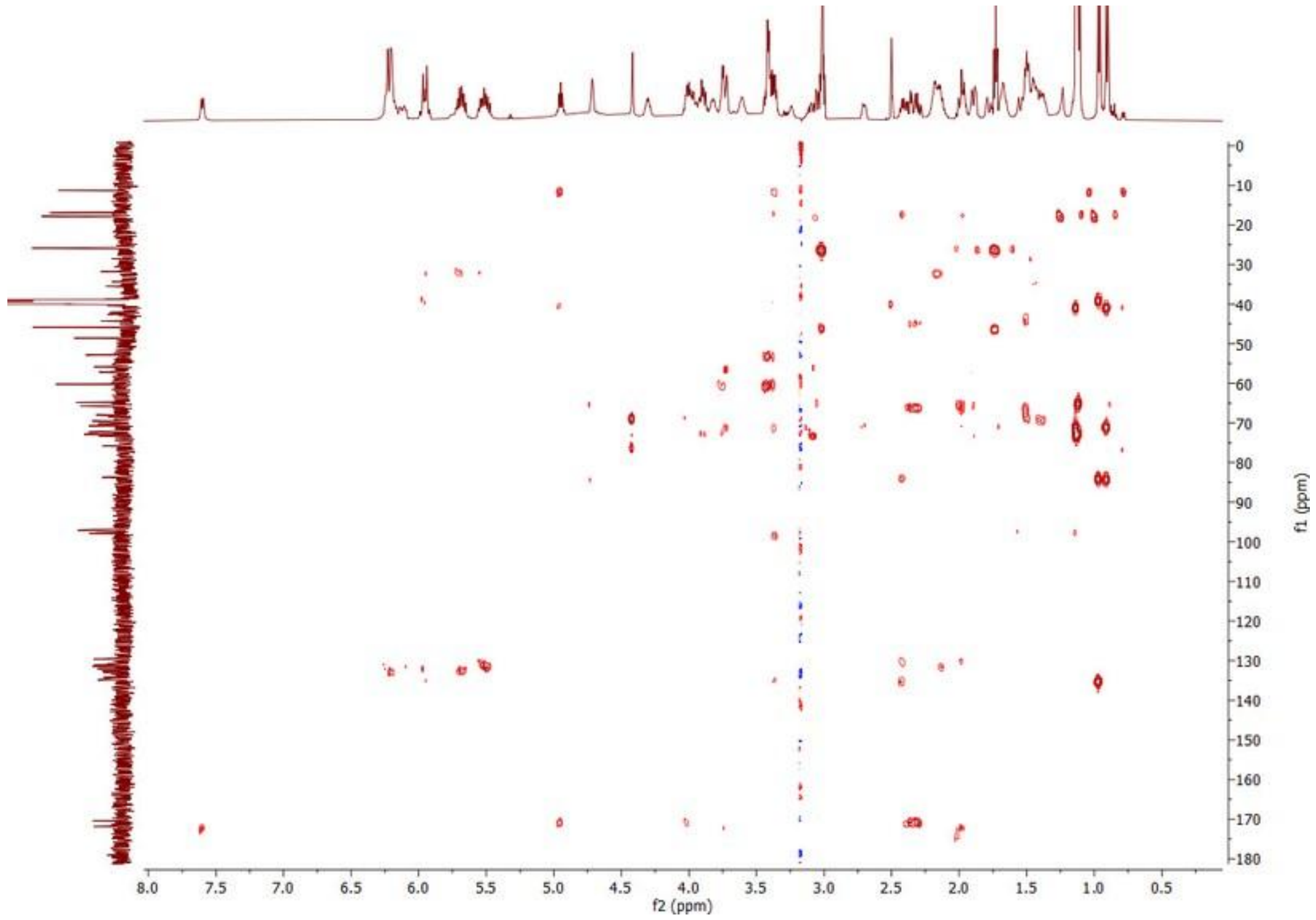

Supplementary Fig. 43. NMR data for Nys34 continued. (e) shows ^{1}H-^{13}C HMBC NMR of Nys34 in DMSO-*d6*.

Position	^{1}H found NysA3 in DMSO-d6	^{1}H found Nys31 in DMSO-d6	^{1}H found Nys34 in DMSO-d6	^{13}C found NysA3 in DMSO-d6	^{13}C found Nys34 in DMSO-d6
1				170.86	170.43
2	2.43	2.36	2.33	42.99	42.3
3	4.10	3.99	4.02	66.14	65.76
4	1.60	1.50	1.50	44.75	44.27
5	3.91	3.90	3.92	68.42	67.98
6	1.49	1.49	1.49	44.79	45.85
7	3.70	3.60	3.61	69.92	70.14
8	1.55	1.51	1.54	34.81	35.51
9	1.52	1.45	1.44	29.01	28.2
10	3.31	3.24	3.24	73.80	73.36
11	3.99	3.98	3.97	70.76	70.74
12	1.63,1.77	1.56,1.68	1.54, 1.66	42.52	42.54
13				97.62	97.10
14	1.24,1.97	1.13,1.88	1.13, 1.89	44.89	45.89
15	4.05	4.00	3.99	65.97	64.79
16	1.97	1.98	1.97	58.15	57.20
17	4.07	3.98	4.00	66.34	65.76
18	1.79,1.90	1.76,1.89	1.74, 1.90	37.87	35.51
19	4.44	4.30	4.30	75.57	75.80
20	5.84	5.68	5.70	134.98	134.8
21-26	6.21-6.29	6.10-6.25	6.1-6.23	130.15-133.37	129.63-133.2
27	5.79	5.67	5.65	134.65	134.3
28	2.27	2.18	2.18	32.19	31.76
29	2.24	2.15	2.14	32.23	31.76
30	5.63	5.52	5.52	131.91	132.08
31	6.04	5.96	5.97	131.55	131.45
32	6.04	5.94	5.94	131.55	131.09
33	5.58	5.51	5.50	135.32	134.86
34	2.50	2.49	2.50	39.23	40.37
34-CH_3	1.05	0.96	0.96	17.59	16.95
35	3.46	3.32	3.37	84.22	83.75
36	2.06	1.97	1.97	40.94	40.28
36-CH_3	1.00	0.89	0.90	11.82	11.32
37	5.02	4.95	4.94	71.22	70.74
37-CH_3	1.23	1.12	1.11	18.40	17.87
3′ NH_2	8.45	8.40	8.36		
-CONH-		7.33	7.61		
1′	4.57	4.36	4.41	97.84	97.71
2′	3.78	3.74	3.74	69.23	69.49
3′	2.77	2.63	2.69	56.51	55.86
4′	3.16	3.03	3.02	71.56	72.29
5′	3.24	3.05	3.07	73.30	73.4
6′	1.23	1.12	1.13	17.56	16.95
1″	4.81	4.73	4.71	98.38	97.89
2″	1.88,1.98	1.78,1.90	1.78,1.90	35.97	35.51
3″	3.84	3.90	3.89	66.22	65.76
4″	3.14	3.03	3.00	72.74	72.86
5″	3.98	3.98	3.98	65.20	64.79
6″	1.20	1.11	1.12	18.25	17.80
-CH_2 of Serinol			3.42		60.20
-CH of Serinol			3.70		52.86

Supplementary Fig. 44. ^{1}H NMR spectral data for NysA3 and its C16 derivatives

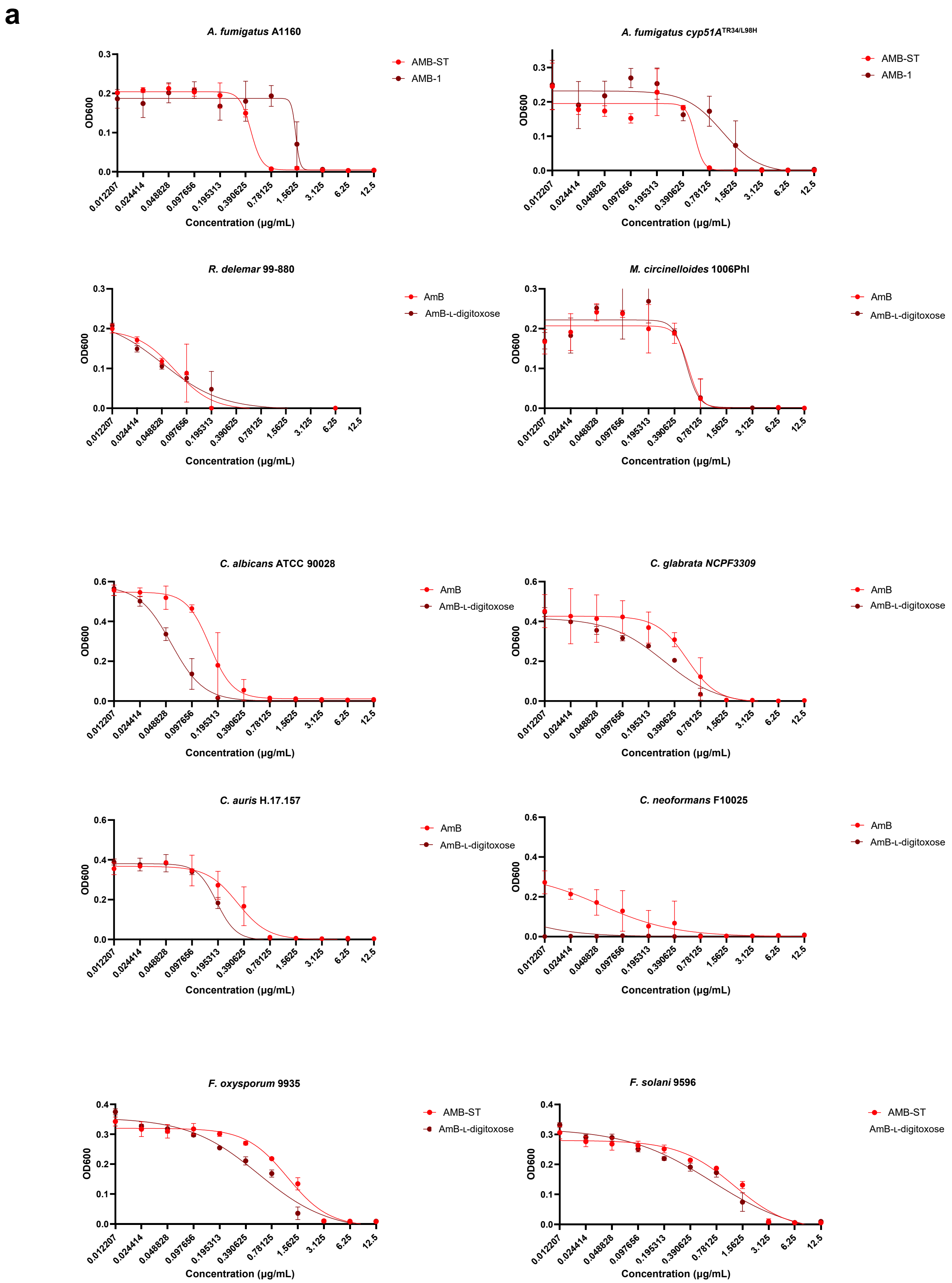

Supplementary Fig. 45. IC_{50} graphs. (a) shows IC_{50} graphs for AmB and its sugar derivative AmB-L-digitoxose against *A. fumigatus* A1160, *A. fumigatus cyp51A*$^{TR34/L98H}$, *R. delemar* 99-880, *M. circinelloides* 1006Phl, *C. albicans* 90028, *C. glabrata* NCPF3309, *C. auris* H.17.157, *C. neoformans* F10025, *F. oxysporum* 9935 and *F. solani* 9596. Measurements were done in biological triplicates with error bars shown as ± SD.

b

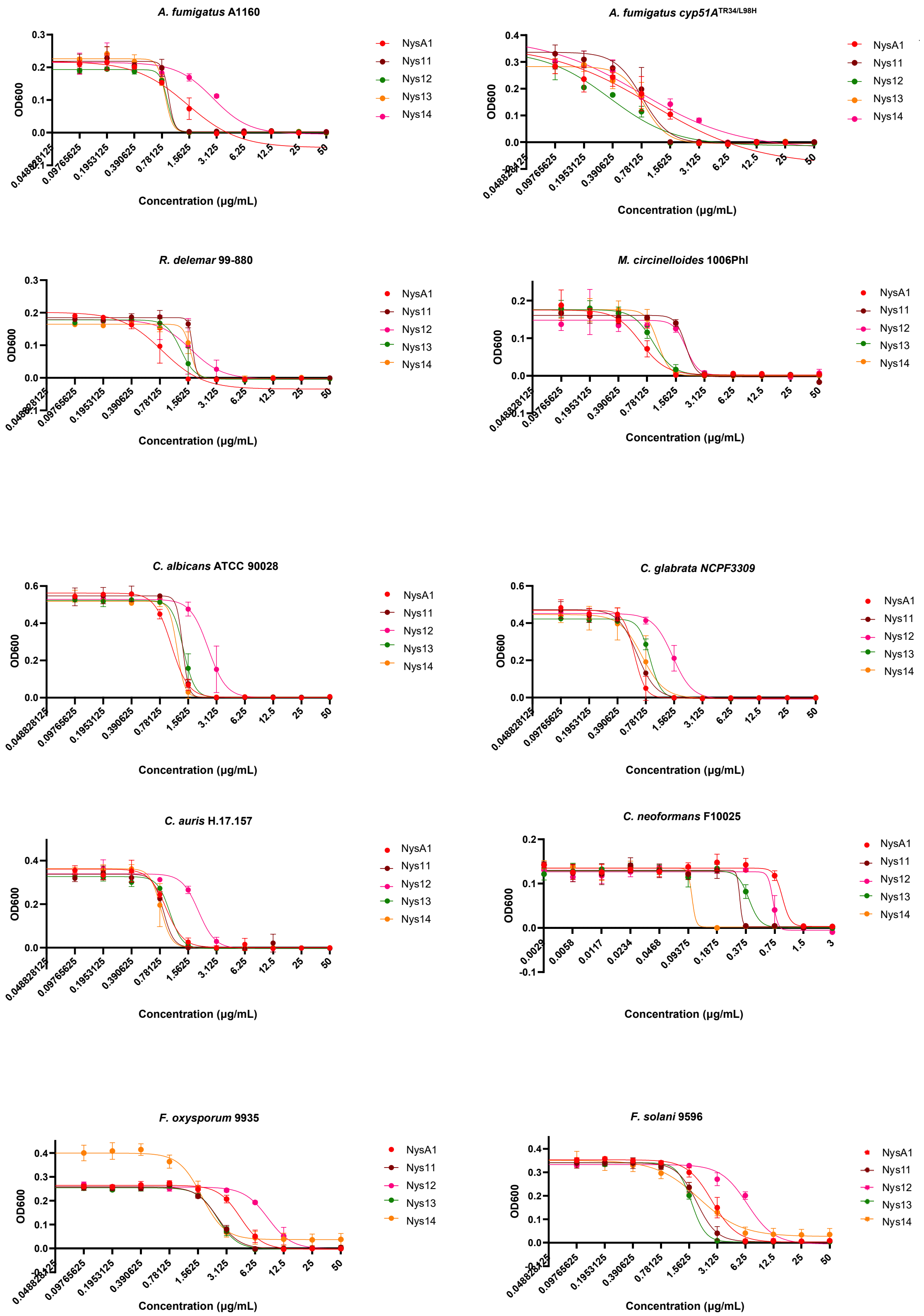

Supplementary Fig. 45. IC$_{50}$ graphs continued. (b) shows IC$_{50}$ graphs for NysA1 and its derivatives against *A. fumigatus* A1160, *A. fumigatus cyp51A*$^{TR34/L98H}$, *R. delemar* 99-880, *M. circinelloides* 1006Phl, *C. albicans* 90028, *C. glabrata* NCPF3309, *C. auris* H.17.157, *C. neoformans* F10025*, F. oxysporum* 9935 and *F. solani* 9596. Measurements were done in biological triplicates with error bars shown as ± SD.

c

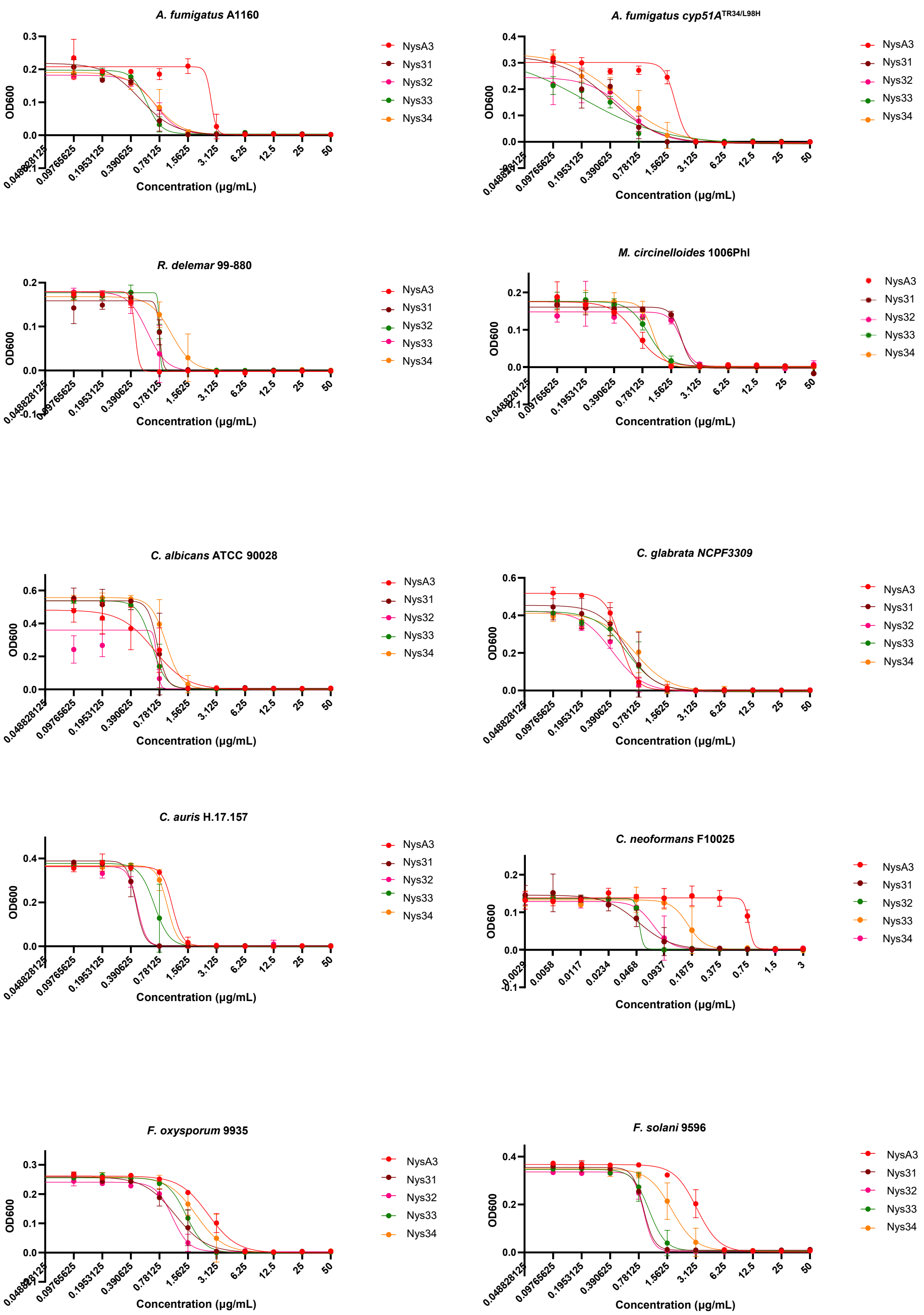

Supplementary Fig. 45. IC_{50} graphs continued. (c) shows IC_{50} graphs for NysA3 and its derivatives against *A. fumigatus* A1160, *A. fumigatus cyp51A*$^{TR34/L98H}$, *R. delemar* 99-880, *M. circinelloides* 1006Phl, *C. albicans* 90028, *C. glabrata* NCPF3309, *C. auris* H.17.157, *C. neoformans* F10025*, F. oxysporum* 9935 and *F. solani* 9596. Measurements were done in biological triplicates with error bars shown as ± SD.

d

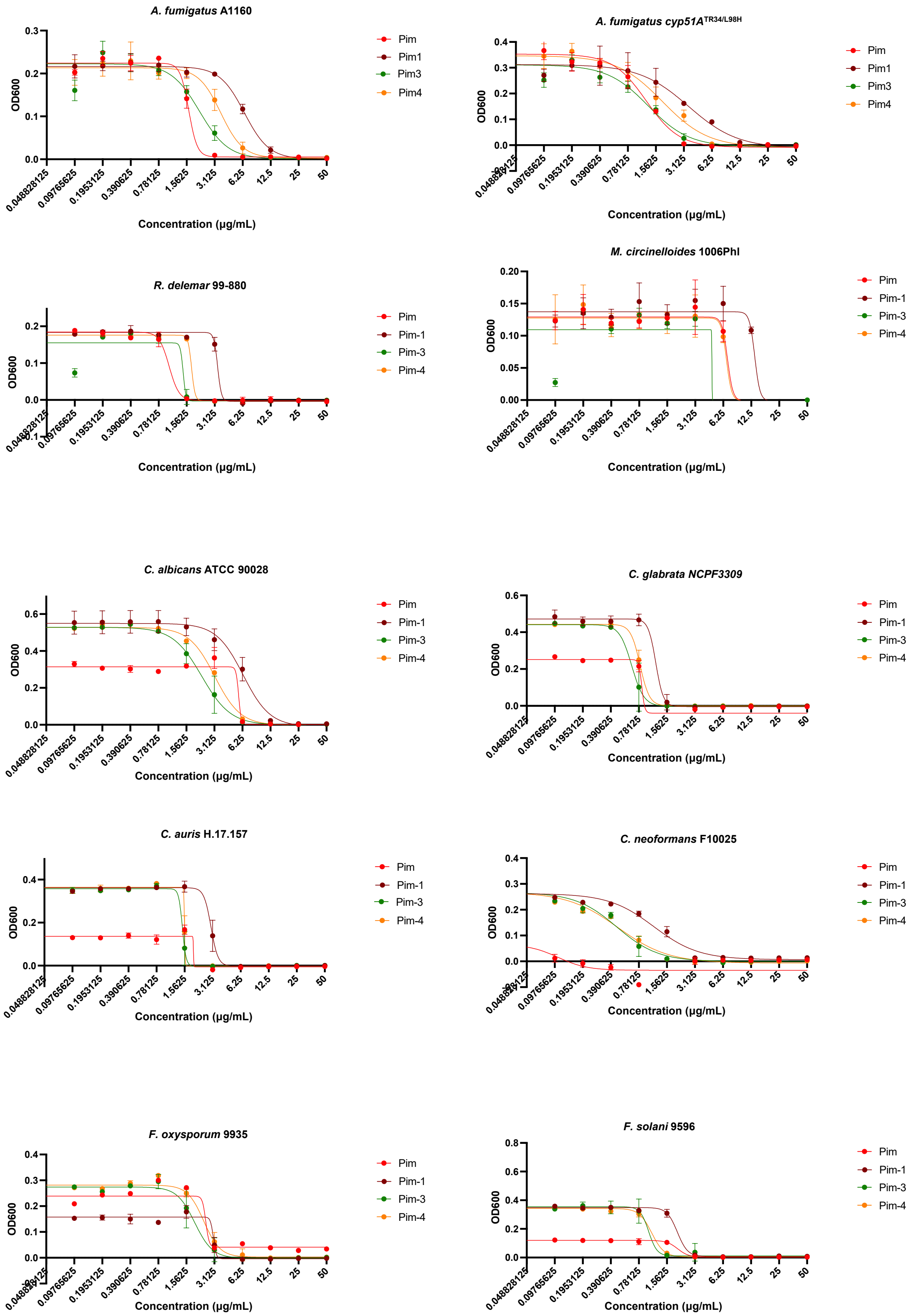

Supplementary Fig. 45. IC$_{50}$ graphs continued. (d) shows IC$_{50}$ graphs for Pim and its derivatives against *A. fumigatus* A1160, *A. fumigatus cyp51A*$^{TR34/L98H}$, *R. delemar* 99-880, *M. circinelloides* 1006Phl, *C. albicans* 90028, *C. glabrata* NCPF3309, *C. auris* H.17.157, *C. neoformans* F10025, *F. oxysporum* 9935 and *F. solani* 9596. Measurements were done in biological triplicates with error bars shown as ± SD.

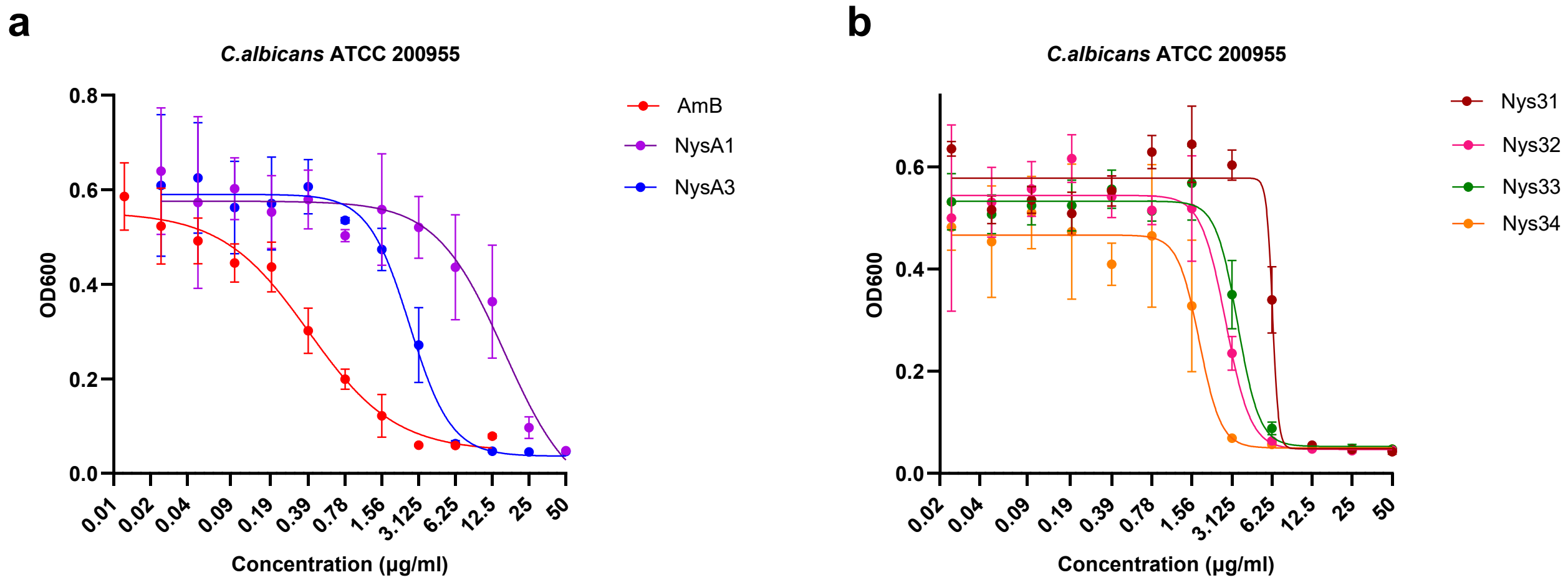

Supplementary Fig. 46. IC_{50} graphs of polyenes against drug-resistant *C. albicans* ATCC 200955. (a) shows IC_{50} graphs for AmB, NysA1 and NysA3, (**b)** shows IC_{50} graphs of NysA3 derivatives (Nys31, Nys32, Nys33 and Nys34). Measurements were done in biological triplicates with error bars shown as ± SD.

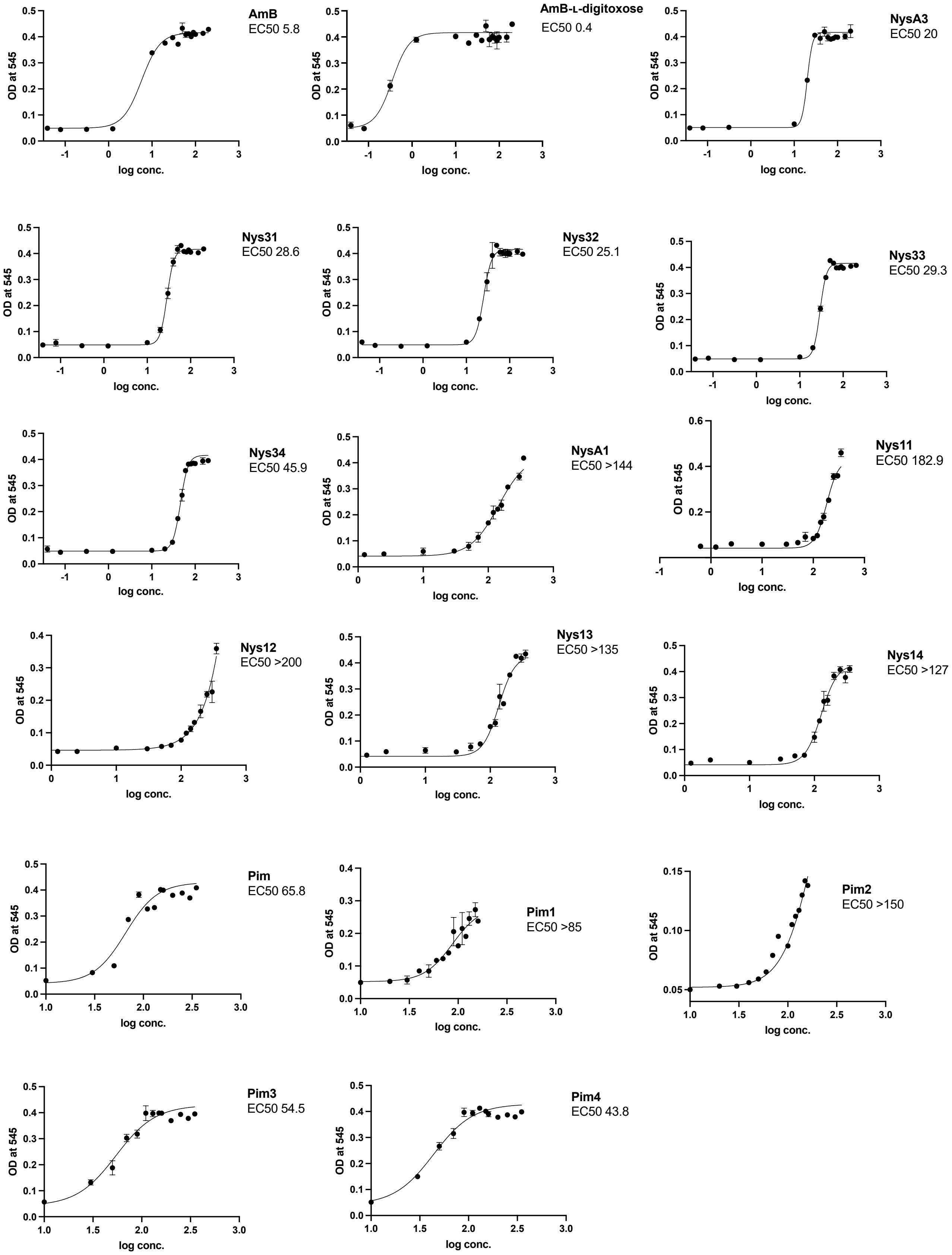

Supplementary Fig. 47. Horse blood haemolytic activity. The figure shows EC_{50} (μM) of AmB, AmB-L-digitoxose, NysA3, Nys31, Nys32, Nys33, Nys34, NysA1, Nys11, Nys12, Nys13, Nys14, Pim, Pim1, Pim2, Pim3 and Pim4. Data points are an average of three independent experiments with error bars shown as ± SD.

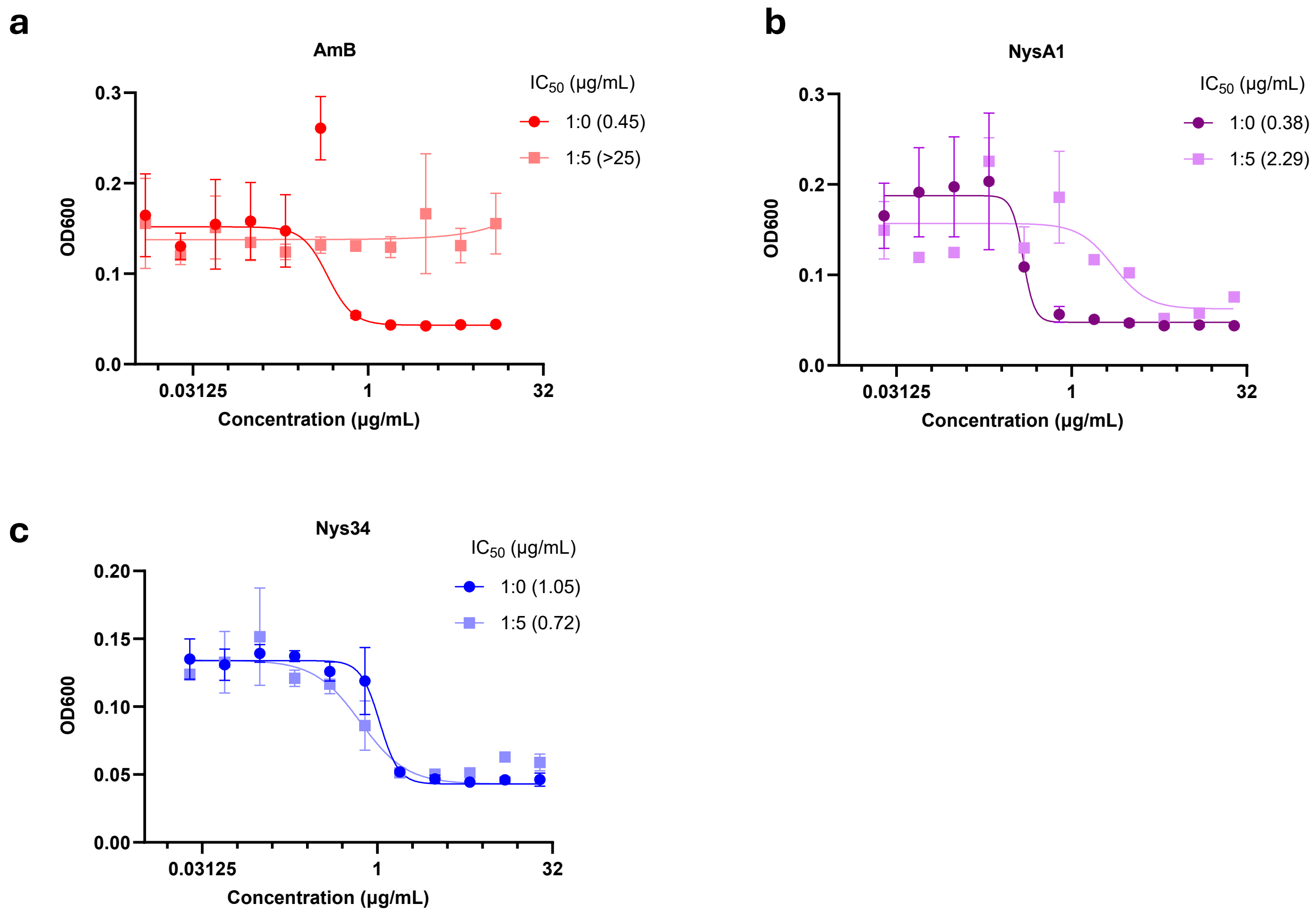

Supplementary Fig. 48. Effect of ergosterol pre-complexation on the antifungal activity of polyenes. The figure shows the effect of **(a)** AmB, **(b)** NysA1 and **(c)** Nys34 against *A. fumigatus* A1160 at 24 hrs. The polyene only dose response curves are shown in darker tones of red, purple and blue for AmB, NysA1 and Nys34, respectively. The dose response curves for polyene-ergosterol in 1:5 ratio are shown in lighter tones of red, purple and blue for AmB, NysA1 and Nys34, respectively. Measurements were done in biological triplicates with error bars shown as ± SD.

References

57. Gilchrist, C. L. M. et al. T. cblaster: A remote search tool for rapid identification and visualization of homologous gene clusters. *Bioinformatics Advances* 1, *1* (2021). https://doi.org/10.1093/bioadv/vbab016
58. Blin, K. et al. antiSMASH 8.0: Extended gene cluster detection capabilities and analyses of chemistry, enzymology. and regulation. *Nucleic Acids Research* 53, *1*, 32-38 (2025). https://doi.org/10.1093/nar/gkaf334
59. Zallot, R., Oberg, N., Gerlt, J. A. The EFI Web Resource for Genomic Enzymology Tools: Leveraging Protein, Genome, and Metagenome Databases to Discover Novel Enzymes and Metabolic Pathways. *Biochemistry* 58, *41*, 4169-4182 (2019). http://dx.doi.org/10.1021/acs.biochem.9b00735
60. ByteDance AML AI4Science Team, et al. Advancing Structure Prediction Through a Comprehensive AlphaFold3 Reproduction. *BioRxiv* (2025). https://www.biorxiv.org/content/10.1101/2025.01.08.631967
61. The PyMOL Molecular Graphics System, Version 3.0 Schrödinger, LLC. PyMOL | pymol.org
62. Koos, M. R. M., Kummerlöwe, G., Kaltschnee, L., Thiele, C. M., Luy, B. CLIP-COSY: A Clean In-Phase Experiment for the Rapid Acquisition of COSY-type Correlations. *Angew. Chem. Int. Ed.*, **55**, 7655 (2016). https://doi.org/10.1002/anie.201510938
63. Thiele, C. M., Petzold, K., Schleucher, J. EASY ROESY: Reliable Cross-Peak Integration in Adiabatic Symmetrized ROESY. *Chem. - Eur. J.* **15**, 585-588 (2009). https://doi.org/10.1002/chem.200802027
64. Borgos, S. E. F. et al. Probing the Structure−Function Relationship of Polyene Macrolides: Engineered Biosynthesis of Soluble Nystatin Analogues. *J. Med. Chem.* **49**, *8*, 2431–2439 (2006). https://doi.org/10.1021/jm050895w
65. Li, K., Guo, Z., Bai, L. Digitoxose as powerful glycosyls for building multifarious glycoconjugates of natural products and un-natural products. *Synth Syst Biotechnol*. **9**, *4*, 701-712 (2024). https://doi.org/10.1016/j.synbio.2024.05.012
66. Madduri, K., Waldron, C., Merlo, D. J. Rhamnose Biosynthesis Pathway Supplies Precursors for Primary and Secondary Metabolism in *Saccharopolyspora spinosa. J. Bacteriol.* **183**, *19*, 5632-5638 (2001). https://doi.org/10.1128/jb.183.19.5632-5638.2001
67. Caffrey P., Lynch S., Flood E., Finnan S., Oliynyk M., Amphotericin biosynthesis in Streptomyces nodosus: deductions from analysis of polyketide synthase and late genes. *Chem. Biol.* **8**, 713–723 (2001). https://doi.org/10.1016/S1074-5521(01)00046-1
68. Lairson, L.L, Henrissat, B., Davies, G.J., Withers, S.G. Glycosyltransferases: Structures, Functions, and Mechanisms. *Annu. Rev. Biochem*. **77**, *1*, 521-555 (2008). https://doi.org/10.1146/annurev.biochem.76.061005.092322
69. Yang, Y. et al. Development of Starch-Based Antifungal Coatings by Incorporation of Natamycin/Methyl-*β*-Cyclodextrin Inclusion Complex for Postharvest Treatments on Cherry Tomato against *Botrytis cinerea*. *Molecules* **24**, 3962 (2019). https://doi.org/10.3390/molecules24213962
