# Supplementary material for: Enzymatic glycosylation and amidation reshapes polyene bioactivity
Source: Nature. 2026 Jul 29;657(8130):295–303. doi: 10.1038/s41586-026-10834-8 (PMC13538060; doi:10.1038/s41586-026-10834-8)
Supplement: Supplementary file 2 [file 41586_2026_10834_MOESM2_ESM.pdf]

nature portfolio

Corresponding author(s): Jason Micklefield

Last updated by author(s): 16-06-2026

Reporting Summary

Nature Portfolio wishes to improve the reproducibility of the work that we publish. This form provides structure for consistency and transparency in reporting. For further information on Nature Portfolio policies, see our Editorial Policies and the Editorial Policy Checklist.

Statistics

For all statistical analyses, confirm that the following items are present in the figure legend, table legend, main text, or Methods section.

n/a	Confirmed	
☐	☒	The exact sample size (*n*) for each experimental group/condition, given as a discrete number and unit of measurement
☐	☒	A statement on whether measurements were taken from distinct samples or whether the same sample was measured repeatedly
☐	☒	The statistical test(s) used AND whether they are one- or two-sided<br>*Only common tests should be described solely by name; describe more complex techniques in the Methods section.*
☒	☐	A description of all covariates tested
☒	☐	A description of any assumptions or corrections, such as tests of normality and adjustment for multiple comparisons
☐	☒	A full description of the statistical parameters including central tendency (e.g. means) or other basic estimates (e.g. regression coefficient) AND variation (e.g. standard deviation) or associated estimates of uncertainty (e.g. confidence intervals)
☒	☐	For null hypothesis testing, the test statistic (e.g. *F*, *t*, *r*) with confidence intervals, effect sizes, degrees of freedom and *P* value noted<br>*Give P values as exact values whenever suitable.*
☒	☐	For Bayesian analysis, information on the choice of priors and Markov chain Monte Carlo settings
☒	☐	For hierarchical and complex designs, identification of the appropriate level for tests and full reporting of outcomes
☒	☐	Estimates of effect sizes (e.g. Cohen's *d*, Pearson's *r*), indicating how they were calculated

Our web collection on statistics for biologists contains articles on many of the points above.

Software and code

Policy information about availability of computer code

Data collection	LC-HRMS was performed on an Agilent 1290 Infinity II, coupled to a 6560 Ion Mobility Q-TOF + 1300 UPLC instrument and 1290 Infinity II HPLC coupled to 6546 LC/Q-TOF (Agilent Technologies).<br>Compound structures were confirmed by TopSpin v3.6.3 & IconNMR (NMR) v5.0.11.<br>DNA sequencing was performed using services from Eurofins Genomics. De novo whole genome sequencing was performed using a Sequel I PacBio instrument.<br>Samples were centrifuged using Eppendorf 5810R. Bacterial cultures were grown using New Brunswick Innova 44 shaker incubator.<br>Synergy HT Microplate Reader was used for cell viability assays and for monitoring bacterial growth. ThermoFisher Nanodrop Ultra was used to quantify DNA and protein. PCR was performed on Eppendorf Mastercycler Nexus GX2.
Data analysis	MestReNova v11 (NMR), Shimadzu LabSolutions v 5.132, ChemDraw, Professional v21 (chemical structures and exact mass calculations), PyMOL v2.5.7 (protein structure), Alphafold2, GraphPad Prism version 10.2.3 (347), nlmixr2 (5.0.0), dbCAN3 v5.2.0, HMMER v3.4, BLAST tool (NIH) and BLASTp, Clustal Omega (EMBL-EBI), MEGA12 version 12.1.1, interactive tree of life tool (iTOL) version 7.6, EFI-GNT (Enzyme Function Initiative Genome Neighbourhood Tool) version 2025_04, antiSMASH (bacterial version 8.0).

For manuscripts utilizing custom algorithms or software that are central to the research but not yet described in published literature, software must be made available to editors and reviewers. We strongly encourage code deposition in a community repository (e.g. GitHub). See the Nature Portfolio guidelines for submitting code & software for further information.

Data

Policy information about availability of data

All manuscripts must include a data availability statement. This statement should provide the following information, where applicable:

- Accession codes, unique identifiers, or web links for publicly available datasets
- A description of any restrictions on data availability
- For clinical datasets or third party data, please ensure that the statement adheres to our policy

The whole genome sequences of Streptomyces kasugaensis DSM 40819 and Streptomyces netropsis DSM 40846 (Streptomyces syringium DSM 40846) were submitted to DDBJ/ENA/GenBank and released under the accession numbers JBNAKK000000000 and JBNCJY000000000, respectively. Proteins characterized in this study can be accessed from UniProt using the accession codes presented in Supplementary Figure S2 and amino acid sequences of the enzymes used in this study are presented in Supplementary Excel sheet. All the remaining data are available in the main text or the supplementary materials. Correspondence and requests for materials should be addressed to J.M.

Research involving human participants, their data, or biological material

Policy information about studies with human participants or human data. See also policy information about sex, gender (identity/presentation), and sexual orientation and race, ethnicity and racism.

Reporting on sex and gender	N/A
Reporting on race, ethnicity, or other socially relevant groupings	N/A
Population characteristics	N/A
Recruitment	N/A
Ethics oversight	N/A

Note that full information on the approval of the study protocol must also be provided in the manuscript.

Field-specific reporting

Please select the one below that is the best fit for your research. If you are not sure, read the appropriate sections before making your selection.

☒ Life sciences ☐ Behavioural & social sciences ☐ Ecological, evolutionary & environmental sciences

For a reference copy of the document with all sections, see nature.com/documents/nr-reporting-summary-flat.pdf

Life sciences study design

All studies must disclose on these points even when the disclosure is negative.

Sample size

Quantitative assays were performed in triplicate and mean and standard deviation values calculated. The sample size (50-100uL) chosen is based on our past experience with the in vitro experiments and three times were sufficient as the results are highly reproducible. This is inline with what has been reported in citation 51 Zhang, C. et al ChemBioChem, 9: 2506-2514 (2008).
The IC50 (against fungi and yeast) and EC50 (horse blood haemolysis) in 100-200 uL serial dilurions in 96 well plate in triplicates as per guidelines from citation 54 European Committee for Antimicrobial Susceptibility Testing. E.DEF 7.4 (2023).
For mouse pharmacokinetic (PK) analysis of the Nys34 compounds was performed with 20 CD1 male and female mice (10 males and 10 females of ~20 g). Blood microsamples were taken twice per mouse from the tail vein in a heparinised capillary tube and a third terminal cardiac puncture sample was taken from each mouse to limit the number of mice used for this experiment in line with the 3R's principles. Microsamples from mice were taken at 0.5, 1, 2, 4 and 8 hrs post dosing with the cardiac punctures performed at the 8 and 24 hrs post dosing time points following the scheme outlined previously. Sample sizes followed previous published pk studies as shown in citation 55 Chapman, K. et al. Drug discovery today 19, 5, (2014).
For mouse infection model group sizes were defined by the expected size of the change in CFUs and have been informed by prior pilot data. Using pooled standard error (based on the analysis of the ratio of the geometric means of triplicate data sets) we predict that 50% reduction in CFUs can be determined with 10 mice per group (80% power). In these experiments 9-10 mice per group were used.

Data exclusions

Outliers or poor data points from IC50 graphs (Hek293 and HepG2) and antifungal testing were discarded, otherwise no data was excluded.

Replication

The IC50 (against fungi and yeast) and EC50 (horse blood haemolysis) were performed in three biological replicates where each data point represents the mean and error bars represent ± SD. All in vitro quantitative assays were performed in triplicates as independent experiments. This was in accordance with previous work reported by Maji, A., et al. Nature. 1–7 (2023).

Randomization	For tests involving each cell line and horse blood, compounds at different dilutions were added to randomly assigned rows on 96 well plates. Mice were randomly allocated to each treatment group.
Blinding	For in vitro assays with glycosyltransferases or amidotransferase, blinding could not be applied due to specific reaction conditions for each and for analysis of the assays blinding was not necessary as readings were taken from the instruments such as HPLC and LCMS.<br>For cell culture toxicity testing blinding to cell lines was not used due to different morphologies of the cultured cell lines and as different subculturing practice was required due to the different proliferation rates of each specific cell line. All measurements were performed by the instrument (plate reader) so blinding was not necessary.<br>Mouse infection experiments were carried out by an infection technician. CFU counts on blinded samples were performed by another individual. Data was unblinded post logging of CFU data.

Reporting for specific materials, systems and methods

We require information from authors about some types of materials, experimental systems and methods used in many studies. Here, indicate whether each material, system or method listed is relevant to your study. If you are not sure if a list item applies to your research, read the appropriate section before selecting a response.

Materials & experimental systems

n/a	Involved in the study
☒	☐ Antibodies
☐	☒ Eukaryotic cell lines
☒	☐ Palaeontology and archaeology
☐	☒ Animals and other organisms
☒	☐ Clinical data
☒	☐ Dual use research of concern
☒	☐ Plants

Methods

n/a	Involved in the study
☒	☐ ChIP-seq
☒	☐ Flow cytometry
☒	☐ MRI-based neuroimaging

Eukaryotic cell lines

Policy information about cell lines and Sex and Gender in Research

Cell line source(s)	Human cell lines including A549 (alveolar basal epithelial), HEK293 (embryonic kidney) and HEPG2 (hepatoma), were sourced from Sigma-Aldrich (Merck).
Authentication	Cells were used as received, without further authentication
Mycoplasma contamination	The tissue culture facility that was used is routinely checked for Mycoplasma contamination. Individual cell lines (A549, HEK293, HEPG2) also tested negative for the presence of Mycoplasma before starting experiments.
Commonly misidentified lines (See ICLAC register)	No commonly misidentified cell lines were used.

Animals and other research organisms

Policy information about studies involving animals; ARRIVE guidelines recommended for reporting animal research, and Sex and Gender in Research

Laboratory animals	CD1 mice from Charles River UK were ordered at 16-18g. Mice were between 4 and 5 weeks old at the time of infection. The mice were quarantined and acclimatised to the facility for a minimum of one week prior to any regulated procedures taking place. Mouse husbandry was performed in individually ventilated cages on a 12-hour light and dark cycle with temperature regulated to 21 °C (±2 °C) and 40-50% humidity, mice were kept in cages containing nesting material and wood chips. Access to water and food was provided ad libitum for the entirety of the study. Pharmacokinetic (PK) analysis was performed with 20 CD1 male and female mice (10 males and 10 females, of ~20 g)and 3R's principle was applied. Population PK modelling and Monte Carlo simulations were performed in R using nlmixr2 to predict non-toxic regimens prior to carrying out efficacy experiments.<br>For efficacy experiments requiring infections with Aspergillus fumigatus conidia; mice were immunocompromised 24 hrs prior to infection by subcutaneous injection of Kenalog (Triamcinolone Acetonide, Bristol Myers Squibb) at 40 mg/Kg, on the introduction of immunosuppression the water was supplemented with 2 g/L antibiotic (Neomycin, Sigma) and changed daily. Mice were infected intranasally with freshly prepared 5x10^5 spores of CEA10 in 40 µl of sterile saline. 10 CD1 (5 male and 5 female) mice were used in each treatment arm of the efficacy experiments.
Wild animals	No wild animals were used.
Reporting on sex	Initial toxicity studies using one mouse for each drug and concentration were performed on male mice only to reduce the total number of animals used. All further subsequent tests were performed on equal numbers of male and female mice. No assessment on any potential sex based efficacy differences were performed.

Field-collected samples | The study did not involve field collected animals or samples.

Ethics oversight | Ethics were approved by the University of Manchester animal facility

Note that full information on the approval of the study protocol must also be provided in the manuscript.

Plants

Seed stocks | *Report on the source of all seed stocks or other plant material used. If applicable, state the seed stock centre and catalogue number. If plant specimens were collected from the field, describe the collection location, date and sampling procedures.*

Novel plant genotypes | *Describe the methods by which all novel plant genotypes were produced. This includes those generated by transgenic approaches, gene editing, chemical/radiation-based mutagenesis and hybridization. For transgenic lines, describe the transformation method, the number of independent lines analyzed and the generation upon which experiments were performed. For gene-edited lines, describe the editor used, the endogenous sequence targeted for editing, the targeting guide RNA sequence (if applicable) and how the editor was applied.*

Authentication | *Describe any authentication procedures for each seed stock used or novel genotype generated. Describe any experiments used to assess the effect of a mutation and, where applicable, how potential secondary effects (e.g. second site T-DNA insertions, mosiacism, off-target gene editing) were examined.*
