# Supplementary material for: Enzymatic glycosylation and amidation reshapes polyene bioactivity
Source: Nature. 2026 Jul 29;657(8130):295–303. doi: 10.1038/s41586-026-10834-8 (PMC13538060; doi:10.1038/s41586-026-10834-8)
Supplement: Supplementary file 3 [file 41586_2026_10834_MOESM3_ESM.pdf]

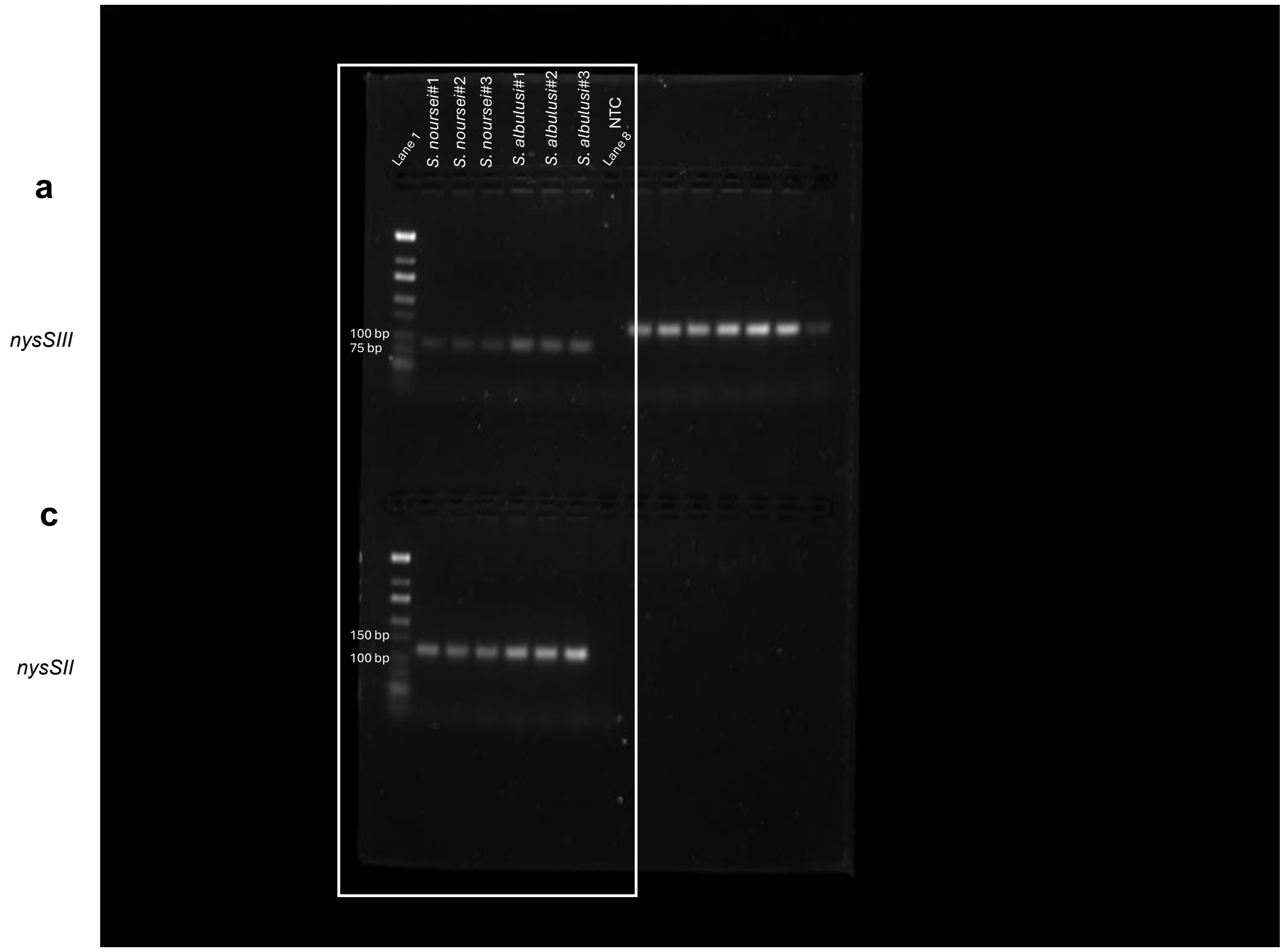

Raw gel for Supplementary Fig. 11a and 11c. Products of RT-qPCR assays ran on a 4% (w/v) agarose gel for *nysSIII* and *nysSII,* alongside no-template controls in lane 8 for **(a)** and **(c)**. Expected band sizes are 84 bp and124 bp, respectively. TriDye™ Ultra Low Range DNA Ladder was used for reference in lane 1. The gel outside of lane 8 was cropped (removed). The section used for this study is delineated by the white rectangle.

b

nysSVII

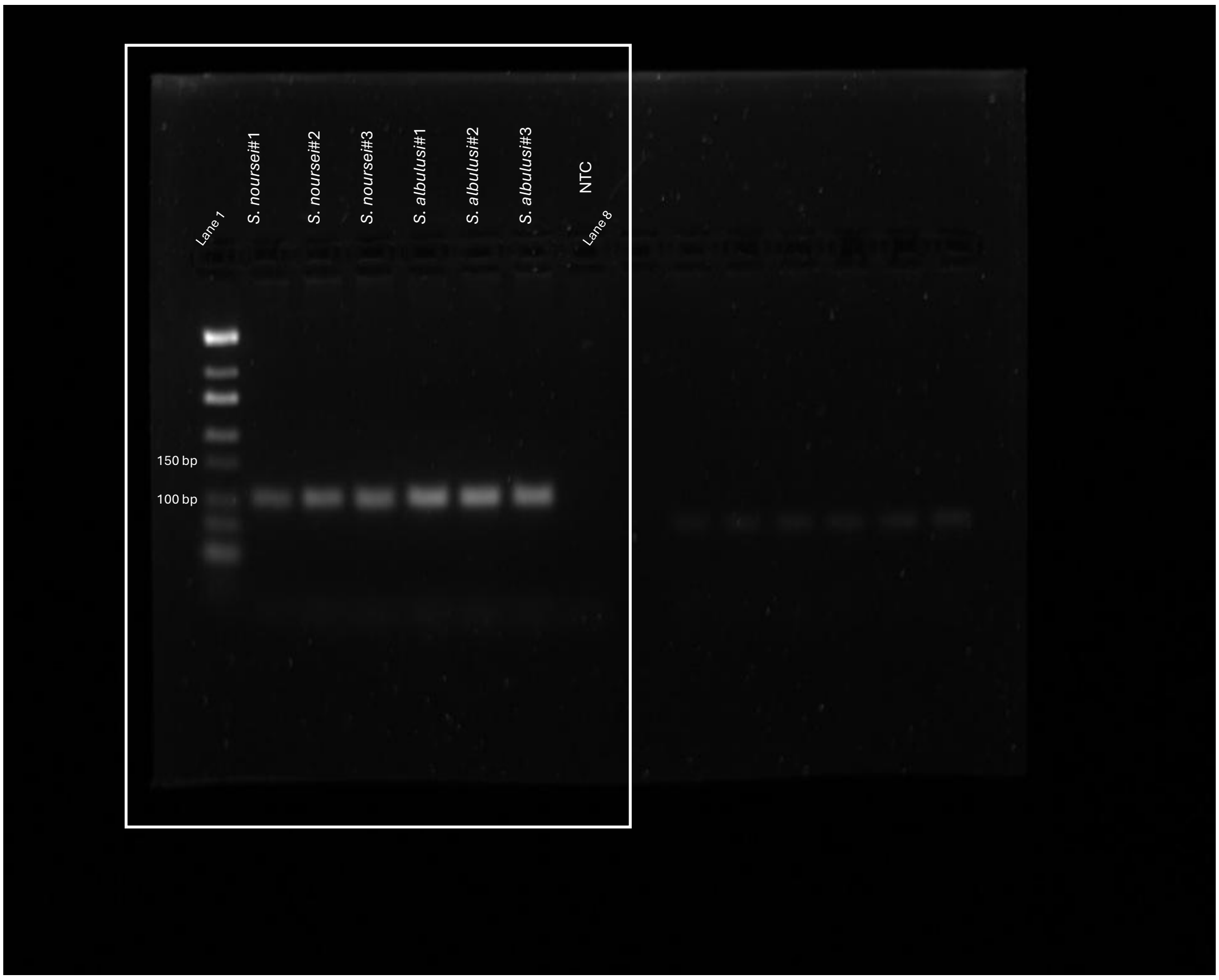

Raw gel for Supplementary Fig. 11b. Products of RT-qPCR assays ran on a 4% (w/v) agarose gel for *nysSVII,* alongside no-template control in lane 8. Expected band size is109 bp. TriDye™ Ultra Low Range DNA Ladder was used for reference in lane 1. The gel outside of lane 8 was cropped (removed). The section used for this study is delineated by the white rectangle.

d

16S rRNA

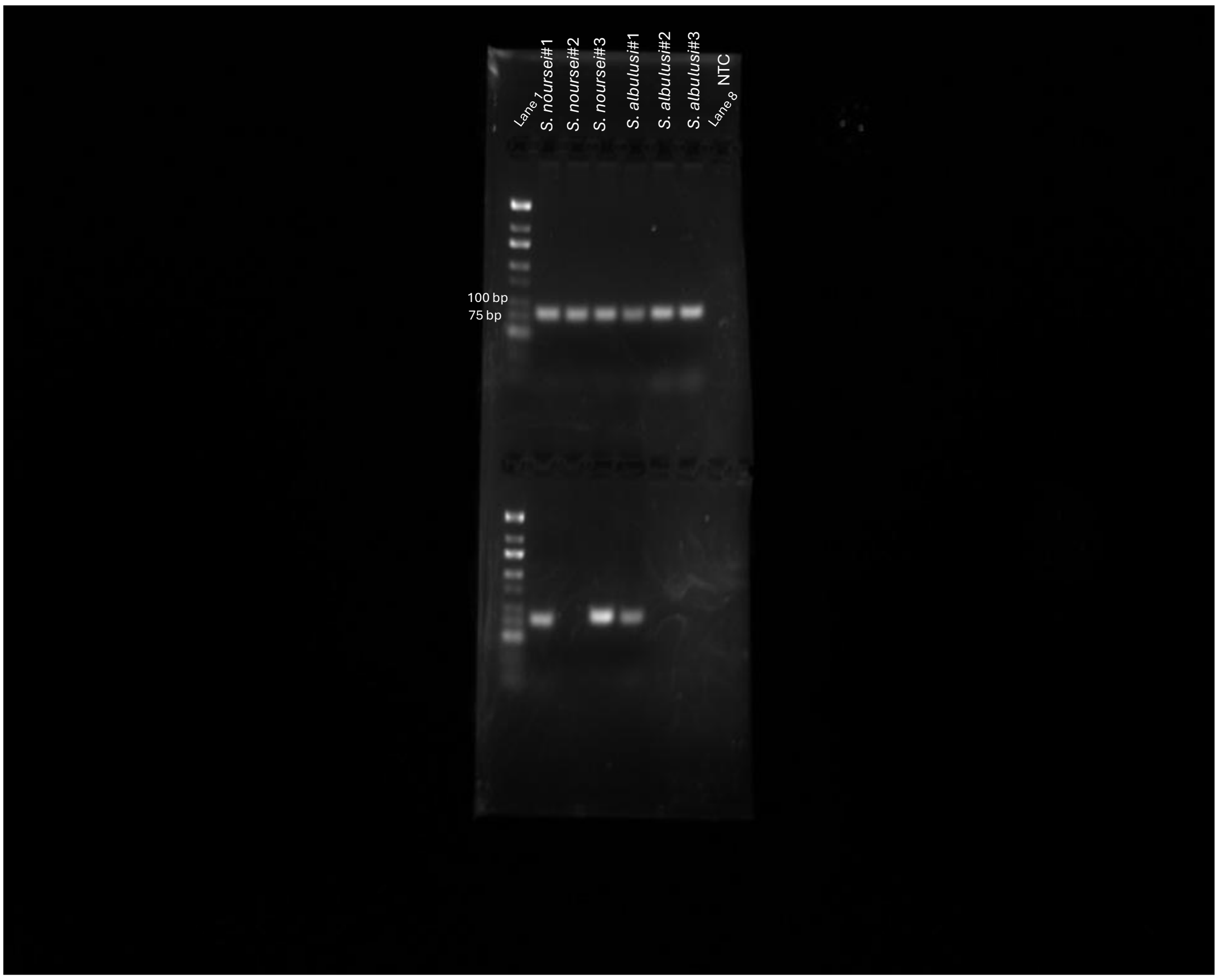

Raw gel for Supplementary Fig. 11d. Products of RT-qPCR assays ran on a 4% (w/v) agarose gel for 16S rRNA internal control. Expected band size is 80 bp. The no-template control was run in lane 8. TriDye™ Ultra Low Range DNA Ladder was used for reference in lane 1. The bottom gel was cropped (removed).
