# Supplementary material for: Enzymatic glycosylation and amidation reshapes polyene bioactivity
Source: Nature. 2026 Jul 29;657(8130):295–303. doi: 10.1038/s41586-026-10834-8 (PMC13538060; doi:10.1038/s41586-026-10834-8)
Supplement: Supplementary file 4 [file 41586_2026_10834_MOESM6_ESM.pdf]

nature portfolio

Peer Review File

Enzymatic glycosylation and amidation reshapes polyene bioactivity

Corresponding Author: Professor Jason Micklefield

Any redactions in this file are there to maintain patient confidentiality, the confidentiality of unpublished data, or to remove third-party material.

This file contains all reviewer reports in order by version, followed by all author rebuttals in order by version.

Version 0:

Reviewer comments:

Referee #1

(Remarks to the Author)
This manuscript tackles the important area of macrolide antifungal discovery and development, an urgent area given the dearth of new work in this area and challenges with accessing these large, complex molecules, problems that are urgent to address given the rise in fungal infections. The key contribution / discovery is of the new glycosyltransferase that guides discovery of new molecules via genome mining, and these molecules in some cases possesses enhanced bioactivity compared to AmB. The manuscript is very long with variable level of experimental support for some of the claims, and the I think the revision can work to streamline the logic especially for readers outside of this area and potentially go more in-depth on areas where the results are fully understood and leave out results that are less solid.

1) They state that "no genes present in the BGC, or that have been identified elsewhere on the S. noursei genome that might account for the second sugars in NysA3 and Nys1070"; however, the authors also do not expand on why is was so hard for others to find a glycosyltransferase in this genome (they also do not cite this sentence, making it hard to understand who overlooked this previously). I note that accession number provided in Supplementary Figure 2 is an entry that is already annotated as a glycosyl transferase and the authors in the next sentence state that "Analysis of the S. noursei genome sequence revealed a candidates GT …"

Given this is the key discovery in the paper, the authors need to provide some further insight into either what was so challenging for others to miss this gene, and explain why they were successful in this quest. (Are there many GTs in this genome, for example, making it hard to link a specific one?)

Is this GT unusual in some way …? The sub-title reads "novel polyene glycosyltransferases" yet it is somehow still unclear why these are "novel."

2) Similarly, the authors state that they found three GTs through wider BLAST searches, but I'm quite surprised that they only found three. I believe some information is missing – perhaps they meant they only selected three for analysis (among many)?

Would the authors see if they might have missed any? If they can do some bioinformatics to look at the distribution and genome neighborhoods analysis and put into the SI, this would give a better sense of the distribution of this gene and the potential for genome-guided discovery of new antifungals.

3) Ref 34/35 - The location of the mini-gene cluster outside of the "main" gene cluster is certainly notable, but there are many such examples in the literature on secondary metabolism, way more than "a few."

4) Is it possible to provide direct evidence that the mini-gene cluster indeed produces TDP-L-digitoxose? Or elaborate why you think it produces this product for the general reader?

5) Similarly, the authors could directly prove that nysSV is linked to NysA3 production via gene deletion and

complementation studies. Furthermore, if I followed your logic in the sentence “Unclear why NysA3 is the major product in S. albus, …” does transcript analysis provide any insight?

6) The name “mycoheptin A” is confusing, since you already have a “mycoheptin.” Based on what I read, I was quite confused by this structure. A full structure is provided, but if I understand correctly, you are assigning a structure based on high-resolution mass analysis, which is inconsistent with standards in the field of natural products and wouldn’t enable publication of this structure in specialist venues. (I make this note as an example, please check for other examples where you may have over-assigned structural information based on the available structural data or bioinformatic analysis)

Minor comments

7) A number of chromatograms lack information about how peaks were assigned (as chemical standard traces are not shown), what type of analysis is presented (EIC or TIC?) (e.g. Fig 2b).

8) “the subcluster containing MycS3” “the subcluster encoding MycS3 …”?

Referee #2

(Remarks to the Author)
I find this manuscript highly interesting and a significant, timely contribution to antifungal drug development, particularly considering the growing global resistance to current therapies and the toxicity limitations of widely used polyenes such as amphotericin B (AmB) and nystatin A1 (NysA1). These broad-spectrum antifungals are considered last-resort treatments; notably, AmB is the only polyene used for systemic fungal infections. However, its significant nephrotoxicity remains a major clinical concern. Given these challenges, the subject matter is both timely and highly significant, warranting publication in Nature provided the reviewers' comments are adequately addressed.

The study delivers substantial advances by identifying and characterizing novel biosynthetic enzymes, specifically glycosyl-transferases and amido-transferases (GTs), that enable modular and efficient derivatization of polyene scaffolds. Through a combination of genome mining, heterologous expression, in vitro enzymology, and fermentation-based production (which is the most advantageous strategy for active pharmaceutical ingredient production when such a possibility exists), the authors demonstrate that these enzymes can be exploited to introduce both sugar and carboxylate modifications that enhance antifungal efficacy, improve solubility, and significantly reduce mammalian toxicity. These attributes are key to overcoming the limitations of current polyene antifungal agents.

One of the study’s strongest features is the discovery of glycosyltransferases such as KfuSV, MycS3, and SnNysSV, which show broad substrate tolerance and can glycosylate clinically relevant polyenes both in vitro and in vivo. Identifying these enzymes not only clarifies the biosynthetic origins of rare polyenes like NysA3 and selvamicin but also enables an impressive diversity of enzymatic glycosylation strategies.

The authors show that expressing GT clusters in heterologous hosts can produce glycosylated polyene derivatives, including novel AmB analogues, offering a scalable approach for generating improved antifungal agents. Additionally, the discovery and structural characterization of previously unknown polyenes such as kasufungins and mycopentins expands the known range of antifungal macrolides, adding significant novelty to the manuscript.

The functional re-evaluation of the amido-transferase PcsA, which previous reports had deemed incompatible with larger polyenes, is another significant advancement. Here, the authors not only purify PcsA by inserting a His-tag into a flexible loop but also show it can efficiently catalyze C16-carboxylate derivatization on both NysA1 and NysA3, generating amide, hydroxamate, and acylhydrazine derivatives. I find the ability to replace the carboxyl group through enzymatic methods, rather than via complex synthetic routes, to offer an important and high useful improvement.

It would further enrich the study to address other underexplored polyene scaffolds such as filipin and natamycin, both of which represent structurally distinct yet clinically and mechanistically relevant antifungal agents.

The study demonstrates that sugar-modified AmBS and NysA3 analogues exhibit enhanced antifungal activity relative to their parent polyenes across a panel of clinically relevant and WHO-priority fungal pathogens, including C. albicans, C. auris, C. neoformans, and resistant strains such as A. fumigatus TR34/L98H. Of note, the specific pathogen identities and assay details are found in the supplementary The specific strain information is provided in the supplementary material but is not clearly presented alongside Figure 5, which limits the immediate interpretability and impact of the main data.

Another simple yet important observation is the apparent ability of the modified compounds to combat or reduce fungal tolerance, an effect that becomes more pronounced after 48 hours of incubation. Moreover, it would be of interest to test these compounds directly against strains with known high-level resistance to polyenes (these are becoming increasingly and alarmingly prevalent), especially C. auris and C. glabrata, in comparison with AmB, to better define their potential in overcoming clinically relevant resistance.

The study reports that C35-sugar addition and C16-amidation (for example, in Nys31–Nys34) improve antifungal potency while significantly reducing red blood cell hemolysis. While the hemolysis results seem experimentally sound and relevant as a measure of membrane disruption activity as well as membrane selectivity, hemolytic activity alone does not provide a

proper assessment of polyene toxicity, which is associated with nephrotoxicity. The study does not include toxicity data from kidney epithelial models or in vivo systems, which are more predictive of therapeutic safety for this compound class. Therefore, although reduced hemolysis is encouraging, additional experiments are necessary to establish a robust toxicological profile.

Referee #3

(Remarks to the Author)
Here, the author reports the discovery of glycosyltransferases that can catalyze the addition of a second sugar at the C35 position of polyene natural products, enabling the generation of various polyene derivatives. This discovery offers a valuable tool for research into polyene sugar modifications. However, the research lacks a systematic examination of the underlying mechanisms and bioactivity.
Firstly, the identification of glycosyltransferases capable of adding a second sugar to the polyene structure has not been accompanied by a thorough enzymatic study. Key questions remain unanswered: What is the detailed catalytic mechanism? Is it possible to obtain the crystal structure? Why does this type of enzyme specifically catalyze the C35 modification? What is the substrate scope?
Secondly, in the bioactivity assessment, the author compares the modified polyene natural products with Amphotericin B (AmpB). However, the evaluation of activity is overly simplistic, relying solely on in vitro antifungal assays. A more comprehensive approach is necessary, incorporating various assays such as ergosterol binding, in vitro antifungal testing, and in vivo antifungal evaluation to draw valid conclusions.
Lastly, the author should provide an explanation for why modified polyenes exhibit increased bioactivity and decreased toxicity. Is there a general principle that governs this phenomenon?
Consequently, I believe the results and novelty of this work do not meet the publication standards of Nature.

Version 1:

Reviewer comments:

Referee #1

(Remarks to the Author)
The manuscript continues to tackle the important area of antifungal discovery and development, an important and understudied area. In my view, the big accomplishment is an antifungal (Nys34) that is superior to AmB and accessible by enzymatic modification. We should be putting antifungal discovery front & center in journals like Nature. However, the paper itself is quite long and in some parts a bit hard to follow with topics including partial enzyme characterization, natural product isolation and pathway prediction, and gene sub-cluster descriptions loosely linked to what seems to be the key accomplishment (i.e. new antifungals). I elaborate on these issues below.

(1) The current title is highly generic and the claim is unclear. Would the authors select a clear, succinct title that details their claim in specific terms? A visual abstract would also be useful.

(2) The structure of NysA34 (final lead compound) should be highlighted specifically in the text, and compared side-by-side with AmB (the comparison compound). The structure is rather hidden with how substituents are abbreviated, which also occludes the fact that the amidotransferase was not used in making this molecule (it was a chemical synthesis step).

(3) Enzymes - The gene selSV and its “subcluster” were discovered/described in 2016 by other groups. I understand the gene was not characterized at that time and therefore function was unknown. However, I am still not very convinced of the novelty of these enzymes esp. with a lack of typical characterization methods like kinetic rate constants or X-ray crystal structures. In Response to Reviewer 3, the authors provide the type of information which begins to contextualize the enzymes. For example, I learned from their response that these are Class I GTases related to YjiC, which has been structurally characterized; this information should also be put into the paper. I’m assuming these are inverting GTs with an oxocarbenium intermediate; is that correct? Given that, what is the role of the His as a general base and how does the (AlphaFold) structure support the mechanism? – add a scheme to SI Fig. 51. Similarly, conclusions regarding promiscuity seem disconnected from the work of others in the field; are GTases typically promiscuous, and how novel is this result?

(4) Another aspect that I am still curious about is the novelty/function of the subclusters. The new heterologous expression data is great. However, the authors state in the paper that they predicted that these are (sub)gene clusters for TDP-L-digitoxose, but don’t say why. I believe this is because the biosynthetic pathway to TDP-L-digitoxose in bacteria was already known in enough detail to predict it here. If so, they are missing citations of those papers. See more below in my comment regarding SI Figs 2/3.

(5) The natural products isolation work (driven by genome mining) is interesting, and it’s great to see new molecules; however, the authors could better use the figures to help the reader follow the logic of the story. I find myself getting lost in the many molecules and strains under study. Furthermore, in a general journal like Nature, the authors should make clear that the biosynthetic predictions from AntiSMASH are not experimentally validated (i.e. Ext. Data Fig. 6 should clarify it is a prediction based on bioinformatics and that the authors do not know yet that the dashed genes are inactive).

(6) The sentence in the Results, “Sequencing of the selvamicin biosynthetic gene cluster …”, references a paper by a

different lab that reports two such BGCs. The placement of this sentence in Results seem to imply this work is part of the work performed by the authors when it was in fact already performed by other labs a decade ago. These details should be moved to the Introduction and the work more clearly attributed to the right people.

(7) To clarify, the amidotransferase is not used to produce NysA34, correct? If correct, I believe this fact should be better highlighted by the authors. It seems this enzyme may be useful for making other antifungals, but not for the lead compound? They should clarify why they include the amidotransferase in an already long, dense paper.

Minor comments

Accession numbers are missing throughout the manuscript.

Fig. 2
• Panel a shows % glycosylation with TDP-D-glucose, which is quite an unusual metric compared to what would normally be reported. Is this a typical metric for glycosyltransferases for some reason? How does this value compare to other GTases that use TDP-D-glucose?
• I'm also confused why the assays is with TDP-D-glucose when the informatics suggests the co-substrate is TDP-L-digitoxose.
• Conditions for reactions assayed are not described; what "control" means is unclear.

Fig. 3
• Missing accession number details; inconsistent and in some cases incomplete labelling of species names.

Fig. 4
• Chemical standard control traces are missing.
• Panels b and c appear to be the high-res mass versions of panel a (and are unlikely needed in the main panel of a Nature-level journal).
• Panel e is labelled "IC50" in a column that lists the names of strains; I think the label is meant for other columns.
• Standard error values appear to have been missed.
• Panel f appears to be missing the units.
• Panel g is an AlphaFold model; how reliable is this model?

Suppl. Fig. 3
• This phylogenetic tree is odd, listing some branches with an enzyme name and others showing an unlabelled gene cluster. I'm not sure any other readers aside from the authors will be able to make use of these data, since it's unclear what is being shown, i.e. missing any labels.
• I don't totally follow the workflow in this Suppl. Fig., however, I do note that the "sub-clusters" from the three strains are clearly distinct in terms of the number of genes they contain. Are they all matching what is shown in Suppl. Fig. 2? Annotation is provided in SI Fig. 2 for nys but without elaboration. I therefore suggest the authors provide some context for the non-expert reader on whether their "discovery" of these sub-clusters remains novel, add citation of relevant work by others that allowed them to make their predictions for TDP-L-digitoxose biosynthesis (i.e. what does each encoded enzyme do in this pathway?), and add some clear elaboration of how the sub-clusters are distinct from one another, despite all producing TDP-L-digitoxose.

Referee #2

(Remarks to the Author)
The revised manuscript includes now the LC HRMS and NMR data with the corresponding spectra and chromatograms in the Supplementary Information. These crucial additions bring the compound characterization up to the required level of chemical characterization standards. The readers can now evaluate peak assignments, mass accuracy, and confirm the reported chemical structures as well as reproduce the experiments for any future studies in the field. I do, however, think that the organization of the added data is not always clear. Some spectra are not linked to specific compounds in the main text, and the numbering of Supplementary figures too dense and hence, confusing. It can be difficult to locate the exact dataset without having to go through multiple captions. Cross reading between the main text and Supplementary figures is still inefficient.

Crucial reverse glycosylation assays, heterologous expression experiments, and NMR characterization of products have been added. These now provide convincing confirmation of the assigned glycosyltransferases and confirm the structures of the modified polyenes. The authors initially carried out assignments based on biosynthetic logic, which are very important and often strongly support the data. However, the essential evidence that has now been added provide direct experimental demonstration of enzyme activity and help confirm the product polyene structures. Adding RT-qPCR data also supports the biological interpretation by pointing out differences in production between strains. At the same time, the integration of these datasets into the main text is not clear and easy to understand and integrate. The linkage between specific figures and claims in the main text is sometime hard and challenging to follow and can be simplified and improved.

More enzymatic assays with addition polyene substrates such as natamycin have been included. These valuable experiments show that the enzymatic platform is not restricted to a single polyene scaffold and can act on structurally diverse polyenes. In my opinion, this dramatically improves the scope of the work and now confirms the claim of broader applicability. I do however think that these very important results are mainly reported in the revised Supplementary

Information and are not emphasized enough in the main text given their importance.

Cytotoxicity assays at the cell-based level in HEK293, A549, and HEPG2 cell lines have been added in the revised study. These experiments expand the evaluation of toxicity beyond hemolysis. This information is now closer to accepted preclinical standards and further supports the claim that the modified compounds may have improved safety profiles. The data indicates reduced toxicity compared to the parent compound, which is an important result. Key quantitative values such as IC50 are not clearly presented to allow quick comparison across compounds.

Mouse tolerability studies, pharmacokinetic analysis, and an in vivo infection model have also been added. These experiments fundamentally improve this study by clearly demonstrating that the modified polyenes are not only active in vitro but also tolerated and effective a rodent in vivo model. The pharmacokinetic analysis, including model fitting and diagnostic plots, now provides a basis for dosing. The infection model showing reduction in fungal burden adds important translational relevance and clearly demonstrates clinical development potential that may rise from this study.

Pharmacokinetic analysis is presented mainly in the Supplementary Information file with limited explanation in the main text, which make interpretations inaccessible to a general multidisciplinary readership such as that of Nature. Some PK figures also do not clearly indicate sample size or variability directly on the plots.

Experiments addressing sterol interactions, including ergosterol-related analyses, have been added in the revised manuscript as well. This data contributes to a mechanistic explanation for reduced toxicity by linking structural modifications to changes in sterol affinity. This supports the interpretation of improved safety. However, despite their strength, these results are only briefly discussed in the main text, and their importance is not emphasized.

The extended dose-response experiments and additional replicates that have been included improve the effectiveness of the statistical analysis of the biological conclusions and allows more reliable comparison between the modified polyenes. I do however suggest that a single table that lists key values such as IC50 or EC50 is added in the SI and referred to in the relevant places in the main text.

To conclude, I am highly supportive of the acceptance of this manuscript in Nature, provided that the authors address the key points outlined above. In my view, the work is already within the quality range expected for journals in the Nature family, and with a more focused and simplified presentation, it would be well positioned for a broader readership. The suggested revisions are aimed at improving clarity, accessibility, and impact, and once implemented, will further strengthen what is already a significant and highly appealing contribution.

Referee #3

(Remarks to the Author)
In this work, the authors provide sufficient data to confirm that they identified two types of glycosyltransferases capable of attaching sugar to the unusual C35 position of a polyene, generating a promising candidate, Nys34, which exhibits potent antifungal activity along with low toxicity and high solubility. In the revised manuscript, although the authors did not obtain a crystal structure of the glycosyltransferase to explain the mechanism, it is still acceptable to combine AlphaFold with point mutation analysis to propose a model. Several issues have been raised after reviewing the revised manuscript.

1. I am a little bit confused about the naming of the natural product in this paper, as also noted by reviewer 2. It appears that the two compounds identified in this paper share exactly the same structures as those previously identified as mandimycin and mandimycin B, originally described by Wang et al. (Nature, 2025, 640:743). In the present manuscript, however, these compounds are renamed as mycopeptins C and A, respectively. Although the authors provide a rationale for this nomenclature change—primarily to align with earlier terminology established for this biosynthetic pathway in 1977—I find this renaming neither necessary nor advisable, as it may in fact introduce confusion in the field for the following reasons. First, mandimycin represents a structurally and functionally distinct class of polyene macrolides, characterized by a unique 5+1 polyene motif and a mode of action that differs fundamentally from classical heptaene-containing polyenes. The original designation by Wang et al. appropriately reflects these distinguishing features, and this distinction is further supported by the data presented in the current manuscript. Second, the proposed nomenclature “mycopeptin” is not fully consistent with the historical nomenclature it seeks to reference (e.g., “mycoheptin”) and does not clearly convey the relationship among compounds within the same class. From a nomenclature standpoint, this inconsistency may hinder rather than enhance clarity. More importantly, the suffix “-peptin” is conventionally associated with peptide-derived natural products (e.g., NRPs or RiPPs) and is rarely used for polyketide-type compounds such as polyene macrolides. Its use here is potentially misleading and may create unnecessary ambiguity for researchers working in natural product chemistry and biosynthesis. Therefore, I do not find sufficient justification for renaming these previously reported compounds, which have already been clearly defined and widely recognized in the literature. To maintain consistency and avoid confusion, I strongly recommend that the authors adhere to the nomenclature established in the prior 2025 Nature publication. “Mycopeptin C” and “mycopeptin A” should be restored to mandimycin and mandimycin B, respectively, and the newly identified compound “mycopeptin B” could be more appropriately designated as mandimycin C.

2. The authors provided in vivo antifungal efficacy experiments in the revised manuscript, which is commendable. For in vivo studies of polyene antifungals, the therapeutic route is typically intravenous rather than intraperitoneal. Can the authors explain why they did not administer Nystain 34 through IV? Was it because of toxicity or formulation?

3. I note that Nys34 was generated by ethanolamine modification of Nystatin A3. Actually, in 2022, Tamar et al. (ACS Med.

Chem. Lett., 2022, 13:182-187) performed similar experiments and obtained similar results, showing that C16-modified Nystatin analogues exhibited potent antifungal activity as well as significantly low toxicity and high water solubility. Therefore, does the sugar modification at the C35 position or the amide derivatives modification at the C16 position account for the observed effects in this case?

Version 2:

Reviewer comments:

Referee #1

(Remarks to the Author)
The authors have considered the reviewer comments and updated the manuscript accordingly. I do not have further feedback to provide at this time.

Open Access This Peer Review File is licensed under a Creative Commons Attribution 4.0 International License, which permits use, sharing, adaptation, distribution and reproduction in any medium or format, as long as you give appropriate credit to the original author(s) and the source, provide a link to the Creative Commons license, and indicate if changes were made.
In cases where reviewers are anonymous, credit should be given to 'Anonymous Referee' and the source.
The images or other third party material in this Peer Review File are included in the article's Creative Commons license, unless indicated otherwise in a credit line to the material. If material is not included in the article's Creative Commons license and your intended use is not permitted by statutory regulation or exceeds the permitted use, you will need to obtain permission directly from the copyright holder.
To view a copy of this license, visit https://creativecommons.org/licenses/by/4.0/

Dear Dr Le Bailly,

Our manuscript, 'Discovery and characterization of new pathways to improved polyene antifungal agents' (2025-04-10717), has been extensively revised and now includes substantial additional experimental results. We have carefully addressed all the reviewers' comments, and we believe that incorporating the recommended additional research has significantly strengthened the paper.

We recognize that the inclusion of new data has increased the overall length of the manuscript. However, to avoid confusion, we have not removed any substantial portions of the original (black) text, thereby preserving the main flow of the paper. We felt that deleting sections of the original manuscript to accommodate the additional results and discussion requested by the reviewers might make it more difficult to clearly track the revisions. If the reviewers and editors consider it appropriate, the manuscript could readily be shortened by consolidating the Introduction, Conclusion, and portions of the discussion related to NMR structure determination. All additional experimental data and revisions are highlighted in blue text in both the manuscript and the Supplementary Information.

Our point-by-point responses to the reviewers' comments are provided below, with page, line, and figure numbers referring to the locations of edits in the revised manuscript and Supplementary Information. We have cross-referenced between reviewers' comments where there is overlap. However, where referees have asked similar or related questions, we have reiterated some responses so that each response to the reviewer can be read independently.

Sincerely,

Jason Micklefield
Imperial College London

The Editor (Dr Le Bailly) As part of the revisions, we would encourage testing of one of the compounds *in vivo.*

We have tested one of the most promising polyene variants (Nys34) in a mouse model of invasive aspergillosis, demonstrating *in vivo* efficacy by reducing fungal burden with no signs of toxicity. Our *in vivo* data is very promising, compared with Amphotericin B and Nystatin A1 (current drugs in the clinic), which are reported to show significant toxicity in mice (see 2.4 & 3.2 below).

Referee #1 This manuscript tackles the important area of macrolide antifungal discovery and development, an urgent area given the dearth of new work in this area and challenges with accessing these large, complex molecules, problems that are urgent to address given the rise in fungal infections. The key contribution / discovery is of the new glycosyltransferase that guides discovery of new molecules via genome mining, and these molecules in some cases possesses enhanced bioactivity compared to AmB. The manuscript is very long with variable level of experimental support for some of the claims, and the I think the revision can work to streamline the logic especially for readers outside of this area and potentially go more in-depth on areas where the results are fully understood and leave out results that are less solid.

We are grateful for the reviewer's helpful comments. We have fully addressed them by providing additional experimental and *in silico* data, thereby strengthening areas that the reviewer felt were unclear or less well understood. We are confident that all parts of the paper are robust (solid), and therefore no results have been removed. As stated above, this has increased the length of the manuscript. However, if necessary, it would be straightforward to reduce the length of the text.

[1.1] They state that "no genes present in the BGC, or that have been identified elsewhere on the *S. noursei* genome that might account for the second sugars in NysA3 and Nys1070"; however, the authors also do not expand on why is was so hard for others to find a glycosyltransferase in this genome (they also do not cite this sentence, making it hard to understand who overlooked this previously).

NysA3 and Nys1070 were first isolated in 1979 [ref 32] and 2004 [ref 24] respectively. Although the biosynthetic pathway leading to NysA1 (the main product) in *S. noursei* has been fully elucidated [e.g. ref 25], the biosynthetic origins of the second sugar moieties in NysA3 and Nys1070 remain unreported. Considering the medical importance of this class of natural products, it is reasonable to assume that, if the elucidation of these pathways were straightforward, such findings would have been published by now. We did not discuss the difficulties we encountered finding the GT enzymes, given the space limitations. However, we elaborate on the challenges below and include all the additional experimental data and discussion that this reviewer requests. **To avoid confusion, we have revised the relevant sentence to read: *"no genes present in the BGC, or elsewhere on the S. noursei genome, have been shown to encode enzymes responsible for the incorporation of the second sugars in NysA3 and Nys1070."* (see p3, line 19-21).**

[1.2] I note that accession number provided in Supplementary Figure 2 is an entry that is already annotated as a glycosyl transferase and the authors in the next sentence state that "Analysis of the S. noursei genome sequence revealed a candidates GT …" Given this is the key discovery in the paper, the authors need to provide some further insight into either what was so challenging for others to miss this gene, and explain why they were successful in this quest. (Are there many GTs in this genome, for example, making it hard to link a specific one?)

In this sentence (p3, line 21-22) we are referring to a "*candidate GT, which we named SnNysSV*" that might add a second sugar to produce NysA3 or Nys1070 in *S. noursei*. At this point in the paper, we have not proved this is the GT in question and so it remains a candidate. The challenge identifying the GT (SnNsySV), stems from the fact that there are 77 genes predicted (annotated) to encode GT enzymes in *S. noursei*. Of these, 13 are from the GT1 family which include enzymes that glycosylate natural products including polyketide macrolides [Zhang 2020]. We selected three (ANZ14691.1, ANZ15335.1 & ANZ15356.1) that are most similar to SelSV for further bioinformatics evaluation, including analysing the sequence and predicted functions of the co-located genes (using antiSMASH). From this we prioritised (ANZ14691.1, SnNysSV), as it was associated with a sugar subcluster similar to the SelSV subcluster. At the outset of the project, we did overproduce the three candidate GTs, from *S. noursei*, and all were subjected to *in vitro* assays, only (ANZ14691.1, SnNysSV) showed any glycosylation activity with polyene substrates. Although this did rule out the possibility that other related GT may be involved, we did not feel it was necessary to include this in the paper. **We have provided more extensive information on the bioinformatics workflow in materials and methods (p2, SI) and in an expanded Supplementary Table 1 (p13, SI).**

[1.3] Is this GT unusual in some way …? The sub-title reads "novel polyene glycosyltransferases" yet it is somehow still unclear why these are "novel."

The section following this subtitle (p3, line 4 onwards) goes on to describe three GTs (NysSV, MycS3 & KfuSV) catalysing additions of a range of different sugars to the C35 position of NysA1 and AmB, which has not been described previously (i.e. which is novel). Although the GTs are novel, in the context of the reactions they catalyse, we have removed the word novel from the subtitle. **Additional data on KfuSV's unusually broad substrate scope is discussed in the response 2.1 below, paper p3 (line 32-38), revised Fig. 2c, Extended Data Fig. 1, methods (p3-4, SI), and additional Supplementary Figs. 4-6 (p19-22, SI). We also provide new structural information, mutagenesis experiments and discussion about the GT enzymes' mechanism. See the response 3.1 below, paper p6, line 8-15, methods (p5, SI) and Supplementary Figs. 51 & 52 (p59 & 60, SI).**

[1.4] Similarly, the authors state that they found three GTs through wider BLAST searches, but I'm quite surprised that they only found three. I believe some information is missing – perhaps they meant they only selected three for analysis (among many)? Would the authors see if they might have missed any? If they can do some bioinformatics to look at the distribution and genome neighborhoods analysis and put into the SI, this would give a better sense of the distribution of this gene and the potential for genome-guided discovery of new antifungals.

The reviewer is correct; the wider blast search we refer to (p3, line 24 onwards) did reveal more than three GTs related to SelSV and SnNysSV that may be involved in polyene pathways, and the three most interesting were selected for further studies. We used bioinformatics tools (EFI-GNT, clusterBLAST and antiSMASH) to identify strains possessing related GTs, and also BGCs predicted to encode polyene-like polyketide synthetases. In total, we found more than ten strains harbouring related GTs and PKS predicted to assemble polyenes. From these, we selected the three GT-containing strains discussed in the paper for investigation. We did not report our broader findings, as the paper and SI were already extensive. **To address the reviewer's helpful comments, we discussed more details of the bioinformatics analysis we carried out in the paper (p3, line 24-26), methods (p2-3, SI), and in an additional Supplementary Fig. 3 (p18, SI).** This additional analysis is helpful for the reader and future research, as it points to the possibility that other strains may also produce new and possibly useful polyenes possessing additional sugar moieties.

[1.5] Ref 34/35 - The location of the mini-gene cluster outside of the "main" gene cluster is certainly notable, but there are many such examples in the literature on secondary metabolism, way more than "a few."

We did not find many characterised examples where genes clusters are split across different loci. Nevertheless, we did not intend to suggest ref 34 and 35 were the only other examples. To avoid confusion, **we have the removed the words "a few" and modified the sentence (p4, line 19-21)** to read – *The observation that the NysSV subclusters and nystatin BGC are at separate chromosomal loci is notable given genes for most reported bacterial natural product pathways are clustered together.*

[1.6] Is it possible to provide direct evidence that the mini-gene cluster indeed produces TDP-L-digitoxose? Or elaborate why you think it produces this product for the general reader? Similarly, the authors could directly prove that nysSV is linked to NysA3 production via gene deletion and complementation studies. Furthermore,

if I followed your logic in the sentence "Unclear why NysA3 is the major product in S. albus, …" does transcript analysis provide any insight?

The reviewer makes several good points. We had expressed the KfuSV subcluster in the AmB producer, *S. nodusus*, which produced AmB-L-digitoxose **(see paper p6 line 15-18, Fig. 4a-c, Supplementary Fig. 53 (p61, SI))**. This confirmed that the KfuSV sub-cluster makes and adds L-digitoxose to the C35-position of AmB. The NysSV and KfuSV subclusters are similar **(see Supplementary Fig. 40 (p49, SI),** both sugar subcluster genes share sequence identity of 54-74% which is strong evidence that the NysSV subclusters also make and add L-digitoxose to C35 of NysA1 (which is structurally related to AmB), generating NysA3.

However, to provide more direct evidence linking the NysSV subclusters to the production of NysA3, we first attempted to delete subcluster genes in both *S. albulus* and *S. noursei*, as the reviewer suggests. Unfortunately, we were unable to obtain the knockout strains. We did however succeed in expressing the *Sa*NysSV subcluster in the AmB producer *S. nodusus*, and this strain also provided an AmB derivative possessing C35-L-digitoxose (AmB-L-digitoxose), confirming the *Sa*NsySV subcluster produces and adds L-digitoxose to C35 of the structurally related AmB. **The new data linking NysSV to NysA3 production is discussed in the paper (p6, line 23-25), materials and methods (p7, SI), and in an additional Supplementary Fig. 57 (p66, SI).**

We also succeeded in expressing the *Sa*NysSV subcluster in a heterologous host *S. albus* (not to be mistaken with *S. albulus*). We incubated the lysate of this strain (*S. albus::NysSV-subcluster*) with NysA1 which resulted in production of NysA3, providing **further conclusive evidence that the NysSV subcluster produces L-digitoxose and transfers this sugar to C-35 of NysA1 – see p4, line 15-21 in the paper, materials and methods (p6-7, SI), and new data in a new Supplementary Fig. 16 (p30, SI).**

Also, following the reviewer's guidance, we carried out targeted gene expression (transcript) analysis in the two NysA3 producing strains. Using RT-qPCR, we showed that the NysSV subcluster is expressed to a higher level in *S. albulus*, than in *S. noursei*, which could account for the higher level of NysA3 production we observe in *S. albulus*. **New transcript analysis data is discussed in the paper p4, line 24-28, Extended Data Fig. 3, materials and methods (p7-8, SI), and in an additional Supplementary Fig. 17 (p31, SI).** Taken together the new results are significant, as they link the remote NysSV subcluster to NysA3 production, and suggest *S. albulus* may have evolved to express higher levels of this subcluster, and thus produce more NysA3, than *S. noursei*.

[1.7] The name "mycoheptin A" is confusing, since you already have a "mycoheptin." Based on what I read, I was quite confused by this structure. A full structure is provided, but if I understand correctly, you are assigning a structure based on high-resolution mass analysis, which is inconsistent with standards in the field of natural products and wouldn't enable publication of this structure in specialist venues. (I make this note as an example, please check for other examples where you may have over-assigned structural information based on the available structural data or bioinformatic analysis).

The label A, in mycoheptin A, was an error (typo). There is only one mycoheptin that we have identified in *S. netropsis* (DSM40846). We did not provide NMR data for mycoheptin previously, as it had already been isolated from the same strain and fully characterized by NMR [Ref 38]. To address the reviewer's comments, **we re-isolated mycoheptin and recorded new NMR spectra (discussed on p4, line 45 onwards, with new NMR data reported in Supplementary Figs. 22-28 (p36-39, SI)** and **Supplementary Table 7 (p40, SI),** further confirming that the structure we obtained is identical to mycoheptin reported previously [Ref 37, 38].

We were also able to acquire new NMR data for mycopentin A – see p5, line 1-3, Supplementary Figs. 29-32, (p41-42, SI) and Supplementary Table 8, (p43, SI) – which corresponds structurally to mycoheptin but contains a C30–C31 single bond (i.e., it is a pentaene). Furthermore, we isolated **another congener, mycopentin C, which we characterised by NMR – see p5, line 8-10, Supplementary Figs. 33-38 (p44-46, SI) and Supplementary Table 9 (p47, SI)**. Mycopentin C possesses two additional L-digitoxose residues at C35 and shares the same structure as mandimycin, which was recently isolated from a different strain (DSM40259) [Ref 40]. Based on these new observations, **we revised our previously proposed biosynthetic pathway for the mycopentins (see revised text, p5, line 11-16, Fig. 3d, and updated Extended Data Fig. 5).** We have retained the names mycoheptin and mycopentin to be consistent with the nomenclature first introduced for this polyene pathway in 1977 [Ref 36, 37, 38], which pre-dates the 2025 characterization of mandimycin [Ref 40]. In the *S. netropsis* (DSM40846) strain we explored, the mycopentins (including mandimycin) are all congeners of mycoheptin, which does not appear to be the case in the DSM40259 strain according to the 2025 study [40].

To further investigate this pathway, we incubated MycS3 with TDP-L-digitoxose and mycopentin A, which resulted in the addition of a single L-digitoxose residue to mycopentin A, to give mycopentin B. Analysis of *S. netropsis* (DSM40846) extracts also revealed trace quantities of mycopentin B, the amount of which can be increased upon incubation with MycS3 and TDP-L-digitoxose. Further studies—beyond the scope of the

present work—will therefore be needed to determine how the second L-digitoxose is incorporated and to clarify the timing of L-digitoxose methylation (most likely catalysed by MycS4) to produce mycopentin C. **Additional discussion of the MycS3 assays and mycopentin pathway can be found in the paper p5, line 11-16, Fig. 3d, updated Extended Data Fig. 5, methods (p4-5, SI), and in Supplementary Fig. 39 (p48, SI).** We believe this is a significant addition as it links the family of GTs we have characterised to the biosynthesis of other potentially useful polyenes.

Minor comments

[1.8] A number of chromatograms lack information about how peaks were assigned (as chemical standard traces are not shown), what type of analysis is presented (EIC or TIC?) (e.g. Fig 2b).

We have provided information on the assignment and the type of analysis we used for all the chromatograms presented in the paper and SI.

[1.9] "the subcluster containing MycS3" ◊ "the subcluster encoding MycS3 …"?

We have changed this as requested.

Referee #2 (Remarks to the Author):

I find this manuscript **highly interesting and a significant, timely contribution** to antifungal drug development, particularly considering the growing global resistance to current therapies and the toxicity limitations of widely used polyenes such as amphotericin B (AmB) and nystatin A1 (NysA1). These broad-spectrum antifungals are considered last-resort treatments; notably, AmB is the only polyene used for systemic fungal infections. However, its significant nephrotoxicity remains a major clinical concern. Given these challenges, the subject matter is **both timely and highly significant, warranting publication in Nature** provided the reviewers' comments are adequately addressed.

The study **delivers substantial advances** by identifying and characterizing novel biosynthetic enzymes, specifically glycosyl-transferases and amido-transferases (GTs), that enable modular and efficient derivatization of polyene scaffolds. Through a combination of genome mining, heterologous expression, in vitro enzymology, and fermentation-based production (which is the most advantageous strategy for active pharmaceutical ingredient production when such a possibility exists), the authors demonstrate that these enzymes can be exploited to introduce both sugar and carboxylate modifications that enhance antifungal efficacy, improve solubility, and significantly reduce mammalian toxicity. These attributes are key to overcoming the limitations of current polyene antifungal agents.

One of the study's strongest features is the discovery of glycosyltransferases such as KfuSV, MycS3, and SnNysSV, which show broad substrate tolerance and can glycosylate clinically relevant polyenes both in vitro and in vivo. Identifying these enzymes not only clarifies the biosynthetic origins of rare polyenes like NysA3 and selvamicin but also enables an impressive diversity of enzymatic glycosylation strategies.

The authors show that expressing GT clusters in heterologous hosts can produce glycosylated polyene derivatives, including novel AmB analogues, offering a scalable approach for generating improved antifungal agents. Additionally, the discovery and structural characterization of previously unknown polyenes such as kasufungins and mycopentins expands the known range of antifungal macrolides, adding significant novelty to the manuscript.

The functional re-evaluation of the amido-transferase PcsA, which previous reports had deemed incompatible with larger polyenes, is another significant advancement. Here, the authors not only purify PcsA by inserting a His-tag into a flexible loop but also show it can efficiently catalyze C16-carboxylate derivatization on both NysA1 and NysA3, generating amide, hydroxamate, and acylhydrazine derivatives. I find the ability to replace the carboxyl group through enzymatic methods, rather than via complex synthetic routes, **to offer an important and high useful improvement.**

We are grateful for the reviewer's helpful comments, which we have fully addressed in detail below.

[2.1] It would further enrich the study to address other **underexplored polyene scaffolds** such as filipin and natamycin, both of which represent structurally distinct yet clinically and mechanistically relevant antifungal agents.

We have carried out a more extensive set of reactions with additional polyene scaffolds, including filipin. **We had already explored PcsA reactions with natamycin (see p7 lines 10 & 21-25, Fig. 4d, Supplementary Fig. 58 (p67, SI), methods (p5-6, SI), and Supplementary Table 12 (p83, SI)).** However, we used the name pimaricin (Pim) for this compound. Both names are used in the literature to describe the same compound, which is used to treat fungal infections of the eye. We appreciate this can be confusing, so we have mentioned in the text that pimaricin is also known as natamycin (**p7, lines 10, 16, & 24**). In addition to pimaricin

(natamycin), rimocidin, NysA1, NysA3, and AmB, which we had already tested with PscA, four more diverse polyene scaffolds – Candicidin (used to treat yeast infections), CE-108, mycopentin A, and mycopentin C were all substrates for PscA forming new amide and hydroxamate products; Neither kasufungin B nor mycoheptin were accepted by PscA. **New data on PcsA's diverse polyene scope is discussed on p7, line 18-20, and presented in Supplementary Figs. 62-65 (p71-74, SI).**

Filipin is an interesting suggestion. As it does not possess a carboxylic acid group and therefore could not serve as a substrate for PcsA, we tested it as a potential substrate with the GT enzymes. However, no glycosylated products were obtained, likely because it possesses a substantially different structure compared to the other polyenes in our study. Nevertheless, we have demonstrated a significantly expanded substrate scope of the GTs with diverse polyene scaffolds. In addition to the glycosylation reactions initially carried out with AmB and NysA1, and the reverse glycosylation reactions of NysA3 with NysSV and kasufungin B with KfuSV or SelSV, we show that KfuSV (the most active GT) can also glycosylate mycoheptin, mycopentin A, along with the AmB aglycone (lacking the C19-mycosamine sugar). **Additional data on KfuSV's diverse polyene scope is discussed on p3, line 32-33, and in Supplementary Fig. 4 (p19, SI).**

Furthermore, we have expanded the TDP-sugars scope, from the six originally explored to include 13 additional sugars. From this, we show that KfuSV accepts 11 out of the 19 different TDP-sugar substrates tested. As mentioned above [see 1.7] we also show how MycS3 glycosylates mycopentin A to form mycopentin B with the native TDP-L-digitoxose donor substrate. **New data on the expanded sugar scope of KfuSV is discussed in the main text p3, line 33-38, Fig. 2c, Extended data Fig. 1, methods (p3-4, SI), and Supplementary Figs. 5 & 6 (p20-22, SI).** The new data further reinforces our conclusions that the new GTs, particularly KfuSV, are highly versatile enzymes, with very broad polyene and sugar substrate scope. Our results indicate that the GTs could accept many more polyene-sugar combinations in addition to the large number of examples we describe here, which is significant given the improved biological activities of the polyene derivatives (see below).

[2.2] The study demonstrates that sugar-modified AmB-L-digitoxose and NysA3 analogues exhibit enhanced antifungal activity relative to their parent polyenes across a panel of clinically relevant and WHO-priority fungal pathogens, including C. albicans, C. auris, C. neoformans, and resistant strains such as A. fumigatus TR34/L98H. Of note, the specific pathogen identities and assay details are found in the supplementary The specific strain information is provided in the supplementary material but is not clearly presented alongside Figure 5, which limits the immediate interpretability and impact of the main data.

We have now provided the full names of all strains used in Figs. 4 and 5.

[2.3] Another simple yet important observation is the apparent ability of the modified compounds to combat or reduce fungal tolerance, an effect that becomes more pronounced after 48 hours of incubation. Moreover, it would be of interest to test these compounds directly against strains with known high-level resistance to polyenes (these are becoming increasingly and alarmingly prevalent), especially C. auris and C. glabrata, in comparison with AmB, to better define their potential in overcoming clinically relevant resistance.

We have obtained IC_{50}s for AmB, NysA1, NysA3 and Nys31-34 (7 compounds) vs the 10 fungal pathogens which WHO list has possessing the greatest risk to human health. **We have included IC_{50} data for the polyenes against *Aspergillus fumigatus cyp51A*$^{TR34/L98H}$ isolate and *Candida albicans* ATCC 200955 which are both drug resistant. This is now mentioned in the revised paper on p6 line 32, including an additional reference 44, and p7 line 40, methods (p9, SI), and Supplementary Fig, 101 (p101, SI).** In general, resistance to polyenes is less common than resistance observed with most of the other classes of antifungal drugs (such as azoles). Although strains, such as *C. auris,* with increased polyene resistance have been reported, it is difficult to obtain these strains. We have tried to obtain such polyene resistant-clinical isolates from the medical community, but they were unwilling to release those strains to us, which we find disappointing.

[2.4] The study reports that C35-sugar addition and C16-amidation (for example, in Nys31–Nys34) improve antifungal potency while significantly reducing red blood cell hemolysis. While the hemolysis results seem experimentally sound and relevant as a measure of membrane disruption activity as well as membrane selectivity, hemolytic activity alone does not provide a proper assessment of polyene toxicity, which is associated with nephrotoxicity. The study does not include toxicity data from kidney epithelial models or in vivo systems, which are more predictive of therapeutic safety for this compound class. Therefore, although reduced hemolysis is encouraging, additional experiments are necessary to establish a robust toxicological profile.

As suggested by the referee, we have carried out more detailed toxicity tests comparing AmB and Nys34, one of the most promising new polyene derivatives, with HEK293 (human embryonic kidney), A549 (human alveolar basal epithelial), and HEPG2 (human hepatocellular carcinoma) cells. These cell lines are particularly relevant given polyenes typically exhibit high renal (kidney) toxicity, and our subsequent *in vivo* studies focus on lung infections. **The new *in vitro* toxicity data shows that Nys34 exhibits between 3- and 8-fold lower toxicity than AmB in the cell lines tested, which we present in the main text (p7, line 44-46), and in an**

updated Fig. 5 (panels e-g), with further experimental details provided in Supplementary Information (p9-10, SI).

We have also carried out extensive *in vivo* studies with one of the more promising nystatin derivatives (Nys34) in mice. These studies indicate the Nys34 is well tolerated by mice following repeated intraperitoneal injections of multiple doses (5 mg/kg). We observed the distribution of Nys34 across blood and all major organs and carried out pharmacokinetics (PK). Based on PK data a dosing regimen for a mouse *A. fumigatus* lung infection model was established, which is relevant for the treatment of the most common form of pulmonary aspergillosis that contribute to high mortality rates in humans. Nys34 proved to be very effective at treating *A. fumigatus* infections in the mouse lungs, achieving statistical significance from the vehicle and leading to an 87% reduction in the *A. fumigatus* levels observed in the lungs. Treatment with amphotericin B, following a dosing regimen reported previously for mice (Ref 49), did reduce tissue burden but this change did not reach statistical significance. An additional paper is cited (Ref 48) which described how NysA1 is highly toxic when tested in mice. Overall, our mouse studies indicate that Nys34 is more effective *in vivo* than either the parent polyene NysA1 and AmB, which are currently used in the clinic. **The new animal efficacy studies are presented at the end of the paper (p7, line 46 onwards, and Fig. 5h), with experimental details (p10-11, SI) and further details in Supplementary Fig. 103 (p103, SI).** Taken together, the additional toxicity data and animal studies indicate that Nys34, and potentially other polyene variants described here, do have enhanced properties and have significant potential for further clinical development.

Referee #3 (Remarks to the Author):

Here, the author reports the discovery of glycosyltransferases that can catalyze the addition of a second sugar at the C35 position of polyene natural products, enabling the generation of various polyene derivatives. This discovery offers a valuable tool for research into polyene sugar modifications. However, the research lacks a systematic examination of the underlying mechanisms and bioactivity.

[3.1] Firstly, the identification of glycosyltransferases capable of adding a second sugar to the polyene structure has not been accompanied by a thorough enzymatic study. Key questions remain unanswered: What is the detailed catalytic mechanism? Is it possible to obtain the crystal structure? Why does this type of enzyme specifically catalyze the C35 modification? What is the substrate scope?

We are grateful to the reviewer for their helpful suggestions. The GT enzymes (NysSV, MycS3 and KfuSV) are all members of the GT class 1 family of enzymes, predicted to possess a GT-B fold, and MycS3 and KfuSV contain the conserved E/DQ motif. Some aspects of the mechanism of other members of GT-B enzymes have been described previously, including an X-ray structure of a related GT YjiC [Dai 2021]. In addition to this, we have carried out further experiments to probe the structure and mechanism. Firstly, we have carried out extensive crystallography trails with KfuSV and NysSV. However, despite using a robotics platform to screen many different conditions, in Imperial's well equipped structural biology facility, we have yet to generate crystals required for structure determination. Although some proteins can be straightforward to crystalise, KfuSV and NysSV are not. Considering this, we have used AlphaFold to generate models of KfuSV. Further *in silico* substrate docking was then used to predict the architecture of active site, establish possible catalytic residues, and residues involved substrate recognition. Based on these studies, several residues were targeted for mutagenesis and the activity of those mutants were used to provide further insight into the enzyme mechanism. **New structural and mutagenesis data are described in the paper (p6, line 8-15), with additional experimental details (p5, SI) and results in the Supplementary Figs. 51 & 52, (p59 & 60, SI).** From our KfuSV model with polyene docked, we observe an active-site residue (H13), which is predicted to be positioned closed to the C35-OH. When we mutate this residue to Ala, 95% of the activity is lost. We suggest that His13 may thus H-bond to the C35-OH, and may function as a general base and/or contribute to position the substrate close to the sugar-TDP co-substrate. Additional active site residues (H294, T299 and D318) may also contribute to catalysis and/or substrate binding, given that mutation to alanine results in substantial or even total loss of activity. Interestingly, KfuSV T297A shows improved activity with TDP-glucose and non-native polyene substrates. We suggest that this mutation increases the size of TDP-sugar binding pocket, which enables better accommodation of non-native substrates.

We have added extensive additional data on the GT substrate scope. As mentioned above [2.1], initially we tested the scope of the GTs with AmB and NysA1 (Fig 2), and the reverse glycosylation reactions of NysSV with NysA3, and KfuSV or SelSV with kasufungin B (Fig 4). **Further examples showing expanded polyene (acceptor) substrate scope including glycosylation reactions with the most active GT in the family KfuSV and mycoheptin, mycopentin A, along with the AmB aglycone (lacking the C19-mycosamine sugar) have been added to the paper (see p3, line 32-33, and Supplementary Fig. 4 (p19, SI)). We also include further *in vitro* data on MycS3 showing how this GT transfers a single L-digitoxose sugar to mycopentin A – see revised text, p5, line 11-16, Fig. 3d, and Supplementary Fig. 39 (p48, SI).** Furthermore, we have expanded the sugar substrate scope significantly, from the six TDP-sugars originally

explored to include 13 additional TDP-sugars. This shows that KfuSV accepts 11 out of the 19 different TDP-sugar substrates tested. We also note that whilst KfuSV accept sugar substrates with a C2-OH group but not those with a C2-F substituent. We rationalise that this is likely due the higher electronegativity of the C2-F substituent which can destabilise the oxocarbenium ion that is proposed to be formed during glycosylation. This observation is in line with other GT-B enzyme studies and provides further evidence into the mechanism of the GTs. **New data on the expanded sugar scope of KfuSV is discussed in the main text p3, line 33-37, Fig. 2c, and methods (p3, SI), with further data in Supplementary Figs. 5 & 6 (p20-22, SI).**

The extensive additional experiments we have carried out to probe the structure-activity relationship and substrate scope of KfuSV further strengthens our conclusion that the newly identified GTs (especially KfuSV) are highly versatile enzymes with broad polyene and sugar substrate ranges. Our results suggest that these GTs can likely accept many more polyene–sugar combinations beyond the extensive set we report here, and are amenable to further structure-guided engineering, which is significant given the improved *in vitro* and *in vivo* bioactivity data we observe (see below).

[3.2] Secondly, in the bioactivity assessment, the author compares the modified polyene natural products with Amphotericin B (AmpB). However, the evaluation of activity is overly simplistic, relying solely on in vitro antifungal assays. A more comprehensive approach is necessary, incorporating various assays such as ergosterol binding, in vitro antifungal testing, and in vivo antifungal evaluation to draw valid conclusions.

We have carried out extensive new *in vitro* and *in vivo* antifungal evaluation and toxicity testing as also discussed above [2.3 & 2.4]. More detailed toxicity tests for AmB and Nys34 one of the most promising new polyene derivatives were carried out with HEK293 (human embryonic kidney), A549 (human alveolar basal epithelial) and HEPG2 (human liver) cells. These cell lines are particularly relevant given polyenes typically exhibit high renal (kidney) toxicity, and our subsequent *in vivo* studies focuses on lung infections. **The new *in vitro* toxicity data shows that Nys34 exhibits 3- to 8- fold lower toxicity than AmB, in the three cell lines, which we present in the main text (p7, line 44-46), and in Fig. 5 (panels e-g), with further experimental details provided in Supplementary Information (methods, p9-10).**

As suggested by both reviewers (2 & 3) we have conducted extensive *in vivo* studies with one of the more promising nystatin derivatives (Nys34) in mice. These studies indicate the Nys34 is well tolerated by mice following repeated intraperitoneal injections of multiple doses (5 mg/kg). We observed the distribution of Nys34 across blood and all major organs and carried out pharmacokinetics (PK). Based on PK data a dosing regimen for a mouse *A. fumigatus* lung infection model was established, which is relevant for the treatment of the most common form of pulmonary aspergillosis that contribute to high mortality rates in humans. Nys34 proved to be very effective at treating *A. fumigatus* infections in the mouse lungs achieving statistical significance from the vehicle and leading to an 87% reduction in the *A.fumigatus* levels observed in the lungs. Treatment with amphotericin B, following a dosing regimen reported previously for mice (Ref 49), did reduce tissue burden but this change did not reach statistical significance. An additional paper is cited (Ref 48) which describes how NysA1 is highly toxic when tested in mice. Overall, our mouse studies indicate that Nys34 is more effective *in vivo* than either the parent polyene NysA1 and AmB, which are currently used in the clinic. **The new animal efficacy studies are presented at the end of the paper (p7, line 46 onwards, and Fig. 5h), with experimental details (p10-11, SI) and further details in Supplementary Fig. 103 (p103, SI).** Taken together, the additional toxicity data and animal studies indicate that Nys34, and potentially other polyene variants described here, do have enhanced properties and have significant potential for further clinical development.

Finally, as suggested by this reviewer, we have explored ergosterol binding. Previously UV-vis studies have been used to explore the binding of AmB to sterols [e.g. Ref. 6]. Following this approach, we compared the binding of ergosterol with Nys34, which we used in our *in vivo* experiments, and the parent polyenes (NysA1 & AmB). Our UV-vis titration experiments show increasing concentrations of ergosterol lead to significant hypochromic and red shifts with NysA1 and AmB consistent with complexation, which agrees with other studies suggesting NysA1 and AmB share a similar sterol-sponge mechanism of action [ref. 6 & 19]. In contrast, UV experiments with Nys34 show no evidence of ergosterol binding. This suggests the Nys variants possessing an additional C35-sugar and amide modification is likely to exhibit an alternative mechanism of action. **The UV-vis experiments are described in the paper (p8, line 18-19, and Fig. 5i-k), with experimental details (p12, SI) and further details in the Supplementary Table 5 (p15, SI).** In addition, we also explored the effect of polyene-ergosterol precomplexation on antifungal activity with the yeast *Saccharomyces cerevisiae*. Precomplexation of NysA1 and AmB (1:5 polyene:erg ratio) resulted in a significant, 3.4- and 11.8-fold reduction in IC_{50} respectively, which aligns with previous experiments, which were used to support the sterol-sponge hypothesis [ref. 19]. In contrast, precomplexation of Nys34 and ergosterol (1:5), had no effect on antifungal activity and further points to the new Nys34 possessing a different mechanism of action to AmB and NysA1. **The effects of ergosterol precomplexation on antifungal activity is described in the paper (p8, line 19-24, and Fig. 5l-n), and a new Supplementary Fig. 104 (p104, SI) with experimental details in the Supplementary Information (p10, SI).**

[3.3] Lastly, the author should provide an explanation for why modified polyenes exhibit increased bioactivity and decreased toxicity. Is there a general principle that governs this phenomenon?

Our UV-vis titration and precomplexation experiments (3.2 above) indicate that Nys34 does not bind to ergosterol and is thus likely to interact with other components of fungal cell membranes, which may account for the increased bioactivity and reduced toxicity we observe. Previous studies suggest that the fungicidal action of AmB, NysA1, and other polyenes is largely attributed to their propensity to extract ergosterol from the membrane, forming an extramembranous sponge (aggregate). Our observation that Nys34 retains high potency and *in vivo* efficacy despite not binding ergosterol indicates that sponge-formation is not required for fungicidal activity, in this case. Although this is a notable finding, more extensive biophysical and cell-based mechanism of action studies would be required to define the principles that may govern the changes in bioactivity and toxicity that we observe with Nys34 and related polyene variants in our paper. Such detailed studies would be beyond the scope and time scale of our paper, which is already very extensive and cuts across multiple scientific fields. We note that studies into the mechanism of action of AmB have been reported in many publications over the last 50 years, with alternative mechanisms proposed; from research on transmembrane pore formation in the 1970s (e.g. reviewed by Bollard 1986), to sterol sponge formation described more recently (e.g. ref. 4, 6 & 19). We do not believe it is feasible for us to carry out the large amount of experimentation that these earlier studies indicate would be necessary to decipher the alternative mechanism by which the nystatin variants we have generated exert their potent fungicidal action.

[3.4] Consequently, I believe the results and novelty of this work do not meet the publication standards of Nature.

We are grateful to the reviewer for their opinions and suggestions. We believe we have addressed all their concerns with a significant set of new experiments that they requested, including extensive GT enzymology, structure, mutagenesis, and mechanistic studies. We have provided a very large data set on the substrate scope of the GT enzymes. Also, as requested, we have provided conclusive experiments on ergosterol binding, further *in vitro* antifungal testing, and *in vivo* antifungal evaluation in mice.

Referee #1 The manuscript continues to **tackle the important area of antifungal discovery and development**, an important and understudied area. In my view, **the big accomplishment is an antifungal (Nys34) that is superior to AmB** and accessible by enzymatic modification. We should be putting antifungal discovery front & center in journals like Nature. However, the paper itself is quite long and in some parts a bit hard to follow with topics including partial enzyme characterization, natural product isolation and pathway prediction, and gene sub-cluster descriptions loosely linked to what seems to be the key accomplishment (i.e. new antifungals). I elaborate on these issues below.

We are grateful for the referee's very rigorous review. The referee has made a lot of helpful comments, on issues of fine details and clarity. Whilst extensive, when considered one-by-one, their comments do not detract from the quality, significance or novelty of our work. We have fully addressed all their points (as we did with their first set of extensive questions that were on other aspects of the work), and completed all the revisions they suggest which further improves the paper.

We agree that the discovery of new and improved antifungals is an important advance, and we have further emphasised this big accomplishment in the paper (**see revised conclusion p8**). The new natural products, biosynthetic gene clusters, and enzymes we have discovered and characterised here are also important, as they provide the template, pathways and biocatalysts from which we can diversify, optimise, and modulate activities of polyenes. We would not have discovered improved polyene antifungals without characterising the enzyme, pathways, clusters etc. **We have moved some of the more technical detail (e.g. discussion on NMR characterisation and bioinformatics) from the paper to SI and explained more clearly the thread and scope of the paper. The conclusion has been rewritten so that the main accomplishments and novelty of the work are more clearly emphasised.**

[1.1] The current title is highly generic and the claim is unclear. Would the authors select a clear, succinct title that details their claim in specific terms? A visual abstract would also be useful.

We have changed the title to be more specific and clearer – Enzymatic glycosylation and amidation reshapes polyene bioactivity. We are very happy to provide a graphical abstract, but the guidelines for the journal ask for a text abstract of no more than 200 words (no visuals/images).

[1.2] The structure of NysA34 (final lead compound) should be highlighted specifically in the text, and compared side-by-side with AmB (the comparison compound). The structure is rather hidden with how substituents are abbreviated, which also occludes the fact that the amidotransferase was not used in making this molecule (it was a chemical synthesis step).

We stated very clearly in the paper from the outset that Nys34 was produced by enzymatic glycosylation and chemical addition of serinol see (1.7 below). We included the structure of Nys34 in Fig. 4, along with structures of 15 other polyene molecules that we produced. Full structures for all the compounds would not fit on one page, so we drew three substructures in a conventional way (i.e. AmB, Nys and Pim scaffolds) highlighting the different groups (R & Y) introduced. Nevertheless, **as the reviewer suggests, we have drawn out in full the structure of Nys34 and placed it alongside AmB, in Fig. 5c (p22).**

[1.3] Enzymes - The gene selSV and its "subcluster" were discovered/described in 2016 by other groups. I understand the gene was not characterized at that time and therefore function was unknown. However, I am still not very convinced of the novelty of these enzymes esp. with a lack of typical characterization methods like kinetic rate constants or X-ray crystal structures. In Response to Reviewer 3, the authors provide the type of information which begins to contextualize the enzymes. For example, I learned from their response that these are Class I GTases related to YjiC, which has been structurally characterized; this information should also be put into the paper. I'm assuming these are inverting GTs with an oxocarbenium intermediate; is that correct? Given that, what is the role of the His as a general base and how does the (AlphaFold) structure support the mechanism? – add a scheme to SI Fig. 51. Similarly, conclusions regarding promiscuity seem disconnected from the work of others in the field; are GTases typically promiscuous, and how novel is this result?

We discussed the selvamicin work (Van Arnam 2016, ref 23) in the introduction, in Fig 1, and extensively at the beginning of the results section (p3, line 5-9), citing the paper five times in the paper and eight times in SI. We were very clear how we used this work as our starting point. **We have added a further statement to the introduction (p2, line 19-22 stating that selvamicin BGC was sequenced (in ref 23 not by us) and that none of the enzymes in this pathway were characterised**. The fact that none of the enzymes were characterised (including SelSV), and that selvamicin has very little antifungal activity, means this paper, whilst important, does not detract from the novelty of our work.

None of the referees asked us to provide additional kinetics data for GT enzymes in their first reviews. Had we been asked, we would have done this, although it is not straightforward for the native TDP-sugar substrate (see 1.8 below). Many other additional experiments were suggested by the reviewers. **We successfully delivered all the additional experiments requested except for the X-ray crystal structures of the GT(s) that referee #3 requested**. As we pointed out, we put significant efforts into crystallising the GTs, but we could not get structures. In the absence of an X-ray structure, we reported an AlphaFold structure along with mutagenesis studies providing additional structural and mechanistic insights (referee #3 agreed this was acceptable). **As requested, we have mentioned in the paper (p5, line 22-23) that the AlphaFold structure shows similarity to the reported structure of YjiC (now cited in ref 42)**. The GT enzymes we described are all inverting GTs, which is evident from the stereochemistry of the products which we determined by extensive NMR experiments. Inverting GTs are suggested to proceed by an S_N2-like mechanism *via* an oxocarbenium-like transition state. The AlphaFold structure shows His13 is 2.6 Å from the polyene C35-OH, and mutagenesis of His13Ala results in 95% loss of activity, which is consistent with His13 functioning as a general base. **We have added the additional discussion on these points and included a scheme to Supplementary Fig. 20 (p54 of SI) as requested.**

We mentioned in the paper that the GT KfuSV is a flexible enzyme for polyene diversification, but **we did not provide any conclusions/claims about our GTs' promiscuity relative to other GTs, at any point**. However, we did show that KfuSV can glycosylate five out of five different polyene scaffolds, and also accepts 12 out of 20 sugar substrates tested so far (including reverse glycosylation with the native substrate), indicating that it is significantly more promiscuous than related GTs. In the most relevant work by others in the field, the GTs that add mycosamine to polyenes (AmphDI & NysDI, **Fig 1**) were tested with a panel of 21 different sugar-GDPs and just one polyene substrate (the AmB aglycone), but only two of the 21 sugars tested were accepted (new ref. 51, Zhang 2008). The authors **conclude that the "stringent sugar nucleotide specificity of the polyene GTs might, in part, restrict the generation of variant [polyene] glycosides."** Given that the mycosamine sugar is essential for antifungal activity, there have been significant efforts to modify/or replace this sugar in polyenes. As we pointed out in the introduction, the majority of work to produce polyene

derivatives, including polyenes with alternative sugars, has relied on challenging multistep synthetic methods, (ref 6 Maji 2023, ref 20 Wilcock 2013, & Croatt 2011) which is likely due to the lack of promiscuity (stringency) exhibited by the other polyene GTs and difficulties producing the sugar-NDPs (also discussed by Zhang 2008 in ref 51).

To address the reviewers' comments, we have introduced discussion on the unusual promiscuity of our GT (KfuSV) and the work on related GTs (see p8, line 17-21 & ref 51), in the revised conclusion. We have also explained that the novelty of the GT enzymes we discovered comes from their capability to selectively add many different second sugars to C35 of therapeutically relevant polyenes, which is not possible by either existing chemical or enzymatic methods. Further emphasising that the addition of a second C35-sugar combined with C16-amide groups has a profound effect on the biological properties of the polyenes.

[1.4] Another aspect that I am still curious about is the novelty/function of the subclusters. **The new heterologous expression data is great.** However, the authors state in the paper that they predicted that these are (sub)gene clusters for TDP-L-digitoxose, but don't say why. I believe this is because the biosynthetic pathway to TDP-L-digitoxose in bacteria was already known in enough detail to predict it here. If so, they are missing citations of those papers. See more below in my comment regarding SI Figs 2/3.

We showed from heterologous expression that KfuSV and NysSV subclusters both produce and add TDP-L-digitoxose to the polyenes, providing very definitive proof of their functions. We indicated in Supplementary Figs 12a and 17a (SI, p25 & 43) that our initial predications on the functions of these subclusters were based on their similarity to the TDP-sugar subcluster in the selvamicin producer (ref 23). However, **we agree that we did not explicitly state this, and so we have added a statement in the captions of supplementary information Figs 12 and 17 (SI p25 & 43) that point to other studies that identified putative TDP-L-digitoxose clusters (ref 23 and new ref 64 Li 2024).** We have also added discussion explaining that whilst TDP-L-digitoxose subclusters have been observed in other biosynthetic gene clusters (i.e. at the genetic level), the enzymes that produce this sugar have not been fully characterised *in vitro,* and some predictions remain to be tested.

[1.5] The natural products isolation work (driven by genome mining) is interesting, and it's great to see new molecules; however, the authors could better use the figures to help the reader follow the logic of the story. I find myself getting lost in the many molecules and strains under study. Furthermore, in a general journal like Nature, the authors should make clear that the biosynthetic predictions from AntiSMASH are not experimentally validated (i.e. Ext. Data Fig. 6 should clarify it is a prediction based on bioinformatics and that the authors do not know yet that the dashed genes are inactive).

We appreciate that we have generated many natural product derivatives, using a range of different enzymes and microbial host strains. We believe this is very important advance for the field and we have endeavoured to explain this as clearly as possible in the revised paper. In addition, **we have provided an inventory of all strains used, in a new Supplementary Table. 1, p6. The NMR data and bioinformatics analysis have also be co-located in consolidated SI figures, which should help the reader navigate the information more easily.**

We have not suggested that we have carried out experiments to validate all steps in all the pathways we have described in the paper. Most of the bioinformatics discussion is now in the SI, and to avoid any confusion**, we have now explained at all points exactly where pathways arise from bioinformatics predications, experiments we did in this paper, or a combination of the two (see revised caption of supplementary Fig 12 & 17 (SI p25-29 & 43-45)**. In the case of Extended Data Fig 6 (now Supplementary Fig 17b) we predict structures of intermediates (functions) of polyketide PKS enzymes. The PKS are very well characterised enzymes, and it is possible to predict with good accuracy their functions based on bioinformatics. It is also possible to accurately predict which PKS domains are redundant (inactive), as we did in extended data Fig 6, by using bioinformatics analysis in combination with structure of the final natural product (kasufungin) which we obtained experimentally by NMR. For instance, a dehydratase (DH) domain of module 11 is present in the PKS (KfuJ) sequence and it must be inactive (or it is skipped) otherwise the C3-OH group in the kasufungin products (clearly observed in our NMR data) would not be present. The DH if active would have eliminated the C3-OH and there would be a C2-C3 double bond. **These more subtle points have also been discussed in the captions to supplementary Fig 12 & 17 (SI p26-29 & 44-46).**

[1.6] The sentence in the Results, "Sequencing of the selvamicin biosynthetic gene cluster …", references a paper by a different lab that reports two such BGCs. The placement of this sentence in Results seem to imply this work is part of the work performed by the authors when it was in fact already performed by other labs a decade ago. These details should be moved to the Introduction and the work more clearly attributed to the right people.

We discussed and cited the selvamicin work (ref 23) extensively (see 1.3) and did not imply that we sequenced the selvamicin BGC, which is why we cited ref 23 at the end of the sentence in question. To avoid any doubt,

we have added a statement to the introduction on p2, line 19-23 indicating that the Sel BGC was sequenced previously by others (ref 23), and we have modified the sentence (on p3, line 7-8) so it is even clearer that this is not our work. In Fig 3 we have also made a note that the Sel BGC is sequenced by others (ref 23), as opposed to myc and kfu BGCs that we sequenced.

[1.7] To clarify, the amidotransferase is not used to produce NysA34, correct? If correct, I believe this fact should be better highlighted by the authors. It seems this enzyme may be useful for making other antifungals, but not for the lead compound? They should clarify why they include the amidotransferase in an already long, dense paper.

We had stated very clearly in all versions of the paper the "carboxylic acid was also chemically modified with serinol to generate a derivative Nys34 possessing the serinol amide…" and at no point do we suggest otherwise (also see 1.2 above). **We have emphasised this further in the revised paper (p6, line 41-44) by stating the serinol amide of Nys34 was introduced chemically (and is inaccessible with PcsA), as opposed to sugar moiety of Nys34 which is introduced enzymatically.**

We focused on Nys34 for the animal studies because we were asked to investigate a single compound rather than multiple candidates. Ethical considerations and time constraints further reinforced the need to concentrate on one compound. We selected Nys34 based on its bioactivity, and because it was easier in the short term to purify the quantities required for additional biological and animal studies. **Several other Nys derivatives we generated (featuring both the C35-sugar and C16-carboxylate modifications introduced enzymatically) showed comparable *in vitro* biological activity and may be equally or more effective *in vivo* against different fungal pathogens. We have discussed this point in the revised conclusion (p8, line 31-33).**

We believe it is important to retain discussion of the amidotransferase (PcsA) modifications as this leads to polyenes with excellent properties, and we show that in addition to Nystatin and AmB, PcsA can be used to derivatise other clinically relevant polyenes (natamycin and candicidin). Referee #2 asked in their first review for us to do more work on the PcsA scope, and test it with more polyene scaffolds, which we did. Also in their second review referee #2 asks that we include more discussion about PcsA in the paper rather than the SI (i.e. rather than removing this from the paper, they are asking for more).

Minor comments:

Accession numbers are missing throughout the manuscript. These have been added throughout (see 1.9 below)

[1.8] Fig. 2 – Panel a shows % glycosylation with TDP-D-glucose, which is quite an unusual metric compared to what would normally be reported. Is this a typical metric for glycosyltransferases for some reason? How does this value compare to other GTases that use TDP-D-glucose? I'm also confused why the assays is with TDP-D-glucose when the informatics suggests the co-substrate is TDP-L-digitoxose. Conditions for reactions assayed are not described; what "control" means is unclear.

We have changed % glycosylation to % conversion. It is common to report % conversions for enzyme reactions. For instance, this is how others have reported the activity of GTs (e.g. AmphDI & NysDI in ref 51. Zhang *et al.*). We mentioned **in the paper "in the absence of the native substrates we tested its [SelSV] *in vitro* activity using TDP-D-glucose." We have extended this sentence in the revised manuscript to make the point more clearly (p3, line 10-11)**. Also, at this initial stage of the study, we are not entirely sure what the native substrates are (for SelSV it could TDP-L-digitoxose or -4-*O*-methyl-L-digitoxose). Further on in the study, **we do provide definitive experimental evidence (not just informatics) that the co-substrate is TDP-L-digitoxose for all GTs (SelSV, KfuSV and both NysSV)**. The NysSV enzymes when incubated with NysA3 (polyene-L-digitoxose) and TDP produced NysA1 (aglycone) and TDP-L-Digitoxose (see p4 line 7-11, p9 line 32-38, Fig. 3f & Supplementary Fig. 9). As explained in the paper, GT reactions are well known to operate as reversible equilibria (see Ref. 35). Therefore, our reverse glycosylation experiments provide evidence for GT function that is equally definitive to testing the same reaction in the forward direction (referee #2 agrees these assays are crucial evidence, see 2.2 below).

Polyene (aglycone) + TDP–L-Digitoxose ⇌ (GT) Polyene–L-Digitoxose + TDP

We also performed the same type of highly definitive reverse glycosylation reactions with KfuSV (see p5 line 15-17, p9 line 32-38, Fig. 3g & Supplementary Fig. 19) and with SelSV (see p5 line 17-21, p9 line 32-38, Fig. 3g and Extended Data Fig. 5). Furthermore, we assayed MycS3 in the forward glycosylation reaction incubating dihydromycoheptin with crude TDP-L-digitoxose (see p4, line 40-44 & Supplementary Fig. 16). Obtaining TDP-L-digitoxose in sufficient quantities and purity for more extensive enzyme assays, in the forward direction, could not provide any more definitive proof of function, and would have required total synthesis of TDP-L-digitoxose which is not trivial. The chemical synthesis of TDP-L-digitoxose would not have added greatly to the paper, given that we show we can produce and add L-digitoxose to polyenes enzymatically, by heterologous expression, which is much cleaner and far more efficient.

Finally, the controls in Fig 2b are the substrates incubated in the absence of the enzyme **(we have included this missing information in the Fig 2 caption on p18).**

[1.9] Fig. 3 – Missing accession number details; inconsistent and in some cases incomplete labelling of species names.

We included additional strain codes (e.g. DSM or ATCC codes) for all species. There are 77 different genes in the clusters drawn in Fig. 3, which already extends to a full page. Given that it would not be feasible (legible) if we added >10 character labels (accession numbers) to each gene (arrow) in Fig 3, **we have added accession numbers to identify all of the gene products in supplementary Figs. 12 & 17 (SI p25 & 43)** The genome sequences of strains *S. kasugaensis* (DSM40819) and *S. netropsis* (DSM40846) that we re-sequenced have been deposited with NCBI and are embargoed. If this paper were to be accepted the accession codes for the genes in this strains will become available and will be added. The protein sequences of all genes in the aforementioned *S. kasugaensis* and *S. netropsis* BGCs are also provided in the supplementary sequences excel sheet.

[1.10] Fig. 4 – Chemical standard control traces are missing. Panels b and c appear to be the high-res mass versions of panel a (and are unlikely needed in the main panel of a Nature-level journal). Panel e is labelled "IC50" in a column that lists the names of strains; I think the label is meant for other columns. Standard error values appear to have been missed. Panel f appears to be missing the units. Panel g is an AlphaFold model; how reliable is this model?

Fig 4(a) is the HPLC of the *S. nodosus* (AmB producer) expressing the *KfuSV* subcluster using the *pSET-ermE** vector showing production of AmB-L-digitoxose. The controls are *S. nodosus* (wild-type) and *S. nodosus* with the empty *pSET-ermE** vector. The wavelength for HPLC detection is 405 nm which is very specific for the AmB chromophore. There are no other metabolites (peaks) in this strain that absorb at 405 nm. We isolated the AmB-L-digitoxose and fully characterised it by very detailed 2D NMR (Supplementary Fig 22). It is not feasible to include a chemical standard for AmB-L-digitoxose. Simple methylation of the C35-OH of AmB requires 8 chemical steps (<4% yield, see ED Fig. 1 in ref 6) and to stereoselectively install a L-digitoxose at C35 of AmB, by chemical synthesis, would require many more additional steps. **To address this point, we have included in Fig 4 caption that the HPLC detection wavelength is specific for the AmB chromophore and point to full NMR characterisation in the Supplementary Information.** We have also removed panel b and c as the referee suggests. We have re-labelled the table (now panel d) as requested. **The IC_{50} values are all derived from triplicate experiments which are reported for each individual strain and compound, with error bars included for all measurements (Supplementary Fig 45-46, p91-95). We have produced the IC_{50} data in the way recommended by European Committee for Antimicrobial Susceptibility Testing (EUCAST).** We have mentioned this in the experimental methods (p13) and cited the EUCAST guidelines (Ref. 53 & 54). Units have been added for panel f (now panel e). The alpha-fold model of PcsA is shown to help explain how this type of enzyme works (it is not simple to explain in words alone). The model is consistent with structures of related glutamine amidotransferases that have been determined by X-ray crystallography. There are no intrinsically disordered regions, or other sequence, within PcsA that AlphaFold may find difficult to predict, so there are no reasons why the structure would not be inaccurate.

[1.11] Suppl. Fig. 3 –This phylogenetic tree is odd, listing some branches with an enzyme name and others showing an unlabelled gene cluster. I'm not sure any other readers aside from the authors will be able to make use of these data, since it's unclear what is being shown, i.e. missing any labels. I don't totally follow the workflow in this Suppl. Fig., however, I do note that the "sub-clusters" from the three strains are clearly distinct in terms of the number of genes they contain. Are they all matching what is shown in Suppl. Fig. 2? Annotation is provided in SI Fig. 2 for nys but without elaboration. I therefore suggest the authors provide some context for the non-expert reader on whether their "discovery" of these sub-clusters remains novel, add citation of relevant work by others that allowed them to make their predictions for TDP-L-digitoxose biosynthesis (i.e. what does each encoded enzyme do in this pathway?), and add some clear elaboration of how the sub-clusters are distinct from one another, despite all producing TDP-L-digitoxose.

The phylogenetic tree was introduced to address the referee's previous question that there is likely to be more clusters with related GT that have the capability to produce novel polyenes with additional (second) sugars. This is definitely the case, but it is difficult to present in a single figure. If we presented the full analysis of each putative cluster (which we have already carried out), it would extend across many additional pages. Considering this, **we have revised the tree showing the accession codes of the related GTs and the strains where we find them (Supplementary Fig 3, p11), and edited the caption so the workflow is clearer**. The reader can then refer to the genome sequences of these strains, and identify loci of the additional clusters, and interrogate these further if they wish. The reviewer is correct that the annotation of *S. netropsis* (DSM40846) does not show a full set of TDP-L-digitoxose genes. We did mention this in the caption of Supplementary Fig 12, p25 and to address this we have added further discussion about TDP-L-digitoxose biosynthesis and additional citations to previous work (Supplementary Figs. 12a, 17a, ref 23 & 65). Also see the response 1.4 above.

Referee #2: The revised manuscript includes now the LC HRMS and NMR data with the corresponding spectra and chromatograms in the Supplementary Information. These crucial additions bring the compound characterization up to the required level of chemical characterization standards. The readers can now evaluate peak assignments, mass accuracy, and confirm the reported chemical structures as well as reproduce the experiments for any future studies in the field.

[2.1] I do, however, think that the organization of the added data is not always clear. Some spectra are not linked to specific compounds in the main text, and the numbering of Supplementary figures too dense and hence, confusing. It can be difficult to locate the exact dataset without having to go through multiple captions. Cross reading between the main text and Supplementary figures is still inefficient.

We were asked to carry out a great deal of additional experimental work, the vast majority of which we completed successfully. A total of 32 additional SI figures were required to address the earlier reviewers' comments, making 104 SI figures in total. As we need to mention all of these in the paper, the cross-referencing did become very dense. **To address this, we have consolidated some of the bioinformatics, NMR and other Supplementary Figs**. For example, instead of 8 separate kasufungin B NMR data figures, we have grouped these as a single Supplementary Fig. 18 (with the different spectra/data labelled a to j).

[2.2] Crucial reverse glycosylation assays, heterologous expression experiments, and NMR characterization of products have been added. These now provide convincing confirmation of the assigned glycosyltransferases and confirm the structures of the modified polyenes. The authors initially carried out assignments based on biosynthetic logic, which are very important and often strongly support the data. However, the essential evidence that has now been added provide direct experimental demonstration of enzyme activity and help confirm the product polyene structures. Adding RT-qPCR data also supports the biological interpretation by pointing out differences in production between strains. At the same time, the integration of these datasets into the main text is not clear and easy to understand and integrate. The linkage between specific figures and claims in the main text is sometime hard and challenging to follow and can be simplified and improved.

We think that the confusion locating the RT-qPCR data could be that some of it was presented in an Extended Data Figure, and some in a Supplementary Figure. **As stated above, we have reorganised the extended data and SI Figs to avoid the reader moving round the paper and SI as much. All RT-qPCR data is in a single revised Supplementary Fig. 11 (SI, p24).**

[2.3] More enzymatic assays with addition polyene substrates such as natamycin have been included. These valuable experiments show that the enzymatic platform is not restricted to a single polyene scaffold and can act on structurally diverse polyenes. In my opinion, this dramatically improves the scope of the work and now confirms the claim of broader applicability. I do however think that these very important results are mainly reported in the revised Supplementary Information and are not emphasized enough in the main text given their importance.

We agree that including more additional substrates improves the scope and broader applicability of the work, and we have emphasised this in the revised conclusion (p8). In balance with reviewer #1's comments that the amidotransferase enzyme portion is too in depth, and due to the word and page count, we feel it is difficult to include too much more discussion of this in the paper.

[2.3] Cytotoxicity assays at the cell-based level in HEK293, A549, and HEPG2 cell lines have been added in the revised study. These experiments expand the evaluation of toxicity beyond hemolysis. This information is now closer to accepted preclinical standards and further supports the claim that the modified compounds may have improved safety profiles. The data indicates reduced toxicity compared to the parent compound, which is an important result. Key quantitative values such as IC50 are not clearly presented to allow quick comparison across compounds.

We have included a table for all antifungal IC_{50} data and have similarly added a new table for the toxicity data, with human cell lines, that we measured. **Both can now be found in revised Extended Data Fig 7**.

[2.4] Mouse tolerability studies, pharmacokinetic analysis, and an in vivo infection model have also been added. These experiments fundamentally improve this study by clearly demonstrating that the modified polyenes are not only active in vitro but also tolerated and effective a rodent in vivo model. The pharmacokinetic analysis, including model fitting and diagnostic plots, now provides a basis for dosing. The infection model showing reduction in fungal burden adds important translational relevance and clearly demonstrates clinical development potential that may rise from this study. Pharmacokinetic analysis is presented mainly in the Supplementary Information file with limited explanation in the main text, which make interpretations inaccessible to a general multidisciplinary readership such as that of Nature. Some PK figures also do not clearly indicate sample size or variability directly on the plots.

The PK analysis was carried out to determine an optimal dosing regimen and has been contextualised in the main body of the text (P7 lines 14-22) with sampling details given in the methods section. **To enhance the**

prominence of this information, we have moved the PK analysis from SI into the paper, Extended Data Fig 9, with additional information including sample size and dosing times in the figure legend.

[2.5] Experiments addressing sterol interactions, including ergosterol-related analyses, have been added in the revised manuscript as well. This data contributes to a mechanistic explanation for reduced toxicity by linking structural modifications to changes in sterol affinity. This supports the interpretation of improved safety. However, despite their strength, these results are only briefly discussed in the main text, and their importance is not emphasized.

We were limited by the word length of the paper. However, we have consolidated some of the introduction (background) and taken some of technical discussion on bioinformatics and NMR analysis into SI (without losing any data or explanation of the NMR assignments or in silico work that we did). This has freed up more space where we discuss the lack of ergosterol binding **in the conclusion section, pointing out that more studies are required to define Nys34's alternative mechanism of action (p8, line 27-31).**

[2.6] The extended dose-response experiments and additional replicates that have been included improve the effectiveness of the statistical analysis of the biological conclusions and allows more reliable comparison between the modified polyenes. I do however suggest that a single table that lists key values such as IC50 or EC50 is added in the SI and referred to in the relevant places in the main text.

We have done this as stated above (see 2.3).

To conclude, I am highly supportive of the acceptance of this manuscript in Nature, provided that the authors address the key points outlined above. In my view, the work is already within the quality range expected for journals in the Nature family, and with a more focused and simplified presentation, it would be well positioned for a broader readership. The suggested revisions are aimed at improving clarity, accessibility, and impact, and once implemented, will further strengthen what is already a significant and highly appealing contribution.

Referee #3: In this work, the authors provide sufficient data to confirm that they identified two types of glycosyltransferases capable of attaching sugar to the unusual C35 position of a polyene, generating a promising candidate, Nys34, which exhibits potent antifungal activity along with low toxicity and high solubility. In the revised manuscript, **although the authors did not obtain a crystal structure of the glycosyltransferase to explain the mechanism, it is still acceptable to combine AlphaFold with point mutation analysis to propose a model**. Several issues have been raised after reviewing the revised manuscript.

[3.1] I am a little bit confused about the naming of the natural product in this paper, as also noted by reviewer 2. It appears that the two compounds identified in this paper share exactly the same structures as those previously identified as mandimycin and mandimycin B, originally described by Wang et al. (Nature, 2025, 640:743). In the present manuscript, however, these compounds are renamed as mycope**p**tins C and A, respectively. Although the authors provide a rationale for this nomenclature change—primarily to align with earlier terminology established for this biosynthetic pathway in 1977—I find this renaming neither necessary nor advisable, as it may in fact introduce confusion in the field for the following reasons. First, mandimycin represents a structurally and functionally distinct class of polyene macrolides, characterized by a unique 5+1 polyene motif and a mode of action that differs fundamentally from classical heptaene-containing polyenes. The original designation by Wang et al. appropriately reflects these distinguishing features, and this distinction is further supported by the data presented in the current manuscript. Second, the proposed nomenclature "mycope**p**tin" is not fully consistent with the historical nomenclature it seeks to reference (e.g., "mycoheptin") and does not clearly convey the relationship among compounds within the same class. From a nomenclature standpoint, this inconsistency may hinder rather than enhance clarity. More importantly, the suffix "-pe**p**tin" is conventionally associated with peptide-derived natural products (e.g., NRPs or RiPPs) and is rarely used for polyketide-type compounds such as polyene macrolides. Its use here is potentially misleading and may create unnecessary ambiguity for researchers working in natural product chemistry and biosynthesis. Therefore, I do not find sufficient justification for renaming these previously reported compounds, which have already been clearly defined and widely recognized in the literature. To maintain consistency and avoid confusion, I strongly recommend that the authors adhere to the nomenclature established in the prior 2025 Nature publication. "Mycope**p**tin C" and "mycope**p**tin A" should be restored to mandimycin and mandimycin B, respectively, and the newly identified compound "mycope**p**tin B" could be more appropriately designated as mandimycin C.

We did not use the suffix -pe**p**tin. The suffix -pe**n**tin for pentaene was used. Nevertheless, we understand the points the reviewer makes here, and we explained the rationale for the compound naming in our previous response. The original parent compound mycoheptin, we assume was named based on myco (referring to fungi) and its seven consecutive double bonds which constitute a heptaene. Given the mycoheptin is the parent compound produced by the strain *S. netropsis* (DSM40846), we were referring to the other congeners as mycope**n**tins (not mycope**p**tins) as they are derived from the same cluster/enzyme systems as mycoheptin but

are pentaenes not heptaenes (by virtue of one of the double bonds being reduced). We do, however, appreciate that having two sets of names for same structures could cause confusion. Therefore, **as the reviewer suggests we have used the name mandimycin (not mycopentin).** We have not named the other two compounds that are likely to be biosynthetic intermediates of mandimycin, as it is not clear if they are produced by the wild type mandimycin producer strain (DSM40259). Typically, names are given to the main pathway products isolated from the wild-type strain.

[3.2] The authors provided in vivo antifungal efficacy experiments in the revised manuscript, which is commendable. For in vivo studies of polyene antifungals, the therapeutic route is typically intravenous rather than intraperitoneal. Can the authors explain why they did not administer Nystain 34 through IV? Was it because of toxicity or formulation?

Our use of animal models is bound by legislative requirements and ethical constraints. In relation to this study, we need to consider the impact of dosing route on the welfare of the mice we are using. First in mouse studies often use intraperitoneal (IP) dosing, even though this route is rarely used clinically, as it is minimally stressful to the animals. The drug can be administered repeatedly (which is discouraged for IV administration in mice due to cumulative irritant effects) and generally avoids high peak plasma concentrations (Cmax) associated with IV injections that can be potentially toxic. An interesting discussion piece on the merits, or otherwise, of IP dosing can be reviewed in Al Shoyaib *et al. Pharm Res* **37**, 12 (2020) where they conclude that "*IP administration of drugs in experimental animals is a justifiable route for pharmacological and proof-of-concept studies where the goal is to evaluate the effect(s) of target engagement rather than properties of a drug formulation and/or its pharmacokinetics for clinical translation*" the latter of which is typically addressed at later stages of drug development. In several mouse models of infection described previously, AmB has been administered by intraperitoneal (IP) injection to limit toxicity e.g. Graybill *et al. Antimicrob Agents Chemother* **39**, 1885 (1995) and Wiederhold *et al Antimicrob Agents Chemother* **50**, 469 (2006). Nystain derivatives have also been shown to have favourable PK profiles in mice following IP administration, as reported by Lepak *et al*. *Antimicrob Agents Chemother* **68**, e0123424 (2024). These factors, combined with ethical considerations were the drivers for our choice of route of administration. **We have moved the PK studies to Extended Data Fig 9 and provided more IP dosing regimen in the figure caption (p32).**

[3.3] I note that Nys34 was generated by ethanolamine modification of Nystatin A3. Actually, in 2022, Tamar et al. (ACS Med. Chem. Lett., 2022, 13:182-187) performed similar experiments and obtained similar results, showing that C16-modified Nystatin analogues exhibited potent antifungal activity as well as significantly low toxicity and high water solubility. Therefore, does the sugar modification at the C35 position or the amide derivatives modification at the C16 position account for the observed effects in this case?

Nys34 has a C16-amide generated from serinol (not ethanolamine), and has the additional sugar moiety at C35, which is not present in the ethanolamide derivative (3) or any of the other polyenes described in Tamar's paper (Amir *et al*. ref. 30). Given that the compounds are all different and the experiments in Amir *et al* were conducted in a different way, and do not include any *in vivo* data, it is difficult to compare results. In referee #3's first review, they indicated that we needed to include "*in vivo* antifungal evaluation to draw valid conclusions." We did this and showed excellent *in vivo* data with a relevant mouse infection model. We had already stated in the introduction that modifications of the C16-carboxyl group of polyenes are known to reduce toxicity. We also discussed the work of Maji *et al.* 2023 (ref. 6) in the introduction, noting they produced a polyene with C16-amide which shows promising *in vivo* properties. **We have further emphasised the importance of amide modifications and referenced (Amir, *et al*. ref. 30), in the introduction (p2, line 25-28) in addition to discussing the paper of Maji 2023 (ref 6, p2, line 30-33).**

In our work we found that derivatives with additional C35-sugar and C16-amide modifications (Nys31-34) had superior antifungal activity, compared with the parent NysA1 and derivatives with just C16-amide modification (Nys11-14), across a wide range of different fungal pathogens. So, it is the case that the combination of additional sugar and amide groups is most effective. Although Amir *et al* indicate compound 3 (in ref.30) is less toxic, they also state that it is does not have higher potency that than the parent NysA1.
